# An umpolung-enabled copper-catalysed regioselective hydroamination approach to α-amino acids†

**DOI:** 10.1039/d1sc03692k

**Published:** 2021-07-27

**Authors:** Soshi Nishino, Masahiro Miura, Koji Hirano

**Affiliations:** Department of Applied Chemistry, Graduate School of Engineering, Osaka University Suita Osaka 565-0871 Japan k_hirano@chem.eng.osaka-u.ac.jp; Innovative Catalysis Science Division, Institute for Open and Transdisciplinary Research Initiatives (ICS-OTRI), Osaka University Suita Osaka 565-0871 Japan

## Abstract

A copper-catalysed regio- and stereoselective hydroamination of acrylates with hydrosilanes and hydroxylamines has been developed to afford the corresponding α-amino acids in good yields. The key to regioselectivity control is the use of hydroxylamine as an umpolung, electrophilic amination reagent. Additionally, a judicious choice of conditions involving the CsOPiv base and DTBM-dppbz ligand of remote steric hindrance enables the otherwise challenging C–N bond formation at the α position to the carbonyl. The point chirality at the β-position is successfully controlled by the Xyl-BINAP or DTBM-SEGPHOS chiral ligand with similarly remote steric bulkiness. The combination with the chiral auxiliary, (−)-8-phenylmenthol, also induces stereoselectivity at the α-position to form the optically active unnatural α-amino acids with two adjacent stereocentres.

## Abstract 2

A copper-catalysed regio- and enantioselective hydroamination of acrylates has been developed to afford the corresponding optically active unnatural α-amino acids.

## Graphical abstract

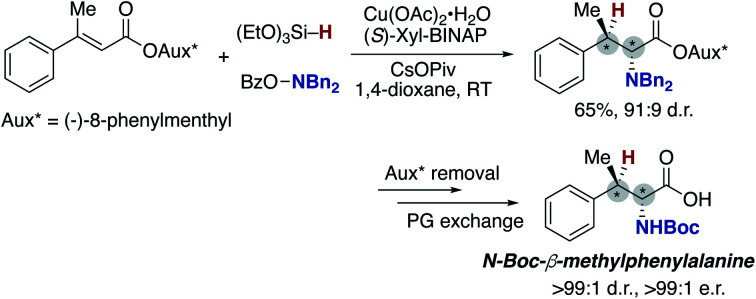

## Introduction

**Scheme 1:**
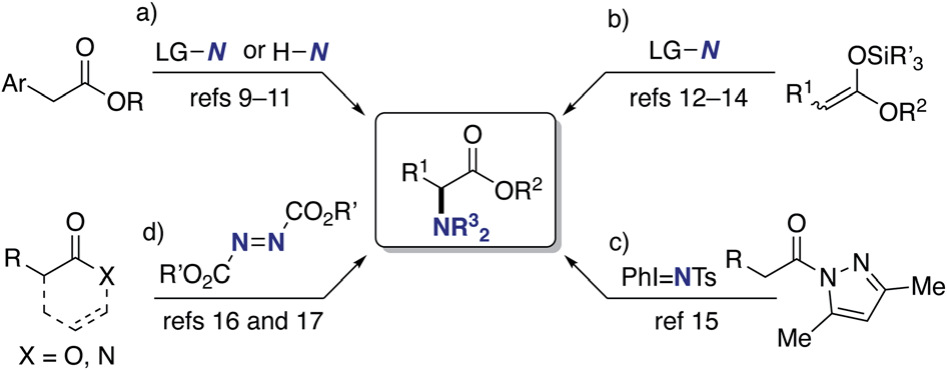
C–N bond forming approaches to α-amino acids. (a) Amination of phenylacetates, (b) amination of ketene silyl acetals, (c) amination of acylpyrazoles, and (d) amination with azo reagents. LG = leaving group, Ts = *p*-toluenesulfonyl.

α-Amino acids are prevalent structural motifs in many biologically active compounds and pharmaceutical agents, especially peptide drugs. In particular, unnatural α-amino acids have received significant attention because when they replace natural α-amino acids in the original drug structure, the potential for activity improvement and the discovery of new functions increases.^1^ Multicomponent couplings such as the Strecker, Ugi, and Petasis reactions are classical but the most powerful approaches to the aforementioned target structures.^2^ The catalytic hydrogenation of α-dehydroamino acid derivatives also provides promising access to unnatural α-amino acids.^3^ Additionally, the decoration of naturally occurring α-amino acids by the C–C bond forming reactions under phase-transfer,^4^ palladium,^5^ copper,^6^ and iridium/copper dual catalysis^7^ as well as cross-dehydrogenative-coupling (CDC) conditions^8^ has also been explored. On the other hand, the C–N bond formation at the α position to the carbonyl in carboxylic acid derivatives can also be a good alternative (Scheme 1). Vedejs reported the KO-*t*-Bu-mediated direct α-amination of phenylacetates with the electrophilic amination reagent (4-MeOC_6_H_4_)_2_(O)PO–NH_2_ (Scheme 1a).^9^ Shi also revealed that the related α-amination was possible with di-*tert*-butyldiaziridinone in the presence of copper salts.^10^ MacMillan developed the CuBr_2_/O_2_-catalysed ideal C–H/N–H coupling of phenylacetates and free NH amines through the transient formation of α-bromo esters.^11^ The copper-catalysed electrophilic amination of ketene silyl acetals with hydroxylamines^12^ or chloroamines^13^ can also access α-amino acids and was independently developed by our group and the Miura/Murakami research group (Scheme 1b). Kiyokawa and Minakata recently designed (diarylmethylene)amino-substituted hypervalent iodine(iii) reagents and succeeded in the amination of the same ketene silyl acetals under catalyst-free conditions.^14^ Yazaki and Ohshima achieved the copper-catalysed direct α-amination of acylpyrazoles as the carboxylic acid surrogates with the PhI = NTs nitrenoid source (Scheme 1c).^15^ Wasa^16^ and Sawamura/Shimizu^17^ groups independently developed boron-based catalyst systems for the α-amination of esters, amides, and free carboxylic acids with azodicarboxylates (Scheme 1d). Despite these certain advances, there are still several drawbacks; the substrates are limited to the relatively acidic C–H of phenylacetates (Scheme 1a); the preactivation of carboxylic acids by strong bases/silyl halides is inevitable (Scheme 1b); condensation with a special pyrazole-based directing group is necessary (Scheme 1c); the initial product is the hydrazine derivative, and thus the resultant N–N bond should be reductively cleaved to obtain the targeted α-amino acids (Scheme 1d). Moreover, the highly stereocontrolled process remains largely elusive. Thus, the concise and efficient synthesis of α-amino acids based on C–N bond formation is still a formidable challenge.^18^

**Scheme 2:**
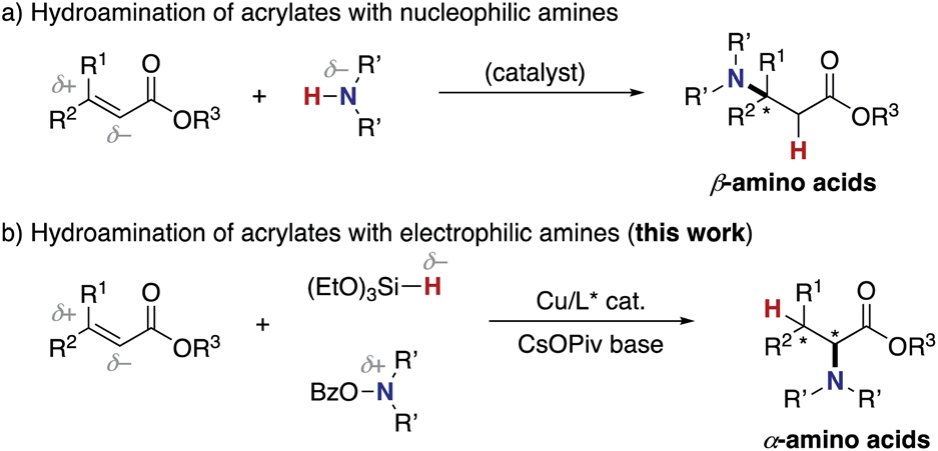
Hydroamination approaches to amino acids. (a) Usual hydroamination and (b) umpolung hydroamination. Bz = benzoate, Piv = *tert*-butylcarbonyl.

Meanwhile, the catalytic hydroamination of readily available α,β-unsaturated carboxylic acid derivatives such as acrylic acids also seems to be an attractive approach to amino acids. However, because of the innate polarity of α,β-unsaturated carbonyls and amines, the nucleophilic amino group generally adds at the electrophilic β-position, thus delivering the β-amino acids selectively (Scheme 2a).^19^ Therefore, in spite of its potential, α-amino acids are generally difficult to prepare by conventional hydroamination reactions. Herein, we report a copper-catalysed regioselective hydroamination of α,β-unsaturated esters with hydrosilanes and hydroxylamines to form α-amino acid derivatives with high regioselectivity (Scheme 2b). The key to successful regioselectivity control is the introduction of a polarity inversion concept, that is an umpolung strategy;^20^ the hydrosilane and hydroxylamine work as the nucleophilic hydrogen (hydride) and amino electrophile, respectively, to induce the desired α-amination selectivity. The judicious choice of the CsOPiv base and bisphosphine ligands of remote steric hindrance enables the otherwise challenging C–N bond formation at the α position to the carbonyl. The asymmetric induction at the β position is also possible by a similarly bulky Xyl-BINAP or DTBM-SEGPHOS chiral ligand. Moreover, the point chirality at the α-position is also successfully controlled by using an (−)-8-phenylmenthol auxiliary. Thus, unnatural α-amino acids with adjacent two stereocentres are obtained with high enantioselectivity. The detailed optimization studies, substrate scope, application to conjugation with bioactive amines, and preliminary mechanistic studies are disclosed herein.

## Results and discussion

**Scheme 3:**
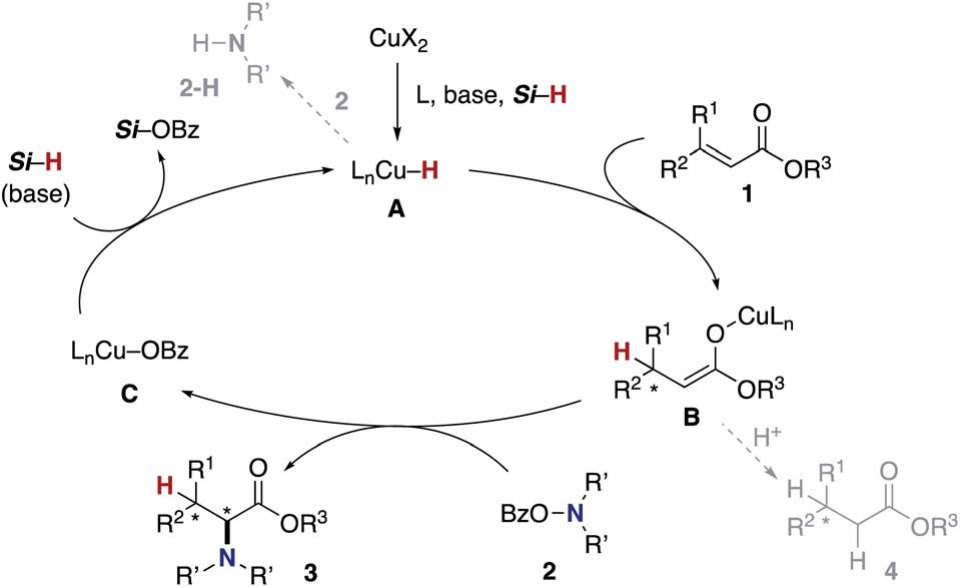
Working hypothesis.

**Scheme 4:**
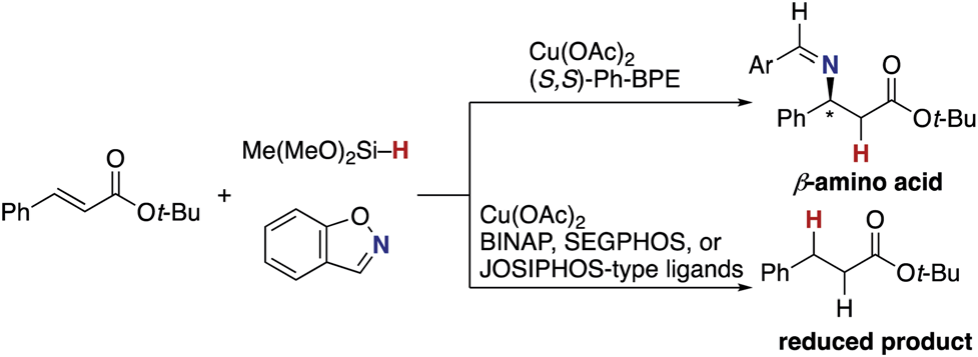
Attempt to synthesize α-amino acids by Guo and Buchwald.

The blueprint for the regioselective hydroamination of acrylates is based on the recent advances of copper-catalysed net hydroamination of alkenes with hydrosilanes and hydroxylamines, which was originally and independently developed by our group^21^ and the Buchwald research group.^22^ Our working hypothesis is shown in Scheme 3. A copper hydride species **A** is initially formed from the starting copper salt CuX_2_ and hydrosilane Si–H with the assistance of an external base. The acrylate **1** undergoes the regioselective 1,4-addition with the L_*n*_Cu–H **A** to afford the *O*-bound copper enolate **B**, where the regioselectivity is controlled by the innate electronic bias of acrylate **1** toward the nucleophilic copper hydride.^23^ Subsequent electrophilic amination^24^ with the *O*-benzoylhydroxylamine **2** delivers the desired α-amino acid derivative **3**.^25^ The concurrently formed copper benzoate **C** is converted back to the catalytically active copper hydride **A***via* direct metathesis with the hydrosilane Si–H or base-assisted stepwise ligand exchange. If the appropriate chiral ligand (L) is employed, enantioselectivity is induced in the 1,4-addition step (**A** to **B**) to control the point chirality at the β-position. The additional point chirality at the α-position can also be controlled by the proximal chirality at the β-position (substrate control), the chirality of the ligand on copper (catalyst control), or both, giving optically active α-amino acids with two adjacent stereocentres. However, there is a considerable challenge associated with the aforementioned reaction design; Guo and Buchwald recently reported the related attempt of the copper-catalysed regioselective hydroamination of cinnamates with 1,2-benzisoxazole as the electrophilic amino source (Scheme 4).^26*a*^ While the (*S*,*S*)-Ph–BPE ligand successfully gave the corresponding β-amino acid derivative, the α-amino acid was not obtained at all, and instead the simply reduced byproduct **4** was observed exclusively. Around the same time, the research group of Xiong, Guan, and Zhang also reported the related hydroamination of cinnamates, in which the corresponding α-amino acids were detected but as the minor product.^26*b*^ Thus, the development of suitable reaction conditions for the suppression of the undesired protonation from the copper enolate **B** is the most important but challenging issue (**B** to **4**). An additional potent side reaction is the reductive N–O bond cleavage of **2** with the copper hydride **A**, just consuming the electrophilic amination reagent (**A** and **2** to **2-H**). Namely, the copper hydride **A** should react with the unsaturated ester **1** preferably over the hydroxylamine **2**.

Our optimization studies commenced with (*E*)-β-methylcinnamate (*E*)-**1a** and *O*-benzoly-*N*,*N*-dibenzylhydroxylamine (**2a**) to identify the suitable ligand, base, and solvent in the presence of Cu(OAc)_2_ and polymethylhydrosiloxane (PMHS; Table 1). The initial attempt to apply our previous optimal conditions for the styrene hydroamination (CF_3_-dppbz (dppbz = 1,2-bis(diphenylphosphino)benzene) ligand and the LiO-*t*-Bu base)^21*a*^ remained unsuccessful; similar to the aforementioned result in Scheme 4, just saturated ester **4a** was observed (entry 1). Additional screening of ligands and solvents was also performed, but the use of LiO-*t*-Bu only afforded the simply reduced product regardless of other reaction parameters. Thus, we then switched attention to conditions including the bulky dppbz-type ligand, namely, DTBM-dppbz,^27^ the CsOAc base, and 1,4-dioxane solvent, which were uniquely effective for the related hydroamination of 1-trifluoromethylalkenes.^21*f*^ Gratifyingly, the desired β-methyl-α-amino acid derivative **3aa** ^28^ was formed in 62% ^1^H NMR yield (*syn*/*anti* = 44 : 56) albeit with the concomitant formation of **4a** in 43% (entry 2). Prompted by this preliminary but intriguing result, several acetate-type bases were investigated (entries 3–7), with CsOPiv proving to be best in terms of efficiency and diastereoselectivity. As a general trend, the yield increased with increasing the size of the counter cation (Cs > K > Na). Other cesium bases such as Cs_2_CO_3_ and CsF showed lower performance (entries 8 and 9). The reaction also proceeded even in the absence of external bases, but the yield of **3aa** largely dropped (entry 10). Even with the DTBM-dppbz ligand, the Li- or NaO-*t*-Bu bases totally shut down the formation of **3aa** (entries 11 and 12). We next examined the effect of hydrosilanes; some alkoxy-substituted hydrosilanes provided the desired **3aa**, with (EtO)_3_SiH giving the α-amino acid **3aa** in a comparable yield with a slightly better *syn*/*anti* ratio (entry 13). As far as we tested, the reaction was less dependent on the copper catalyst precursor, but Cu(OAc)_2_·H_2_O slightly improved the yield (entry 14). On the other hand, the substituent on the aromatic ring in the dppbz-type ligand was critical; the parent dppbz and *ortho*-substituted *o*-Me-dppbz resulted in much less productivity (entries 15 and 16) whereas *para*- and *meta*-substituted dppbzs gave the α-amino acid **3aa** in moderate to good yields, except for the electron-withdrawing *p*-CF_3_-dppbz (entries 17–22). The observed better performance of dppbzs of remote steric hindrance can be associated with the attractive London dispersion,^29^ which accelerates the C–N bond formation (**B** to **C** and **3** in Scheme 3) as well as the addition step (**A** to **B** in Scheme 3). The yield of **3aa** further increased at a higher reaction concentration (entry 23). The final investigation of reaction stoichiometry revealed that the use of **2a** as the limiting agent was the best, and the desired **3aa** was isolated in 92% yield with 42 : 58 *syn*/*anti* ratio (entry 24). Additional observations are to be noted; other bidentate/monodentate phosphine and NHC ligands mainly formed the reduced **4a** or recovered the starting **1a** intact; the solvent screening uncovered that **3aa** was formed uniquely in 1,4-dioxane; no conversion was observed in the absence of any copper salts or ancillary ligands; other leaving groups on the nitrogen were tested, but OBz was identified to be the best in terms of reactivity and availability (see the ESI† for more detailed optimization studies).

**Table 1:** Optimization studies for copper-catalysed regioselective hydroamination of acrylate (*E*)-**1a** with *O*-benzoylhydroxylamine **2a** for synthesis of α-amino acid **3aa** a

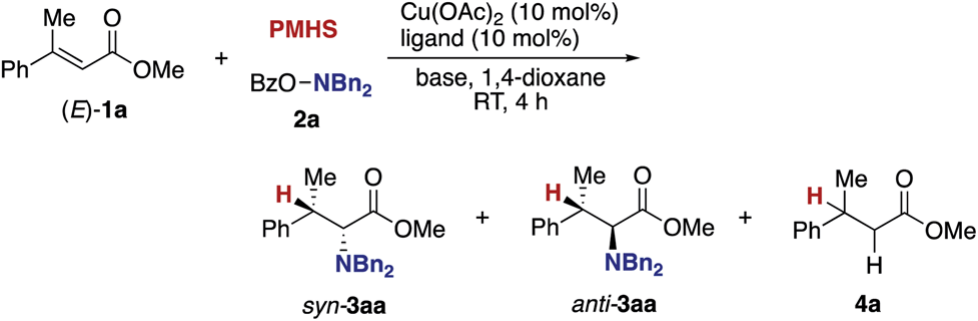				
Entry	Ligand	Base	Yield of **3aa** (%), *syn*/*anti*^b^	Yield of **4a** c (%)
1	CF_3_-dppbz	LiO-*t*-Bu	0, —	9
2	DTBM-dppbz	CsOAc	62, 44 : 56	43
3	DTBM-dppbz	KOAc	38, 47 : 53	57
4	DTBM-dppbz	NaOAc	26, 50 : 50	63
5	DTBM-dppbz	CsOPiv	71, 42 : 58	29
6	DTBM-dppbz	KOPiv	70, 44 : 56	30
7	DTBM-dppbz	NaOPiv	0, —	0
8	DTBM-dppbz	Cs_2_CO_3_	30, 37 : 67	59
9	DTBM-dppbz	CsF	34, 44 : 56	64
10	DTBM-dppbz	None	22, 32 : 68	55
11	DTBM-dppbz	LiO-*t*-Bu	0, —	51
12	DTBM-dppbz	NaO-*t*-Bu	0, —	49
13^d^	DTBM-dppbz	CsOPiv	71, 38 : 62	30
14^d^^,^^e^	DTBM-dppbz	CsOPiv	77, 42 : 58	27
15^d^^,^^e^	dppbz	CsOPiv	10, 40 : 60	67
16^d^^,^^e^	*o*-Me-dppbz	CsOPiv	0, —	0
17^d^^,^^e^	MeO-dppbz	CsOPiv	21, 48 : 52	80
18^d^^,^^e^	*p*-CF_3_-dppbz	CsOPiv	0, —	0
19^d^^,^^e^	*p-t*-Bu-dppbz	CsOPiv	32, 41 : 59	73
20^d^^,^^e^	CF_3_-dppbz	CsOPiv	46, 41 : 59	55
21^d^^,^^e^	*t*-Bu-dppbz	CsOPiv	47, 38 : 62	53
22^d^^,^^e^	TMS-dppbz	CsOPiv	26, 42 : 58	65
23^d^^,^^e^^,^^f^	DTBM-dppbz	CsOPiv	82, 44 : 56	19
24^d^^,^^e^^,^^f^^,^^g^	DTBM-dppbz	CsOPiv	99, 38 : 62 (92, 42 : 58)	84
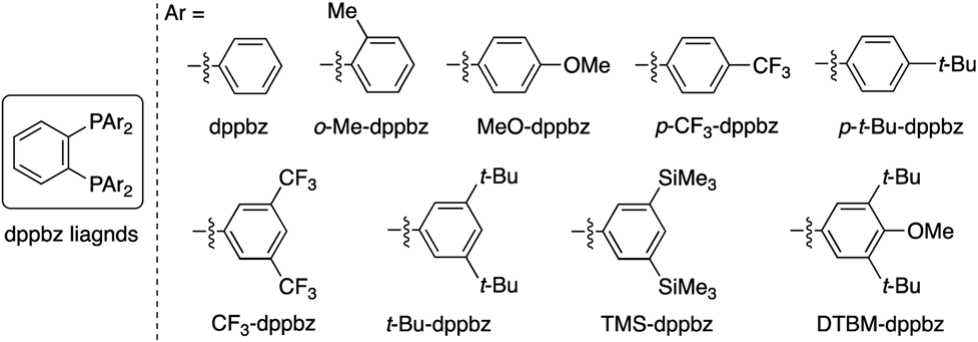				

a Conditions: Cu(OAc)_2_ (0.025 mmol), ligand (0.025 mmol), (*E*)-**1a** (0.25 mmol), **2a** (0.38 mmol), PMHS (0.75 mmol based on Si–H), base (0.75 mmol), solvent (1.5 mL), RT, 4 h, N_2_.

b Estimated by ^1^H NMR based on 0.25 mmol with CH_2_Br_2_ as the internal standard. The *syn*/*anti* ratio is determined in the crude mixture. Isolated yield is in parentheses.

c Estimated by ^1^H NMR based on 0.25 mmol with CH_2_Br_2_ as the internal standard.

d With (EtO)_3_SiH instead of PMHS.

e With Cu(OAc)_2_·H_2_O instead of anhydrous Cu(OAc)_2_.

f In 1,4-dioxane (1.0 mL).

g With **1a** (0.50 mmol) and **2a** (0.25 mmol).

**Scheme 5:**
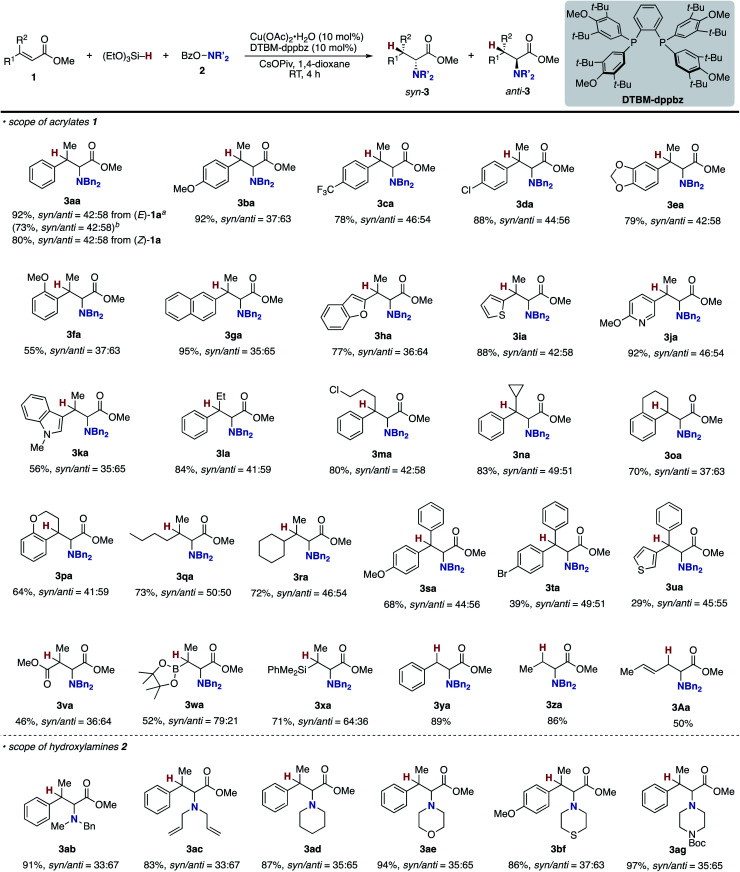
Scope of copper-catalysed regioselective hydroamination of acrylates **1** with *O*-benzoylhydroxylamines **2** for synthesis of α-amino acids **3**. Conditions: Cu(OAc)_2_·H_2_O (0.015 mmol), DTBM-dppbz (0.015 mmol), **1** (0.30 mmol), **2** (0.15 mmol), (EtO)_3_Si–H (0.45 mmol), CsOPiv (0.45 mmol), 1,4-dioxane (0.60 mL), RT, 4 h, N_2_. Isolated yields are shown. ^*a*^ On a 0.25 mmol scale. ^*b*^ On a 1.0 mmol scale.

With optimal conditions in hand (entry 24 in Table 1), we next examined the substrate scope of the umpolung-enabled regioselective hydroamination of acrylates toward α-amino acids (Scheme 5). The reaction was compatible with the electron-donating methoxy, electron-withdrawing trifluoromethyl, and chloro groups at the *para* position of the phenyl ring in the model substrate **1a** to form the corresponding β-methylphenylalanine derivatives **3ba–da** in good yields. The methylenedioxy substituent was also tolerated (**3ea**), while the *ortho*-substitution was somewhat detrimental to the reaction (**3fa**). The substrates that bear higher fused naphthalene and heteroaromatic benzofuran, thiophene, and pyridine all worked well to deliver the targeted α-amino acids **3ga–ja** in 77–95% yields. Additionally, the biologically interesting β-methyltryptophan^30^**3ka** was accessible. The copper catalysis accommodated some other bulkier alkyl substituents at the β-position; the ethyl-, chloropropyl-, and cyclopropyl-substituted cinnamates underwent the regioselective hydroamination smoothly (**3la–na**). Moreover, the cyclic systems could also be employed (**3oa** and **3pa**). The successful conversion of β,β-dialkyl-substituted acrylates to deliver the corresponding isoleucine derivatives **3qa** and **3ra** in synthetically useful yields is particularly notable. The β,β-diaryl substitution pattern was also viable (**3sa–ua**) albeit with somewhat lower efficiency. The ester, boryl, and silyl functionalities at the β-position were amenable to the regioselective hydroamination, and the biologically interesting aspartic acid **3va**, β-boryl-α-amino acid **3wa**,^31^ and β-silyl-α-amino acid **3xa** ^32^ were obtained in acceptable yields. Uniquely in the case of **3wa** and **3xa**, the *syn*-isomer was mainly formed because of the intramolecular oxygen to boron or silicon coordination in the *O*-bound copper enolate intermediate (**B** in Scheme 3).^33^ On the other hand, the reaction of the simple cinnamate and crotonate also proceeded without any difficulties to form **3ya** and **3za** in high yields. Different from the work by Guo and Buchwald,^26^ the regioisomeric β-amino acid was not detected at all in the hydroamination of cinnamate **1y**. The α,β,γ,δ-unsaturated sorbate was also applicable, and the corresponding 1,4-adduct **3Aa** was formed exclusively.

Several acyclic and cyclic *O*-benzoylhydroxylamines **2** underwent copper-catalysed hydroamination; *N*-benzyl-*N*-methylamine, *N*,*N*-diallylamine, piperidine, morpholine, thiomorpholine, and piperazine all were adopted in the reaction to afford the corresponding α-amino acids **3ab–ae**, **3bf**, and **3ag** in good to excellent yields. Additionally it is worth noting that (1) the reaction could also be conducted on a 1.0 mmol scale (**3aa**); (2) when the (*Z*)-type substrate was employed, the yield was slightly lower, but the same *syn*/*anti* ratio was observed (**3aa**), thus supporting the intermediacy of the common *O*-bound copper enolate (**B** in Scheme 3).^34^

The aforementioned success prompted us to explore enantioselective conditions by the judicious choice of ancillary chiral ligands (Table 2). Given the positive effects of the DTBM substituent observed in Table 1, we first investigated (*R*)-DTBM-BINAP, -SEGPHOS, and -MeO-BIPHEP in conjunction with a Cu(OAc)_2_·H_2_O catalyst. The DTBM-SEGPHOS ligand promoted the reaction to form **3aa** in 60% isolated yield with 42 : 56 *syn*/*anti* ratio and 99 : 1 e.r. for each diastereomer (entry 2), while no conversion occurred in the presence of DTBM-BINAP and -MeO-BIPHEP (entries 1 and 3). Intriguingly, the relatively small (*R*)-DM-SEGPHOS also showed high enantioselectivity (98 : 2 e.r.) albeit with somewhat lower yield of **3aa** (entry 4). On the other hand, the parent (*R*)-SEGPHOS largely decreased the yield (entry 5). Inspired by the comparable performance of DM-SEGPHOS, (*R*)-Xyl-BINAP and parent (*R*)-BINAP were also tested (entries 6 and 7). Gratifyingly, the better isolated yield and similarly high enantioselectivity were obtained with the (*R*)-Xyl-BINAP ligand (81% yield and 97 : 3 e.r.; entry 6). Additional screening of copper salts revealed that the combination of CuCl/(*R*)-DTBM-SEGPHOS resulted in better conversion than that of Cu(OAc)_2_·H_2_O/(*R*)-DTBM-SEGPHOS (entries 8 *vs.* 1) whereas in the case of (*R*)-Xyl-BINAP, CuCl showed slightly lower activity than Cu(OAc)_2_·H_2_O (entries 9 *vs.* 6). On the basis of the above optimization studies, conditions with Cu(OAc)_2_·H_2_O and (*R*)-Xyl-BINAP were identified to be the best from the viewpoints of catalytic activity and enantioselectivity (entry 6).^35^ The *syn*/*anti* ratio was low, but both isomers could be separated to each other by chromatographic purification. The relative and absolute configurations were assigned by comparison of ^1^H NMR spectra and specific rotation with the reported values after the derivatization. Given the high enantioselectivity also in the reduced byproduct **4a**, the point chirality at the β-position was well controlled but not at the α-position (see the ESI† for details).

**Table 2:** Optimization studies for copper-catalysed regio- and enantioselective hydroamination of (*E*)-**1a** with **2a** for asymmetric synthesis of α-amino acid **3aa** a

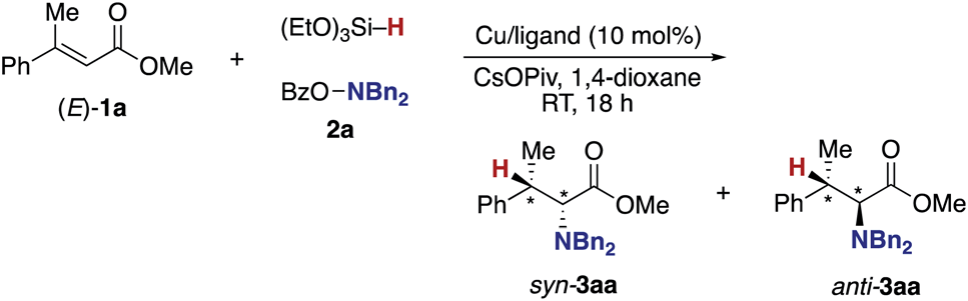				
Entry	Cu/ligand	Yield of **3aa** (%), *syn*/*anti*^b^	e.r.^c^	
			*syn*	*anti*
1	Cu(OAc)_2_·H_2_O/(*R*)-DTBM-BINAP	0, —	—	—
2	Cu(OAc)_2_·H_2_O/(*R*)-DTBM-SEGPHOS	60, 44 : 56	99 : 1	99 : 1
3	Cu(OAc)_2_·H_2_O/(*R*)-DTBM-MeO-BIPHEP	0, —	—	—
4	Cu(OAc)_2_·H_2_O/(*R*)-DM-SEGPHOS	42, 44 : 56	98 : 2	98 : 2
5	Cu(OAc)_2_·H_2_O/(*R*)-SEGPHOS	18,^d^ 44 : 56	n.d.	n.d.
6	Cu(OAc)_2_·H_2_O/(*R*)-Xyl-BINAP	81, 43 : 57 (31, 41)^e^	97 : 3	97 : 3
7	Cu(OAc)_2_·H_2_O/(*R*)-BINAP	23, 44 : 56	94 : 6	94 : 6
8	Cu(OAc)_2_·H_2_O/(*R*)-DTBM-SEGPHOS	73, 42 : 58	96 : 4	96 : 4
9^f^	CuCl/(*R*)-Xyl-BINAP	65, 43 : 57	97 : 3	97 : 3
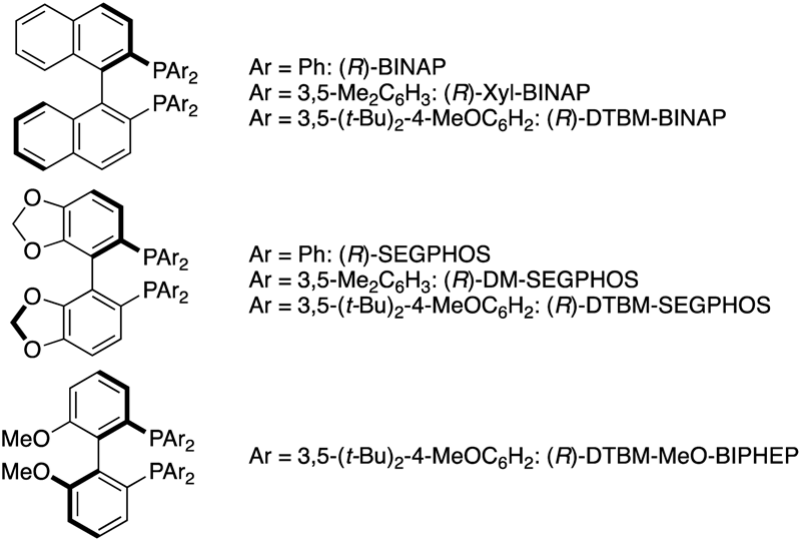				

a Conditions: Cu (0.015 mmol), ligand (0.015 mmol), (*E*)-**1a** (0.30 mmol), **2a** (0.15 mmol), (EtO)_3_Si–H (0.45 mmol), CsOPiv (0.45 mmol), 1,4-dioxane (0.60 mL), RT, 18 h, N_2_.

b Isolated yields are shown. The *syn*/*anti* ratio is determined in the crude mixture.

c The enantiomeric ratios (e.r.) were determined by HPLC analysis on a chiral stationary phase.

d ^1^H NMR yield.

e The isolated yields of *syn*-**3aa** and *anti*-**3aa** after the separation.

f 4 h. n.d. = not determined.

**Scheme 6:**
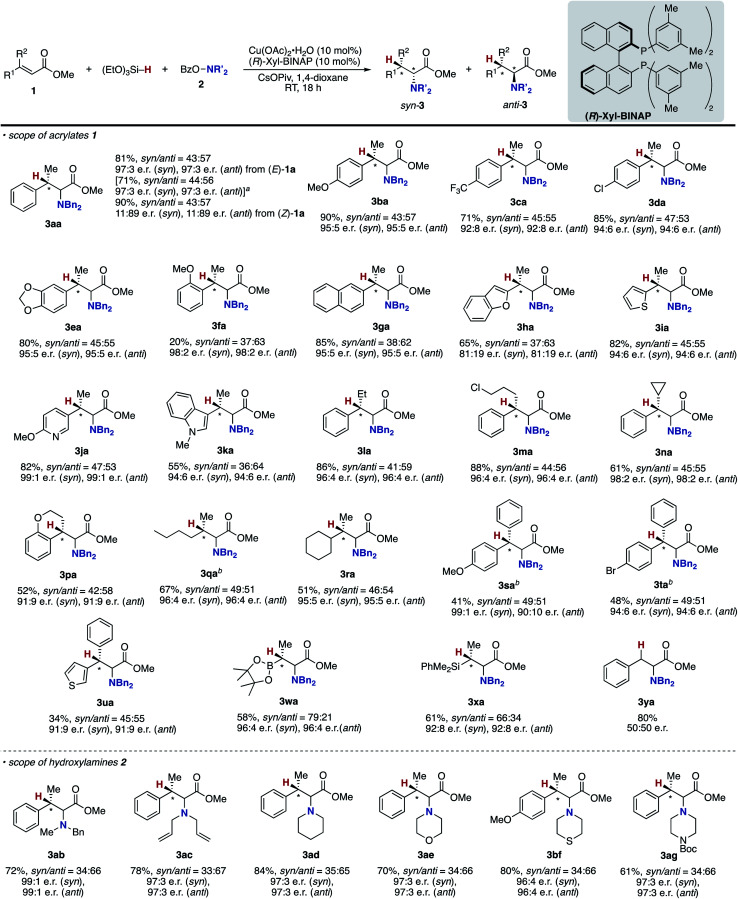
Scope and limitation of copper-catalysed regio- and enantioselective hydroamination of acrylates **1** with *O*-benzoylhydroxylamines **2** for asymmetric synthesis of α-amino acids **3**. Conditions: Cu(OAc)_2_·H_2_O (0.015 mmol), (*R*)-Xyl-BINAP (0.015 mmol), **1** (0.30 mmol), **2** (0.15 mmol), (EtO)_3_Si–H (0.45 mmol), CsOPiv (0.45 mmol), 1,4-dioxane (0.60 mL), RT, 18 h, N_2_. Isolated yields are shown. ^*a*^ On a 1.0 mmol scale. ^*b*^ With CuCl/(*R*)-DTBM-SEGPHOS instead of Cu(OAc)_2_·H_2_O/(*R*)-Xyl-BINAP.

Under conditions of entry 6 in Table 2, a variety of β-methylcinnamates underwent regio- and enantioselective hydroamination to form the corresponding β-methylphenylalanine derivatives **3ba–ga** in good yields with 92 : 8–98 : 2 e.r. (Scheme 6). Similarly under the nonenantioselective conditions, heteroaromatic substituents were also compatible to deliver the hydroaminated products with high enantioselectivity, except for the benzofuran substrate (**3ha–ka**). The asymmetric catalysis accommodated several other alkyl substituents at the β-position (**3la–na** and **3pa**) as well as the β,β-dialkyl substitution (**3qa** and **3ra**). In particular, both diastereomers of isoleucine derivative **3qa** were obtained with high enantiopurity. In the reaction of β,β-diaryl-substituted acrylates, the yield was somewhat lower, but the high enantiomeric ratio still remained (**3sa–ua**). Moreover, the β-boryl- and β-silyl-α-amino acids **3wa** and **3xa** were successfully synthesized in enantioenriched forms. In contrast, the simple methyl cinnamate formed the completely racemic product **3ya**, thus suggesting almost no control of point chirality at the α-position by the catalyst. As the amino sources, not only the acyclic but also cyclic hydroxylamines were readily and stereoselectively coupled with the β-methylcinnamates to afford the corresponding optically active α-amino acids **3ab–ae**, **3bf**, and **3ag** with high enantiomeric ratios (96 : 4–99 : 1 e.r.). In some cases (**3qa**, **3sa**, and **3ta**), the combination of CuCl/(*R*)-DTBM-SEGPHOS instead of Cu(OAc)_2_·H_2_O/(*R*)-Xyl-BINAP showed better performance from the viewpoints of efficiency and enantioselectivity. The enantioselective reaction could also be performed on a 1.0 mmol scale (**3aa**), indicating the good reliability and reproducibility of the asymmetric copper catalysis.

On the other hand, the stereoisomeric (*Z*)-**1a** was converted to the opposite enantiomers with a moderate enantiomeric ratio (**3aa**), supporting that the enantioface selection in the reaction of the copper hydride and acrylate **1** (**A** to **B** in Scheme 3) occurs in the 1,4-addition manner rather than the 1,2-insertion.^36^

**Fig. 1:**
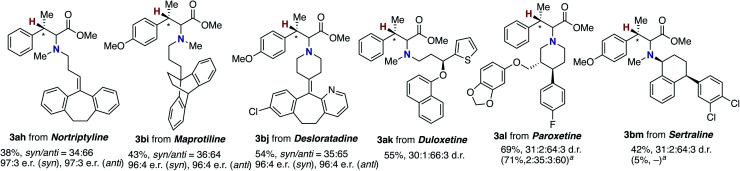
Modification of biologically active amines by conjugation with acrylate **1a** through coper-catalysed enantioselective hydroamination. For conditions, see the footnote in Scheme 6. ^*a*^ With (*S*)-Xyl-BINAP instead of (*R*)-Xyl-BINAP.

The newly developed asymmetric copper catalysis was applicable to the derivatization of several biologically active alkylamines (Fig. 1). Nortriptyline and maprotiline, antidepressant drugs, were conjugated with the β-methylcinnamate **1a** and **1b** under the (*R*)-Xyl-BINAP-ligated asymmetric copper catalysis to form the corresponding α-amino acids **3ah** and **3bi**, respectively, with high enantioselectivity. In a similar manner, desloratadine, an antihistamine agent, was successfully modified with **1b**, where the heterocyclic pyridine and aryl-Cl moiety were tolerated (**3bj**). Moreover, the chiral amines including duloxetine (antidepressant and anticonvulsant), paroxetine, and sertraline (selective serotonin reuptake inhibitors) were viable substrates, giving the highly functionalized α-amino acids **3ak**, **3al**, and **3bm** with acceptable diastereomeric ratios (d.r.). Notably, the α-chiral amine, sertraline, showed a significant match/mismatch phenomenon and resulted in almost no formation of the aminated product in the presence of (*S*)-Xyl-BINAP, while the reaction of paroxetine that bears the chiral centres at the remote positions proceeded smoothly even with (*S*)-Xyl-BINAP to furnish the product with the opposite stereoselectivity.

**Scheme 7:**
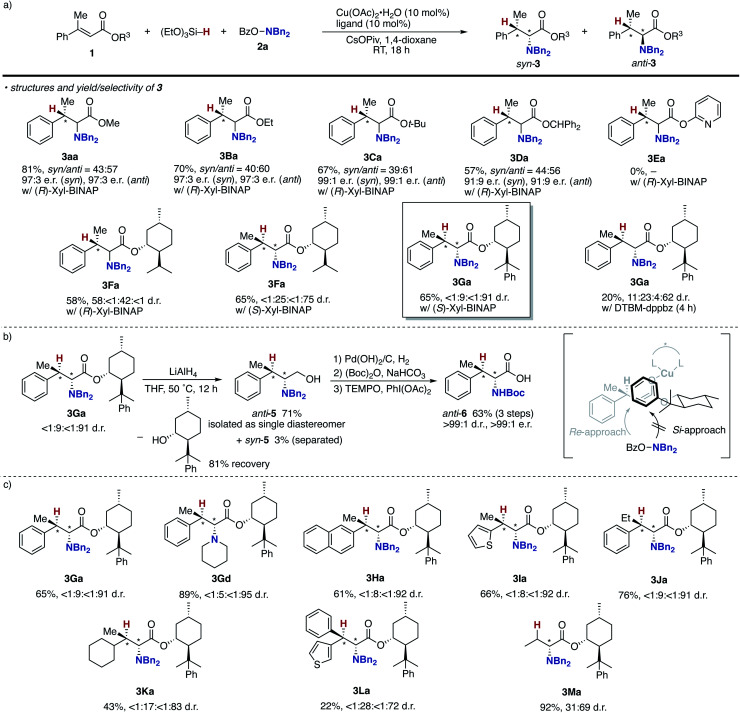
Attempts to control point chirality at the α-position. (a) Identification of alkyl groups in acrylate **1**, (b) removal of auxiliary and additional transformations, and (c) substrate scope.

As mentioned above, the Cu/Xyl-BINAP catalyst system successfully controlled the point chirality at the β-position but not at the α-position. Given the intermediacy of the *O*-bound copper enolate (**B** in Scheme 3), the steric and electronic effects of the alcohol moiety in **1** (R^3^ of **B** in Scheme 3) can affect the stereochemical outcome. Accordingly, several alkyl and aryl esters were prepared and subjected to the enantioselective conditions (Scheme 7a). Unfortunately, the ethyl, *tert*-butyl, and diphenylmethyl esters showed negligible effects on the stereoselectivity, and the corresponding α-amino acids were formed with low to moderate diastereomeric ratios similar to the methyl ester model substrate (**3Ba–Da***vs.***3aa**). The additionally coordinating pyridyl ester did not undergo hydroamination at all (**3Ea**). We then switched attention to the use of chiral alcohol, namely, the chiral auxiliary for improvement of the diastereoselectivity. Pleasingly, the l-(−)-menthol skeleton was found to be the promising candidate to increase the diastereomeric ratio to 25 : 75 (**3Fa**), only when combined with (*S*)-Xyl-BINAP. The value of d.r. was further improved to 9 : 91 with the assistance of readily prepared (−)-8-phenylmenthol^37^ (**3Ga**). The achiral DTBM-dppbz resulted in poor reactivity and moderate stereoselectivity, thus suggesting the necessity of double asymmetric induction arising from the chiral ligand and auxiliary. Treatment with LiAlH_4_ readily converted **3Ga** to the corresponding chiral 1,2-aminoalcohol, and the major *anti*-**5** could be isolated in a pure form with recovery of the chiral auxiliary (Scheme 7b). Subsequent protecting group exchange on nitrogen and TEMPO oxidation afforded the Boc-protected β-methylphenylalanine *anti*-**6** with >99 : 1 d.r. and >99 : 1 e.r. (see the ESI† for detailed stereochemical assignment). Given the absolute configuration of *anti*-**6**, the *Si*-face of copper enolate is efficiently blocked by the bulky PhMe_2_C group, and the hydroxylamine **2a** selectively approaches from the *Re*-face to produce the observed stereoisomer. The Xyl-BINAP/8-phenylmenthol double asymmetric induction strategy was applicable to the cyclic amine (**3Gd**) as well as other β-alkyl-substituted cinnamate derivatives (**3Ha–Ja**) to furnish the corresponding α-amino acids with good diastereoselectivity (>10 : 90; Scheme 7c). The β,β-dialky and -diaryl substrates (**3Ka** and **3La**) could also be employed albeit with slightly reduced stereoselectivity. On the other hand, the simple crotonate resulted in moderate stereochemical induction (**3Ma**). Even with the achiral DTBM-dppbz, the β-monosubstituted **1M** gave **3Ma** with the same 31 : 69 d.r. (data not shown), thus suggesting that the point chirality at the α-position is controlled just by the steric repulsion between the 8-phenylmenthol auxiliary and the substituent at the β-position without influence of the phosphine ligand on copper.

**Scheme 8:**
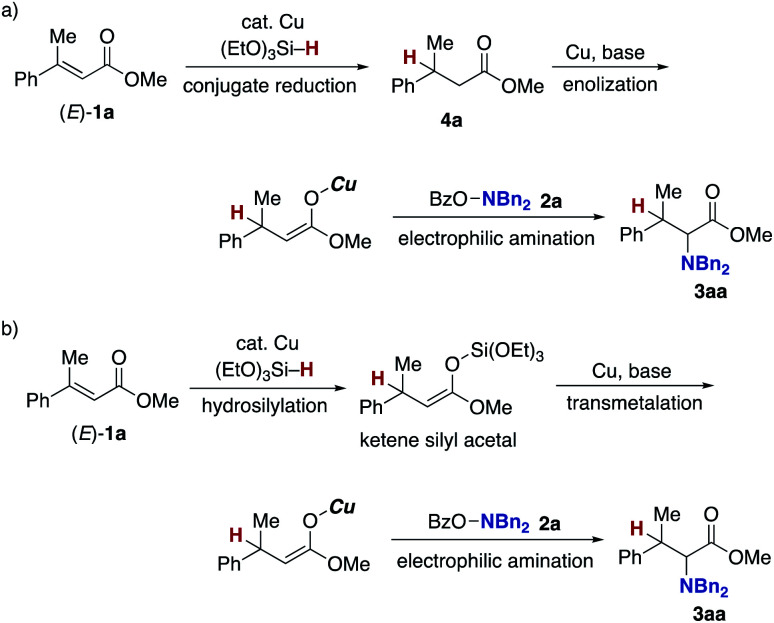
Two other possible pathways in C–N bond formation. (a) Conjugate reduction/enolization/electrophilic amination pathway and (b) hydrosilylation/transmetalation/electrophilic amination pathway.

**Scheme 9:**
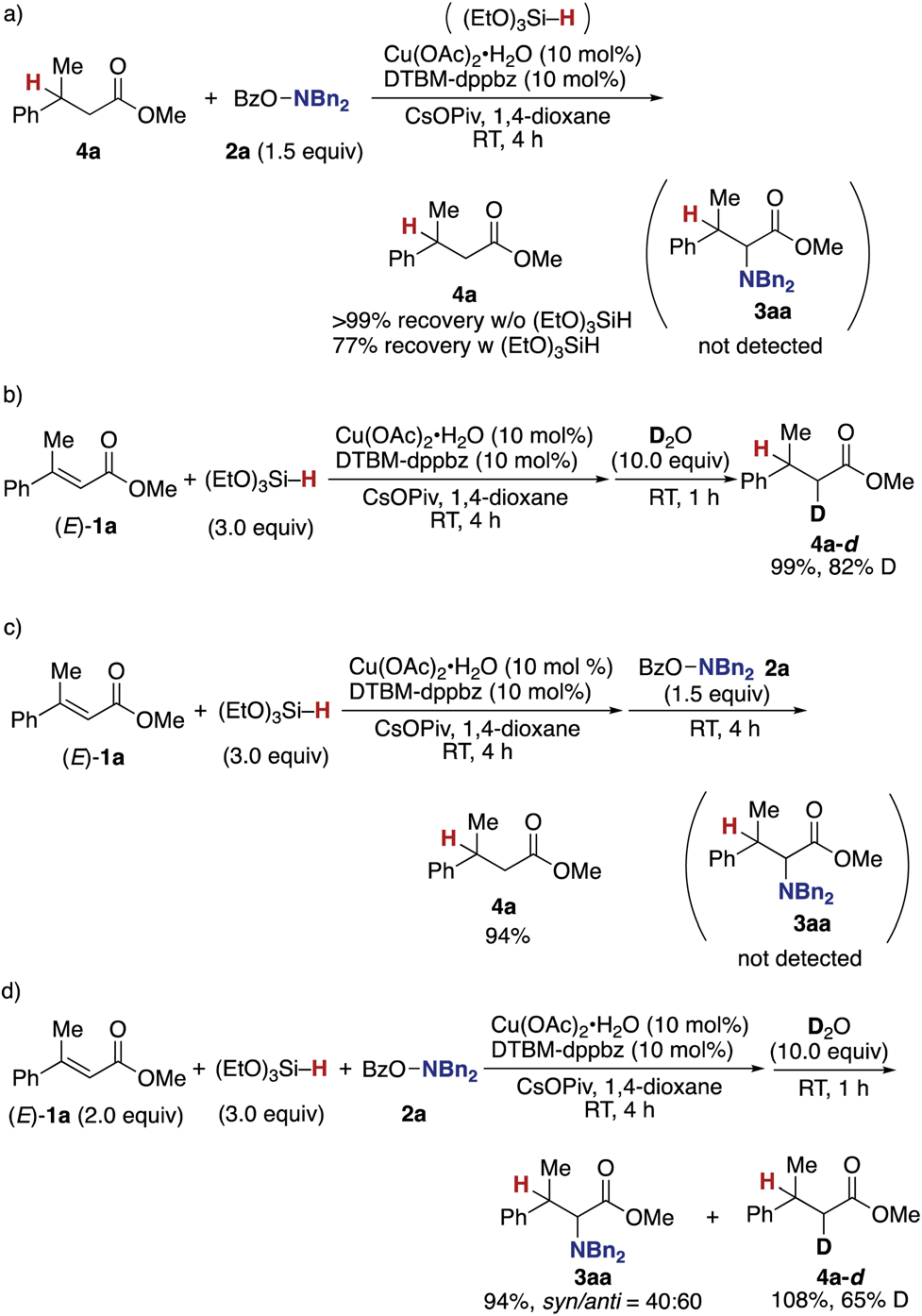
Control experiments. (a) Possibility of **4a** as intermediate and (b)–(d) possibilities of ketene silyl acetal as intermediate.

Finally mechanistic studies were implemented. In Scheme 3, we propose C–N bond formation by the reaction of the hydroxylamine **2** and copper enolate **B** directly formed through the 1,4-addition of the copper hydride **A** to the acrylate **1**, but there are two other potential pathways: one is the stepwise conjugate reduction/enolization/electrophilic amination (Scheme 8a) and another is the hydrosilylation/transmetalation from Si to Cu/electrophilic amination (Scheme 8b). To investigate these possibilities, we performed some control experiments. When the independently prepared simply reduced **4a** was subjected to reaction conditions including the copper catalyst, base, and **2a**, no aminated product **3aa** was observed with **4a** left intact (Scheme 9a). The additional use of (EtO)_3_SiH also gave no **3aa**, thus excluding the possibility demonstrated in Scheme 8a. The intermediacy of the ketene silyl acetal (Scheme 8b) was also examined by the following experiments; the copper-catalysed reaction of **1a** with (EtO)_3_SiH in the absence of **2a** (4 h) was followed by the addition of D_2_O to afford the deuterated **4a-d** in 99% yield with 82% D content (Scheme 9b).^38^ This result suggests *in situ* formation of the ketene silyl acetal. However, the quenching with the hydroxylamine **2a** instead of D_2_O only formed the protonated **4a** without any detectable amount of the aminated product **3aa** (Scheme 9c). Thus, the ketene silyl acetal can be generated under the optimal catalytic conditions but as nonproductive species, just en route to the protonated byproduct; under optimal conditions, the transmetalation from Si to Cu in the ketene silyl acetal might be unfavored.^39^ Actually, also under catalytic optimal conditions, the D_2_O quenching afforded the partial but a significant amount of deuterated **4a-d** along with the aminated product **3aa** (Scheme 9d). On the basis of the findings in Scheme 9, the originally proposed direct electrophilic amination of the firstly generated copper enolate with hydroxylamines is most favourable.

**Scheme 10:**
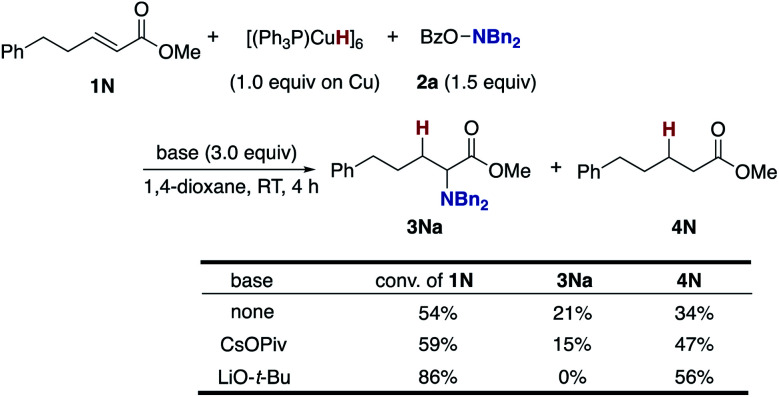
Effects of external bases under silane-free, stoichiometric conditions with [(Ph_3_P)CuH]_6_.

To gain insight into the origin of the positive effects of the CsOPiv base (Table 1, entries 5 *vs.* 10), we then investigated the amination/protonation selectivity under silane-free, stoichiometric conditions on CuH in the absence and presence of CsOPiv (Scheme 10); upon treatment of unsaturated ester **1N** with Stryker's reagent, [(PPh_3_)CuH]_6_,^40^ and the hydroxylamine **2a**, the corresponding α-amino acid derivative **3Na** was formed in 21% yield along with 34% of the simply reduced **4N**. The addition of CsOPiv was almost negligible, delivering **3Na** and **4N** in similar 15 and 47% yields, respectively. Thus, acceleration of the C–N bond forming step (**B** to **C** and **3** in Scheme 3) by the action of CsOPiv is unlikely. On the other hand, LiO-*t*-Bu totally shut down the formation of **3Na**, which is consistent with the optimization studies in entries 1 and 11 of Table 1.

**Scheme 11:**
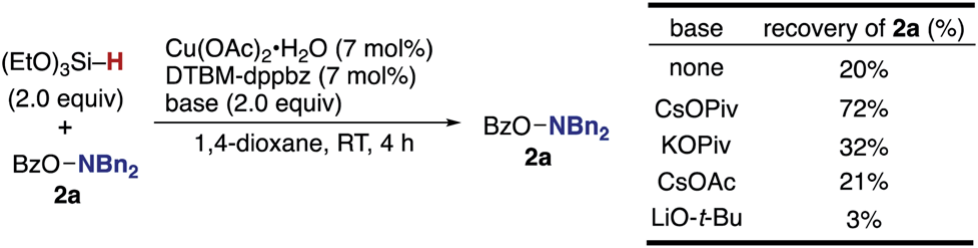
Effects of external bases in the decomposition of hydroxylamine **2a**.

The aforementioned results prompted us to check the dependence of the hydroxylamine decomposition side pathway (**A** and **2** to **2-H** in Scheme 3)^22*e*,*f*^ on the external base (Scheme 11). Under the external-base free conditions, 80% of the hydroxylamine **2a** was decomposed within 4 h. In sharp contrast, the rate of decomposition dramatically decreased in the presence of CsOPiv, and 72% of **2a** was retained after 4 h. These outcomes suggest that the CsOPiv base suppresses the competitive N–O bond cleavage by the CuH species to keep the concentration of the hydroxylamine higher and increase the amination product over the protonation byproduct.^41^ Other bases also suppressed the decomposition to some extent, except for strongly basic LiO-*t*-Bu, but with CsOPiv proving to be best.

## Conclusions

We have developed an umpolung-enabled copper-catalysed regioselective hydroamination of α,β-unsaturated esters with hydrosilanes and hydroxylamines to deliver the corresponding α-amino acid derivatives. The judicious choice of the CsOPiv external base and supporting ligand with remote steric bulkiness promotes the otherwise challenging C–N bond formation at the α position to the carbonyl. The asymmetric induction at the β-position is possible by using the suitable chiral Xyl-BINAP or DTBM-SEGPHOS bisphosphine ligand. Moreover, combined with the 8-phenylmenthol chiral auxiliary, the point chirality at the α-position can also be controlled, giving optically active unnatural α-amino acids with two adjacent stereocentres. Asymmetric copper catalysis is also applied to the conjugation of α-amino acids with biologically active complex amines. Some mechanistic experiments suggest the pivotal role of the copper enolate in the C–N forming step and the unique effect of CsOPiv to suppress the competitive but nonproductive decomposition pathway of the hydroxylamines. The obtained results can provide a new repertoire of C–N bond formation approaches to unnatural and complicated chiral α-amino acid derivatives. More detailed mechanistic studies and further development of related copper catalysis for more complicated and densely functionalized α-amino acids are ongoing in our laboratory and will be reported in due course.

## Data availability

All experimental procedures and spectroscopic data can be found in the ESI.†

## Author contributions

S. N. and K. H. conceived the idea. S. N. performed all experiments including condition optimizations and exploring the scope. K. H. supervised the project. M. M. supported other authors to perform the project well. All the authors discussed the results and commented on the manuscript.

## Conflicts of interest

There are no conflicts to declare.

## Supplementary Material

SC-012-D1SC03692K-s001
