# Supplementary material for: An umpolung-enabled copper-catalysed regioselective hydroamination approach to α-amino acids
Source: Chem Sci. 2021 Jul 27;12(34):11525–37. doi: 10.1039/d1sc03692k (PMC8409476; doi:10.1039/d1sc03692k)
Supplement: SC-012-D1SC03692K-s001 [file SC-012-D1SC03692K-s001.pdf]

Electronic Supplementary Material (ESI) for Chemical Science.
This journal is © The Royal Society of Chemistry 2021

Supporting Information

An Umpolung-Enabled Copper-Catalysed Regioselective Hydroamination Approach to α-Amino Acids

Soshi Nishino,† Masahiro Miura,‡ and Koji Hirano*,†

† *Department of Applied Chemistry, Graduate School of Engineering, Osaka University, Suita, Osaka 565-0871, Japan*

‡ *Innovative Catalysis Science Division, Institute for Open and Transdisciplinary Research Initiatives (ICS-OTRI), Osaka University, Suita, Osaka 565-0871, Japan*

E-mail: k_hirano@chem.eng.osaka-u.ac.jp (K.H.)

Contents

**Instrumentation and Chemicals**	S2–S3
**Experimental Procedures**	S4–S7
**Detailed Optimization Studies**	S8–S13
**Unsuccessful Substrates**	S14
**Stereochemical Assignment**	S15–S17
**Chiral HPLC Charts of Enantioenriched Products**	S18–S51
**Characterization Data for Products**	S52–S148
**References**	S149

Instrumentation and Chemicals

^{1}H, $^{13}C\{^{1}H\}$, $^{19}F\{^{1}H\}$, ^{11}B NMR spectra were recorded at 400, 100, 376, and 128 MHz respectively, for $CDCl_3$ or DMSO-d_6 solutions. HRMS data were obtained by APCI using TOF. GC analysis was carried out using a silicon OV-17 column (2.6 mm i.d. x 1.5 m) or a CBP-1 capillary column (0.5 mm i.d. x 25 m). TLC analyses were performed on commercial glass plates bearing a 0.25 mm layer of Merck silica gel $60F_{254}$. Silica gel (Wakosil C-200) was used for column chromatography. Gel permeation chromatography (GPC) was performed by LC-20AR (pump, SHIMADZU, 7.5 mL/min) and SPD-20A (UV detector, SHIMADZU, 254 nm) with two in-line YMC-GPC T2000 (20 x 600 mm, particle size: 10 μm) (preparative columns, YMC).

Unless otherwise noted, materials obtained from commercial suppliers were used as received. 1,4-Dioxane was dried on a Glass Contour Solvent dispensing system (Nikko Hansen & Co., Ltd.) prior to use. $Cu(OAc)_2$•H_2O was obtained from FUJIFILM Wako Pure Chemical Co. Optically active (*R*)- and (*S*)-Xyl-BINAP and (*R*)-DTBM-SEGPHOS ligands and $(EtO)_3SiH$ were available from TCI. CsOPiv (purchased from Aldrich) should be crushed to pieces with a mortar and a pestle in a glovebox filled with nitrogen and then dried at 100 ˚C under high vacuum overnight (note: this preactivation was essential for reproducibility). The acrylates **1a**–**j**, **l**, **n**–**r,** **B**, and **C** were prepared by the standard HWE reaction.[S1] The tryptophan derivative **1k**[S2] and chloro- or β,β-diaryl-substituted substrates (**1m**, **s**–**u**)[S3] were synthesized according to the literature. Dimethyl mesaconate (**1v**) was produced from the commercially available mesaconic acid under conditions of classical Fischer esterification.[S4] The β-boryl- and silyl-substituted acrylates **1w** and **1x** were obtained via conjugate borylation[S5] and silylation[S6] of the corresponding alkynoate, respectively. The methyl cinnamate (**1y**), methyl crotonate (**1z**), and methyl sorbate (**1A**) were commercial sources. The other alkyl and

aryl eaters **1D**–**M** were prepared by the condensation of the corresponding carboxylic acids and alcohols.[S7] *O*-Benzoyl-*N,N*-dibenzylhydroxylamine (**2a**) was obtained by the reaction of *N,N*-dibenzylhydroxylamine with benzoyl chloride, while other *O*-benzoyl-*N,N*-dialkylhydroxylamines **2** were synthesized through the nucleophilic substitution of the corresponding amines with benzoyl peroxide.[S8] DTBM-dppbz ligand was prepared by the reported method.[S9] All reactions were carried out under nitrogen atmosphere unless otherwise noted.

Experimental Procedures

Copper-Catalysed Regioselective Hydroamination of Acrylates

Synthesis of **3aa** (Table 1, entry 27, 0.25 mmol scale): $Cu(OAc)_2 \cdot H_2O$ (5.0 mg, 0.025 mmol), DTBM-dppbz (25.4 mg, 0.025 mmol), and CsOPiv (175.5 mg, 0.75 mmol) were placed in a 20 mL Schlenk tube, which was filled with nitrogen by using the Schlenk technique. 1,4-Dioxane (1.0 mL) was then added to the tube, and the suspension was stirred for 15 min at ambient temperature. $(EtO)_3Si$-H (123.2 mg, 0.75 mmol) was then added via a syringe, and the resulting solution was stirred at the same temperature. After 15 min, *O*-benzoyl-*N*,*N*-dibenzylhydroxylamine (**2a**, 79.3 mg, 0.25 mmol) was added in one portion, and (*E*)-*β*-methylcinnamate (**1a**, 88.1 mg, 0.50 mmol) was finally added dropwise. The reaction solution was stirred at room temperature for additional 4 h. The resulting mixture was directly filtered through a short pad of neutral alumina and Na_2SO_4. The filtrate was evaporated in vacuo and purified by silica gel column chromatography with hexane/ethyl acetate (20/1, v/v) and GPC ($CHCl_3$) to give methyl 2-(dibenzylamino)-3-phenylbutanoate (**3aa**, 85.8 mg, 0.23 mmol) in 92% yield with 42:58 *syn*/*anti* ratio.

Synthesis of **3aa** (Scheme 5, 1.0 mmol scale): $Cu(OAc)_2 \cdot H_2O$ (20.0 mg, 0.10 mmol), DTBM-dppbz (101.5 mg, 0.10 mmol), and CsOPiv (702.1 mg, 3.0 mmol) were placed in a two-necked 20 mL reaction flask, which was filled with nitrogen by using the Schlenk technique. 1,4-Dioxane (4.0 mL) was then added to the tube, and the suspension was stirred for 15 min at ambient temperature. $(EtO)_3Si$-H (492.8 mg, 3.0 mmol) was then added via a syringe, and the resulting solution was stirred at the same temperature. After 15 min, *O*-benzoyl-*N*,*N*-dibenzylhydroxylamine (**2a**, 317.4 mg, 1.0 mmol) was added in one portion, and (*E*)-*β*-methylcinnamate (**1a**, 352.4 mg, 2.0 mmol) was finally added dropwise. The reaction solution was stirred at room temperature for additional 4 h. The resulting mixture was directly filtered through a short pad of neutral alumina and Na_2SO_4. The filtrate was evaporated in vacuo and purified by silica gel column chromatography with hexane/ethyl acetate (20/1, v/v) and GPC ($CHCl_3$) to give methyl 2-(dibenzylamino)-3-phenylbutanoate (**3aa**, 272.3 mg, 0.73 mmol) in 73% yield with 42:58 *syn*/*anti* ratio.

Copper-Catalysed Regio- and Enantioselective Hydroamination of Acrylates

Synthesis of **3aa** (Table 2, 0.15 mmol scale): $Cu(OAc)_2 \cdot H_2O$ (30.0 mg, 0.015 mmol), (*R*)-Xyl-BINAP (11.0 mg, 0.015 mmol), and CsOPiv (105.3 mg, 0.45 mmol) were placed in a 20 mL Schlenk tube, which was filled with nitrogen by using the Schlenk technique. 1,4-Dioxane (0.6 mL) was then added

to the tube, and the suspension was stirred for 15 min at ambient temperature. $(EtO)_3Si$-H (73.9 mg, 0.45 mmol) was then added via a syringe, and the resulting solution was stirred at the same temperature. After 15 min, *O*-benzoyl-*N*,*N*-dibenzylhydroxylamine (**2a**, 47.6 mg, 0.15 mmol) was added in one portion, and (*E*)-*β*-methylcinnamate (**1a**, 52.9 mg, 0.30 mmol) was finally added dropwise. The reaction solution was stirred at room temperature for additional 18 h. The resulting mixture was directly filtered through a short pad of neutral alumina and Na_2SO_4. The filtrate was evaporated in vacuo and purified by silica gel column chromatography with hexane/ethyl acetate (20/1, v/v) and GPC ($CHCl_3$) to give methyl 2-(dibenzylamino)-3-phenylbutanoate (**3aa**, 45.3 mg, 0.12 mmol) in 81% yield with 43:57 *syn*/*anti* ratio. The enantiomeric ratio (e.r.) of each diastereomer was determined to be 97:3 by chiral HPLC analysis on a chiral stationary phase.

Synthesis of **3aa** (Scheme 6, 1.0 mmol scale): $Cu(OAc)_2 \cdot H_2O$ (20.0 mg, 0.10 mmol), (*R*)-Xyl-BINAP (73.5 mg, 0.10 mmol), and CsOPiv (702.1 mg, 3.0 mmol) were placed in a two-necked 20 mL reaction flask, which was filled with nitrogen by using the Schlenk technique. 1,4-Dioxane (4.0 mL) was then added to the tube, and the suspension was stirred for 15 min at ambient temperature. $(EtO)_3Si$-H (492.8 mg, 3.0 mmol) was then added via a syringe, and the resulting solution was stirred at the same temperature. After 15 min, *O*-benzoyl-*N*,*N*-dibenzylhydroxylamine (**2a**, 317.4 mg, 1.0 mmol) was added in one portion, and (*E*)-*β*-methylcinnamate (**1a**, 352.4 mg, 2.0 mmol) was finally added dropwise. The reaction solution was stirred at room temperature for additional 18 h. The resulting mixture was directly filtered through a short pad of neutral alumina and Na_2SO_4. The filtrate was evaporated in vacuo and purified by silica gel column chromatography with hexane/ethyl acetate (20/1, v/v) and GPC ($CHCl_3$) to give methyl 2-(dibenzylamino)-3-phenylbutanoate (**3aa**, 265.2 mg, 0.71 mmol) in 71% yield with 44:56 *syn*/*anti* ratio. The enantiomeric ratio (e.r.) of each diastereomer was determined to be 97:3 by chiral HPLC analysis on a chiral stationary phase.

Removal of Auxiliary (Scheme 7b)

(1*R*,2*S*,5*R*)-5-Methyl-2-(2-phenylpropan-2-yl)cyclohexyl 2-(dibenzylamino)-3-phenylbutanoate (**3Ga**, 55.9 mg, 0.097 mmol) was placed in a 20 mL Schlenk tube, which was filled with nitrogen by using the Schlenk technique. THF (1.5 mL) was then added to the tube, and the suspension was stirred for 5 min at 0 °C. $LiAlH_4$ (11.0 mg, 0.29 mmol) was then added in one portion at 0 °C. The reaction solution was stirred at 50 °C for additional 12 h. The resulting mixture was quenched with $Na_2SO_4 \cdot 10H_2O$ and sat. NH_4Cl aq. The mixture was extracted with ethyl acetate three times, and the combined

organic layer was dried over Na_2SO_4 and concentrated in vacuo. The residue was purified by silica gel column chromatography with hexane/ethyl acetate (10/1 → 5/1) to give (2*R*,3*R*)-2-(dibenzylamino)-3-phenylbutan-1-ol (***anti*-5**, 23.8 mg, 0.069 mmol) and (2*S*,3*R*)-2-(dibenzylamino)-3-phenylbutan-1-ol (***syn*-5**, 1.0 mg, 0.0030 mmol)in 71% and 3% yields, respectively.

Conversion of *anti*-5 into α-Amino Acid *anti*-6 (Scheme 7b)

A 20 mL two-necked reaction flask, equipped with a stir bar was charged with (2*R*,3*R*)-2-(dibenzylamino)-3-phenylbutan-1-ol (***anti*-5**, 23.8 mg, 0.069 mmol), $Pd(OH)_2$ on carbon (20 w%, 4.8 mg), and MeOH (1.0 mL). The flask was evacuated and backfilled with hydrogen (this process was repeated a total of 3 times), and the suspension was stirred at room temperature for 24 h under hydrogen atmosphere (1 atm, balloon). The reaction flask was then evacuated and backfilled with N_2. The resulting mixture was filtered through a pad of Celite, and then evaporated in vacuo to give (2*R*,3*R*)-2-amino-3-phenylbutan-1-ol (9.7 mg, 0.059 mmol) in 85% yield.

(2*R*,3*R*)-2-Amino-3-phenylbutan-1-ol (9.7 mg, 0.059 mmol) and $NaHCO_3$ (24.6 mg, 0.29 mmol) were placed in a 20 mL Schlenk tube, which was filled with nitrogen by using the Schlenk technique. THF (0.25 mL) and H_2O (0.25 mL) were then added to the tube, and the suspension was stirred for 5 min at 0 ^{o}C. $(Boc)_2O$ (12.8 mg, 0.059 mmol) was then added dropwise. The reaction solution was stirred at room temperature for 12 h. The resulting mixture was extracted with ethyl acetate three times, and the combined organic layer was dried over Na_2SO_4 and concentrated in vacuo to give *tert*-butyl ((2*R*,3*R*)-1-hydroxy-3-phenylbutan-2-yl)carbamate (14.3 mg, 0.054 mmol) in 92% yield.

tert-Butyl ((2*R*,3*R*)-1-hydroxy-3-phenylbutan-2-yl)carbamate (14.3 mg, 0.054 mmol), TEMPO (1.7 mg, 0.011 mmol), and $PhI(OAc)_2$ (38.1 mg, 0.12 mmol) were placed in a 20 mL Schlenk tube, which was filled with nitrogen by using the Schlenk technique. CH_3CN (0.40 mL) and H_2O (0.40 mL) were then added to the tube. The reaction solution was stirred at room temperature for 12 h. The resulting mixture was extracted with $CHCl_3$ three times, and the combined organic layer was dried over Na_2SO_4 and concentrated in vacuo. The residue was purified by silica gel column chromatography with hexane/ethyl acetate (1/1) to give (2*R*,3*R*)-2-((*tert*-butoxycarbonyl)amino)-3-phenylbutanoic acid (***anti*-6**, 12.2 mg, 0.044 mmol) in 81% yield. The enantiomeric ratio (e.r.) was determined to be >99:1 by chiral HPLC analysis on a chiral stationary phase.

Oxidation of 3wa (Scheme S1)

To a solution of methyl (2-(dibenzylamino)-3-(4,4,5,5-tetramethyl-1,3,2-dioxaborolan-2-yl)butanoate (**3wa**, 54.2 mg, 0.13 mmol, *syn*/*anti* = 95:5) in THF (1.0 mL) was added aq. NaOH (1.0 M, 1.0 mL) and aq. H_2O_2 (30%, 0.50 mL) in one portion, and the resulting mixture was stirred for 1 h under air. The reaction was quenched with sat. $Na_2S_2O_3$ aq. The mixture was extracted with ethyl acetate three times, and the combined organic layer was dried over Na_2SO_4 and concentrated in vacuo. The residue was purified by silica gel column chromatography with hexane/ethyl acetate (4/1) to give methyl (2*R**,3*R**)-2-(dibenzylamino)-3-hydroxybutanoate (***syn*-3wa-OH**, 34.7 mg, 0.11 mmol, *syn*/*anti* > 99:1) in 85% yield.

Oxidation of 3xa (Scheme S1)

Methyl 2-(dibenzylamino)-3-(dimethyl(phenyl)silyl)butanoate (**3xa**, 35.6 mg, 0.083 mmol, *syn*/*anti* = 89:11) and $Hg(OAc)_2$ (31.9 mg, 0.091 mmol) were placed in a 50 mL flask. AcOOH (9% in AcOH, 1.5 mL) was then added. The reaction solution was stirred at room temperature for 12 h under air. $Na_2S_2O_3$ (395.3 mg, 2.5 mmol) and Zn powder (163.5 mg, 2.5 mmol) were then added (Note: this reduction process was essential for the conversion of the *N*-oxide into the amine). The reaction solution was stirred at 30 °C for additional 6 h under air. The reaction was quenched with sat. $NaHCO_3$ aq. and The resulting mixture was extracted with $CHCl_3$ three times, and the combined organic layer was dried over Na_2SO_4 and concentrated in vacuo. The residue was purified by silica gel column chromatography with hexane/ethyl acetate (4/1) to give methyl (2*R**,3*R**)-2-(dibenzylamino)-3-hydroxybutanoate (***syn*-3xa-OH**, 14.7 mg, 0.047 mmol, *syn*/*anti* > 99:1) in 57% yield.

Hydrogenolysis of *syn*-3aa (Scheme S2)

A 20 mL two-necked reaction flask, equipped with a stir bar was charged with methyl (2*R*,3*S*)-2-(dibenzylamino)-3-phenylbutanoate (***syn*-3aa**, 29.1 mg, 0.078 mmol), $Pd(OH)_2$ on carbon (20 w%, 5.8 mg), and MeOH (1.0 mL). The flask was evacuated and backfilled with hydrogen (this process was repeated a total of 3 times), and the suspension was stirred at room temperature for 24 h under hydrogen atmosphere (1 atm, balloon). The reaction flask was then evacuated and backfilled with N_2. The resulting mixture was filtered through a pad of Celite, and then evaporated in vacuo to give methyl (2*R*,3*S*)-2-amino-3-phenylbutanoate (***syn*-3aa-NH$_2$**, 12.8 mg, 0.066 mmol) in 85% yield.

Detailed Optimization Studies

Table S1. Optimization Studies for Copper-Catalysed Regioselective Hydroamination of (E)-1a with 2a: Nonenantioselective Conditions 1[a]

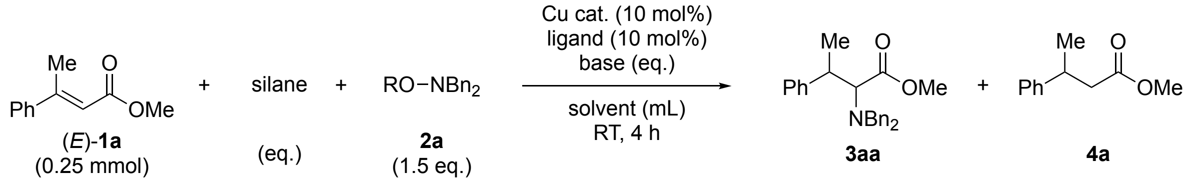

entry	silane (eq.)	2a	Cu cat.	ligand	base (eq.)	solvent (mL)	^{1}H NMR yield (%)[b]			
							**3aa** (*syn/anti*)	**4a**	**1a**	**2a**
1	PMHS (3.0)	**2a**	$Cu(OAc)_2$	DTBM-dppbz	none	1,4-dioxane (1.5)	22 (32:68)	55	0	0
2	PMHS (3.0)	**2a**	$Cu(OAc)_2$	DTBM-dppbz	LiO-*t*-Bu (3.0)	1,4-dioxane (1.5)	0	51	0	80
3	PMHS (3.0)	**2a**	$Cu(OAc)_2$	DTBM-dppbz	NaO-*t*-Bu (3.0)	1,4-dioxane (1.5)	0	49	0	78
4	PMHS (3.0)	**2a**	$Cu(OAc)_2$	DTBM-dppbz	CsF (3.0)	1,4-dioxane (1.5)	34 (44:56)	64	0	0
5	PMHS (3.0)	**2a**	$Cu(OAc)_2$	DTBM-dppbz	Cs_2CO_3 (3.0)	1,4-dioxane (1.5)	30 (37:63)	59	0	11
6	PMHS (3.0)	**2a**	$Cu(OAc)_2$	DTBM-dppbz	CsOAc (3.0)	1,4-dioxane (1.5)	62 (44:56)	43	0	9
7	PMHS (3.0)	**2a**	$Cu(OAc)_2$	DTBM-dppbz	KOAc (3.0)	1,4-dioxane (1.5)	38 (47:53)	57	0	0
8	PMHS (3.0)	**2a**	$Cu(OAc)_2$	DTBM-dppbz	NaOAc (3.0)	1,4-dioxane (1.5)	26 (50:50)	63	0	0
9	PMHS (3.0)	**2a**	$Cu(OAc)_2$	DTBM-dppbz	CsOAc (4.0)	1,4-dioxane (1.5)	65 (46:54)	36	0	23
10	PMHS (3.0)	**2a**	$Cu(OAc)_2$	DTBM-dppbz	CsOAc (2.0)	1,4-dioxane (1.5)	57 (40:60)	50	0	0
11	PMHS (3.0)	**2a**	$Cu(OAc)_2$	DTBM-dppbz	CsOAc (1.0)	1,4-dioxane (1.5)	34 (47:63)	71	0	0
12	$(EtO)_2MeSiH$ (3.0)	**2a**	$Cu(OAc)_2$	DTBM-dppbz	CsOAc (4.0)	1,4-dioxane (1.5)	67 (42:58)	36	0	48
13	$(EtO)_3SiH$ (3.0)	**2a**	$Cu(OAc)_2$	DTBM-dppbz	CsOAc (4.0)	1,4-dioxane (1.5)	71 (39:61)	33	0	48
14	$(HMe_2Si)_2O$ (1.5)	**2a**	$Cu(OAc)_2$	DTBM-dppbz	CsOAc (4.0)	1,4-dioxane (1.5)	64 (44:56)	35	0	31
15	Ph_2SiH_2 (3.0)	**2a**	$Cu(OAc)_2$	DTBM-dppbz	CsOAc (4.0)	1,4-dioxane (1.5)	0	90	0	98
16	PMHS (3.0)	**2a**	$Cu(OAc)_2$	DTBM-dppbz	CsOPiv (3.0)	1,4-dioxane (1.5)	71 (42:58)	29	0	51
17	PMHS (3.0)	**2a**	$Cu(OAc)_2$	DTBM-dppbz	KOPiv (3.0)	1,4-dioxane (1.5)	70 (44:56)	30	0	44
18	PMHS (3.0)	**2a**	$Cu(OAc)_2$	DTBM-dppbz	NaOPiv•H_2O (3.0)	1,4-dioxane (1.5)	0	0	98	149
19	PMHS (3.0)	**2a**	$Cu(OAc)_2$	DTBM-dppbz	CsOPiv (2.0)	1,4-dioxane (1.5)	51 (47:53)	31	0	9
20	PMHS (3.0)	**2a**	$Cu(OAc)_2$	DTBM-dppbz	CsOPiv (4.0)	1,4-dioxane (1.5)	62 (42:58)	41	0	66
21	$(EtO)_2MeSiH$ (3.0)	**2a**	$Cu(OAc)_2$	DTBM-dppbz	CsOPiv (3.0)	1,4-dioxane (1.5)	33 (39:61)	61	0	82
22	$(MeO)_2MeSiH$ (3.0)	**2a**	$Cu(OAc)_2$	DTBM-dppbz	CsOPiv (3.0)	1,4-dioxane (1.5)	0	98	0	86
23	$(TMSO)_2MeSiH$ (3.0)	**2a**	$Cu(OAc)_2$	DTBM-dppbz	CsOPiv (3.0)	1,4-dioxane (1.5)	0	30	71	99
24	$(EtO)_3SiH$ (3.0)	**2a**	$Cu(OAc)_2$	DTBM-dppbz	CsOPiv (3.0)	1,4-dioxane (1.5)	71 (38:62)	30	0	54
25	$(EtO)_3SiH$ (3.0)	**2a**	$Cu(OAc)_2$	DTBM-dppbz	KOPiv (3.0)	1,4-dioxane (1.5)	58 (40:60)	40	0	91
26	$(MeO)_3SiH$ (3.0)	**2a**	$Cu(OAc)_2$	DTBM-dppbz	CsOPiv (3.0)	1,4-dioxane (1.5)	0	99	0	110
27	$(HMe_2Si)_2O$ (1.5)	**2a**	$Cu(OAc)_2$	DTBM-dppbz	CsOPiv (3.0)	1,4-dioxane (1.5)	70 (41:59)	31	0	59
28	Et_3SiH (3.0)	**2a**	$Cu(OAc)_2$	DTBM-dppbz	CsOPiv (3.0)	1,4-dioxane (1.5)	0	0	99	151
29	Ph_2SiH_2 (3.0)	**2a**	$Cu(OAc)_2$	DTBM-dppbz	CsOPiv (3.0)	1,4-dioxane (1.5)	0	69	0	14

30	$(EtO)_3SiH$ (2.5)	**2a**	$Cu(OAc)_2$	DTBM-dppbz	CsOPiv (3.0)	1,4-dioxane (1.5)	63 (44:56)	38	0	92
31	$(EtO)_3SiH$ (2.0)	**2a**	$Cu(OAc)_2$	DTBM-dppbz	CsOPiv (3.0)	1,4-dioxane (1.5)	25 (40:60)	69	0	128
32	$(EtO)_3SiH$ (3.0)	**2a**	$Cu(OPiv)_2$	DTBM-dppbz	CsOPiv (3.0)	1,4-dioxane (1.5)	68 (40:60)	36	0	64
33	$(EtO)_3SiH$ (3.0)	**2a**	$Cu(OAc)_2$•H_2O	DTBM-dppbz	CsOPiv (3.0)	1,4-dioxane (1.5)	77 (42:58)	27	0	48
34	$(EtO)_3SiH$ (3.0)	**2a**	CuOAc	DTBM-dppbz	CsOPiv (3.0)	1,4-dioxane (1.5)	0	76	0	130
35	$(EtO)_3SiH$ (3.0)	**2a**	$Cu(OTf)_2$	DTBM-dppbz	CsOPiv (3.0)	1,4-dioxane (1.5)	72 (42:58)	30	0	62
36	$(EtO)_3SiH$ (3.0)	**2a**	CuCl	DTBM-dppbz	CsOPiv (3.0)	1,4-dioxane (1.5)	69 (42:58)	34	0	30
37	$(EtO)_3SiH$ (3.0)	**2a**	none	DTBM-dppbz	CsOPiv (3.0)	1,4-dioxane (1.5)	0	0	99	149
38	$(EtO)_3SiH$ (3.0)	**2a**	$Cu(OAc)_2$•H_2O	TMS-dppbz	CsOPiv (3.0)	1,4-dioxane (1.5)	26 (42:58)	65	0	105
39	$(EtO)_3SiH$ (3.0)	**2a**	$Cu(OAc)_2$•H_2O	*t*-Bu-dppbz	CsOPiv (3.0)	1,4-dioxane (1.5)	47 (38:62)	53	0	83
40	$(EtO)_3SiH$ (3.0)	**2a**	$Cu(OAc)_2$•H_2O	CF_3-dppbz	CsOPiv (3.0)	1,4-dioxane (1.5)	46 (41:59)	55	0	64
41	$(EtO)_3SiH$ (3.0)	**2a**	$Cu(OAc)_2$•H_2O	*p*-MeO-dppbz	CsOPiv (3.0)	1,4-dioxane (1.5)	21 (48:52)	80	0	104
42	$(EtO)_3SiH$ (3.0)	**2a**	$Cu(OAc)_2$•H_2O	*p*-*t*-Bu-dppbz	CsOPiv (3.0)	1,4-dioxane (1.5)	32 (41:59)	73	0	73
43	$(EtO)_3SiH$ (3.0)	**2a**	$Cu(OAc)_2$•H_2O	*p*-CF_3-dppbz	CsOPiv (3.0)	1,4-dioxane (1.5)	0	0	100	130
44	$(EtO)_3SiH$ (3.0)	**2a**	$Cu(OAc)_2$•H_2O	*o*-Me-dppbz	CsOPiv (3.0)	1,4-dioxane (1.5)	0	0	99	132
45	$(EtO)_3SiH$ (3.0)	**2a**	$Cu(OAc)_2$•H_2O	dppbz	CsOPiv (3.0)	1,4-dioxane (1.5)	10 (40:60)	67	19	108
46	$(EtO)_3SiH$ (3.0)	**2a**	$Cu(OAc)_2$•H_2O	*rac*-BINAP	CsOPiv (3.0)	1,4-dioxane (1.5)	8 (50:50)	25	62	112
47	$(EtO)_3SiH$ (3.0)	**2a**	$Cu(OAc)_2$•H_2O	DPEphos	CsOPiv (3.0)	1,4-dioxane (1.5)	0	45	54	119
48	$(EtO)_3SiH$ (3.0)	**2a**	$Cu(OAc)_2$•H_2O	Xantphos	CsOPiv (3.0)	1,4-dioxane (1.5)	0	52	48	135
49	$(EtO)_3SiH$ (3.0)	**2a**	$Cu(OAc)_2$•H_2O	dppe	CsOPiv (3.0)	1,4-dioxane (1.5)	0	0	99	119
50[c]	$(EtO)_3SiH$ (3.0)	**2a**	$Cu(OAc)_2$•H_2O	PPh_3	CsOPiv (3.0)	1,4-dioxane (1.5)	0	0	99	136
51[c]	$(EtO)_3SiH$ (3.0)	**2a**	$Cu(OAc)_2$•H_2O	DTBMP	CsOPiv (3.0)	1,4-dioxane (1.5)	0	0	99	143
52	$(EtO)_3SiH$ (3.0)	**2a**	$Cu(OAc)_2$•H_2O	IPr•HCl	CsOPiv (3.0)	1,4-dioxane (1.5)	0	94	0	94
53	$(EtO)_3SiH$ (3.0)	**2a**	$Cu(OAc)_2$•H_2O	none	CsOPiv (3.0)	1,4-dioxane (1.5)	0	0	99	139
54	$(EtO)_3SiH$ (3.0)	**2a-CF_3**	$Cu(OAc)_2$•H_2O	DTBM-dppbz	CsOPiv (3.0)	1,4-dioxane (1.5)	48 (44:56)	41	0	0
55	$(EtO)_3SiH$ (3.0)	**2a-OMe**	$Cu(OAc)_2$•H_2O	DTBM-dppbz	CsOPiv (3.0)	1,4-dioxane (1.5)	65 (42:58)	33	0	71
56	$(EtO)_3SiH$ (3.0)	**2a-NMe_2**	$Cu(OAc)_2$•H_2O	DTBM-dppbz	CsOPiv (3.0)	1,4-dioxane (1.5)	41 (49:51)	39	0	72
57	$(EtO)_3SiH$ (3.0)	**2a-Ac**	$Cu(OAc)_2$•H_2O	DTBM-dppbz	CsOPiv (3.0)	1,4-dioxane (1.5)	61 (44:56)	42	0	76
58	$(EtO)_3SiH$ (3.0)	**2a-Piv**	$Cu(OAc)_2$•H_2O	DTBM-dppbz	CsOPiv (3.0)	1,4-dioxane (1.5)	48 (48:52)	54	0	25
59	PMHS (3.0)	**2a-CF_3**	$Cu(OAc)_2$•H_2O	DTBM-dppbz	CsOPiv (3.0)	1,4-dioxane (1.5)	73 (41:59)	27	0	0
60	PMHS (3.0)	**2a-OMe**	$Cu(OAc)_2$•H_2O	DTBM-dppbz	CsOPiv (3.0)	1,4-dioxane (1.5)	73 (44:56)	30	0	61
61	PMHS (3.0)	**2a-NMe_2**	$Cu(OAc)_2$•H_2O	DTBM-dppbz	CsOPiv (3.0)	1,4-dioxane (1.5)	75 (47:53)	28	0	72
62	PMHS (3.0)	**2a-Ac**	$Cu(OAc)_2$•H_2O	DTBM-dppbz	CsOPiv (3.0)	1,4-dioxane (1.5)	76 (45:55)	25	0	23
63	PMHS (3.0)	**2a-Piv**	$Cu(OAc)_2$•H_2O	DTBM-dppbz	CsOPiv (3.0)	1,4-dioxane (1.5)	57 (37:63)	43	0	50
64	$(EtO)_3SiH$ (3.0)	**2a**	$Cu(OAc)_2$•H_2O	DTBM-dppbz	CsOPiv (3.0)	THF (1.5)	0	84	0	46
65	$(EtO)_3SiH$ (3.0)	**2a**	$Cu(OAc)_2$•H_2O	DTBM-dppbz	CsOPiv (3.0)	CPME (1.5)	0	83	0	49
66	$(EtO)_3SiH$ (3.0)	**2a**	$Cu(OAc)_2$•H_2O	DTBM-dppbz	CsOPiv (3.0)	Et_2O (1.5)	0	88	0	33
67	$(EtO)_3SiH$ (3.0)	**2a**	$Cu(OAc)_2$•H_2O	DTBM-dppbz	CsOPiv (3.0)	DCE (1.5)	42 (57:43)	60	0	81
68	$(EtO)_3SiH$ (3.0)	**2a**	$Cu(OAc)_2$•H_2O	DTBM-dppbz	CsOPiv (3.0)	DMF (1.5)	0	64	34	22
69	$(EtO)_3SiH$ (3.0)	**2a**	$Cu(OAc)_2$•H_2O	DTBM-dppbz	CsOPiv (3.0)	toluene (1.5)	0	91	0	22
70	$(EtO)_3SiH$ (3.0)	**2a**	$Cu(OAc)_2$•H_2O	DTBM-dppbz	CsOPiv (3.0)	cyclohexane (1.5)	0	79	0	19

71	$(EtO)_3SiH$ (3.0)	**2a**	$Cu(OAc)_2•H_2O$	DTBM-dppbz	CsOPiv (3.0)	1,4-dioxane (2.5)	43 (40:60)	52	0	76
72	***$(EtO)_3SiH$ (3.0)***	***2a***	***$Cu(OAc)_2•H_2O$***	***DTBM-dppbz***	***CsOPiv (3.0)***	***1,4-dioxane (1.0)***	***83 (45:55)***	***19***	***0***	***35***
73	$(EtO)_3SiH$ (3.0)	**2a**	$Cu(OAc)_2•H_2O$	DTBM-dppbz	CsOPiv (3.0)	1,4-dioxane (0.5)	82 (44:56)	19	0	21
74[d]	$(EtO)_3SiH$ (3.0)	**2a**	$Cu(OAc)_2•H_2O$	DTBM-dppbz	CsOPiv (3.0)	1,4-dioxane (1.0)	63 (43:57)	38	0	65
75[e]	$(EtO)_3SiH$ (3.0)	**2a**	$Cu(OAc)_2•H_2O$	DTBM-dppbz	CsOPiv (3.0)	1,4-dioxane (1.0)	48 (40:60)	35	0	14
76	$(EtO)_3SiH$ (4.0)	**2a**	$Cu(OAc)_2•H_2O$	DTBM-dppbz	CsOPiv (3.0)	1,4-dioxane (1.0)	43 (44:56)	60	0	56

[a] Reaction conditions: Cu cat. (0.025 mmol), ligand (0.025 mmol), (*E*)-**1a** (0.25 mmol), **2a** (0.38 mmol), silane (amount is based on Si–H), base, solvent, RT, 4 h, N_2. [b] Estimated by ^{1}H NMR based on 0.25 mmol with CH_2Br_2 as the internal standard. The *syn/anti* ratio was determined in the crude mixture. [c] With 20 mol % of ligand. [d] At 15 ˚C. [e] At 50 ˚C.

• ancillary ligands

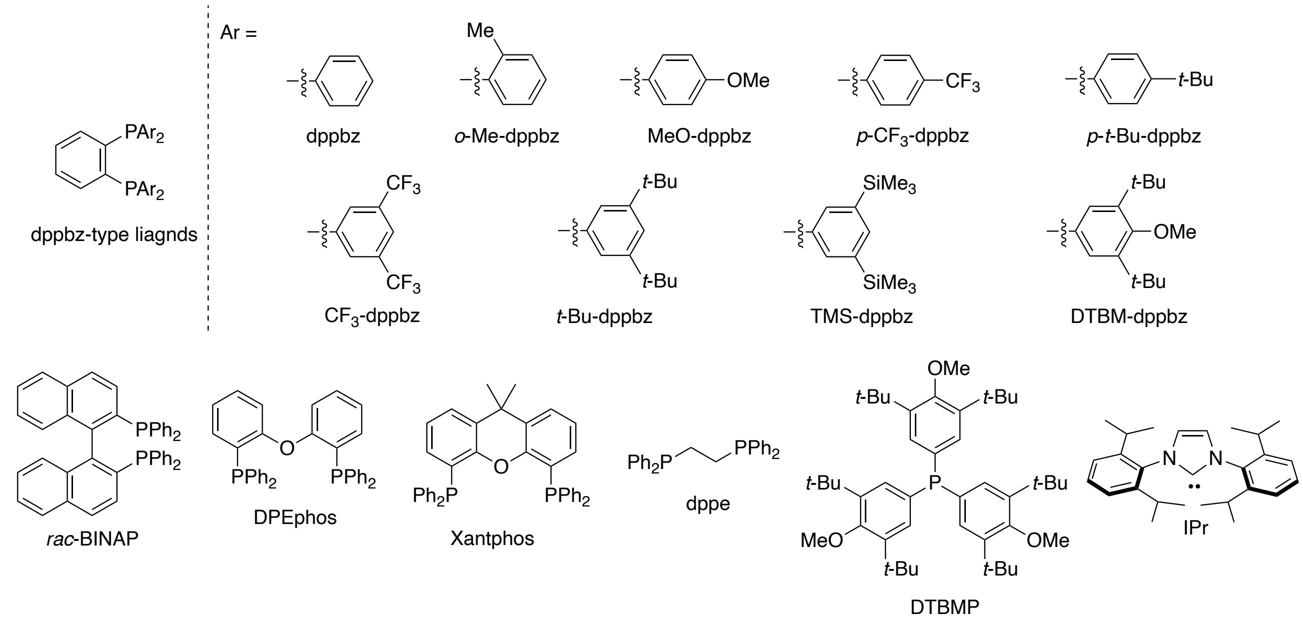

• amination reagents

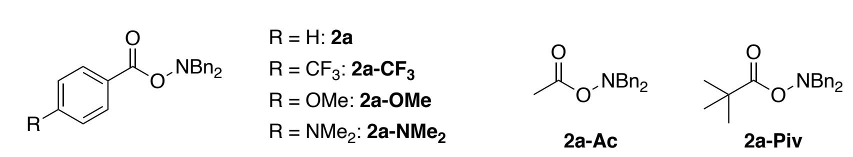

Table S2. Optimization Studies for Copper-Catalysed Regioselective Hydroamination of (E)-1a with 2a: Nonenantioselective Conditions 2[a]

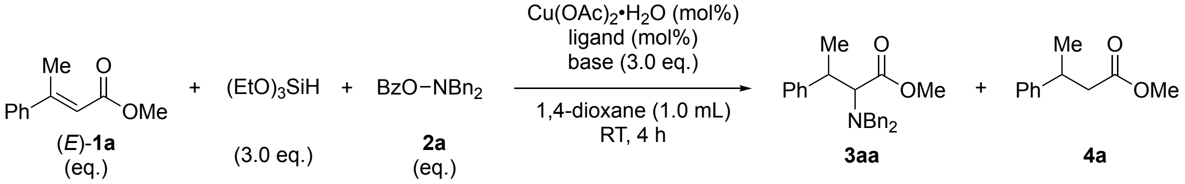

entry	(*E*)-**1a** (eq.)	**2a** (eq.)	$Cu(OAc)_2•H_2O$ (mol %)	ligand (mol %)	base	^{1}H NMR yield (%)[b]			
						**3aa** (*syn/anti*)	**4a**	**1a**	**2a**
1	(*E*)-**1a** (1.0)	**2a** (1.5)	$Cu(OAc)_2•H_2O$ (10 mol %)	DTBM-dppbz (10 mol %)	CsOPiv	82 (44:56)	19	0	21
2	(*E*)-**1a** (1.0)	**2a** (1.2)	$Cu(OAc)_2•H_2O$ (10 mol %)	DTBM-dppbz (10 mol %)	CsOPiv	64 (41:59)	37	0	43
3	(*E*)-**1a** (1.0)	**2a** (1.7)	$Cu(OAc)_2•H_2O$ (10 mol %)	DTBM-dppbz (10 mol %)	CsOPiv	74 (42:58)	30	0	68
4	(*E*)-**1a** (1.0)	**2a** (2.0)	$Cu(OAc)_2•H_2O$ (10 mol %)	DTBM-dppbz (10 mol %)	CsOPiv	59 (46:54)	44	0	105
5	(*E*)-**1a** (1.0)	**2a** (1.5)	$Cu(OAc)_2•H_2O$ (10 mol %)	DTBM-dppbz (20 mol %)	CsOPiv	71 (42:58)	30	0	19
6	(*E*)-**1a** (1.0)	**2a** (1.5)	$Cu(OAc)_2•H_2O$ (10 mol %)	DTBM-dppbz (12 mol %)	CsOPiv	0	60	0	112
7	(*E*)-**1a** (1.0)	**2a** (1.5)	$Cu(OAc)_2•H_2O$ (10 mol %)	DTBM-dppbz (10 mol %)	CsOPiv	80 (41:59)	21	0	36
8	(*E*)-**1a** (1.5)	**2a** (1.0)	$Cu(OAc)_2•H_2O$ (10 mol %)	DTBM-dppbz (10 mol %)	CsOPiv	92 (37:63)	51	0	0
9	***(E)-1a (2.0)***	***2a (1.0)***	***$Cu(OAc)_2•H_2O$ (10 mol %)***	***DTBM-dppbz (10 mol %)***	***CsOPiv***	***99 (38:62)***<br>***92 (42:58)[c]***	***84***	***0***	***0***
10	(*E*)-**1a** (2.0)	**2a** (1.0)	$Cu(OAc)_2•H_2O$ (10 mol %)	DTBM-dppbz (10 mol %)	LiO-*t*-Bu	0	67	63	0
11	(*E*)-**1a** (2.0)	**2a** (1.0)	$Cu(OAc)_2•H_2O$ (10 mol %)	dppbz (10 mol %)	CsOPiv	16 (44:56)	116	36	81

[a] Reaction conditions: $Cu(OAc)_2•H_2O$, ligand, (*E*)-**1a**, **2a**, $(EtO)_3SiH$ (0.75 mmol), base (0.75 mmol), 1,4-dioxane (1.0 mL), RT, 4 h, N_2. [b] Estimated by ^{1}H NMR based on 0.25 mmol with CH_2Br_2 as the internal standard. The *syn/anti* ratio was determined in the crude mixture. [c] Yield and *syn/anti* ratio after isolation.

Table S3. Optimization Studies for Copper-Catalysed Regio- and Enantioselective Hydroamination of (E)-1a with 2a[a]

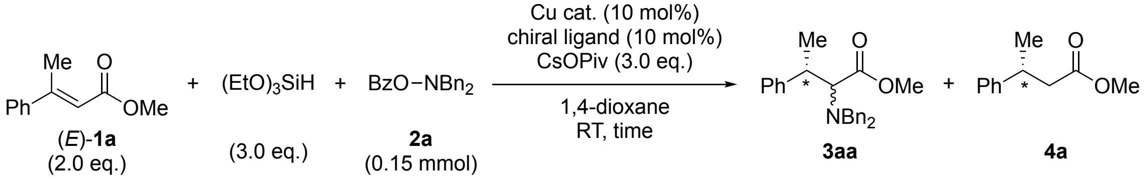

entry	Cu cat.	chiral ligand	time (h)	NMR yield (%)[b]				*syn/anti*[c]	er[d]	
				**3aa**	**4a**	**1a**	**2a**		*syn*	*anti*
1	$Cu(OAc)_2$•H_2O	(*R*)-DTBM-SEGPHOS	4	40	42	114	54	42:58	-	-
2	$Cu(OAc)_2$•H_2O	(*R*)-DTBM-SEGPHOS	18	67 (60)	63	70	20	42:56	99:1	99:1
3	CuCl	(*R*)-DTBM-SEGPHOS	4	50 (41)	69	76	39	41:59	98:2	98:2
4	CuCl	(*R*)-DTBM-SEGPHOS	18	81 (73)	94	23	0	42:58	96:4	96:4
5[e]	CuCl	(*R*)-DTBM-SEGPHOS	18	23	137	38	65	47:53	-	-
6	$Cu(OAc)_2$	(*R*)-DTBM-SEGPHOS	18	0	0	199	99	-	-	-
7	$Cu(OTf)_2$	(*R*)-DTBM-SEGPHOS	18	21	59	117	75	48:52	-	-
8	CuCl	(*R*)-DM-SEGPHOS	4	13 (3)	131	0	76	41:59	98:2	98:2
9	$Cu(OAc)_2$•H_2O	(*R*)-DM-SEGPHOS	18	55 (42)	144	1	10	44:56	98:2	98:2
10	CuCl	(*R*)-SEGPHOS	4	13	54	63	64	44:56	-	-
11	$Cu(OAc)_2$•H_2O	(*R*)-SEGPHOS	18	18	114	68	43	44:56	-	-
12	CuCl	(*R*)-DTBM-BINAP	4	74 (67)	57	37	10	44:56	95:5	95:5
13	CuCl	(*R*)-DTBM-BINAP	18	89 (82)	102	9	0	44:56	95:5	95:5
14[e]	CuCl	(*R*)-DTBM-BINAP	18	36	66	101	56	46:54	-	-
15	$Cu(OAc)_2$•H_2O	(*R*)-DTBM-BINAP	18	0	0	199	99	-	-	-
16	CuCl	(*R*)-Xyl-BINAP	4	74 (65)	112	0	17	43:57	97:3	97:3
17	***$Cu(OAc)_2$•H_2O***	***(R)-Xyl-BINAP***	***18***	***87 (81)***	***104***	***8***	***0***	***43:57***	***97:3***	***97:3***
18[f]	$Cu(OAc)_2$•H_2O	(*R*)-Xyl-BINAP	18	0	80	114	34	-	-	-
19	CuCl	(*R*)-BINAP	4	42 (30)	52	56	42	44:56	94:6	94:6
20	$Cu(OAc)_2$•H_2O	(*R*)-BINAP	18	33 (23)	74	53	11	44:56	94:6	94:6
21	CuCl	(*S*)-Tol-BINAP	4	22 (9)	63	61	65	42:58	5:95	5:95
22	CuCl	(*R*)-H_8-BINAP	4	38 (27)	58	51	42	42:58	99:1	99:1
23	CuCl	(*R*)-Difluorphos	4	11	44	91	75	46:54	-	-
24	CuCl	(*R*)-DTBM-MeO-BIPHEP	4	64 (57)	40	80	35	44:56	96:4	96:4
25	CuCl	(*R*)-DTBM-MeO-BIPHEP	18	61	98	40	24	44:56	-	-
26	$Cu(OAc)_2$•H_2O	(*R*)-DTBM-MeO-BIPHEP	18	0	0	200	100	-	-	-

27	CuCl	(*R*)-MeO-BIPHEP	4	30 (19)	98	67	60	43:57	96:4	96:4
28	$Cu(OAc)_2{\bullet}H_2O$	(*Sp,S'p*)-DMMeO-Mandyphos	4	14 (8)	33	151	64	44:56	72:28	72:28
29	$Cu(OAc)_2{\bullet}H_2O$	(*S,S*)-Xyl-BDPP	4	13 (5)	105	49	87	44:56	82:18	82:18
30	$Cu(OAc)_2{\bullet}H_2O$	(*R,R*)-Ph-BPE	4	0	47	128	86	-	-	-
31	$Cu(OAc)_2{\bullet}H_2O$	(*S,S*)-Me-Duphos	4	0	89	103	88	-	-	-
32	$Cu(OAc)_2{\bullet}H_2O$	(*R,R*)-BenzP*	4	0	150	29	99	-	-	-
33	$Cu(OAc)_2{\bullet}H_2O$	(*R,R*)-QuinoxP*	4	0	130	61	99	-	-	-

[a] Reaction conditions: Cu cat (0.015 mmol), ligand (0.015 mmol), (*E*)-**1a** (0.30 mmol), **2a** (0.15 mmol), $(EtO)_3SiH$ (0.45 mmol), CsOPiv (0.45 mmol), 1,4-dioxane (1.0 mL), RT, N_2. [b] Estimated by ^1H NMR based on 0.15 mmol with CH_2Br_2 as the internal standard. Isolated yields are given in parentheses. [c] The *syn/anti* ratio was determined in the crude mixture. [d] The enantiomeric ratios (er) were determined by HPLC analysis on a chiral stationary phase. [e] With PMHS instead of $(EtO)_3SiH$. [f] With LiO-*t*-Bu instead of CsOPiv.

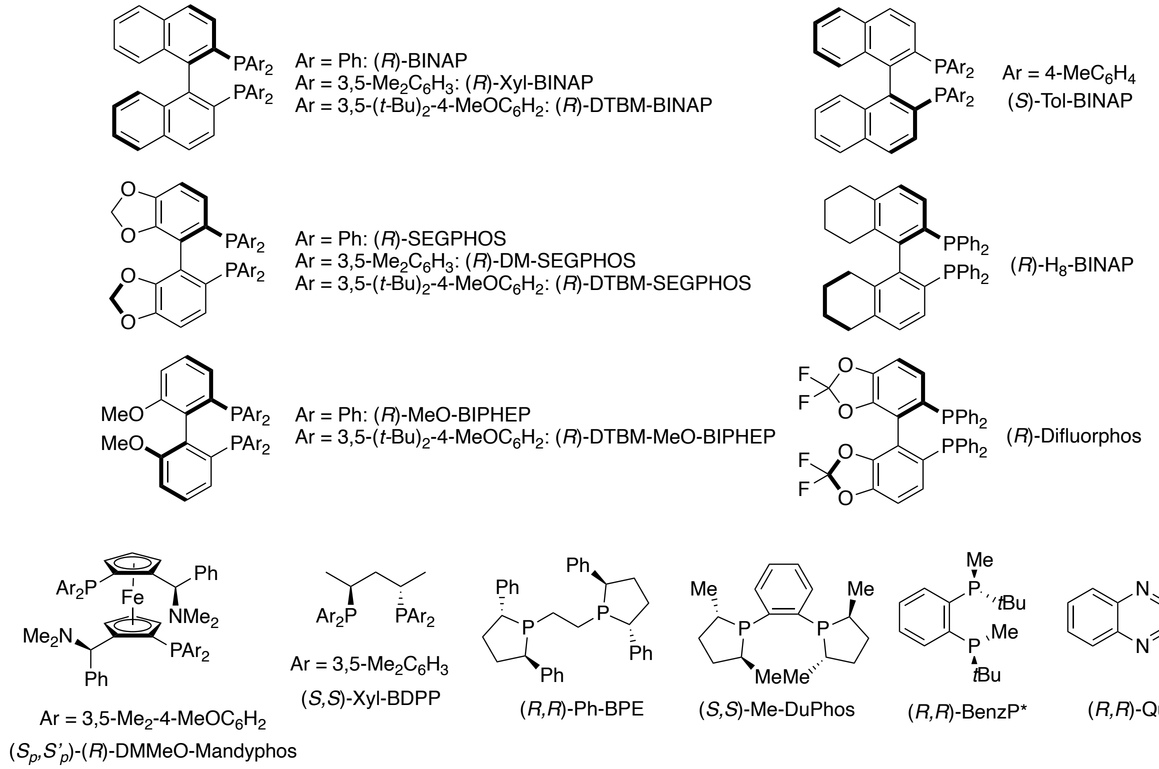

Unsuccessful Substrates

• limitation of α,β-unsaturated carbonyls

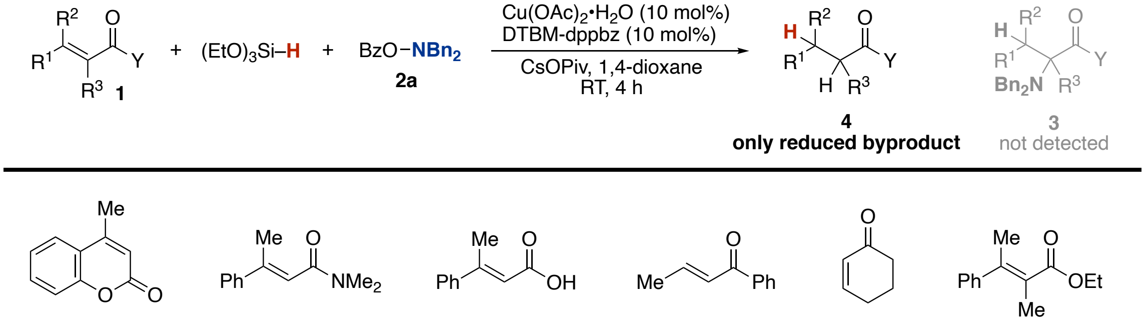

• limitation of hydroxylamine derivatives

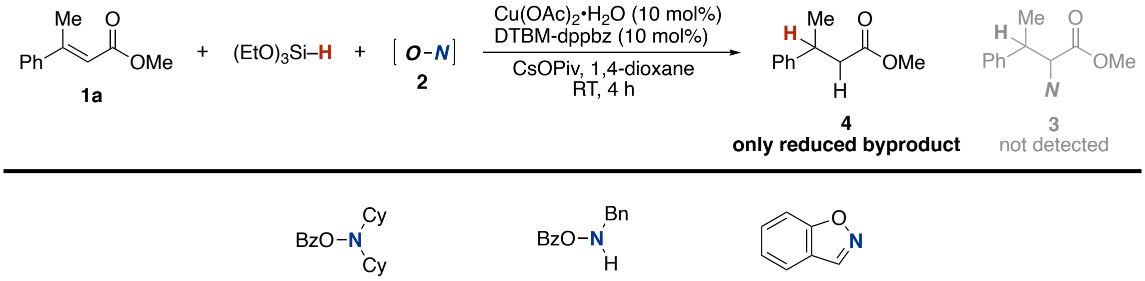

Stereochemical Assignment

Assignment of Relative Stereochemistry of 3wa and 3xa (Scheme 5)

The relative stereochemistry of major isomer of **3wa** and **3xa** (Scheme 5) was determined by comparison of ^{1}H NMR with the reported values[S10] after the oxidation (Scheme S1; see S7 for the experimental details).

Scheme S1. Oxidation and Determination of Relative Stereochemistry of 3wa and 3xa.

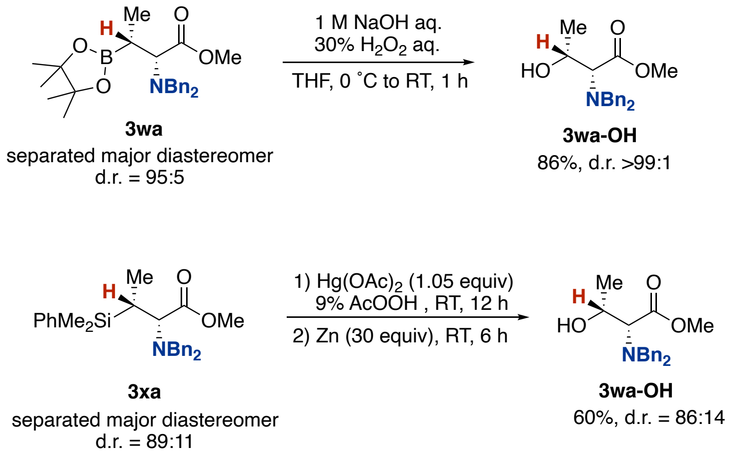

Assignment of Relative and Absolute Configuration of 3aa (Table 2, entry 6)

The relative and absolute configurations of **3aa** (Table 2, entry 6) were determined by comparison of ^{1}H NMR and specific rotation with the reported values[S11] after the chromatographic separation and hydrogenation (Scheme S2; see S7 for the experimental details). The stereochemistry of other products is basically assigned by analogy.

Scheme S2. Separation, Derivatization, and Determination of Relative/Absolute Configuration of 3aa.

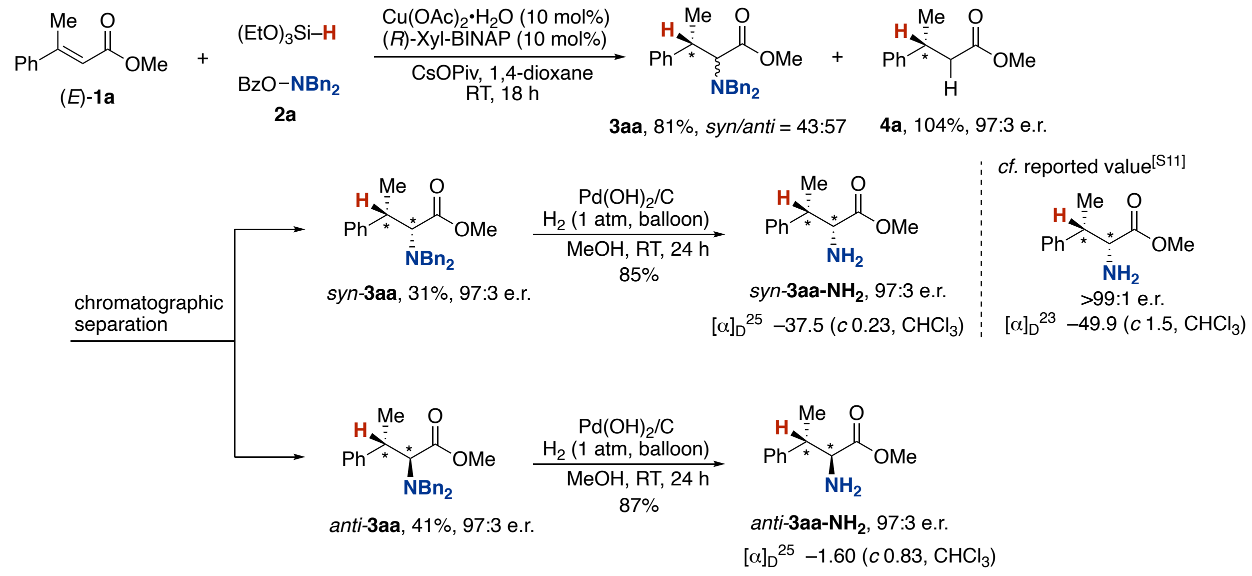

Assignment of Relative/Absolute Configuration and Enantiopurity of 3Ga and 6 (Scheme 7b)

The relative stereochemistry of *anti*-**6** was determined by the comparison of ^{1}H NMR data with the reported value.[S12] The enantiomeric purity was confirmed by chiral HPLC analysis with the authentic samples (Scheme S3).

Scheme S3. Preparation and HPLC Analysis of 6. HPLC Conditions: CHIRAL ART Amylose-SA (3 μm) column, 94/6 (hexane + 0.1vol% TFA)/isopropyl alcohol, 0.3 mL/min, UV detection at 210 nm.

a) authentic sample of diastereo- and enantiomixture *rac*-**6**

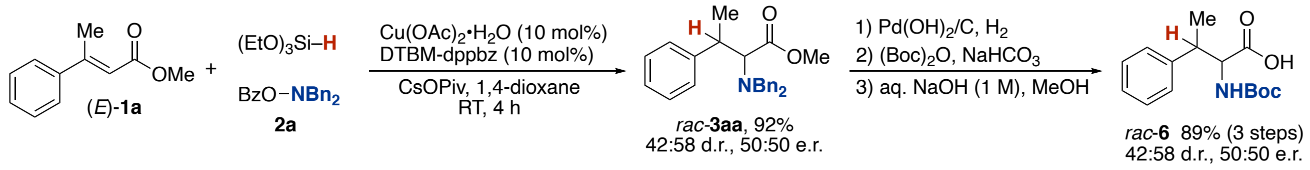

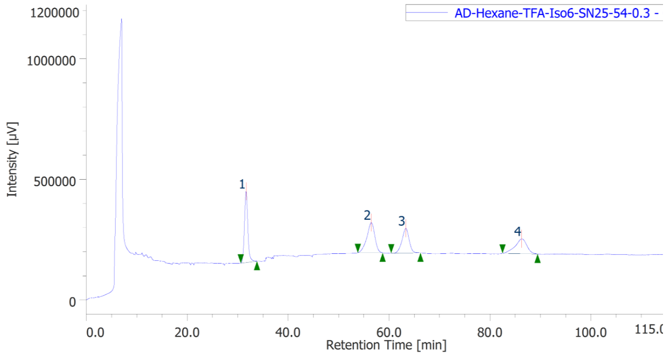

Peak #	Ret. Time	Area	Area %
1	31.647	13775621	29.02
2	56.457	13761428	28.99
3	63.303	9954533	20.97
4	86.273	9978369	21.02

b) authentic sample of (2*S*,3*S*)-*anti*-**6**

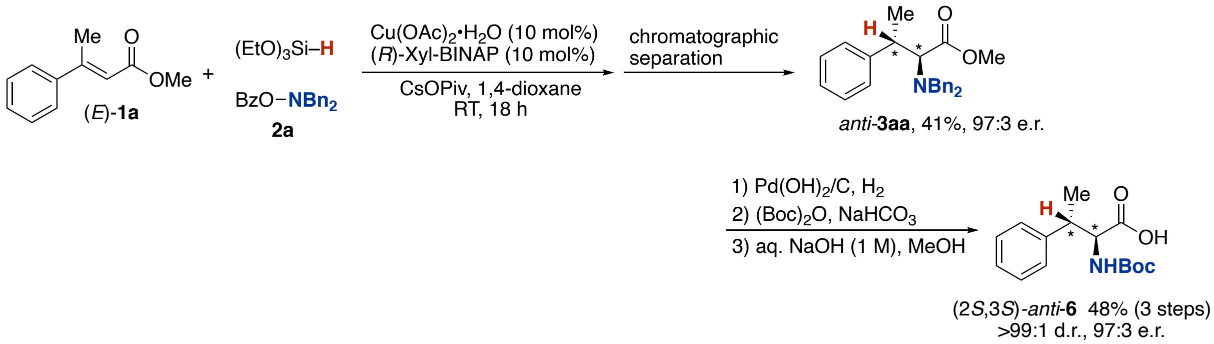

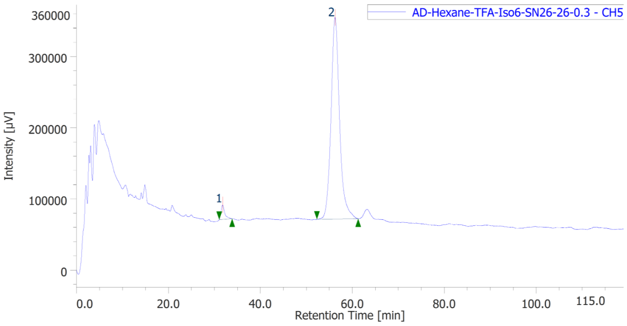

Peak #	Ret. Time	Area	Area %
1	31.763	1132254	3.00
2	56.223	36668794	97.00

c) sample from **3Ga** [(2*R*,3*R*)-*anti*-**6**]

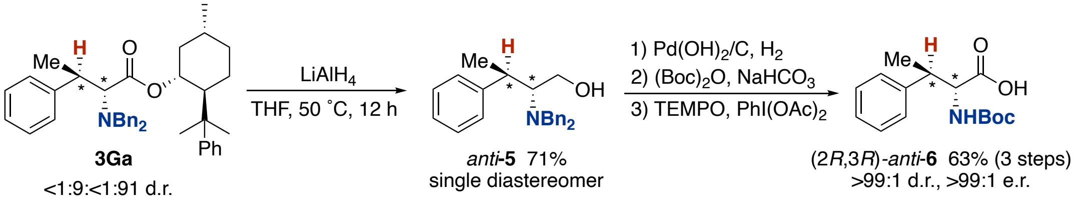

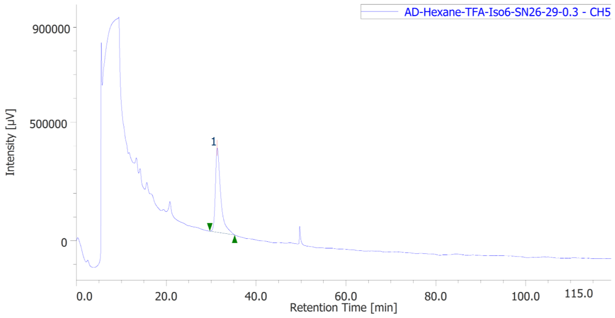

Peak #	Ret. Time	Area	Area %
1	31.340	29344029	100.00

Chiral HPLC Charts of Enantioenriched Products

3aa: The enantiomeric ratio was determined by HPLC analysis in comparison with authentic racemic material (CHIRALPAK AD-H column, 98.5/1.5 hexane/isopropyl alcohol, 0.5 mL/min, major isomers: t_R = 10.7, 24.5 min, minor isomers: t_R = 12.8, 14.3 min, UV detection at 210 nm, 30 °C).

rac-**3aa**

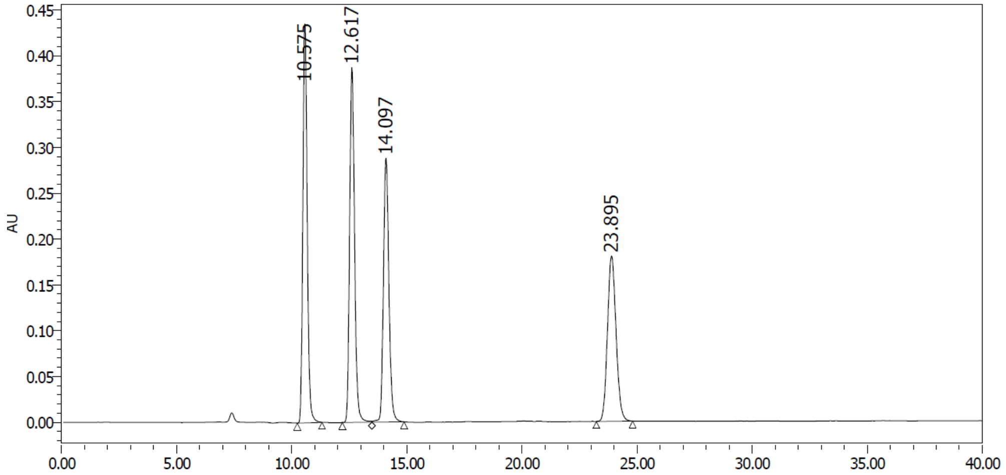

Peak #	Ret. Time	Area	Area %
1	10.575	5713190	27.34
2	12.617	5761699	27.57
3	14.097	4738699	22.68
4	23.895	4682657	22.41

chiral-**3aa**

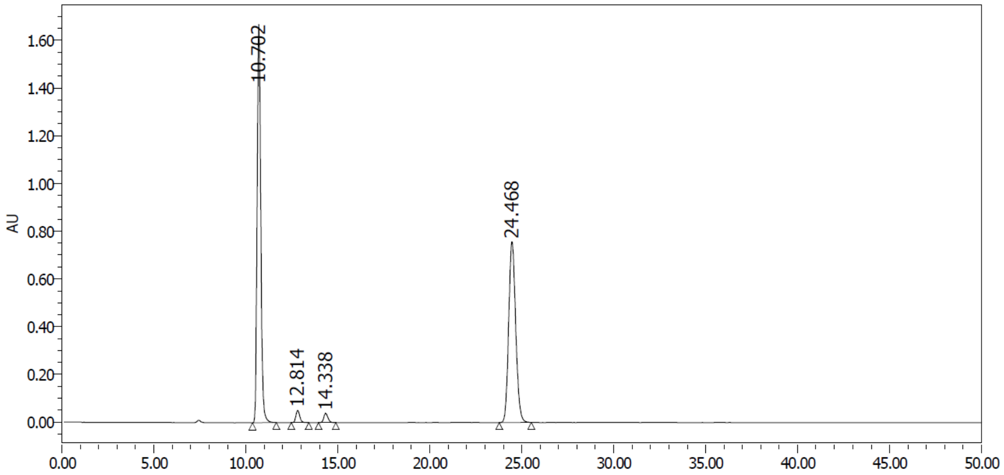

Peak #	Ret. Time	Area	Area %
1	10.702	23106160	51.81
2	12.814	770503	1.73
3	14.338	631510	1.42
4	24.468	20085456	45.04

***syn*-3aa**: The enantiomeric ratio was determined by HPLC analysis in comparison with authentic racemic material (CHIRALPAK AD-H column, 98.5/1.5 hexane/isopropyl alcohol, 0.5 mL/min, major isomer: t_R = 23.3 min, minor isomer: t_R = 13.9 min, UV detection at 210 nm, 30 ºC).

chiral-***syn*-3aa**

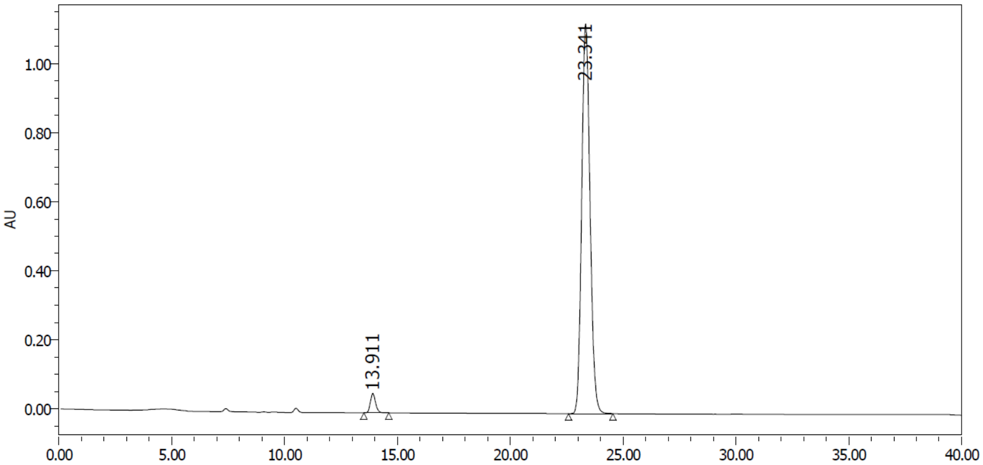

Peak #	Ret. Time	Area	Area %
1	13.911	901596	3.02
2	23.341	28999277	96.98

***anti*-3aa**: The enantiomeric ratio was determined by HPLC analysis in comparison with authentic racemic material (CHIRALPAK AD-H column, 98.5/1.5 hexane/isopropyl alcohol, 0.5 mL/min, major isomer: t_R = 10.6 min, minor isomer: t_R = 12.7 min, UV detection at 210 nm, 30 ºC).

chiral-***anti*-3aa**

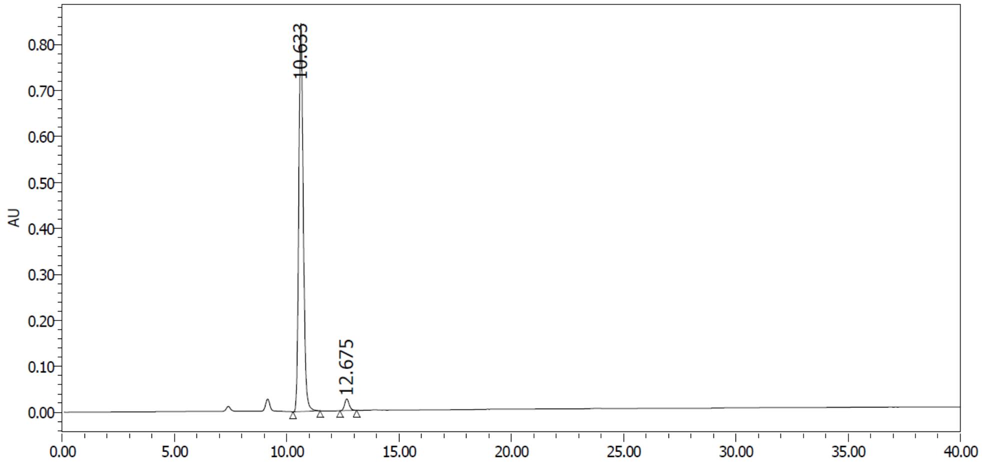

Peak #	Ret. Time	Area	Area %
1	10.633	11798376	97.07
2	12.675	356406	2.93

3ba: The enantiomeric ratio was determined by HPLC analysis in comparison with authentic racemic material (CHIRALPAK AD-H column, 99/1 hexane/isopropyl alcohol, 0.5 mL/min, major isomers: t_R = 11.7, 26.0 min, minor isomers: t_R = 13.4, 15.8 min, UV detection at 210 nm, 30 °C).

rac-**3ba**

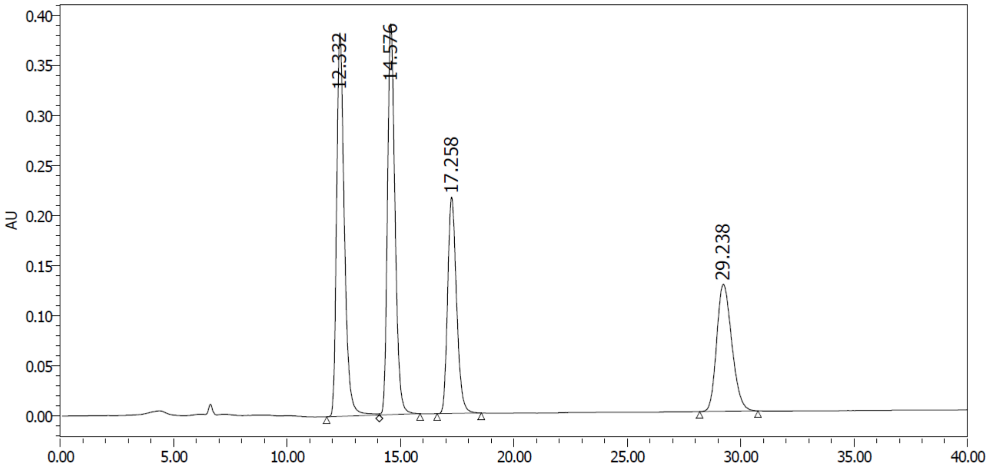

Peak #	Ret. Time	Area	Area %
1	12.332	9104746	30.47
2	14.576	9009963	30.15
3	17.258	5889464	19.71
4	29.238	5878427	19.67

chiral-**3ba**

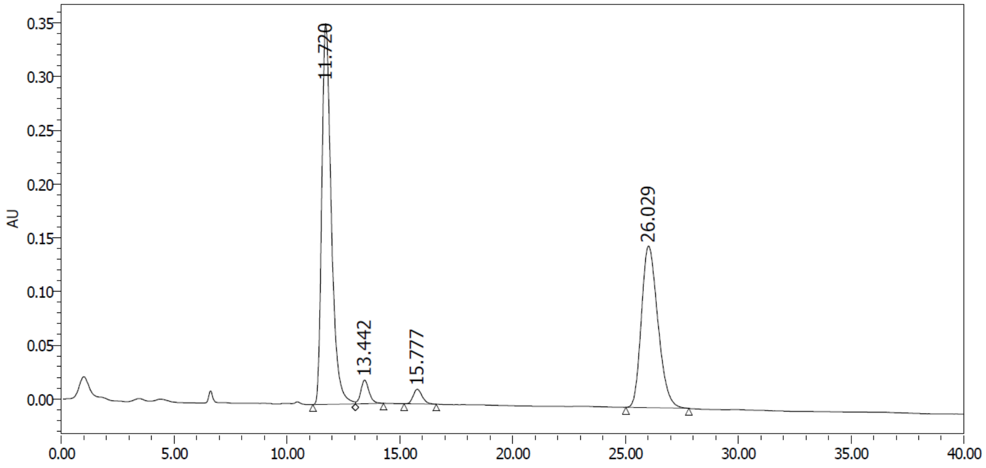

Peak #	Ret. Time	Area	Area %
1	11.720	10394325	54.77
2	13.442	556694	2.93
3	15.777	378885	2.00
4	26.029	7649318	40.30

3ca: The enantiomeric ratio was determined by HPLC analysis in comparison with authentic racemic material (CHIRALPAK AD-H column, 99/1 hexane/isopropyl alcohol, 0.5 mL/min, major isomers: t_R = 8.5, 17.8 min, minor isomers: t_R = 9.8, 11.6 min, UV detection at 210 nm, 30 °C).

rac-**3ca**

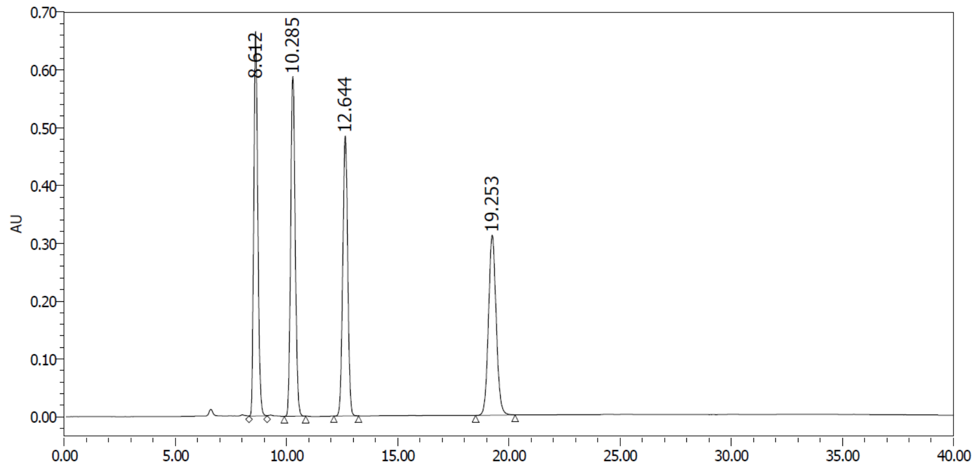

Peak #	Ret. Time	Area	Area %
1	8.612	8357633	26.34
2	10.285	8365412	26.36
3	12.644	7508094	23.66
4	19.253	7497351	23.63

chiral-**3ca**

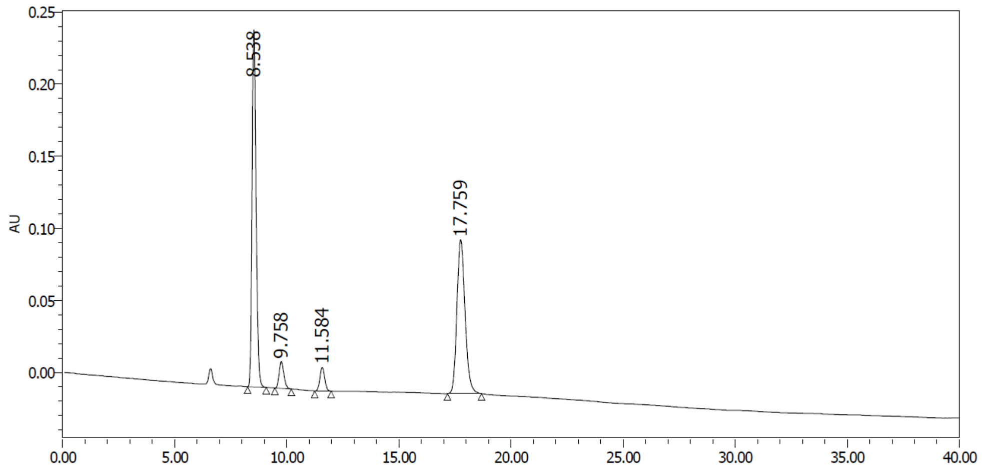

Peak #	Ret. Time	Area	Area %
1	8.538	3095568	49.16
2	9.758	274054	4.35
3	11.584	243065	3.86
4	17.759	2684091	42.63

3da: The enantiomeric ratio was determined by HPLC analysis in comparison with authentic racemic material (CHIRALPAK AD-H column, 99/1 hexane/isopropyl alcohol, 0.5 mL/min, major isomers: t_R = 9.0, 15.8 min, minor isomers: t_R = 10.6, 11.4 min, UV detection at 210 nm, 30 °C).

rac-**3da**

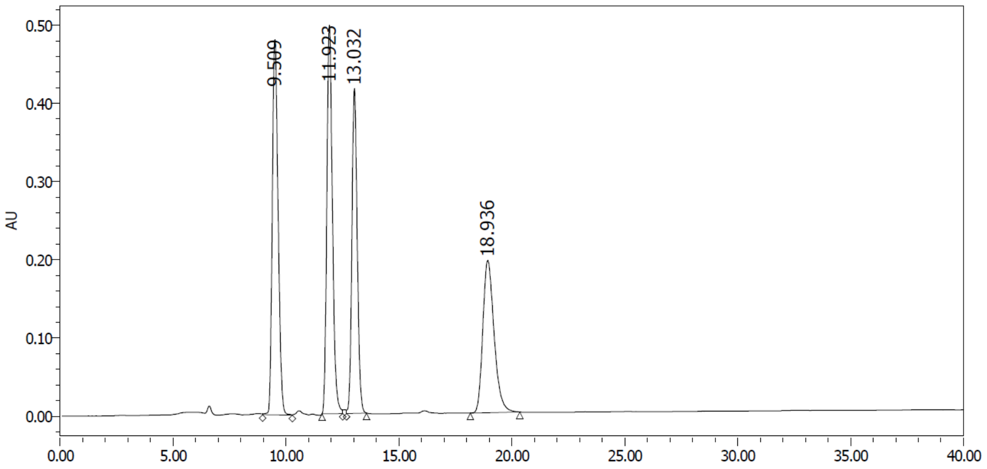

Peak #	Ret. Time	Area	Area %
1	9.509	8587360	28.03
2	11.923	8543344	27.88
3	13.032	6772201	22.10
4	18.936	6736309	21.99

chiral-**3da**

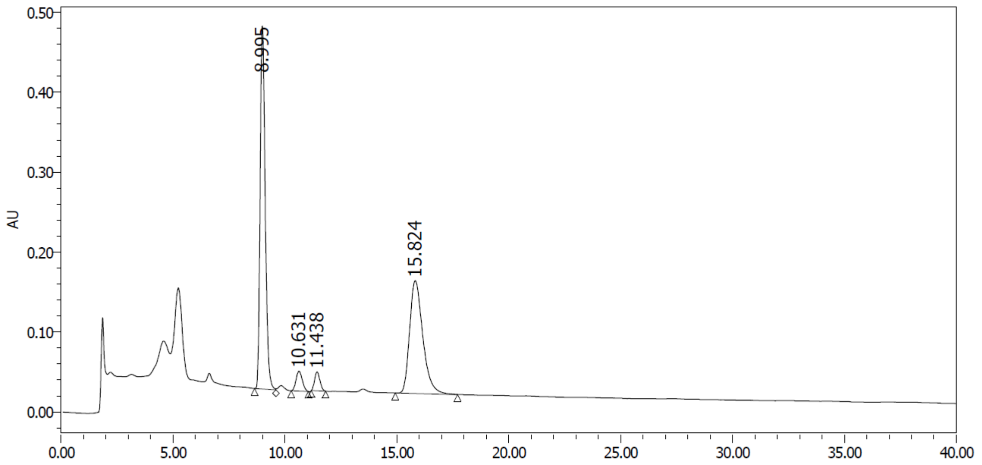

Peak #	Ret. Time	Area	Area %
1	8.995	7102952	51.69
2	10.631	486202	3.54
3	11.438	390179	2.84
4	15.824	5762244	41.93

3ea: The enantiomeric ratio was determined by HPLC analysis in comparison with authentic racemic material (CHIRALPAK AD-H column, 98.5/1.5 hexane/isopropyl alcohol, 0.5 mL/min, major isomers: t_R = 15.9, 36.3 min, minor isomers: t_R = 21.8, 22.8 min, UV detection at 210 nm, 30 °C).

rac-**3ea**

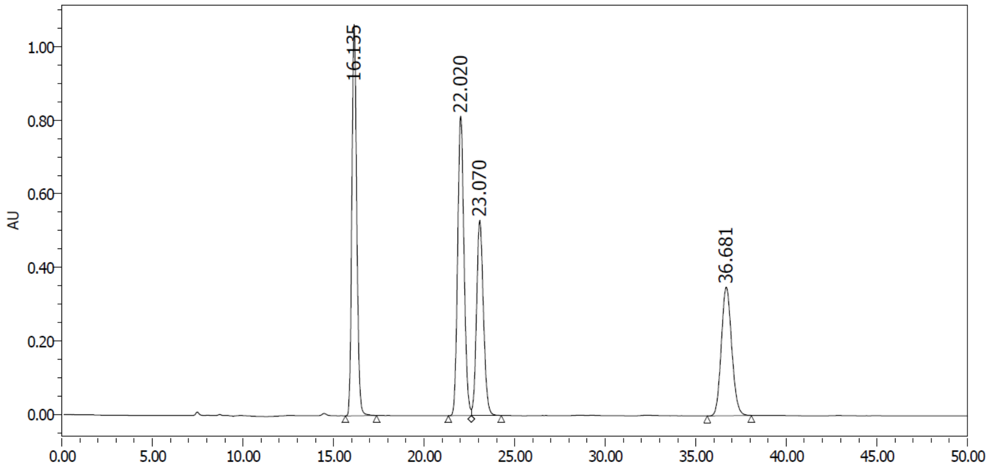

Peak #	Ret. Time	Area	Area %
1	16.135	19859027	29.28
2	22.020	19925726	29.38
3	23.070	14025690	20.68
4	36.681	14006295	20.65

chiral-**3ea**

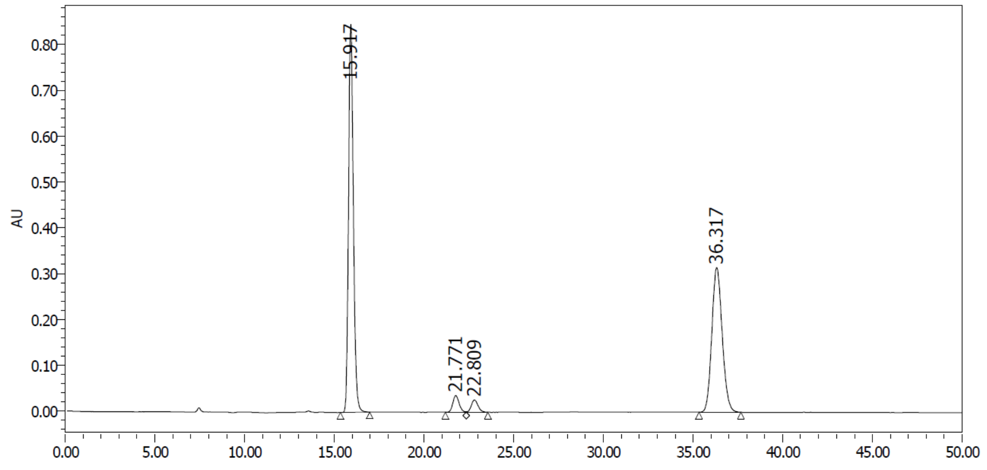

Peak #	Ret. Time	Area	Area %
1	15.917	15910977	52.69
2	21.771	886071	2.93
3	22.809	698640	2.31
4	36.317	12702211	42.06

3fa: The enantiomeric ratio was determined by HPLC analysis in comparison with authentic racemic material (CHIRALPAK AD-H column, 99/1 hexane/isopropyl alcohol, 0.5 mL/min, major isomers: t_R = 12.7, 23.0 min, minor isomers: t_R = 16.5, 17.8 min, UV detection at 210 nm, 30 °C).

rac-**3fa**

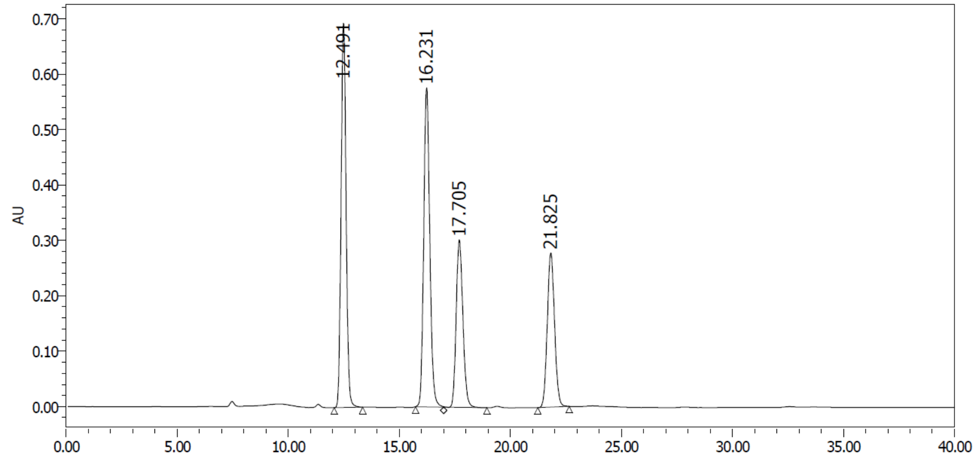

Peak #	Ret. Time	Area	Area %
1	12.491	10869945	31.51
2	16.231	10957573	31.76
3	17.705	6342685	18.38
4	21.825	6331498	18.35

chiral-**3fa**

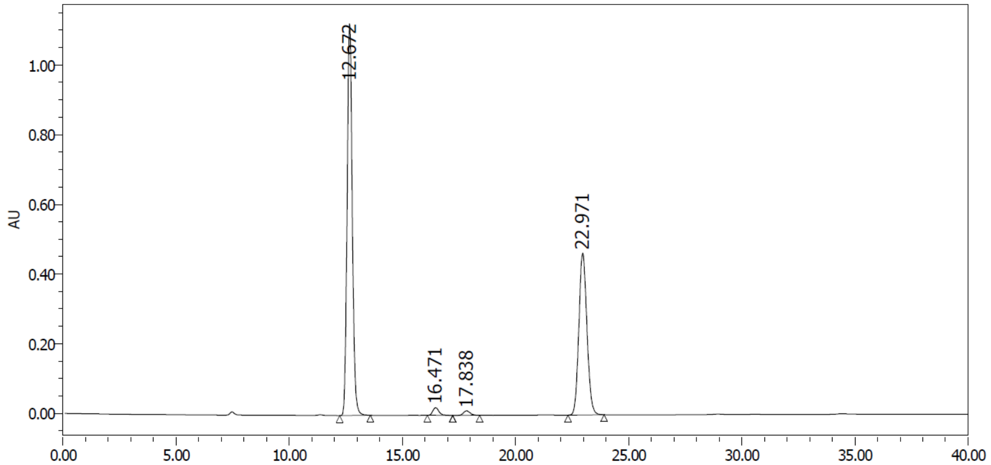

Peak #	Ret. Time	Area	Area %
1	12.672	17837279	59.37
2	16.471	418754	1.39
3	17.838	265435	0.88
4	22.971	11520361	38.35

3ga: The enantiomeric ratio was determined by HPLC analysis in comparison with authentic racemic material (CHIRALPAK AD-H column, 98.5/1.5 hexane/isopropyl alcohol, 0.5 mL/min, major isomers: t_R = 13.1, 34.9 min, minor isomers: t_R = 15.1, 21.6 min, UV detection at 210 nm, 30 ^oC).

rac-**3ga**

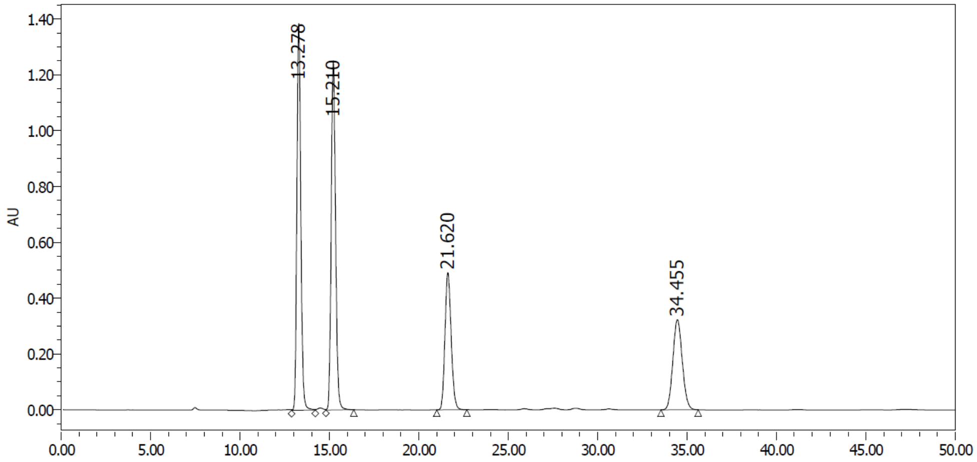

Peak #	Ret. Time	Area	Area %
1	13.278	21216192	32.07
2	15.210	21259839	32.14
3	21.620	11848573	17.91
4	34.455	11827994	17.88

chiral-**3ga**

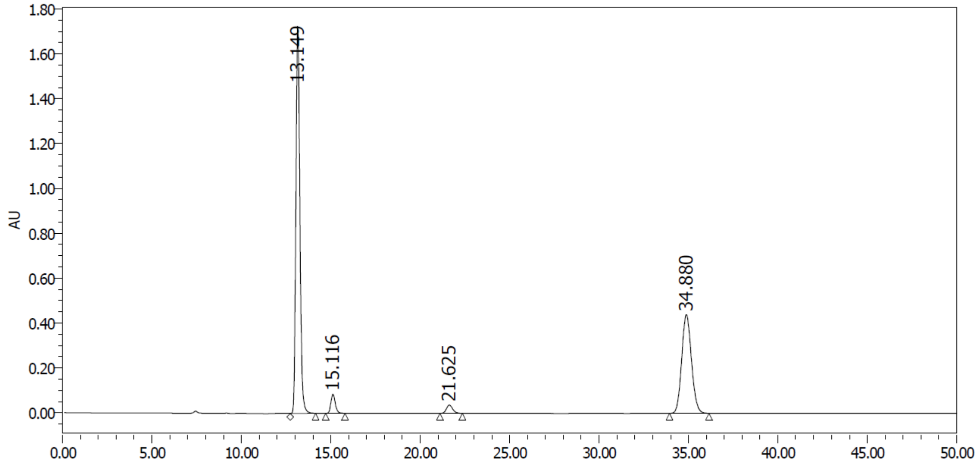

Peak #	Ret. Time	Area	Area %
1	13.149	26750136	58.39
2	15.116	1466423	3.20
3	21.625	901098	1.97
4	34.880	16694407	36.44

3ha: The enantiomeric ratio was determined by HPLC analysis in comparison with authentic racemic material (CHIRALPAK AD-H column, 98.5/1.5 hexane/isopropyl alcohol, 0.5 mL/min, major isomers: t_R = 12.7, 36.1 min, minor isomers: t_R = 15.3, 21.5 min, UV detection at 210 nm, 30 °C).

rac-**3ha**

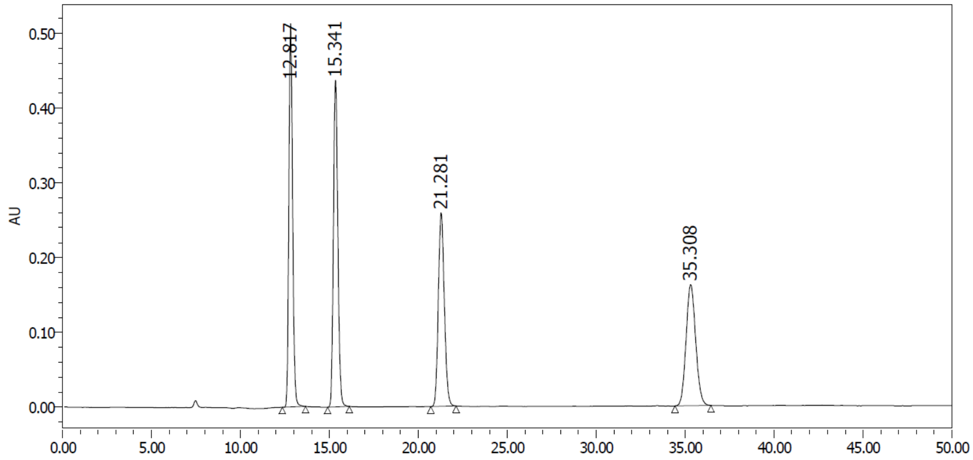

Peak #	Ret. Time	Area	Area %
1	12.817	7551255	27.72
2	15.341	7536829	27.66
3	21.281	6076949	22.31
4	35.308	6078437	22.31

chiral-**3ha**

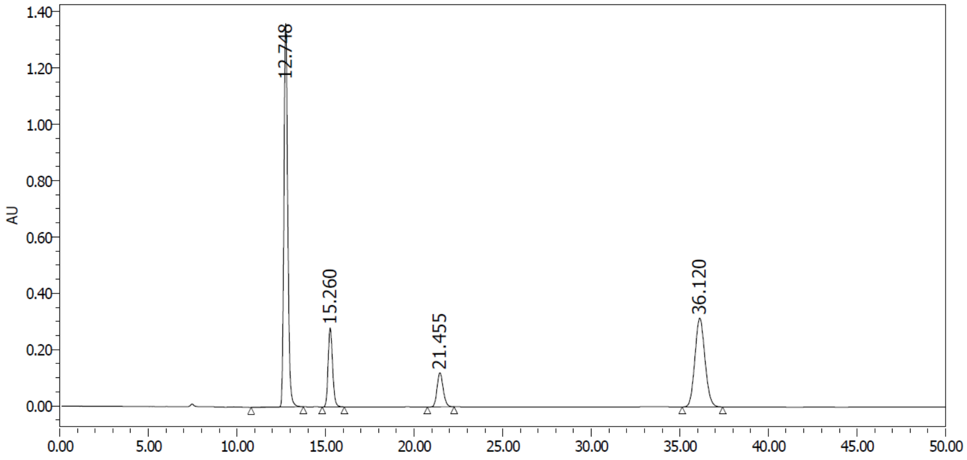

Peak #	Ret. Time	Area	Area %
1	12.748	20648707	50.46
2	15.260	4916948	12.02
3	21.455	2905904	7.10
4	36.120	12446540	30.42

3ia: The enantiomeric ratio was determined by HPLC analysis in comparison with authentic racemic material (CHIRALCEL OD-H column, 99.5/0.5 hexane/isopropyl alcohol, 0.5 mL/min, major isomers: t_R = 11.8, 19.3 min, minor isomers: t_R = 13.3, 13.8 min, UV detection at 210 nm, 30 °C).

rac-**3ia**

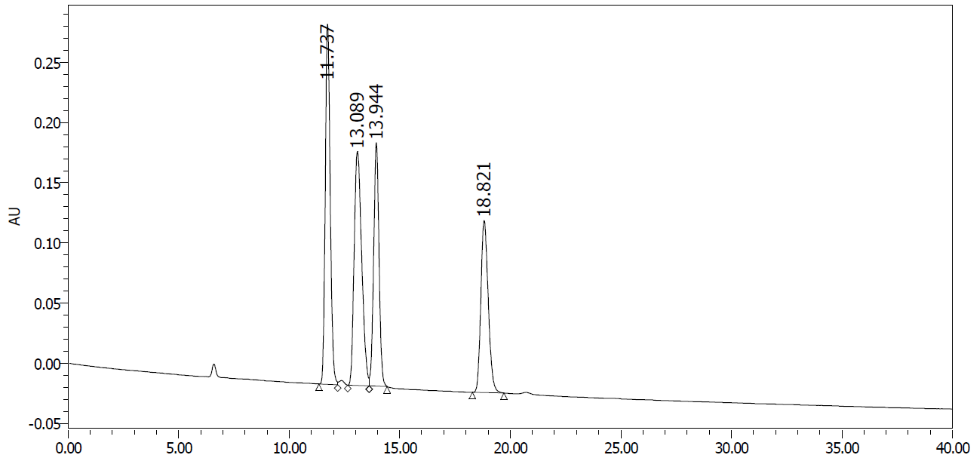

Peak #	Ret. Time	Area	Area %
1	11.737	4645329	29.10
2	13.089	4598657	28.80
3	13.944	3390284	21.23
4	18.821	3332349	20.87

chiral-**3ia**

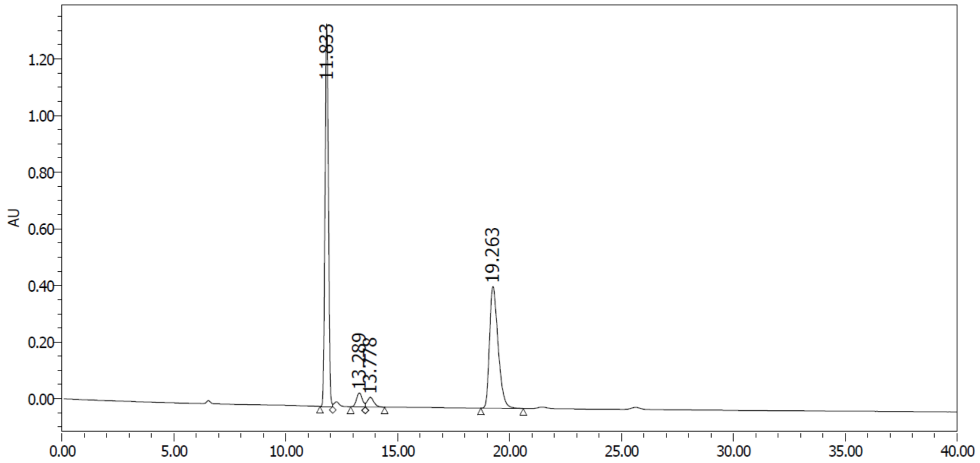

Peak #	Ret. Time	Area	Area %
1	11.833	14203569	52.18
2	13.289	880953	3.24
3	13.778	703011	2.58
4	19.263	11432023	42.00

3ja: The enantiomeric ratio was determined by HPLC analysis in comparison with authentic racemic material (CHIRAL ART Cellulose-SB (3 μm) column, 90/10 hexane/chloroform, 0.2 mL/min, major isomers: t_R = 39.0, 79.8 min, minor isomers: t_R = 44.7, 49.1 min, UV detection at 250 nm, 30 °C).

rac-**3ja**

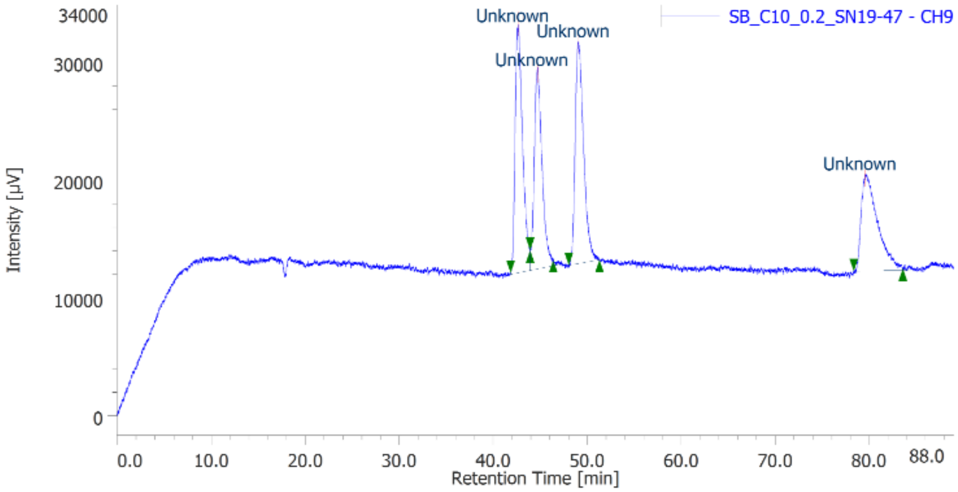

Peak #	Ret. Time	Area	Area %
1	42.627	1161310	26.93
2	44.693	991195	22.98
3	49.063	1168014	27.08
4	79.577	992327	23.01

chiral-**3ja**

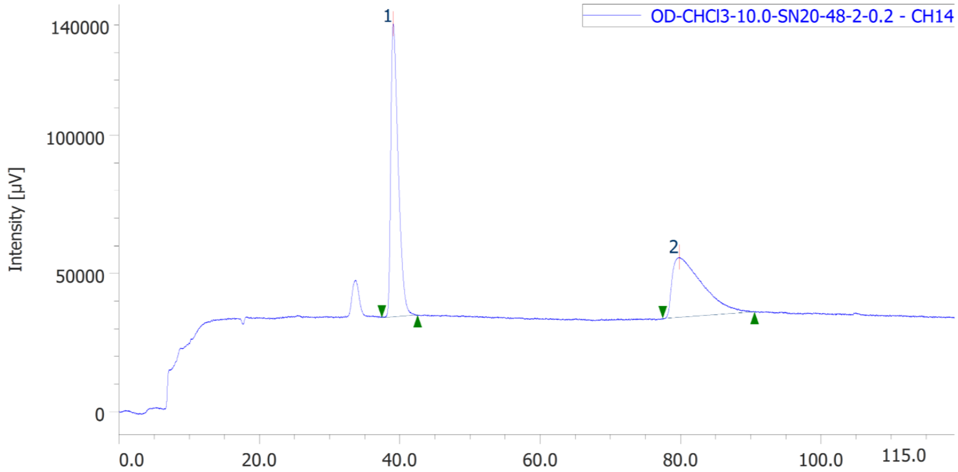

Peak #	Ret. Time	Area	Area %
1	39.033	7760429	54.50
2	79.783	6478002	45.50

3ka: The enantiomeric ratio was determined by HPLC analysis in comparison with authentic racemic material (CHIRALPAK AD-H column, 96/4 hexane/isopropyl alcohol, 0.5 mL/min, major isomers: t_R = 15.1, 36.1 min, minor isomers: t_R = 12.4, 18.3 min, UV detection at 210 nm, 30 °C).

rac-**3ka**

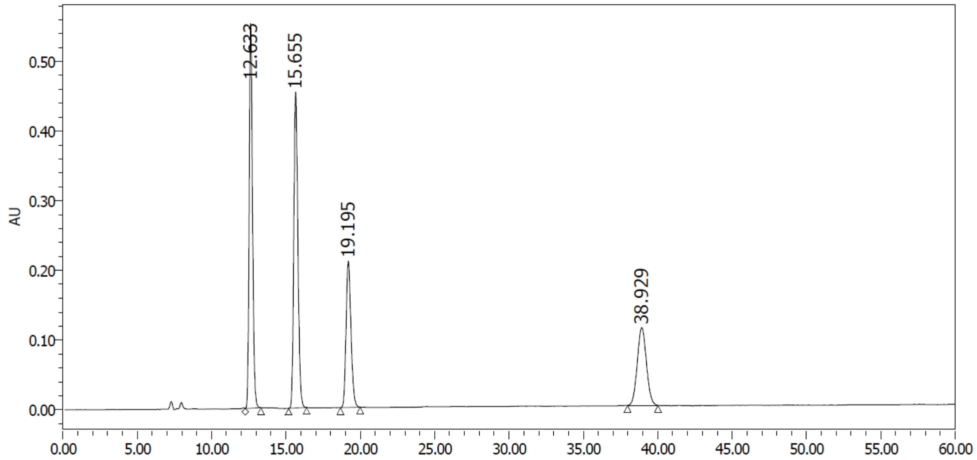

Peak #	Ret. Time	Area	Area %
1	12.633	8617861	32.09
2	15.655	8622121	32.11
3	19.195	4832743	18.00
4	38.929	4781176	17.80

chiral-**3ka**

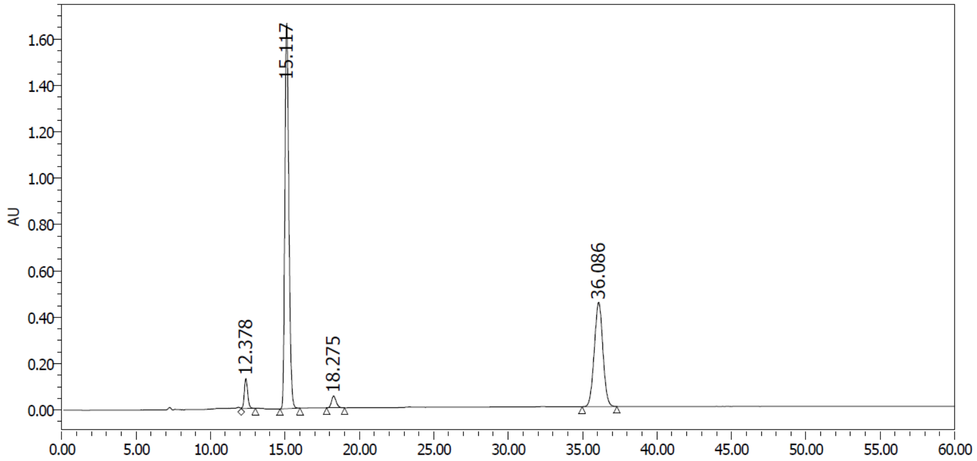

Peak #	Ret. Time	Area	Area %
1	12.378	2012879	3.86
2	15.117	30976564	59.36
3	18.275	1113584	2.13
4	36.086	18079876	34.65

3la: The enantiomeric ratio was determined by HPLC analysis in comparison with authentic racemic material (CHIRALPAK AD-H column, 99/1 hexane/isopropyl alcohol, 0.5 mL/min, major isomers: t_R = 9.3, 16.2 min, minor isomers: t_R = 10.5, 12.1 min, UV detection at 210 nm, 30 ^oC).

rac-**3la**

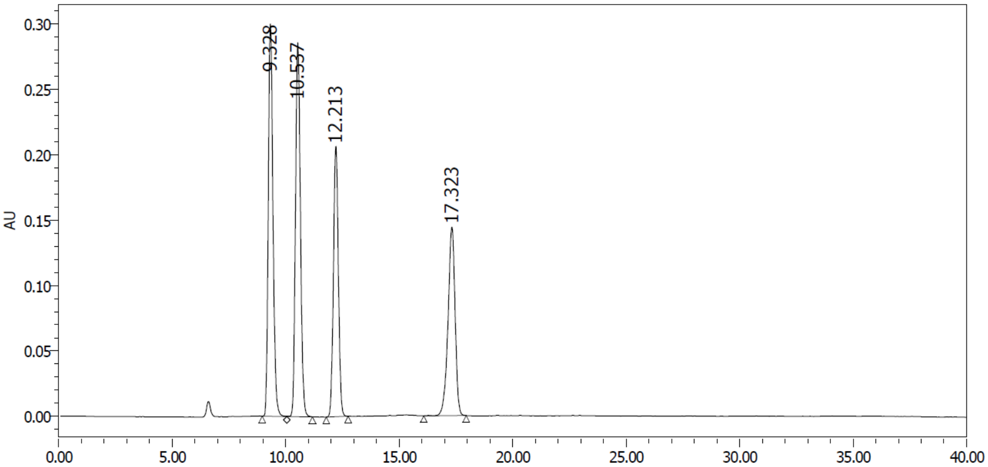

Peak #	Ret. Time	Area	Area %
1	9.328	4291645	28.96
2	10.537	4301534	29.03
3	12.213	3112969	21.01
4	17.323	3112612	21.00

chiral-**3la**

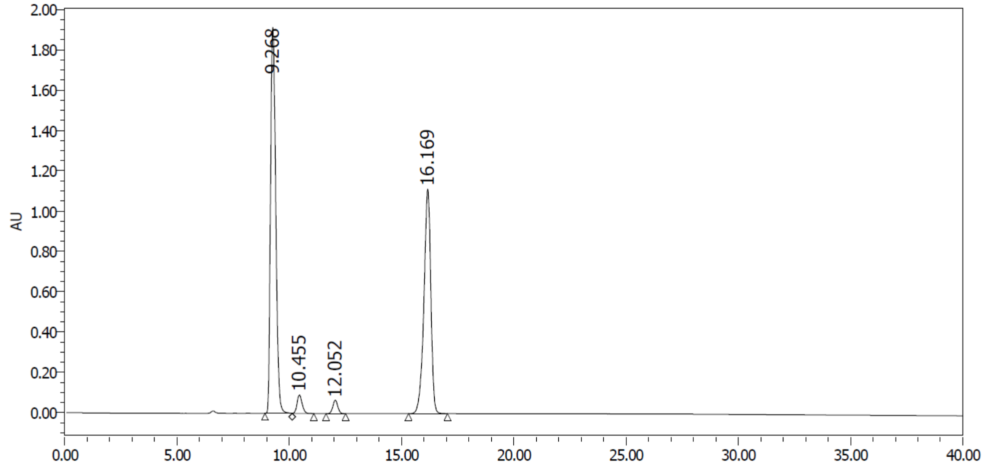

Peak #	Ret. Time	Area	Area %
1	9.268	30891283	54.09
2	10.455	1453248	2.54
3	12.052	1015540	1.78
4	16.169	23747163	41.58

3ma: The enantiomeric ratio was determined by HPLC analysis in comparison with authentic racemic material (CHIRALPAK AD-H column, 98.5/1.5 hexane/isopropyl alcohol, 0.5 mL/min, major isomers: t_R = 15.3, 37.2 min, minor isomers: t_R = 21.5, 22.3 min, UV detection at 210 nm, 30 °C).

rac-**3ma**

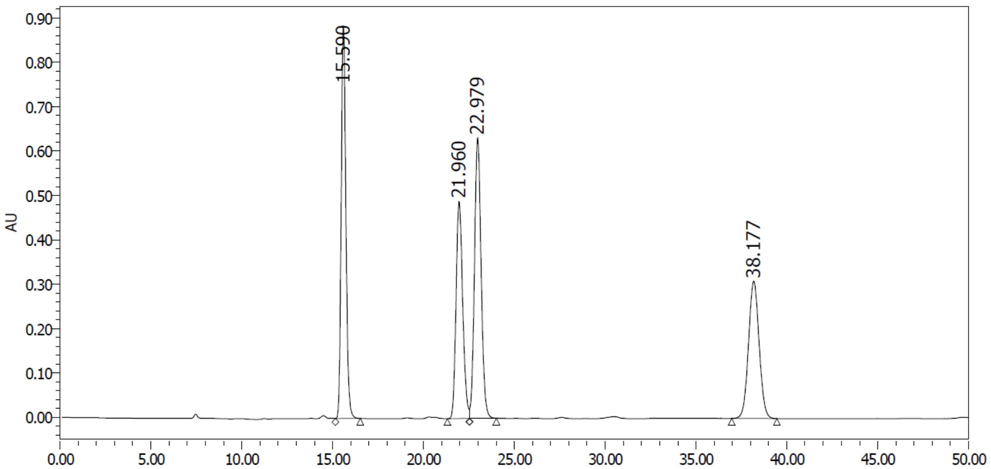

Peak #	Ret. Time	Area	Area %
1	15.590	15885982	27.89
2	21.960	12433187	21.83
3	22.979	16063633	28.20
4	38.177	12573995	22.08

chiral-**3ma**

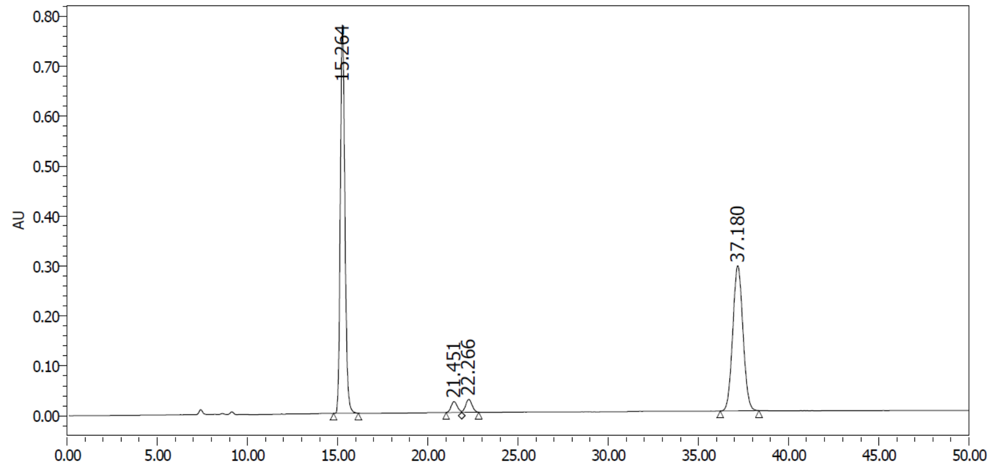

Peak #	Ret. Time	Area	Area %
1	15.264	13727949	52.12
2	21.451	522607	1.98
3	22.266	632813	2.40
4	37.180	11456165	43.49

3na: The enantiomeric ratio was determined by HPLC analysis in comparison with authentic racemic material (CHIRALPAK AD-H column, 98.5/1.5 hexane/isopropyl alcohol, 0.5 mL/min, major isomers: t_R = 12.3, 23.4 min, minor isomers: t_R = 12.9, 17.0 min, UV detection at 210 nm, 30 °C).

rac-**3na**

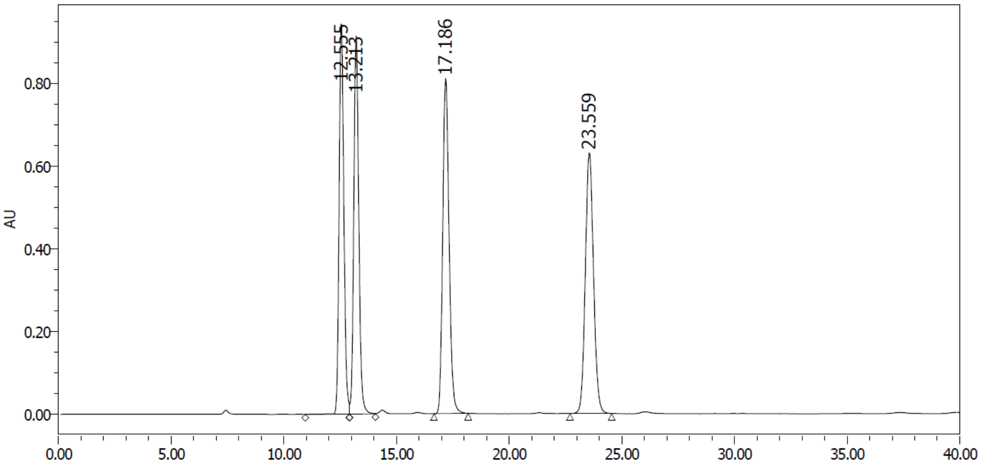

Peak #	Ret. Time	Area	Area %
1	12.555	13788061	23.13
2	13.213	14022128	23.52
3	17.186	15864234	26.61
4	23.559	15947191	26.75

chiral-**3na**

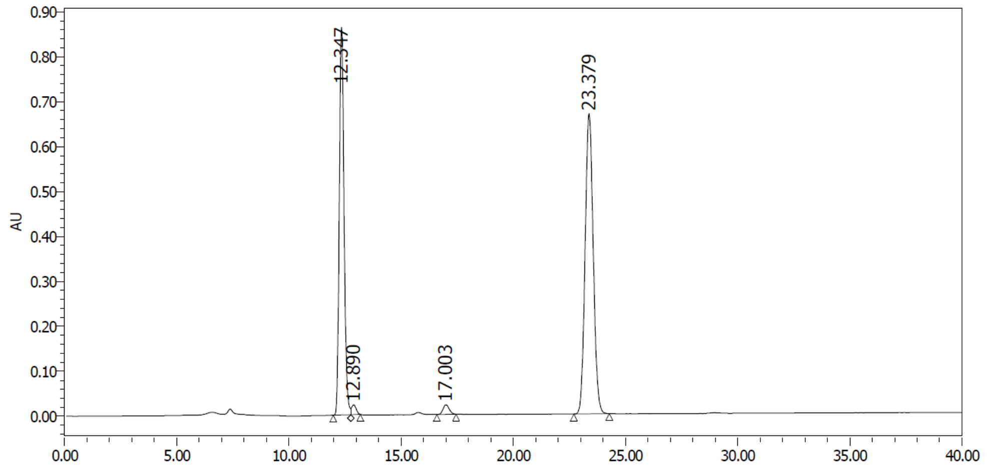

Peak #	Ret. Time	Area	Area %
1	12.347	12691961	42.05
2	12.890	305102	1.01
3	17.003	412762	1.37
4	23.379	16770707	55.57

3pa: The enantiomeric ratio was determined by HPLC analysis in comparison with authentic racemic material (CHIRALCEL OD-H column, 99.4/0.6 hexane/isopropyl alcohol, 0.5 mL/min, major isomers: t_R = 18.0, 22.6 min, minor isomers: t_R = 15.7, 21.6 min, UV detection at 210 nm, 30 °C).

rac-**3pa**

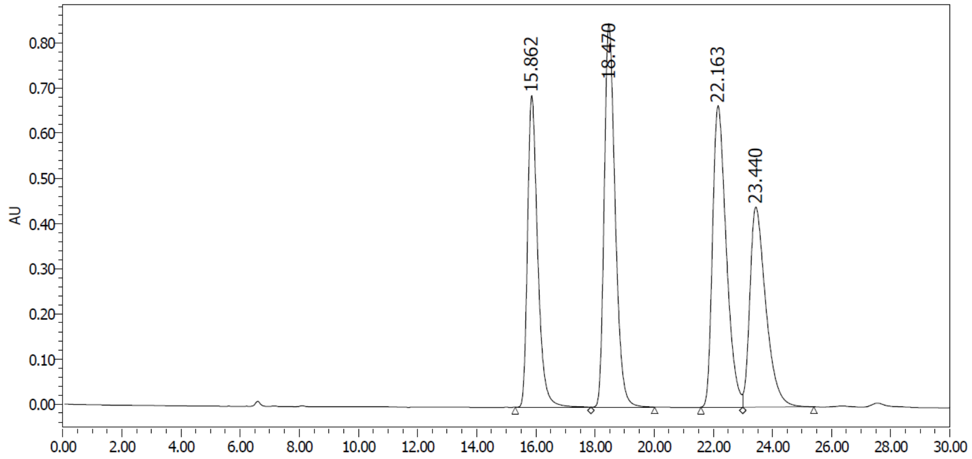

Peak #	Ret. Time	Area	Area %
1	15.862	16503163	21.58
2	18.470	21778585	28.48
3	22.163	21578229	28.21
4	23.440	16618705	21.73

chiral-**3pa**

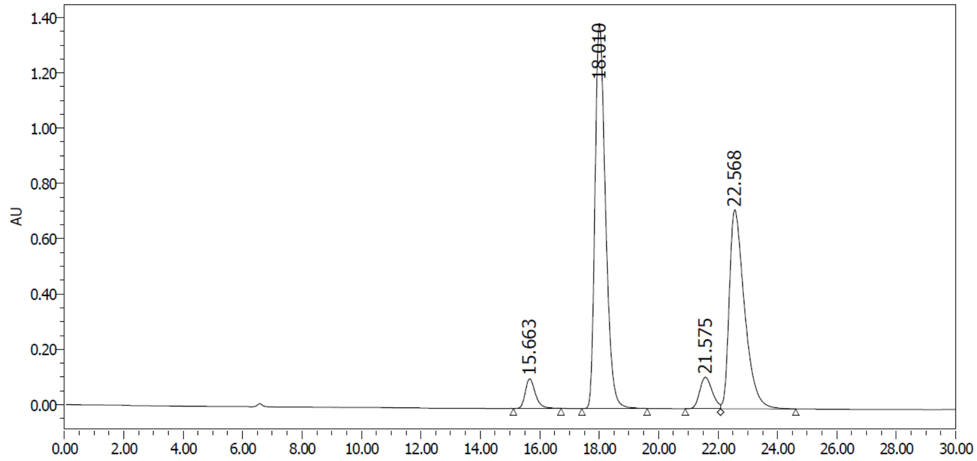

Peak #	Ret. Time	Area	Area %
1	15.663	2522197	3.84
2	18.010	34165441	52.02
3	21.575	3346030	5.09
4	22.568	25646255	39.05

3qa: The enantiomeric ratio was determined by HPLC analysis in comparison with authentic racemic material (CHIRALPAK AD-H column, 99.8/0.2 hexane/isopropyl alcohol, 0.5 mL/min, major isomers: t_R = 14.6, 28.6 min, minor isomers: t_R = 17.2, 20.9 min, UV detection at 210 nm, 30 °C).

rac-**3qa**

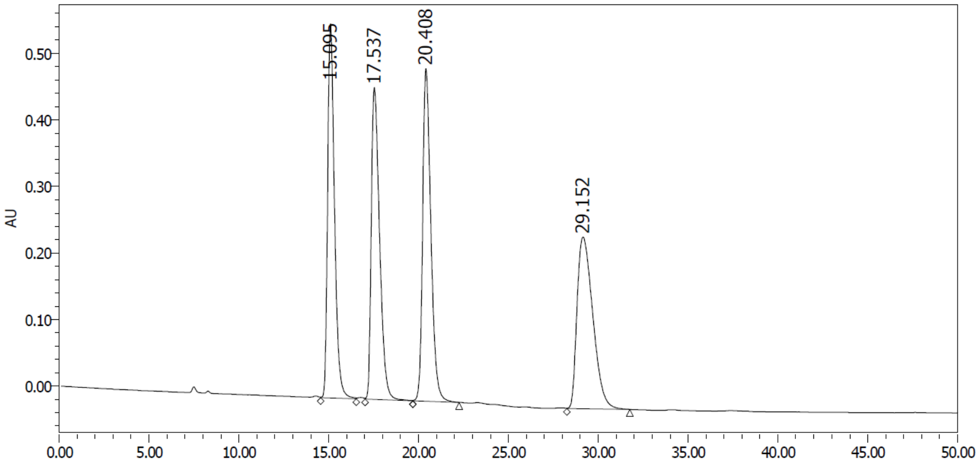

Peak #	Ret. Time	Area	Area %
1	15.095	14914208	24.21
2	17.537	15004617	24.36
3	20.408	15910802	25.83
4	29.152	15763256	25.59

chiral-**3qa**

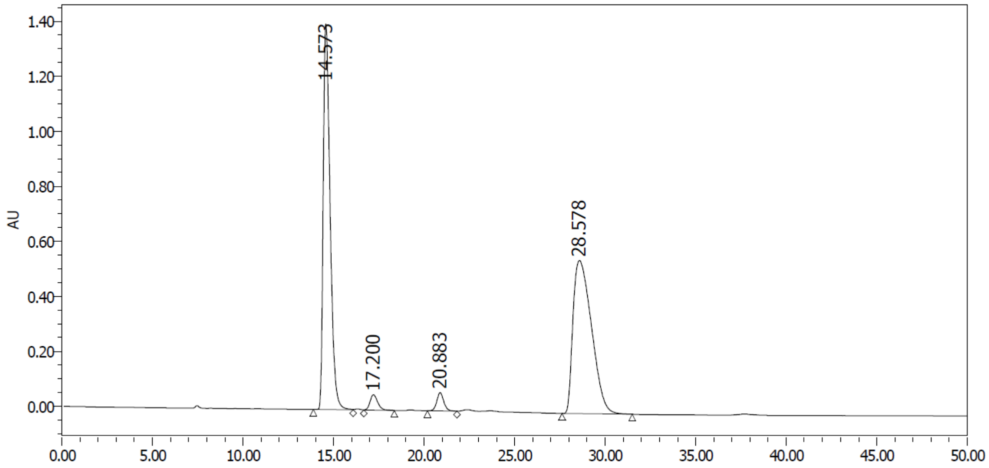

Peak #	Ret. Time	Area	Area %
1	14.573	37070037	46.19
2	17.200	1644499	2.05
3	20.883	1811577	2.26
4	28.578	39731059	49.50

3ra: The enantiomeric ratio was determined by HPLC analysis in comparison with authentic racemic material (CHIRALPAK AD-H column, 99.4/0.6 hexane/isopropyl alcohol, 0.5 mL/min, major isomers: t_R = 13.7, 27.1 min, minor isomers: t_R = 12.9, 21.9 min, UV detection at 210 nm, 30 °C).

rac-**3ra**

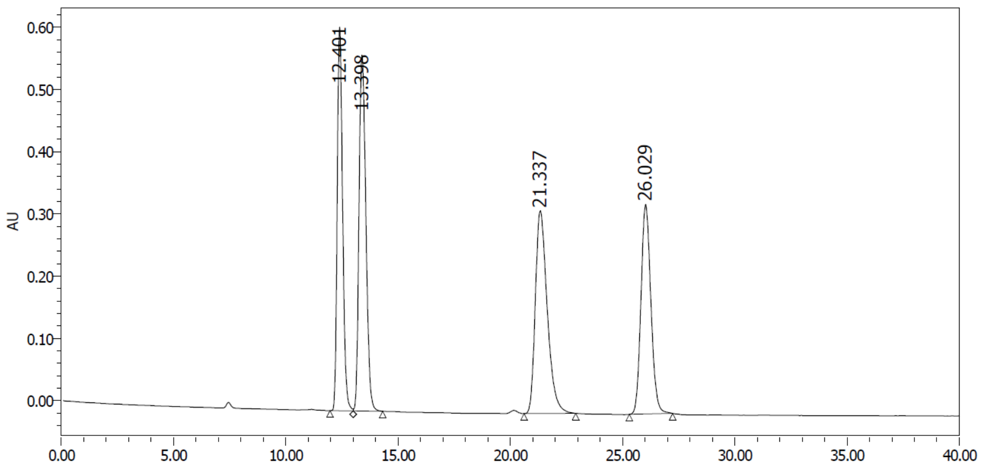

Peak #	Ret. Time	Area	Area %
1	12.401	10256215	22.94
2	13.398	12077521	27.02
3	21.337	12011397	26.87
4	26.029	10358185	23.17

chiral-**3ra**

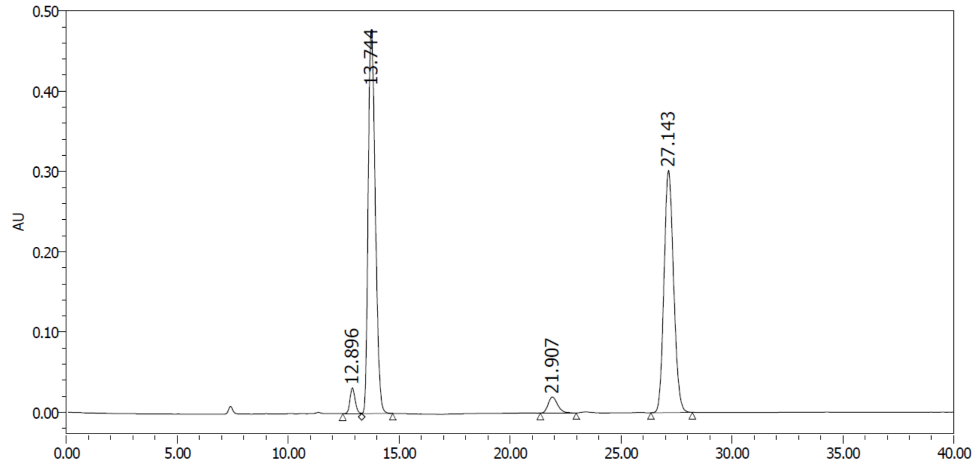

Peak #	Ret. Time	Area	Area %
1	12.896	516852	2.48
2	13.744	10640219	51.08
3	21.907	585849	2.81
4	27.143	9087911	43.63

3sa: The enantiomeric ratio was determined by HPLC analysis in comparison with authentic racemic material (CHIRALPAK AD-H column, 98.5/1.5 hexane/isopropyl alcohol, 0.5 mL/min, major isomers: t_R = 21.6, 53.3 min, minor isomers: t_R = 34.7, 37.5 min, UV detection at 210 nm, 30 ^{o}C).

rac-**3sa**

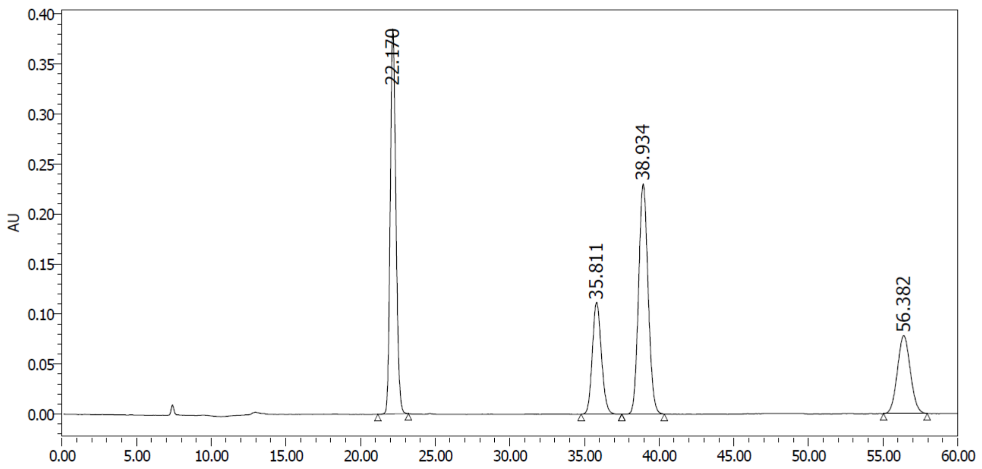

Peak #	Ret. Time	Area	Area %
1	22.170	10173548	34.18
2	35.811	4716004	15.84
3	38.934	10187466	34.22
4	56.382	4690130	15.76

chiral-**3sa**

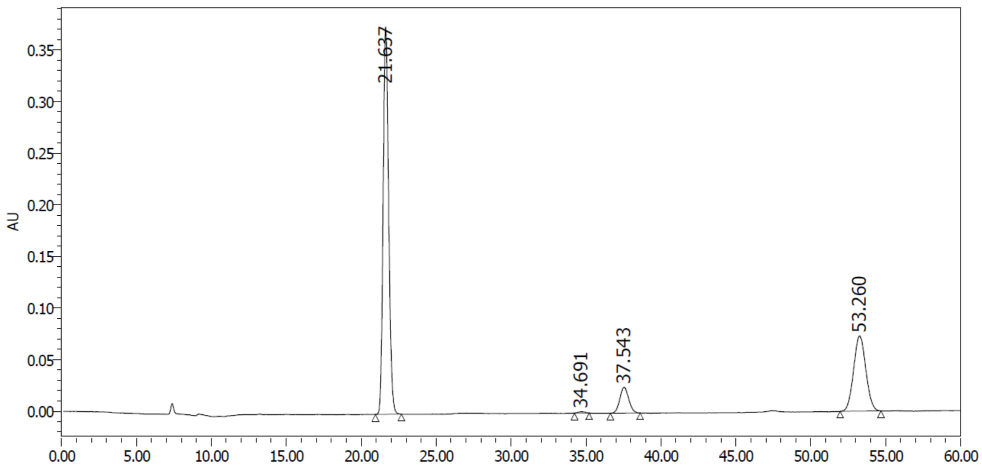

Peak #	Ret. Time	Area	Area %
1	21.637	9585123	64.80
2	34.691	37455	0.25
3	37.543	1032938	6.98
4	53.260	4135482	27.96

3ta: The enantiomeric ratio was determined by HPLC analysis in comparison with authentic racemic material (CHIRALPAK AD-H column, 98.8/1.2 hexane/isopropyl alcohol, 0.5 mL/min, major isomers: t_R = 17.8, 45.8 min, minor isomers: t_R = 34.9, 35.8 min, UV detection at 210 nm, 30 °C).

rac-**3ta**

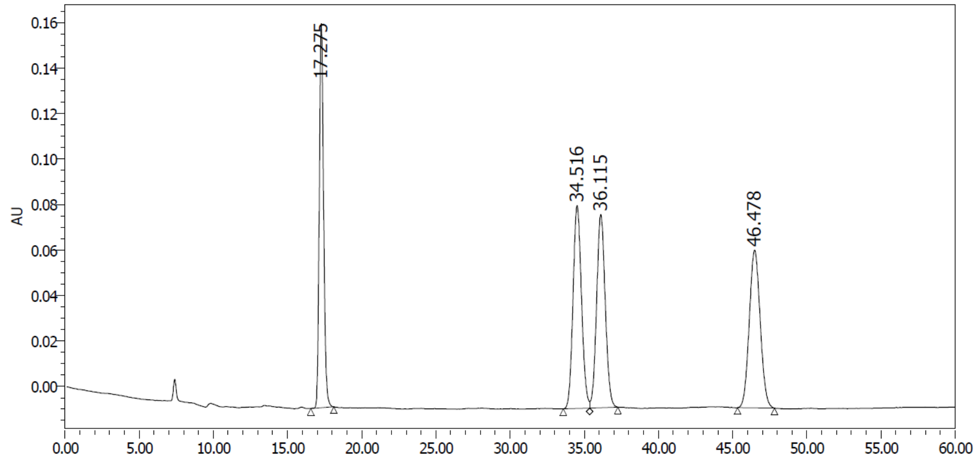

Peak #	Ret. Time	Area	Area %
1	17.275	3432917	24.61
2	34.516	3542709	25.39
3	36.115	3434780	24.62
4	46.478	3541003	25.38

chiral-**3ta**

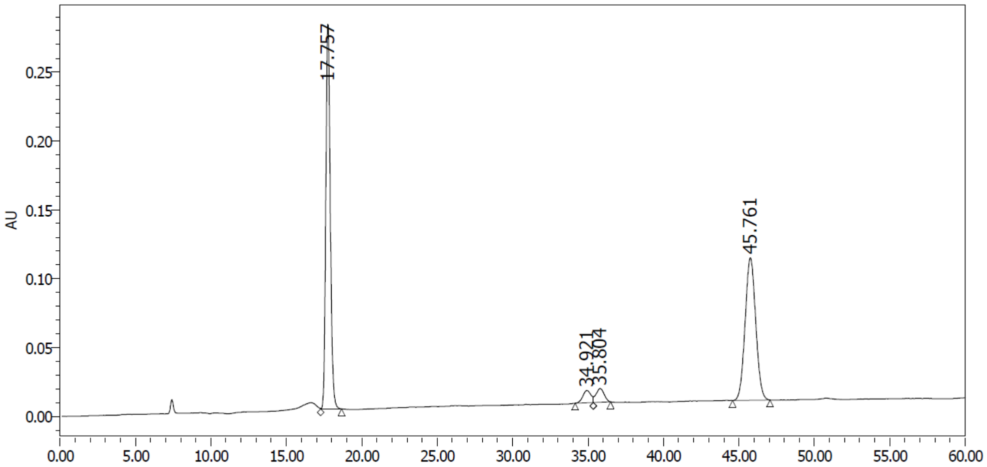

Peak #	Ret. Time	Area	Area %
1	17.757	5742931	49.96
2	34.921	336137	2.92
3	35.804	377598	3.28
4	45.761	5038013	43.83

3ua: The enantiomeric ratio was determined by HPLC analysis in comparison with authentic racemic material (CHIRALPAK AD-H column, 98.5/1.5 hexane/isopropyl alcohol, 0.5 mL/min, major isomers: t_R = 23.3, 37.9 min, minor isomers: t_R = 25.6, 45.2 min, UV detection at 210 nm, 30 °C).

rac-**3ua**

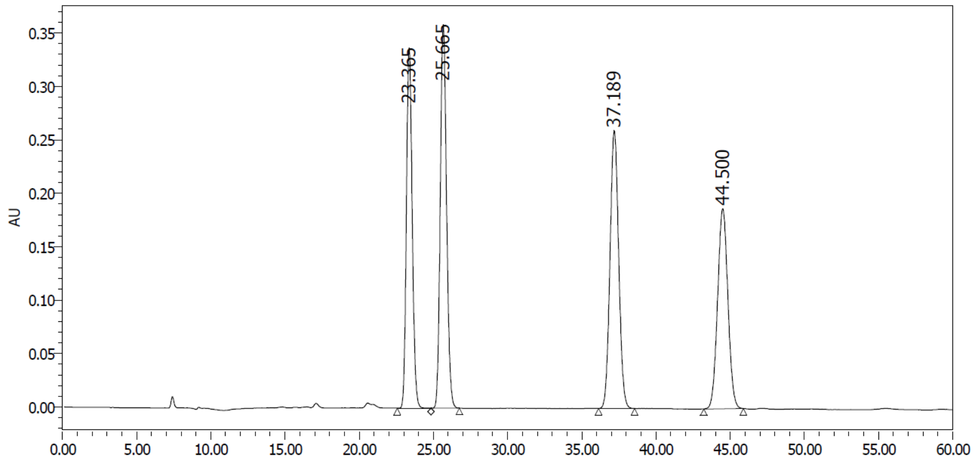

Peak #	Ret. Time	Area	Area %
1	23.365	9063520	23.10
2	25.665	10561383	26.92
3	37.189	10561940	26.92
4	44.500	9048327	23.06

chiral-**3ua**

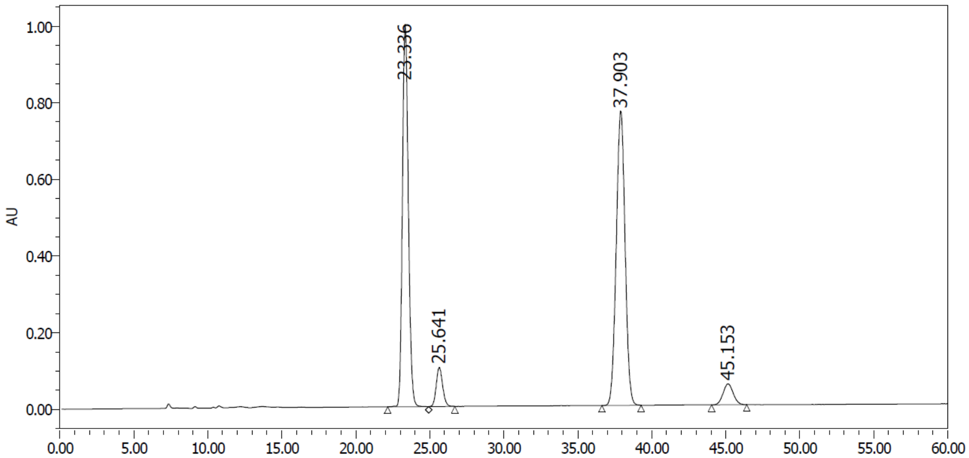

Peak #	Ret. Time	Area	Area %
1	23.336	27540745	42.32
2	25.641	3062977	4.71
3	37.903	31853033	48.95
4	45.153	2615833	4.02

3wa: The enantiomeric ratio was determined by HPLC analysis in comparison with authentic racemic material (CHIRALPAK AD-H column, 99/1 hexane/isopropyl alcohol, 0.5 mL/min, major isomers: t_R = 14.3, 17.2 min, minor isomers: t_R = 26.6, 29.0 min, UV detection at 210 nm, 30 °C).

rac-**3wa**

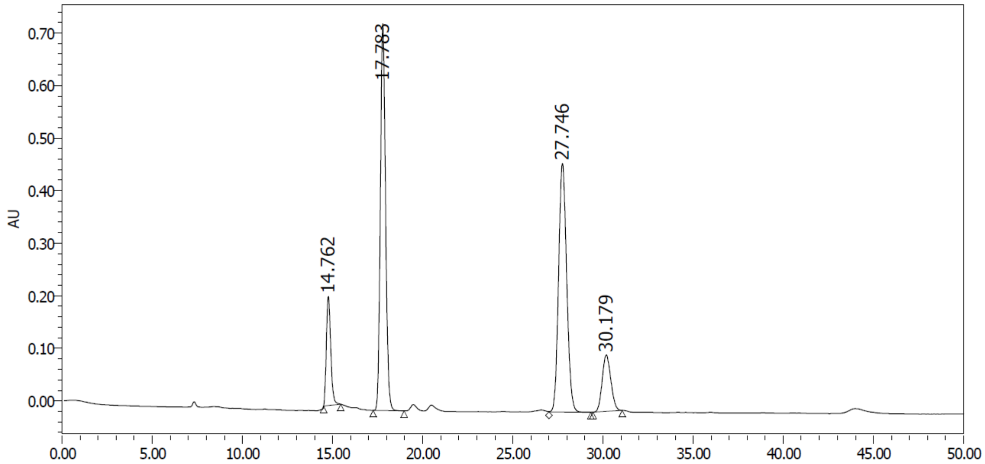

Peak #	Ret. Time	Area	Area %
1	14.762	3520080	9.66
2	17.783	14647851	40.20
3	27.746	14706970	40.36
4	30.179	3565240	9.78

chiral-**3wa**

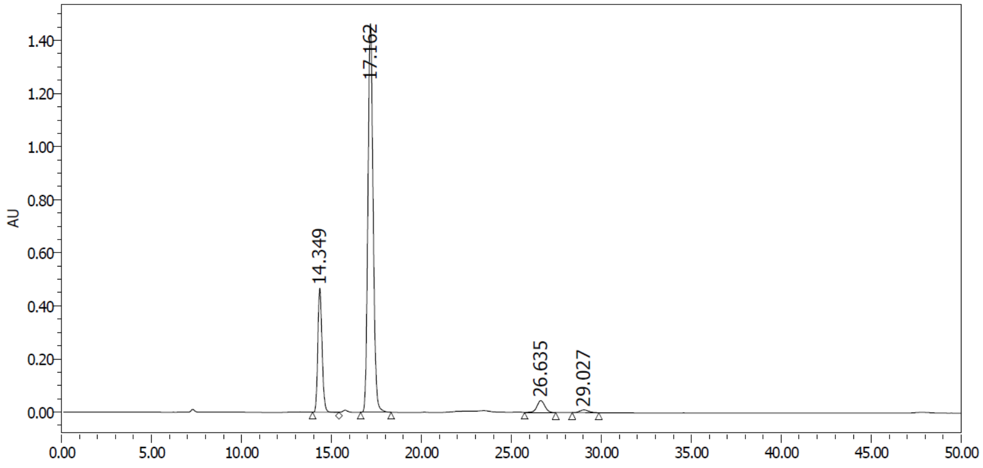

Peak #	Ret. Time	Area	Area %
1	14.349	7679534	19.73
2	17.162	29534984	75.88
3	26.635	1365724	3.51
4	29.027	343597	0.88

3xa: The enantiomeric ratio was determined by HPLC analysis in comparison with authentic racemic material (CHIRALPAK AD-H column, 99/1 hexane/isopropyl alcohol, 0.5 mL/min, major isomers: t_R = 13.0, 16.3 min, minor isomers: t_R = 14.8, 17.4 min, UV detection at 210 nm, 30 °C).

rac-**3xa**

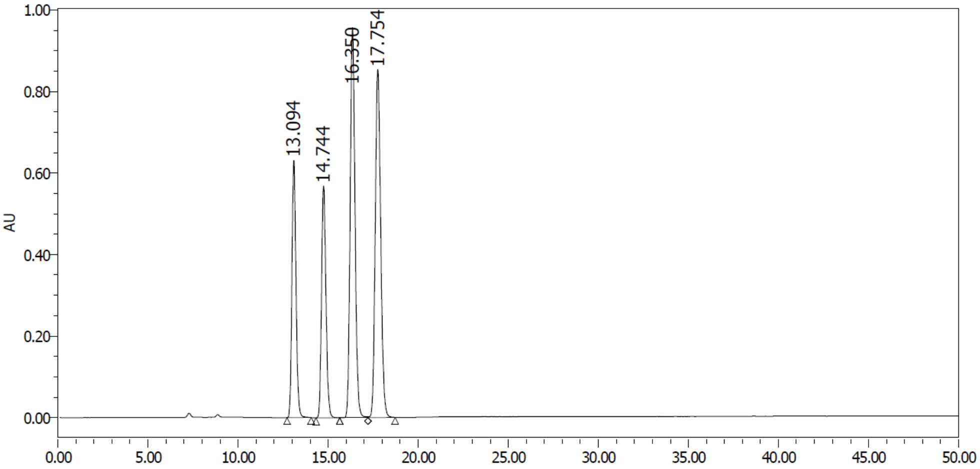

Peak #	Ret. Time	Area	Area %
1	13.094	9821608	17.76
2	14.744	9663391	17.47
3	16.350	17905948	32.37
4	17.754	17917201	32.40

chiral-**3xa**

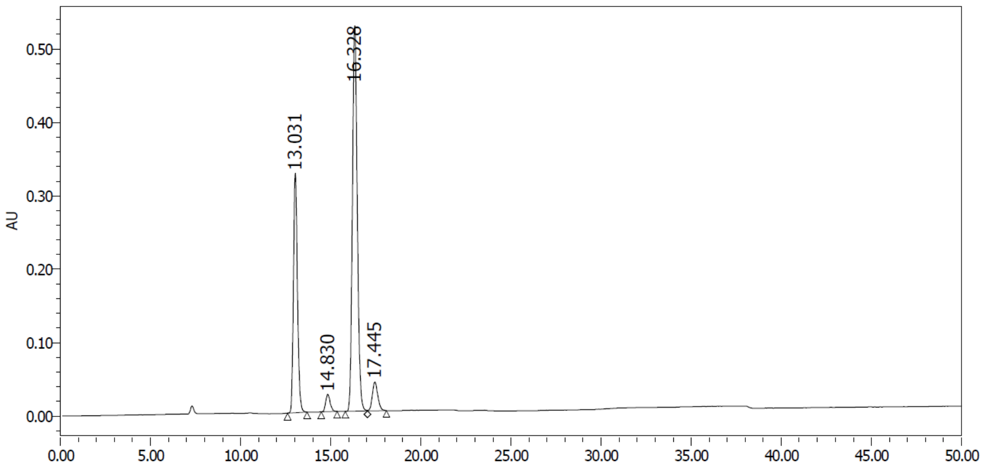

Peak #	Ret. Time	Area	Area %
1	13.031	4993560	31.03
2	14.830	408566	2.54
3	16.328	9869323	61.32
4	17.445	823382	5.12

3ya: The enantiomeric ratio was determined by HPLC analysis in comparison with authentic racemic material (CHIRALPAK AD-H column, 99/1 hexane/isopropyl alcohol, 0.5 mL/min, t_R = 16.6, 18.6 min, UV detection at 210 nm, 30 ^oC).

3ya from nonenantioselective conditions (Scheme 5)

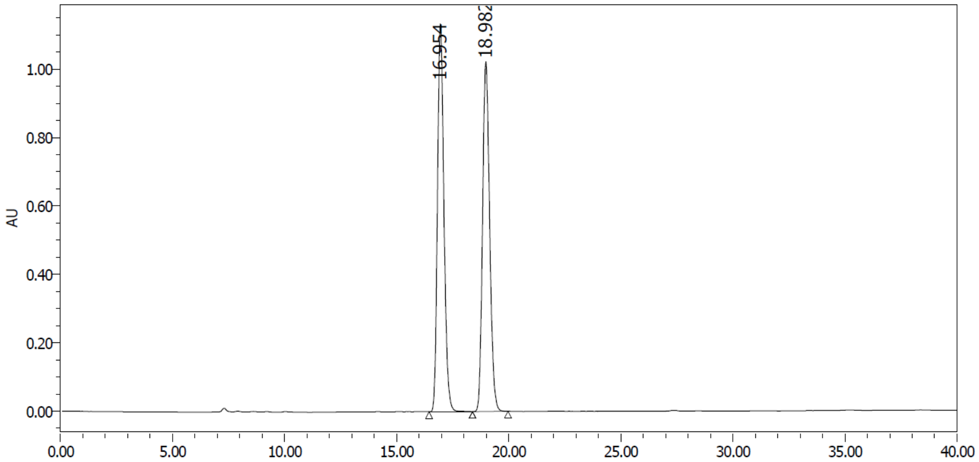

Peak #	Ret. Time	Area	Area %
1	16.954	22723537	50.26
2	18.982	22490070	49.74

3ya from enantioselective conditions (Scheme 6)

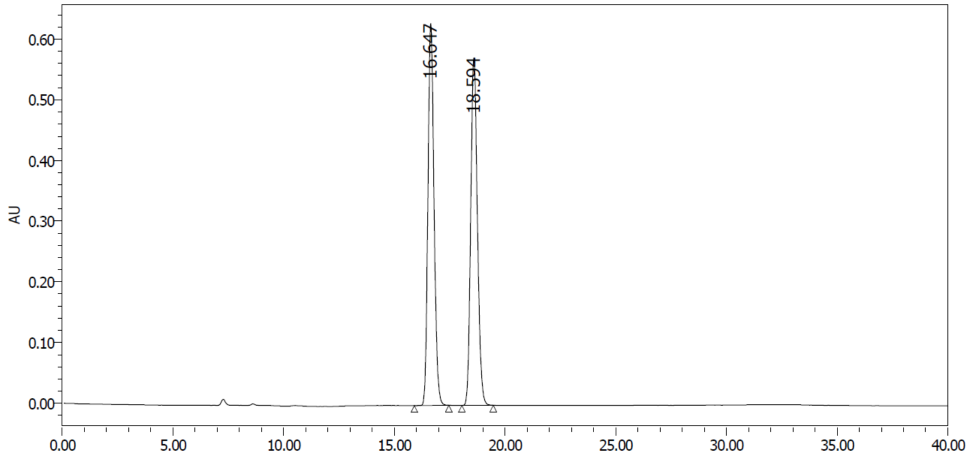

Peak #	Ret. Time	Area	Area %
1	16.647	12011368	49.99
2	18.594	12014967	50.01

3ab: The enantiomeric ratio was determined by HPLC analysis in comparison with authentic racemic material (CHIRALPAK AD-H column, 99.8/0.2 hexane/isopropyl alcohol, 0.5 mL/min, major isomers: t_R = 17.0, 21.9 min, minor isomers: t_R = 16.6, 24.0 min, UV detection at 210 nm, 30 °C).

rac-**3ab**

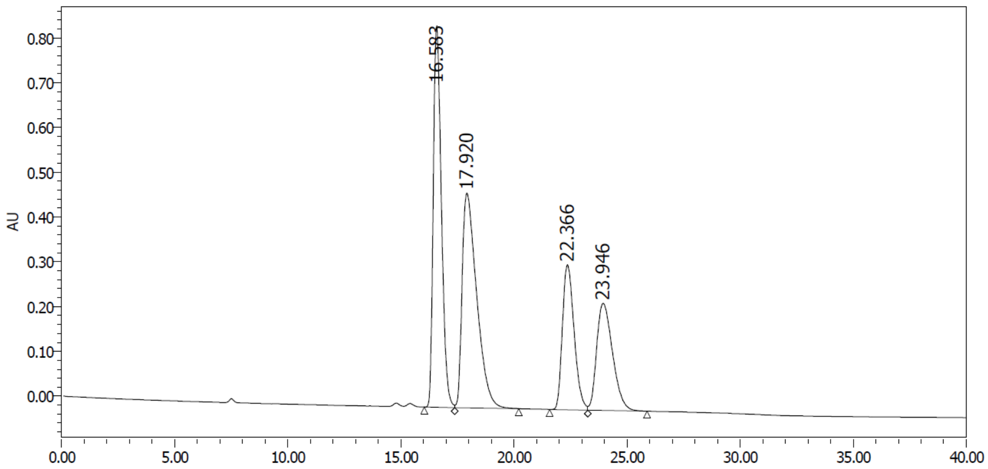

Peak #	Ret. Time	Area	Area %
1	16.583	21211070	32.15
2	17.920	21426078	32.47
3	22.366	11653283	17.66
4	23.946	11694050	17.72

chiral-**3ab**

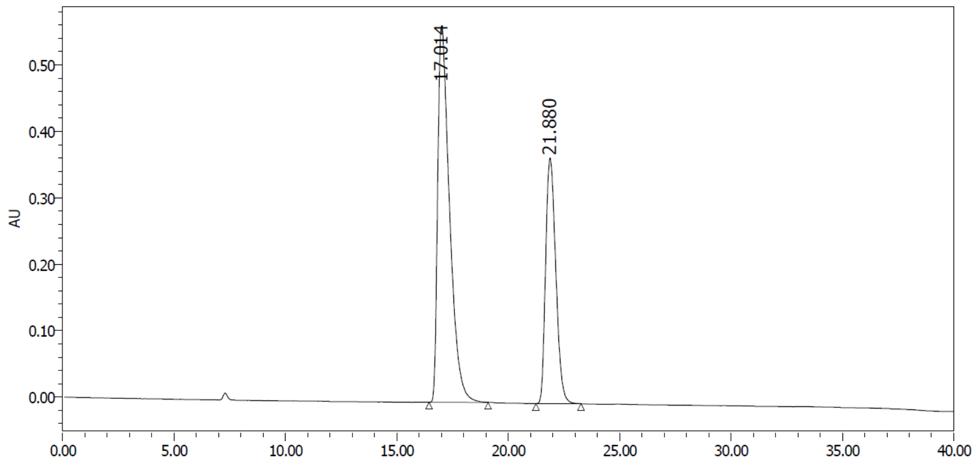

Peak #	Ret. Time	Area	Area %
1	17.014	20997807	64.23
2	21.880	11695700	35.77

3ac: The enantiomeric ratio was determined by HPLC analysis in comparison with authentic racemic material (CHIRALPAK AD-H column, 99/1 hexane/isopropyl alcohol, 0.5 mL/min, major isomers: t_R = 8.7, 13.7 min, minor isomers: t_R = 9.5, 10.1 min, UV detection at 210 nm, 30 °C).

rac-**3ac**

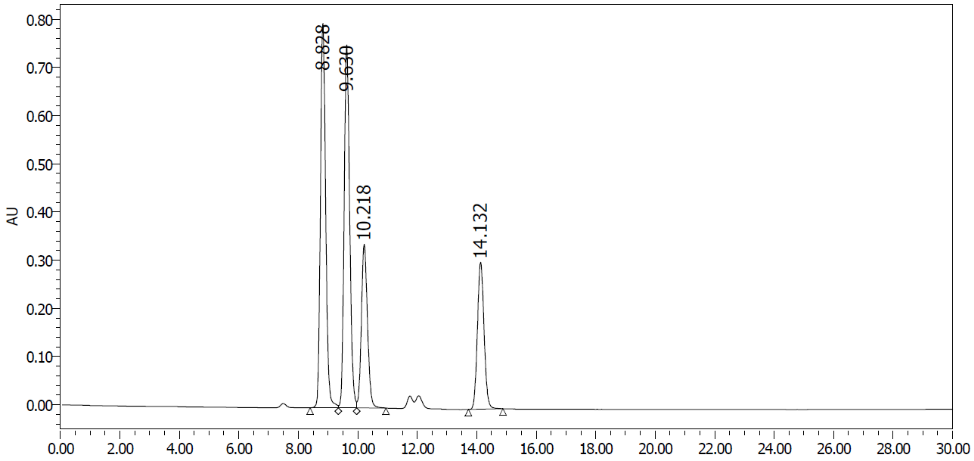

Peak #	Ret. Time	Area	Area %
1	8.828	9800183	33.66
2	9.630	9744989	33.47
3	10.218	4826371	16.58
4	14.132	4745349	16.30

chiral-**3ac**

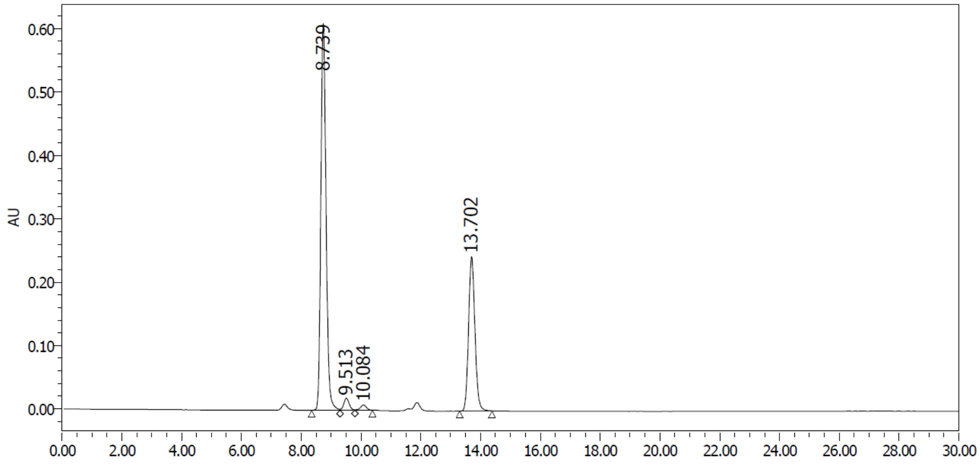

Peak #	Ret. Time	Area	Area %
1	8.739	7374948	65.03
2	9.513	238579	2.10
3	10.084	112675	0.99
4	13.702	3614785	31.87

3ad: The enantiomeric ratio was determined by HPLC analysis in comparison with authentic racemic material (CHIRALPAK AD-H column, 99.2/0.8 hexane/isopropyl alcohol, 0.5 mL/min, major isomers: t_R = 10.4, 19.8 min, minor isomers: t_R = 11.5, 12.4 min, UV detection at 210 nm, 30 °C).

rac-**3ad**

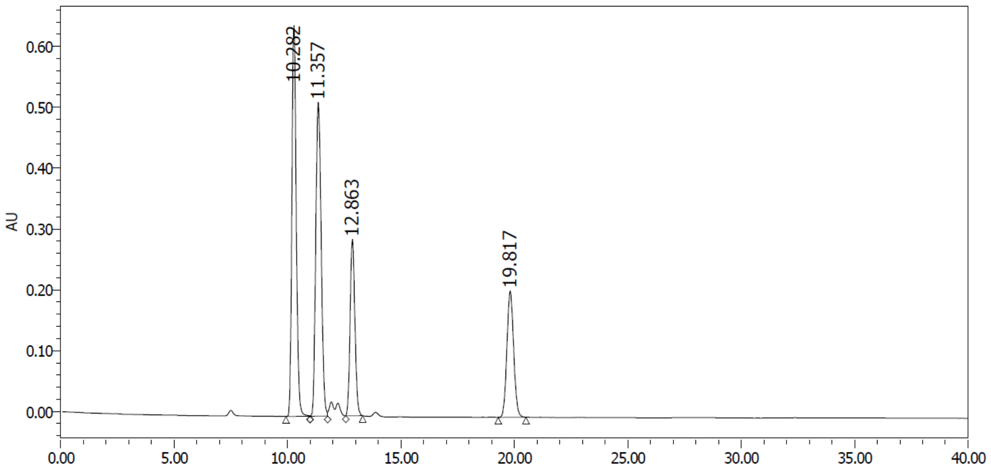

Peak #	Ret. Time	Area	Area %
1	10.282	8601477	33.46
2	11.357	8585506	33.40
3	12.863	4253114	16.55
4	19.817	4263742	16.59

chiral-**3ad**

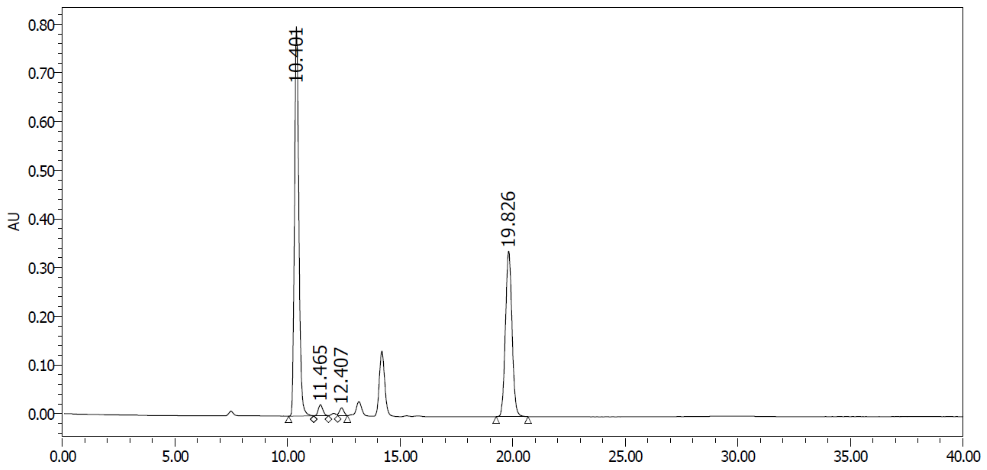

Peak #	Ret. Time	Area	Area %
1	10.401	10794966	59.28
2	11.465	325337	1.79
3	12.407	211374	1.16
4	19.826	6877956	37.77

3ae: The enantiomeric ratio was determined by HPLC analysis in comparison with authentic racemic material (CHIRALCEL OD-H column, 99.6/0.4 hexane/isopropyl alcohol, 0.5 mL/min, major isomers: t_R = 25.1, 36.3 min, minor isomers: t_R = 28.5, 31.5 min, UV detection at 210 nm, 30 °C).

rac-**3ae**

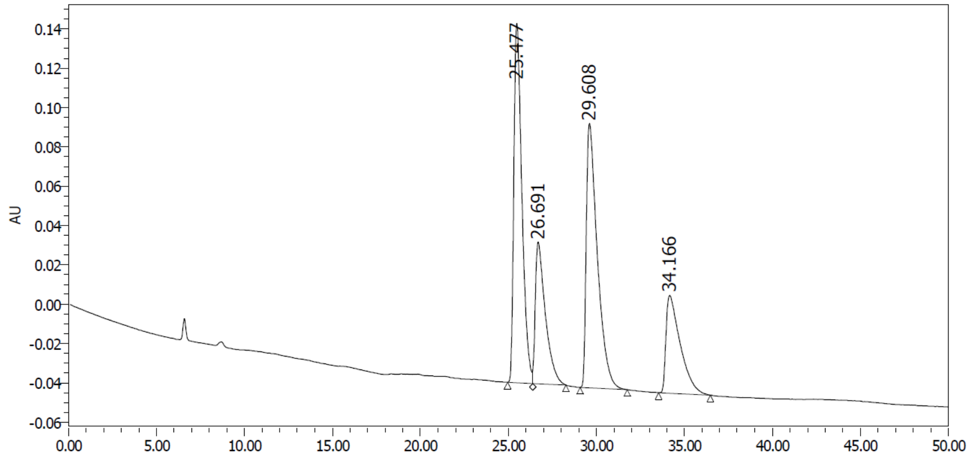

Peak #	Ret. Time	Area	Area %
1	25.477	5828875	33.59
2	26.691	2848133	16.41
3	29.608	5876091	33.86
4	34.166	2798942	16.13

chiral-**3ae**

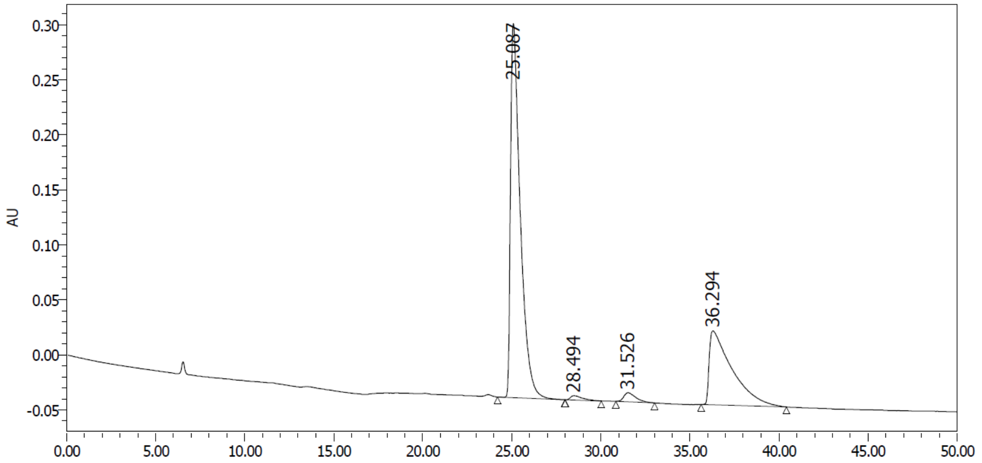

Peak #	Ret. Time	Area	Area %
1	25.087	12936430	66.40
2	28.494	183946	0.94
3	31.526	384104	1.97
4	36.294	5978688	30.69

3bf: The enantiomeric ratio was determined by HPLC analysis in comparison with authentic racemic material (CHIRALCEL OD-H column, 99.8/0.2 hexane/isopropyl alcohol, 0.5 mL/min, major isomers: t_R = 31.4, 44.0 min, minor isomers: t_R = 36.0, 39.5 min, UV detection at 210 nm, 30 °C).

rac-**3bf**

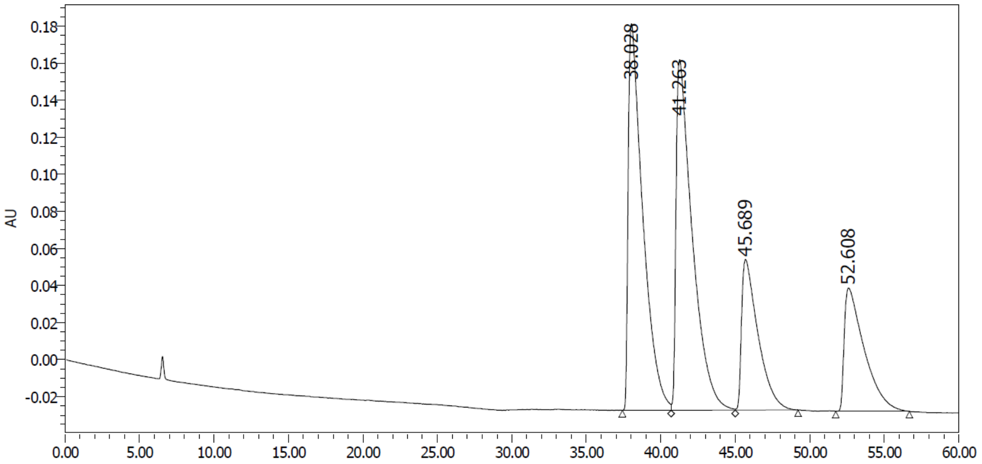

Peak #	Ret. Time	Area	Area %
1	38.028	14256375	34.72
2	41.263	14373976	35.01
3	45.689	6231020	15.18
4	52.608	6199460	15.10

chiral-**3bf**

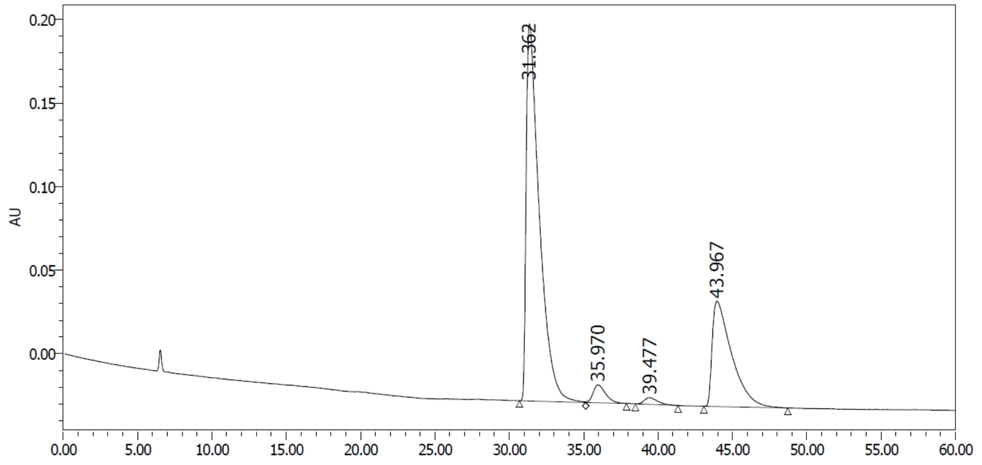

Peak #	Ret. Time	Area	Area %
1	31.362	13892557	67.71
2	35.970	619417	3.02
3	39.477	252043	1.23
4	43.967	5753613	28.04

3ag: The enantiomeric ratio was determined by HPLC analysis in comparison with authentic racemic material (CHIRALCEL OD-H column, 98.8/1.2 hexane/isopropyl alcohol, 0.5 mL/min, major isomers: t_R = 16.0, 17.8 min, minor isomers: t_R = 15.1, 17.0 min, UV detection at 210 nm, 30 °C).

rac-**3ag**

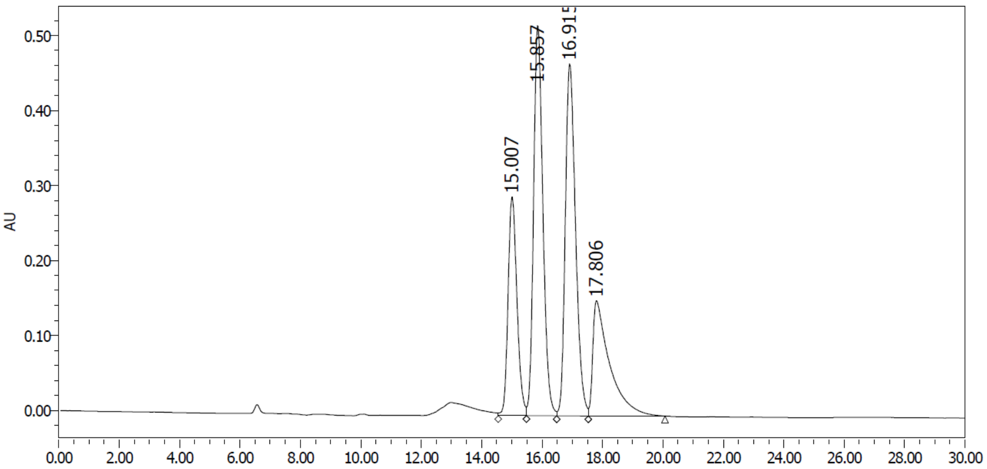

Peak #	Ret. Time	Area	Area %
1	15.007	5959146	17.48
2	15.857	11136417	32.66
3	16.915	11056748	32.43
4	17.806	5940662	17.42

chiral-**3ag**

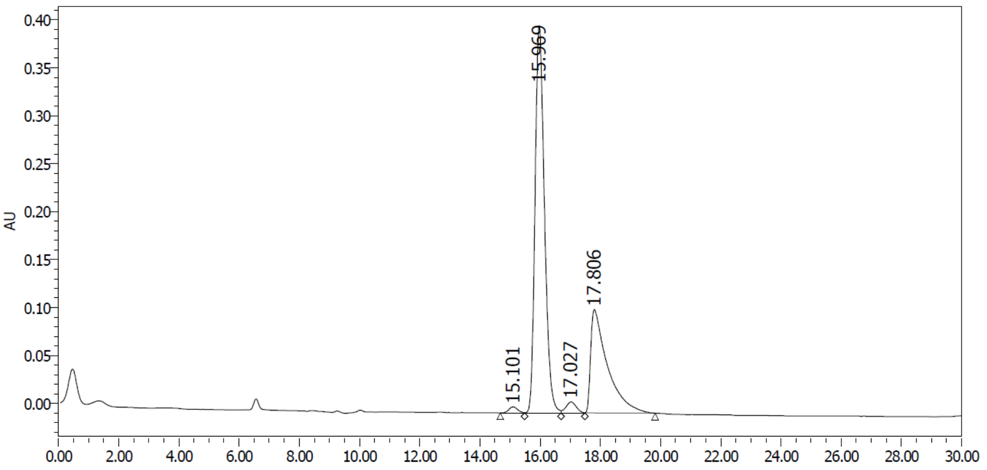

Peak #	Ret. Time	Area	Area %
1	15.101	126479	0.95
2	15.969	8742781	65.65
3	17.027	272077	2.04
4	17.806	4176203	31.36

3ah: The enantiomeric ratio was determined by HPLC analysis in comparison with authentic racemic material (CHIRALPAK AD-H column, 99.8/0.2 hexane/isopropyl alcohol, 0.5 mL/min, major isomers: t_R = 20.2, 34.0 min, minor isomers: t_R = 21.9, 25.4 min, UV detection at 210 nm, 30 °C).

rac-**3ah**

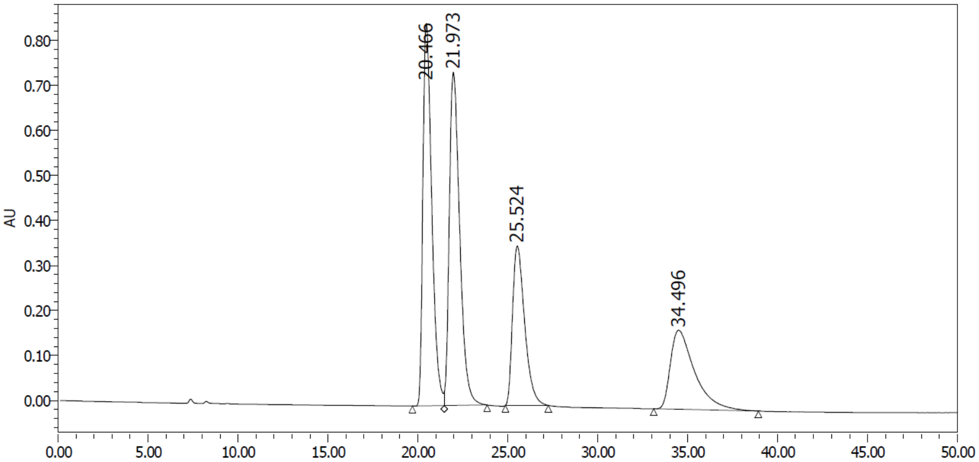

Peak #	Ret. Time	Area	Area %
1	20.466	29269522	31.79
2	21.973	29766128	32.33
3	25.524	16594521	18.03
4	34.496	16432428	17.85

chiral-**3ah**

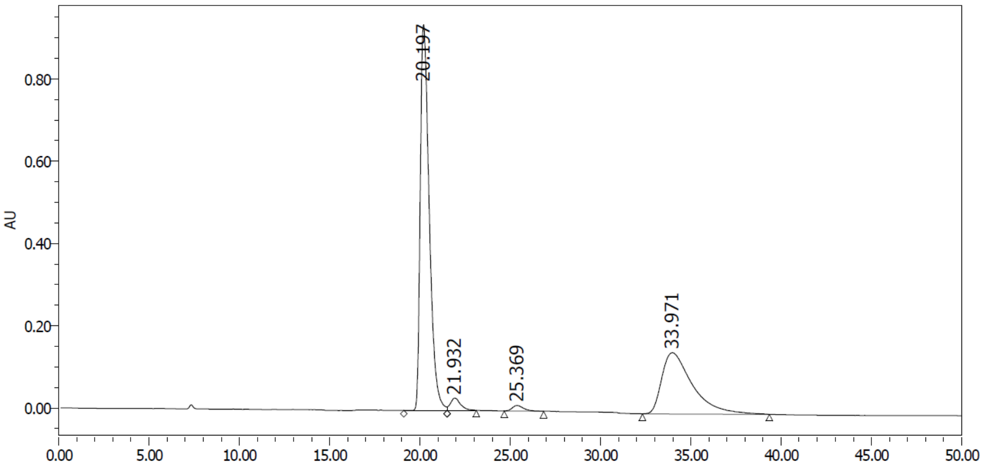

Peak #	Ret. Time	Area	Area %
1	20.197	32413318	62.65
2	21.932	1150130	2.22
3	25.369	588081	1.14
4	33.971	17588274	33.99

3bi: The enantiomeric ratio was determined by HPLC analysis in comparison with authentic racemic material (CHIRALCEL OD-H column, 99.8/0.2 hexane/isopropyl alcohol, 0.5 mL/min, major isomers: t_R = 44.0, 86.9 min, minor isomers: t_R = 41.5, 99.6 min, UV detection at 210 nm, 30 °C).

rac-**3bi**

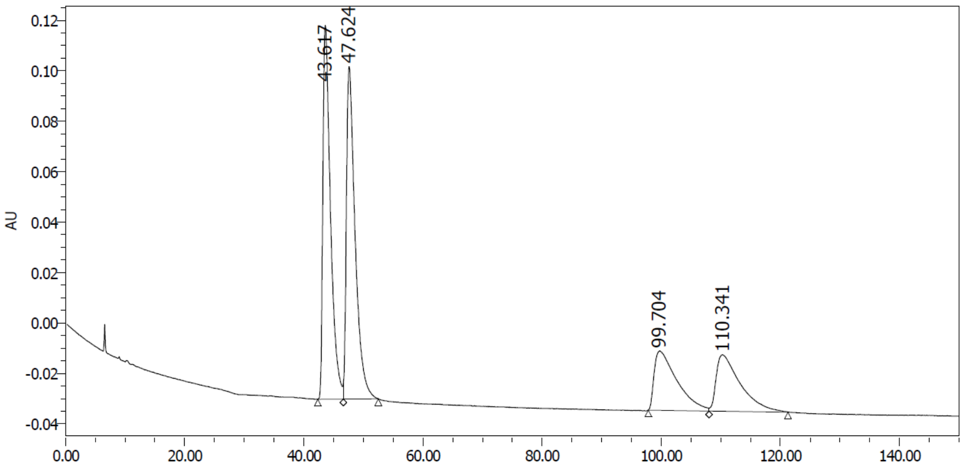

Peak #	Ret. Time	Area	Area %
1	43.617	13241362	33.82
2	47.624	13392873	34.21
3	99.704	6217827	15.88
4	110.341	6295105	16.08

chiral-**3bi**

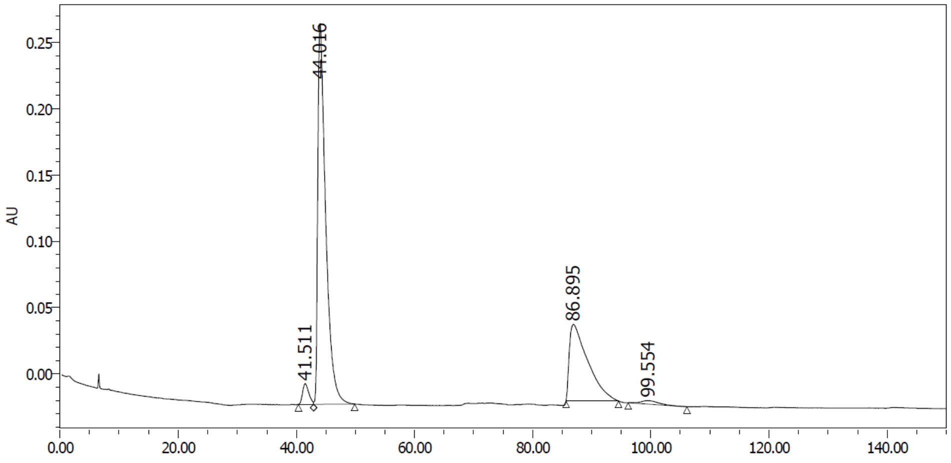

Peak #	Ret. Time	Area	Area %
1	41.511	1148452	2.79
2	44.016	26906838	65.32
3	86.895	12613792	30.62
4	99.554	522917	1.27

***syn*-3bj**: The enantiomeric ratio was determined by HPLC analysis in comparison with authentic racemic material (CHIRAL ART Amylose-SA (3 μm) column, 98/2 hexane/isopropyl alcohol, 0.5 mL/min, major isomers: t_R = 43.2 min, minor isomers: t_R = 48.3 min, UV detection at 240 nm, 30 °C).

rac-***syn*-3bj**

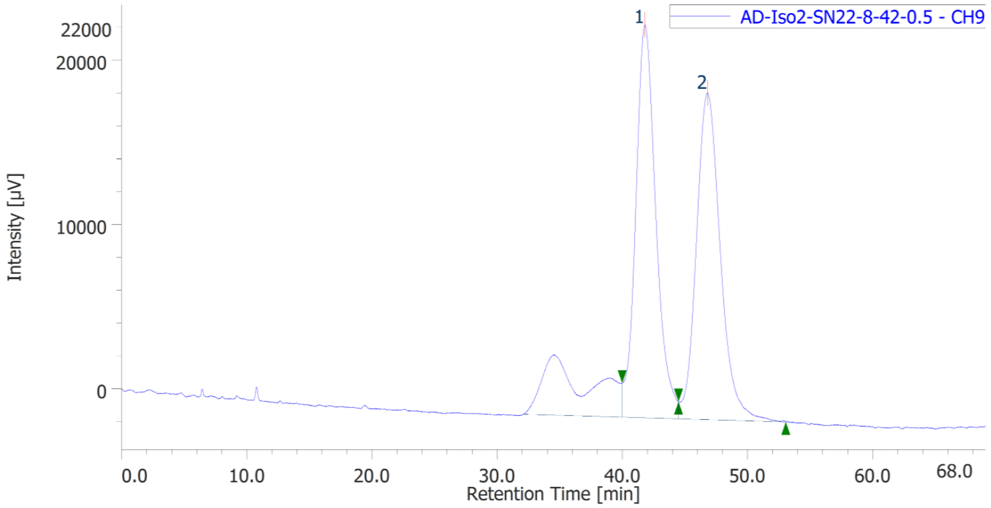

Peak #	Ret. Time	Area	Area %
1	41.797	2679971	50.26
2	46.800	2652760	49.74

chiral-***syn*-3bj**

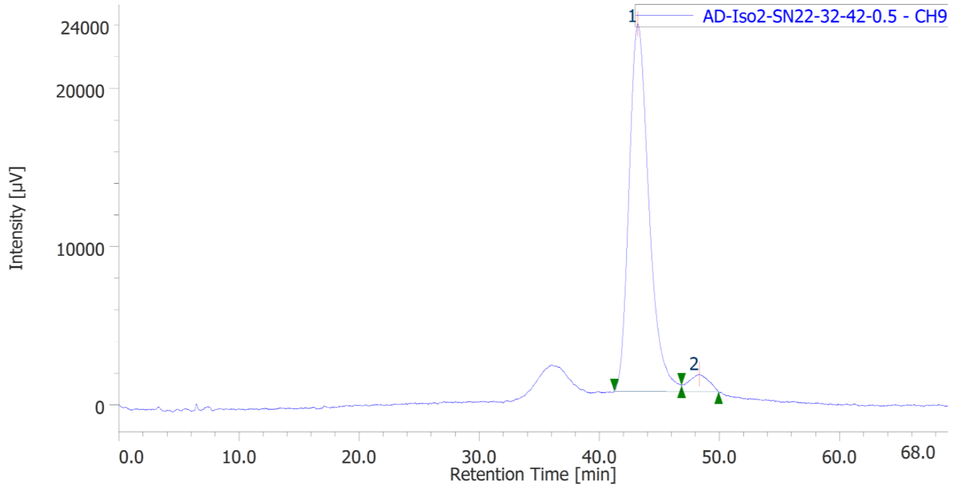

Peak #	Ret. Time	Area	Area %
1	43.183	2669749	95.55
2	48.330	124435	4.45

***anti*-3bj**: The enantiomeric ratio was determined by HPLC analysis in comparison with authentic racemic material (CHIRAL ART Amylose-SA (3 μm) column, 92/8 hexane/isopropyl alcohol, 0.5 mL/min, major isomers: t_R = 17.4 min, minor isomers: t_R = 18.8 min, UV detection at 250 nm, 30 °C).

rac-***anti*-3bj**

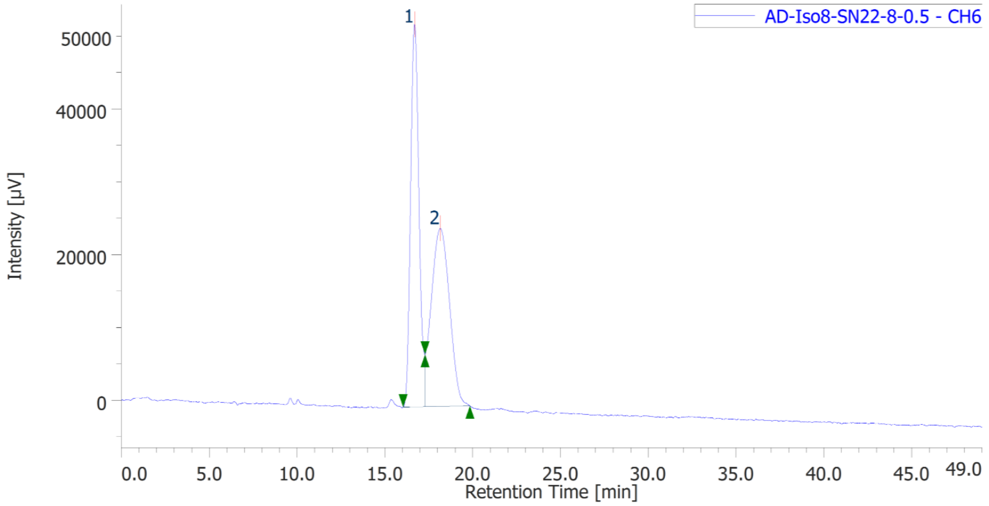

Peak #	Ret. Time	Area	Area %
1	16.683	1738659	49.60
2	18.150	1767086	50.40

chiral-***anti*-3bj**

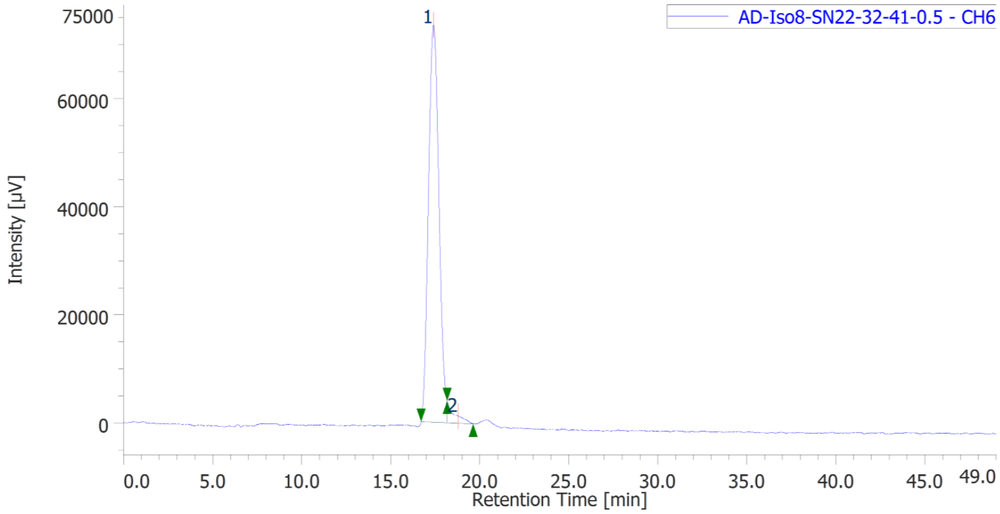

Peak #	Ret. Time	Area	Area %
1	17.403	3002473	96.42
2	18.787	111477	3.58

Characterization Data for Products

^{1}H, ^{13}C{^{1}H}, and ^{19}F{^{1}H} spectra for all compounds are attached in the last part.

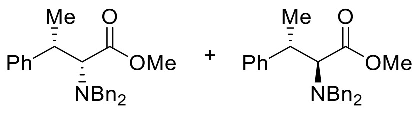

***syn*-3aa** ***anti*-3aa**

A 43:57 diastereomixture of Methyl (2*R*,3*S*)-2-(dibenzylamino)-3-phenylbutanoate (*syn*-3aa) and Methyl (2*S*,3*S*)-2-(dibenzylamino)-3-phenylbutanoate (*anti*-3aa)

It was purified by silica gel column chromatography with hexane/ethyl acetate (20/1, v/v) and GPC ($CHCl_3$): 45.3 mg (81%, 0.15 mmol scale), 265 mg (71%, 1.0 mmol scale); colorless oil; ^{1}H NMR ($CDCl_3$, 400 MHz): δ 7.44 (d, *J* = 7.2 Hz, 0.43 × 4H for ***syn*-3aa**), 7.35 (t, *J* = 7.2 Hz, 0.43 × 4H for ***syn*-3aa**), 7.30-7.25 [(m, 0.43 × 2H for ***syn*-3aa** and 0.57 × 3H for ***anti*-3aa**)], 7.21-7.17 [(m, 0.43 × 2H for ***syn*-3aa** and 0.57 × 6H for ***anti*-3aa**)], 7.15-7.11 (m, 0.43H for ***syn*-3aa**), 7.03-7.00 (m, 0.43 × 2H for ***syn* -3aa**), 6.93-6.86 (m, 0.57 × 6H for ***anti*-3aa**), 4.11 (d, *J* = 13.9 Hz, 0.43 × 2H for ***syn*-3aa**), 3.88 (d, *J* = 13.6 Hz, 0.57 × 2H for ***anti*-3aa**), 3.87 (s, 0.57 × 3H for ***anti*-3aa**), 3.48 (d, *J* = 11.4 Hz, 0.57H for ***anti*-3aa**), 3.46 (s, 0.43 × 3H for ***syn*-3aa**), 3.43 (d, *J* = 11.4 Hz, 0.43H for ***syn*-3aa**), 3.35 (d, *J* = 13.9 Hz, 0.43 × 2H for ***syn*-3aa**), 3.35-3.25 [(m, 0.43H for ***syn*-3aa** and 0.57H for ***anti*-3aa**)], 3.21 (d, *J* = 13.8 Hz, 0.57 × 2H for ***anti*-3aa**), 1.35 (d, *J* = 6.9 Hz, 0.43 × 3H for ***syn*-3aa**), 1.10 (d, *J* = 6.7 Hz, 0.57 × 3H for ***anti*-3aa**); ^{13}C{^{1}H} NMR ($CDCl_3$, 100 MHz): δ 172.4, 171.4, 143.8, 143.6, 139.5, 139.1, 129.2, 129.0, 128.44, 128.39, 128.35, 128.2, 128.0, 127.8, 127.2, 127.0, 126.6, 126.5, 67.4, 66.5, 54.8, 54.4, 51.2, 50.6, 39.5, 39.3, 20.8, 19.3; HRMS (APCI) m/z ([M+H]$^+$) calcd for $C_{25}H_{28}NO_2$: 374.2115, found: 374.2109. CHIRALPAK AD-H column, 98.5/1.5 hexane/isopropyl alcohol, 0.5 mL/min, major isomers: t_R = 10.7, 24.5 min, minor isomers: t_R = 12.8, 14.3 min.

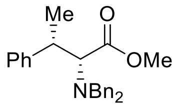

***syn*-3aa**

Methyl (2*R*,3*S*)-2-(dibenzylamino)-3-phenylbutanoate (*syn*-3aa)

It was purified by silica gel column chromatography with hexane/ethyl acetate (20/1, v/v) and GPC ($CHCl_3$ then ethyl acetate): 17.3 mg (31%, 0.15 mmol scale); colorless oil; ^{1}H NMR ($CDCl_3$, 400 MHz): δ 7.44 (d, *J* = 7.2 Hz, 4H), 7.36 (t, *J* = 7.2 Hz, 4H), 7.29-7.26 (m, 2H), 7.21-7.17 (m, 2H), 7.15-7.11 (m, 1H), 7.02-7.00 (m, 2H), 4.11 (d, *J* = 13.9 Hz, 2H), 3.46 (s, 3H), 3.43 (d, *J* = 11.4 Hz, 1H),

3.35 (d, J = 13.9 Hz, 2H), 3.32-3.24 (m, 1H), 1.35 (d, J = 6.9 Hz, 3H); $^{13}C\{^1H\}$ NMR ($CDCl_3$, 100 MHz): δ171.5, 143.8, 139.5, 129.1, 128.5, 128.4, 127.9, 127.2, 126.7, 67.5, 54.9, 50.6, 39.4, 19.3; HRMS (APCI) m/z ($[M+H]^+$) calcd for $C_{25}H_{28}NO_2$: 374.2115, found: 374.2108. CHIRALPAK AD-H column, 98.5/1.5 hexane/isopropyl alcohol, 0.5 mL/min, major isomer: t_R = 23.3 min, minor isomer: t_R = 13.9 min.

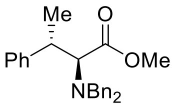

***anti*-3aa**

Methyl (2*S*,3*S*)-2-(dibenzylamino)-3-phenylbutanoate (*anti*-3aa)

It was purified by silica gel column chromatography with hexane/ethyl acetate (20/1, v/v) and GPC ($CHCl_3$ then ethyl acetate): 22.9 mg (41%, 0.15 mmol scale); colorless oil; ^1H NMR ($CDCl_3$, 400 MHz): δ 7.30-7.25 (m, 3H), 7.20-7.17 (m, 6H), 6.93-6.86 (m, 6H), 3.88 (d, J = 13.6 Hz, 2H), 3.87 (s, 3H), 3.48 (d, J = 11.4 Hz, 1H), 3.37-3.29 (m, 1H), 3.21 (d, J = 13.8 Hz, 2H), 1.10 (d, J = 6.7 Hz, 3H); $^{13}C\{^1H\}$ NMR ($CDCl_3$, 100 MHz): δ 172.5, 143.6, 139.1, 129.2, 128.4, 128.3, 128.0, 127.0, 126.5, 66.5, 54.4, 51.1, 39.6, 20.8; HRMS (APCI) m/z ($[M+H]^+$) calcd for $C_{25}H_{28}NO_2$: 374.2115, found: 374.2117. CHIRALPAK AD-H column, 98.5/1.5 hexane/isopropyl alcohol, 0.5 mL/min, major isomer: t_R = 10.6 min, minor isomer: t_R = 12.7 min.

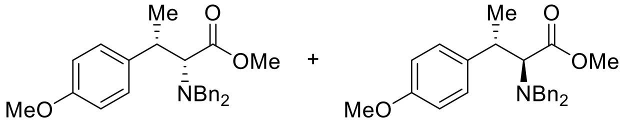

***syn*-3ba** ***anti*-3ba**

A 43:57 diastereomixture of Methyl (2*R*,3*S*)-2-(dibenzylamino)-3-(4-methoxyphenyl)butanoate (*syn*-3ba) and Methyl (2*S*,3*S*)-2-(dibenzylamino)-3-(4-methoxyphenyl)butanoate (*anti*-3ba)

It was purified by silica gel column chromatography with hexane/ethyl acetate (10/1, v/v) and GPC ($CHCl_3$): 54.5 mg (90%, 0.15 mmol scale); colorless oil; ^1H NMR ($CDCl_3$, 400 MHz): δ 7.44 (d, J = 7.2 Hz, 0.43 × 4H for ***syn*-3ba**), 7.35 (t, J = 7.2 Hz, 0.43 × 4H for ***syn*-3ba**), 7.29-7.25 (m, 0.43 × 2H for ***syn*-3ba**), 7.23-7.18 (m, 0.57 × 6H for ***anti*-3ba**), 6.96-6.89 [(m, 0.43 × 2H for ***syn*-3ba** and 0.57 × 4H for ***anti*-3ba**)], 6.83 (s, 0.57 × 4H for ***anti*-3ba**), 6.74 (d, J = 8.7 Hz, 0.43 × 2H for ***syn*-3ba**), 4.10 (d, J = 13.9 Hz, 0.43 × 2H for ***syn*-3ba**), 3.88 (d, J = 13.8 Hz, 0.57 × 2H for ***anti*-3ba**), 3.87 (s, 0.57 × 3H for ***anti*-3ba**), 3.86 (s, 0.57 × 3H for ***anti*-3ba**), 3.74 (s, 0.43 × 3H for ***syn*-3ba**), 3.48 (s, 0.43 × 3H for

***syn*-3ba**), 3.43 (d, J = 11.4 Hz, 0.57H for ***anti*-3ba**), 3.38 (d, J = 11.4 Hz, 0.43H for ***syn*-3ba**), 3.33 (d, J = 13.9 Hz, 0.43 × 2H for ***syn*-3ba**), 3.32-3.21 [(m, 0.43H for ***syn*-3ba** and 0.57H for ***anti*-3ba**)], 3.21 (d, J = 13.8 Hz, 0.57 × 2H for ***anti*-3ba**), 1.32 (d, J = 6.9 Hz, 0.43 × 3H for ***syn*-3ba**), 1.07 (d, J = 6.7 Hz, 0.57 × 3H for ***anti*-3ba**); $^{13}C\{^{1}H\}$ NMR ($CDCl_3$, 100 MHz): δ 172.6, 171.6, 158.4, 158.2, 139.5, 139.2, 135.9, 135.8, 129.2 (2C), 129.0, 128.8, 128.5, 128.1, 127.2, 127.0, 113.8, 113.7, 67.7, 66.7, 55.6, 55.3, 54.8, 54.4, 51.1, 50.7, 38.7, 38.4, 20.9, 19.4; HRMS (APCI) m/z ([M+H]$^+$) calcd for $C_{26}H_{30}NO_3$: 404.2220, found: 404.2229. CHIRALPAK AD-H column, 99/1 hexane/isopropyl alcohol, 0.5 mL/min, major isomers: t_R = 11.7, 26.0 min, minor isomers: t_R = 13.4, 15.8 min.

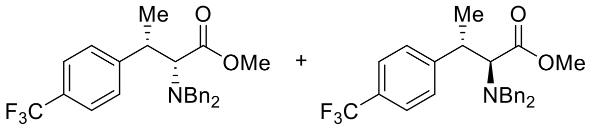

***syn*-3ca** ***anti*-3ca**

A 45:55 diastereomixture of Methyl (2*R*,3*S*)-2-(dibenzylamino)-3-(4-(trifluoromethyl)phenyl)butanoate (*syn*-3ca) and Methyl (2*S*,3*S*)-2-(dibenzylamino)-3-(4-(trifluoromethyl)phenyl)butanoate (*anti*-3ca)

It was purified by silica gel column chromatography with hexane/ethyl acetate (20/1, v/v) and GPC ($CHCl_3$): 47.0 mg (71%, 0.15 mmol scale); colorless oil; ^{1}H NMR ($CDCl_3$, 400 MHz): δ 7.50 (d, J = 8.0 Hz, 0.55 × 2H for ***anti*-3ca**), 7.46-7.42 (m, 0.45 × 6H for ***syn*-3ca**), 7.37 (t, J = 7.2 Hz, 0.45 × 4H for ***syn*-3ca**), 7.31-7.27 (m, 0.45 × 2H for ***syn*-3ca**), 7.22-7.17 (m, 0.55 × 6H for ***anti*-3ca**), 7.12 (d, J = 8.1 Hz, 0.45 × 2H for ***syn*-3ca**), 6.97 (d, J = 8.0 Hz, 0.55 × 2H for ***anti*-3ca**), 6.85-6.81 (m, 0.55 × 4H for ***anti*-3ca**), 4.09 (d, J = 13.7 Hz, 0.45 × 2H for ***syn*-3ca**), 3.89 (s, 0.55 × 3H for ***anti*-3ca**), 3.84 (d, J = 13.8 Hz, 0.55 × 2H for ***anti*-3ca**), 3.502 (d, J = 11.4 Hz, 0.55H for ***anti*-3ca**), 3.496 (s, 0.45 × 3H for ***syn*-3ca**), 3.43 (d, J = 11.4 Hz, 0.45H for ***syn*-3ca**), 3.43-3.32 [(m, 0.45H for ***syn*-3ca** and 0.55H for ***anti*-3ca**)], 3.34 (d, J = 13.8 Hz, 0.45 × 2H for ***syn*-3ca**), 3.20 (d, J = 13.8 Hz, 0.55 × 2H for ***anti*-3ca**), 1.33 (d, J = 6.7 Hz, 0.45 × 3H for ***syn*-3ca**), 1.10 (d, J = 6.7 Hz, 0.55 × 3H for ***anti*-3ca**); $^{13}C\{^{1}H\}$ NMR ($CDCl_3$, 100 MHz): δ 172.0, 171.1, 148.1, 147.9, 139.2, 138.7, 129.2, 129.1, 128.8 (q, J = 32.1 Hz), 128.7, 128.5, 128.22, 128.15, 127.3, 127.2, 125.4 (q, J = 3.8 Hz), 125.1 (q, J = 3.7 Hz), 124.6 (q, J = 270.0 Hz), 124.3 (q, J = 270.4 Hz), 67.0, 66.1, 54.9, 54.4, 51.3, 50.8, 39.5, 39.2, 20.5, 19.2 (One sp^2 C signal overlaps with others.); $^{19}F\{^{1}H\}$ NMR ($CDCl_3$, 376 MHz): δ −62.19 (s), −62.46 (s); HRMS (APCI) m/z ([M+H]$^+$) calcd for $C_{26}H_{27}F_3NO_2$: 442.1988, found: 442.2002. CHIRALPAK AD-H column, 99/1 hexane/isopropyl alcohol, 0.5 mL/min, major isomers: t_R = 8.5, 17.8 min, minor isomers:

t_R = 9.8, 11.6 min.

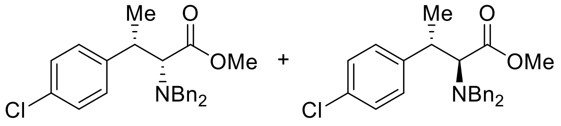

A 47:53 diastereomixture of Methyl (2*R*,3*S*)-3-(4-chlorophenyl)-2-(dibenzylamino)butanoate (*syn*-3da) and Methyl (2*S*,3*S*)-3-(4-chlorophenyl)-2-(dibenzylamino)butanoate (*anti*-3da)

It was purified by silica gel column chromatography with hexane/ethyl acetate (20/1, v/v) and GPC ($CHCl_3$): 52.0 mg (85%, 0.15 mmol scale); colorless oil; ^{1}H NMR ($CDCl_3$, 400 MHz): δ 7.42 (d, *J* = 7.3 Hz, 0.47 × 4H for ***syn*-3da**), 7.35 (t, *J* = 7.2 Hz, 0.47 × 4H for ***syn*-3da**), 7.29-7.27 (m, 0.47 × 2H for ***syn*-3da**), 7.24-7.21 (m, 0.53 × 8H for ***anti*-3da**), 7.16 (d, *J* = 8.5 Hz, 0.47 × 2H for ***syn*-3da**), 6.94 (d, *J* = 8.5 Hz, 0.47 × 2H for ***syn*-3da**), 6.91-6.88 (m, 0.53 × 4H for ***anti*-3da**), 6.80 (d, *J* = 8.4 Hz, 0.53 × 2H for ***anti*-3da**), 4.08 (d, *J* = 13.8 Hz, 0.47 × 2H for ***syn*-3da**), 3.87 (s, 0.53 × 3H for ***anti*-3da**), 3.86 (d, *J* = 13.8 Hz, 0.53 × 2H for ***anti*-3da**), 3.50 (s, 0.47 × 3H for ***syn*-3da**), 3.44 (d, *J* = 11.4 Hz, 0.53H for ***anti*-3da**), 3.37 (d, *J* = 11.4 Hz, 0.47H for ***syn*-3da**), 3.32 (d, *J* = 13.9 Hz, 0.47 × 2H for ***syn*-3da**), 3.32-3.23 [(m, 0.47H for ***syn*-3da** and 0.53H for ***anti*-3da**)], 3.20 (d, *J* = 13.8 Hz, 0.53 × 2H for ***anti*-3da**), 1.30 (d, *J* = 6.8 Hz, 0.47 × 3H for ***syn*-3da**), 1.06 (d, *J* = 6.7 Hz, 0.53 × 3H for ***anti*-3da**); ^{13}C{^{1}H} NMR ($CDCl_3$, 100 MHz): δ 172.1, 171.2, 142.4, 142.3, 139.3, 138.9, 132.3, 132.0, 129.7, 129.23, 129.17, 129.05, 128.6, 128.5, 128.3, 128.2, 127.3, 127.2, 67.3, 66.3, 54.9, 54.4, 51.2, 50.8, 39.0, 38.7, 20.7, 19.2; HRMS (APCI) m/z ([M+H]$^+$) calcd for $C_{25}H_{27}ClNO_2$: 408.1725, found: 408.1713. CHIRALPAK AD-H column, 99/1 hexane/isopropyl alcohol, 0.5 mL/min, major isomers: t_R = 9.0, 15.8 min, minor isomers: t_R = 10.6, 11.4 min.

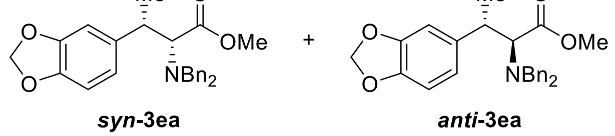

A 45:55 diastereomixture of Methyl (2*S*,3*R*)-3-(benzo[*d*][1,3]dioxol-5-yl)-2-(dibenzylamino)butanoate (*syn*-3ea) and Methyl (2*R*,3*R*)-3-(benzo[*d*][1,3]dioxol-5-yl)-2-(dibenzylamino)butanoate (*anti*-3ea)

It was purified by silica gel column chromatography with hexane/ethyl acetate (10/1, v/v) and GPC

($CHCl_3$): 50.1 mg (80%, 0.15 mmol scale); colorless oil; ^{1}H NMR ($CDCl_3$, 400 MHz): δ 7.43 (d, *J* = 7.3 Hz, 0.45 × 4H for ***syn*-3ea**), 7.35 (t, *J* = 7.2 Hz, 0.45 × 4H for ***syn*-3ea**), 7.29-7.25 (m, 0.45 × 2H for ***syn*-3ea**), 7.24-7.18 (m, 0.55 × 6H for ***anti*-3ea**), 6.98-6.96 (m, 0.55 × 4H for ***anti*-3ea**), 6.73 (d, *J* = 7.9 Hz, 0.55H for ***anti*-3ea**), 6.64 (d, *J* = 8.4 Hz, 0.45H for ***syn*-3ea**), 6.50-6.48 (m, 0.45 × 2H for ***syn*-3ea**), 6.45 (d, *J* = 7.9 Hz, 0.55H for ***anti*-3ea**), 6.28 (s, 0.55H for ***anti*-3ea**), 6.03 (d, *J* = 1.4 Hz, 0.55H for ***anti*-3ea**), 5.96 (d, *J* = 1.4 Hz, 0.55H for ***anti*-3ea**), 5.88 (s, 0.45 × 2H for ***syn*-3ea**), 4.07 (d, *J* = 13.9 Hz, 0.45 × 2H for ***syn*-3ea**), 3.88 (d, *J* = 13.8 Hz, 0.55 × 2H for ***anti*-3ea**), 3.86 (s, 0.55 × 3H for ***anti*-3ea**), 3.53 (s, 0.45 × 3H for ***syn*-3ea**), 3.39-3.33 [(m, 0.45H for ***syn*-3ea** and 0.55H for ***anti*-3ea**)], 3.30-3.19 [(m, 0.45H for ***syn*-3ea** and 0.55H for ***anti*-3ea**)], 3.32 (d, *J* = 13.9 Hz, 0.45 × 2H for ***syn*-3ea**), 3.21 (d, *J* = 13.8 Hz, 0.55 × 2H for ***anti*-3ea**), 1.30 (d, *J* = 6.8 Hz, 0.45 × 3H for ***syn*-3ea**), 1.05 (d, *J* = 6.6 Hz, 0.55 × 3H for ***anti*-3ea**); ^{13}C{^{1}H} NMR ($CDCl_3$, 100 MHz): δ 172.3, 171.4, 147.6, 147.5, 146.11, 146.08, 139.4, 139.1, 137.74, 137.69, 129.2, 129.0, 128.5, 128.1, 127.2, 127.1, 121.6, 121.1, 108.4, 108.2, 108.0, 107.9, 100.94, 100.90, 67.5, 66.6, 54.8, 54.5, 51.1, 50.7, 39.2, 39.0, 20.8, 19.4; HRMS (APCI) m/z ([M+H]$^+$) calcd for $C_{26}H_{28}NO_4$: 418.2013, found: 418.2014. CHIRALPAK AD-H column, 98.5/1.5 hexane/isopropyl alcohol, 0.5 mL/min, major isomers: t_R = 15.9, 36.3 min, minor isomers: t_R = 21.8, 22.8 min.

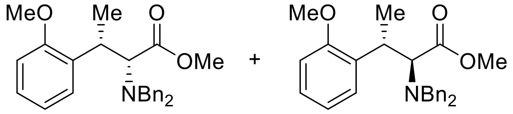

***syn*-3fa** ***anti*-3fa**

A 37:63 diastereomixture of Methyl (2*R*,3*S*)-2-(dibenzylamino)-3-(2-methoxyphenyl)butanoate (*syn*-3fa) and Methyl (2*S*,3*S*)-2-(dibenzylamino)-3-(2-methoxyphenyl)butanoate (*anti*-3fa)

It was purified by silica gel column chromatography with hexane/ethyl acetate (10/1, v/v) and GPC ($CHCl_3$): 12.1 mg (20%, 0.15 mmol scale); colorless oil; ^{1}H NMR ($CDCl_3$, 400 MHz): δ 7.46 (d, *J* = 7.3 Hz, 0.37 × 4H for ***syn*-3fa**), 7.35 (t, *J* = 7.2 Hz, 0.37 × 4H for ***syn*-3fa**), 7.29-7.24 [(m, 0.37 × 2H for ***syn*-3fa** and 0.63H for ***anti*-3fa**)], 7.21-7.16 (m, 0.63 × 6H for ***anti*-3fa**), 7.12-7.08 (m, 0.37H for ***syn*-3fa**), 6.93-6.87 [(m, 0.37H for ***syn*-3fa** and 0.63 × 5H for ***anti*-3fa**)], 6.81-6.75 [(m, 0.37 × 2H for ***syn*-3fa** and 0.63 × 2H for ***anti*-3fa**], 4.12 (d, *J* = 13.9 Hz, 0.37 × 2H for ***syn*-3fa**), 3.92 (d, *J* = 13.8 Hz, 0.63 × 2H for ***anti*-3fa**), 3.94-3.84 (m, 0.63H for ***anti*-3fa**), 3.86 (s, 0.63 × 3H for ***anti*-3fa**), 3.78 (d, *J* = 11.4 Hz, 0.37H for ***syn*-3fa**), 3.67-3.58 [(m, 0.37H for ***syn*-3fa** and 0.63H for ***anti*-3fa**)], 3.62 (s, 0.37 × 3H for ***syn*-3fa**), 3.47 (s, 0.37 × 3H for ***syn*-3fa**), 3.45 (s, 0.63 × 3H for ***anti*-3fa**), 3.36 (d, *J* = 13.9 Hz,

0.37 × 2H for ***syn*-3fa**), 3.19 (d, J = 13.8 Hz, 0.63 × 2H for ***anti*-3fa**), 1.30 (d, J = 6.9 Hz, 0.37 × 3H for ***syn*-3fa**), 1.08 (d, J = 6.8 Hz, 0.63 × 3H for ***anti*-3fa**); $^{13}C\{^1H\}$ NMR ($CDCl_3$, 100 MHz): δ 172.9, 171.8, 157.5, 157.4, 139.8, 139.6, 132.2, 131.7, 129.2, 129.1 (2C), 128.6, 128.4, 128.0, 127.5, 127.2, 127.1, 126.8, 120.6, 120.4, 111.1, 110.3, 66.4, 65.0, 55.4, 54.9 (2C), 54.6, 51.0, 50.6, 34.0, 19.1, 18.1 (One sp^3 C signal merges with the other.); HRMS (APCI) m/z ($[M+H]^+$) calcd for $C_{26}H_{30}NO_3$: 404.2220, found: 404.2222. CHIRALPAK AD-H column, 99/1 hexane/isopropyl alcohol, 0.5 mL/min, major isomers: t_R = 12.7, 23.0 min, minor isomers: t_R = 16.5, 17.8 min.

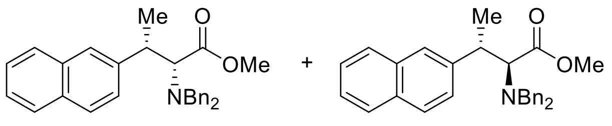

***syn*-3ga** ***anti*-3ga**

A 38:62 diastereomixture of Methyl (2*R*,3*S*)-2-(dibenzylamino)-3-(naphthalen-2-yl)butanoate (*syn*-3ga) and Methyl (2*S*,3*S*)-2-(dibenzylamino)-3-(naphthalen-2-yl)butanoate (*anti*-3ga)

It was purified by silica gel column chromatography with hexane/ethyl acetate (20/1, v/v) and GPC ($CHCl_3$): 54.0 mg (85%, 0.15 mmol scale); colorless oil; ^1H NMR ($CDCl_3$, 400 MHz): δ 7.90-7.88 (m, 0.62H for ***anti*-3ga**), 7.77-7.66 [(m, 0.38 × 3H for ***syn*-3ga** and 0.62 × 2H for ***anti*-3ga**)], 7.50-7.36 [(m, 0.38 × 11H for ***syn*-3ga** and 0.62 × 3H for ***anti*-3ga**)], 7.32-7.28 (m, 0.38 × 2H for ***syn*-3ga**), 7.16-7.13 [(m, 0.38H for ***syn*-3ga** and 0.62 × 2H for ***anti*-3ga**)], 7.08 (t, J = 7.0 Hz, 0.62 × 4H for ***anti*-3ga**), 7.01 (d, J = 8.4 Hz, 0.62H for ***anti*-3ga**), 6.81 (d, J = 7.2 Hz, 0.62 × 4H for ***anti*-3ga**), 4.15 (d, J = 13.9 Hz, 0.38 × 2H for ***syn*-3ga**), 3.91 (d, J = 13.8 Hz, 0.62 × 2H for ***anti*-3ga**), 3.90 (s, 0.62 × 3H for ***anti*-3ga**), 3.61 (d, J = 11.4 Hz, 0.62H for ***anti*-3ga**), 3.57 (d, J = 11.3 Hz, 0.38H for ***syn*-3ga**), 3.53-3.44 [(m, 0.38H for ***syn*-3ga** and 0.62H for ***anti*-3ga**)], 3.40 (s, 0.38 × 3H for ***syn*-3ga**), 3.38 (d, J = 13.9 Hz, 0.38 × 2H for ***syn*-3ga**), 3.23 (d, J = 13.8 Hz, 0.62 × 2H for ***anti*-3ga**), 1.42 (d, J = 6.8 Hz, 0.38 × 3H for ***syn*-3ga**), 1.18 (d, J = 6.6 Hz, 0.62 × 3H for ***anti*-3ga**); $^{13}C\{^1H\}$ NMR ($CDCl_3$, 100 MHz): δ 172.4, 171.4, 141.4, 141.2, 139.5, 139.0, 133.6, 133.5, 132.6, 132.5, 129.2, 129.1, 128.5, 128.1 (2C), 127.83 (2C), 127.78, 127.71, 127.65, 127.2, 127.0, 126.9, 126.8, 126.5, 126.1, 125.9, 125.90, 125.5, 125.4, 67.3, 66.5, 54.9, 54.5, 51.2, 50.7, 39.7, 39.5, 20.7, 19.4; HRMS (APCI) m/z ($[M+H]^+$) calcd for $C_{29}H_{30}NO_2$: 424.2271, found: 424.2274. CHIRALPAK AD-H column, 98.5/1.5 hexane/isopropyl alcohol, 0.5 mL/min, major isomers: t_R = 13.1, 34.9 min, minor isomers: t_R = 15.1, 21.6 min.

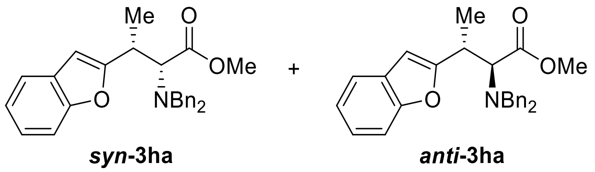

A 36:64 diastereomixture of Methyl (2*R*,3*R*)-3-(benzofuran-2-yl)-2-(dibenzylamino)butanoate (*syn*-3ha) and Methyl (2*S*,3*R*)-3-(benzofuran-2-yl)-2-(dibenzylamino)butanoate (*anti*-3ha)

It was purified by silica gel column chromatography with hexane/ethyl acetate (10/1, v/v) and GPC ($CHCl_3$): 40.3 mg (65%, 0.15 mmol scale); colorless oil; ^{1}H NMR ($CDCl_3$, 400 MHz): δ 7.55-7.53 (m, 0.64H for ***anti*-3ha**), 7.44-7.42 (m, 0.36 × 5H for ***syn*-3ha**), 7.39-7.24 [(m, 0.36 × 7H for ***syn*-3ha** and 0.64 × 3H for ***anti*-3ha**)], 7.20-7.12 [(m, 0.36 × 2H for ***syn*-3ha** and 0.64 × 6H for ***anti*-3ha**)], 7.03-7.01 (m, 0.64 × 4H for ***anti*-3ha**), 6.34 (s, 0.64H for ***anti*-3ha**), 6.30 (s, 0.36H for ***syn*-3ha**), 4.07 (d, *J* = 14.0 Hz, 0.36 × 2H for ***syn*-3ha**), 3.94 (d, *J* = 13.8 Hz, 0.64 × 2H for ***anti*-3ha**), 3.87 (s, 0.64 × 3H for ***anti*-3ha**), 3.66-3.55 [(m, 0.36H × 2H for ***syn*-3ha** and 0.64H × 2H for ***anti*-3ha**)], 3.62 (s, 0.36 × 3H for ***syn*-3ha**), 3.41 (d, *J* = 14.0 Hz, 0.36 × 2H for ***syn*-3ha**), 3.28 (d, *J* = 13.9 Hz, 0.64 × 2H for ***anti*-3ha**), 1.44 (d, *J* = 6.5 Hz, 0.36 × 3H for ***syn*-3ha**), 1.21 (d, *J* = 6.3 Hz, 0.64 × 3H for ***anti*-3ha**); ^{13}C{^{1}H} NMR ($CDCl_3$, 100 MHz): δ 171.44, 171.36, 160.3, 159.9, 154.61, 154.56, 139.1, 139.0, 129.01, 128.98, 128.88, 128.6, 128.5, 128.1, 127.3, 127.0, 123.5, 123.4, 122.6, 122.5, 120.6, 120.5, 111.2, 111.0, 103.2, 102.2, 65.5, 65.0, 54.8, 54.7, 51.3, 51.1, 33.70, 33.66, 17.3, 16.7; HRMS (APCI) m/z ([M+H]$^+$) calcd for $C_{27}H_{28}NO_3$: 414.2064, found: 414.2067. CHIRALPAK AD-H column, 98.5/1.5 hexane/isopropyl alcohol, 0.5 mL/min, major isomers: t_R = 12.7, 36.1 min, minor isomers: t_R = 15.3, 21.5 min.

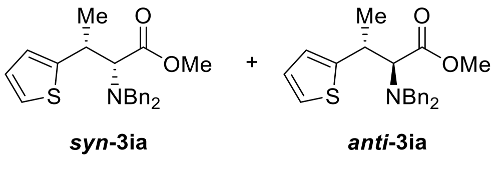

A 45:55 diastereomixture of Methyl (2*R*,3*R*)-2-(dibenzylamino)-3-(thiophen-2-yl)butanoate (*syn*-3ia) and Methyl (2*S*,3*R*)-2-(dibenzylamino)-3-(thiophen-2-yl)butanoate (*anti*-3ia)

It was purified by silica gel column chromatography with hexane/ethyl acetate (20/1, v/v) and GPC ($CHCl_3$): 46.7 mg (82%, 0.15 mmol scale); colorless oil; ^{1}H NMR ($CDCl_3$, 400 MHz): δ 7.42 (d, *J* = 7.2 Hz, 0.45 × 4H for ***syn*-3ia**), 7.35 (t, *J* = 7.2 Hz, 0.45 × 4H for ***syn*-3ia**), 7.29-7.25 (m, 0.45 × 2H for ***syn*-3ia**), 7.24-7.17 (m, 0.55 × 7H for ***anti*-3ia**), 7.06 (d, *J* = 5.1 Hz, 0.45H for ***syn*-3ia**), 7.04-7.01 (m,

0.55 × 4H for ***anti*-3ia**), 6.95 (dd, *J* = 3.4, 5.1 Hz, 0.55H for ***anti*-3ia**), 6.83 (dd, *J* = 3.5, 5.1 Hz, 0.45H for ***syn*-3ia**), 6.69 (d, *J* = 3.6 Hz, 0.45H for ***syn*-3ia**), 6.65 (d, *J* = 3.5 Hz, 0.55H for ***anti*-3ia**), 4.06 (d, *J* = 13.9 Hz, 0.45 × 2H for ***syn*-3ia**), 3.93 (d, *J* = 13.8 Hz, 0.55 × 2H for ***anti*-3ia**), 3.84 (s, 0.55 × 3H for ***anti*-3ia**), 3.72-3.62 [(m, 0.45H for ***syn*-3ia** and 0.55H for ***anti*-3ia**)], 3.59 (s, 0.45 × 3H for ***syn*-3ia**), 3.42 (d, *J* = 11.1 Hz, 0.55H for ***anti*-3ia**), 3.38 (d, *J* = 11.2 Hz, 0.45H for ***syn*-3ia**), 3.33 (d, *J* = 13.9 Hz, 0.45 × 2H for ***syn*-3ia**), 3.26 (d, *J* = 13.8 Hz, 0.55 × 2H for ***anti*-3ia**), 1.42 (d, *J* = 6.9 Hz, 0.45 × 3H for ***syn*-3ia**), 1.19 (d, *J* = 6.8 Hz, 0.55 × 3H for ***anti*-3ia**); $^{13}C\{^1H\}$ NMR ($CDCl_3$, 100 MHz): δ 172.0, 171.3, 147.4, 147.2, 139.2, 139.1, 129.2, 129.0, 128.5, 128.1, 127.3, 127.0, 126.6, 126.3, 124.8, 124.3, 123.4, 123.1, 68.3, 67.9, 54.8, 54.6, 51.2, 50.9, 35.0, 34.7, 21.7, 20.4; HRMS (APCI) m/z ($[M+H]^+$) calcd for $C_{23}H_{26}NO_2S$: 380.1679, found: 380.1675. CHIRALCEL OD-H column, 99.5/0.5 hexane/isopropyl alcohol, 0.5 mL/min, major isomers: t_R = 11.8, 19.3 min, minor isomers: t_R = 13.3, 13.8 min.

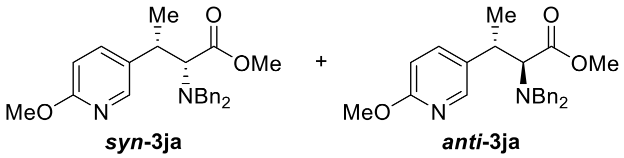

A 47:53 diastereomixture of Methyl (2*R*,3*S*)-2-(dibenzylamino)-3-(6-methoxypyridin-3-yl)butanoate (*syn*-3ja) and Methyl (2*S*,3*S*)-2-(dibenzylamino)-3-(6-methoxypyridin-3-yl)butanoate (*anti*-3ja)

It was purified by silica gel column chromatography with hexane/ethyl acetate (4/1, v/v) and GPC ($CHCl_3$): 49.8 mg (82%, 0.15 mmol scale); colorless oil; ^1H NMR ($CDCl_3$, 400 MHz): δ 7.84-7.83 [(m, 0.47H for ***syn*-3ja** and 0.53H for ***anti*-3ja**)], 7.42 (d, *J* = 7.3 Hz, 0.47 × 4H for ***syn*-3ja**), 7.35 (t, *J* = 7.2 Hz, 0.47 × 4H for ***syn*-3ja**), 7.29-7.25 (m, 0.47 × 2H for ***syn*-3ja**), 7.24-7.19 [(m, 0.47H for ***syn*-3ja** and 0.53 × 6H for ***anti*-3ja**)], 6.92-6.89 (m, 0.53 × 5H for ***anti*-3ja**), 6.67 (d, *J* = 8.5 Hz, 0.53H for ***anti*-3ja**), 6.58 (d, *J* = 8.5 Hz, 0.47H for ***syn*-3ja**), 4.08 (d, *J* = 13.8 Hz, 0.47 × 2H for ***syn*-3ja**), 3.98 (s, 0.53 × 3H for ***anti*-3ja**), 3.874 (s, 0.53 × 3H for ***anti*-3ja**), 3.868 (s, 0.47 × 3H for ***syn*-3ja**), 3.86 (d, *J* = 13.4 Hz, 0.53 × 2H for ***anti*-3ja**), 3.53 (s, 0.47 × 3H for ***syn*-3ja**), 3.40 (d, *J* = 11.4 Hz, 0.53H for ***anti*-3ja**), 3.36 (d, *J* = 11.4 Hz, 0.47H for ***syn*-3ja**), 3.32 (d, *J* = 13.9 Hz, 0.47 × 2H for ***syn*-3ja**), 3.32-3.20 [(m, 0.47H for ***syn*-3ja** and 0.53H for ***anti*-3ja**)], 3.20 (d, *J* = 13.7 Hz, 0.53 × 2H for ***anti*-3ja**), 1.30 (d, *J* = 6.9 Hz, 0.47 × 3H for ***syn*-3ja**), 1.06 (d, *J* = 6.6 Hz, 0.53 × 3H for ***anti*-3ja**); $^{13}C\{^1H\}$ NMR ($CDCl_3$, 100 MHz): δ 172.1, 171.2, 163.2, 163.1, 146.23, 146.19, 139.2, 138.8, 138.14, 138.10, 131.84, 131.80, 129.2,

129.1, 128.5, 128.2, 127.3, 127.2, 110.6, 110.5, 67.2, 66.1, 54.8, 54.4, 53.6, 53.4, 51.2, 50.8, 36.1, 36.9, 20.6, 19.2; HRMS (APCI) m/z ([M+H]$^+$) calcd for $C_{25}H_{29}N_2O_3$: 405.2173, found: 404.2175. CHIRAL ART Cellulose-SB (3 μm) column, 90/10 hexane/chloroform, 0.2 mL/min, major isomers: t_R = 39.0, 79.8 min, minor isomers: t_R = 44.7, 49.1 min.

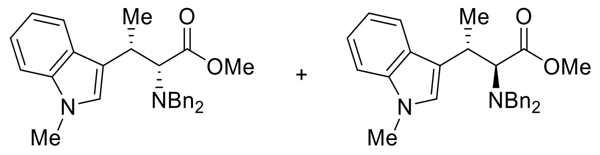

***syn*-3ka** ***anti*-3ka**

A 36:64 diastereomixture of Methyl (2*R*,3*S*)-2-(dibenzylamino)-3-(1-methyl-1*H*-indol-3-yl)butanoate (*syn*-3ka) and Methyl (2*S*,3*S*)-2-(dibenzylamino)-3-(1-methyl-1*H*-indol-3-yl)butanoate (*anti*-3ka)

It was purified by silica gel column chromatography with hexane/ethyl acetate (20/1, v/v) and GPC ($CHCl_3$): 35.2 mg (55%, 0.15 mmol scale); colorless oil; ^{1}H NMR ($CDCl_3$, 400 MHz): δ 7.48 (d, *J* = 7.4 Hz, 0.36 × 4H for ***syn*-3ka**), 7.39-7.27 [(m, 0.36 × 6H for ***syn*-3ka** and 0.64 × 2H for ***anti*-3ka**)], 7.23-7.18 [(m, 0.36 × 2H for ***syn*-3ka** and 0.64H for ***anti*-3ka**)], 7.16-7.07 [(m, 0.36H for ***syn*-3ka** and 0.64 × 6H for ***anti*-3ka**)], 6.96-6.91 [(m, 0.36H for ***syn*-3ka** and 0.64H for ***anti*-3ka**)], 6.83 (d, *J* = 7.0 Hz, 0.64 × 4H for ***anti*-3ka**), 6.69 (s, 0.36H for ***syn*-3ka**), 6.49 (s, 0.64H for ***anti*-3ka**), 4.13 (d, *J* = 13.8 Hz, 0.36 × 2H for ***syn*-3ka**), 3.91 (d, *J* = 13.8 Hz, 0.64 × 2H for ***anti*-3ka**), 3.86 (s, 0.64 × 3H for ***anti*-3ka**), 3.70 (d, *J* = 11.1 Hz, 0.64H for ***anti*-3ka**), 3.70 (s, 0.64 × 3H for ***anti*-3ka**), 3.66-3.57 [(m, 0.36 × 2H for ***syn*-3ka** and 0.64H for ***anti*-3ka**)], 3.65 (s, 0.36 × 3H for ***syn*-3ka**), 3.45 (s, 0.36 × 3H for ***syn*-3ka**), 3.34 (d, *J* = 13.8 Hz, 0.36 × 2H for ***syn*-3ka**), 3.25 (d, *J* = 13.9 Hz, 0.64 × 2H for ***anti*-3ka**), 1.43 (d, *J* = 6.3 Hz, 0.36 × 3H for ***syn*-3ka**), 1.25 (d, *J* = 6.7 Hz, 0.64 × 3H for ***anti*-3ka**); ^{13}C{^{1}H} NMR ($CDCl_3$, 100 MHz): δ 173.1, 171.8, 139.6, 139.4, 137.2, 137.1, 129.2, 129.1, 128.5, 128.0, 127.3, 127.2, 126.9, 126.8, 126.2, 121.4, 121.3, 120.1, 119.7, 118.7, 118.6, 117.1, 116.4, 109.2, 109.1, 66.5, 66.4, 54.7, 54.6, 51.0, 50.6, 32.7, 32.6, 31.4, 30.4, 20.0, 19.3 (One sp^2 C signal overlaps with others.); HRMS (APCI) m/z ([M+H]$^+$) calcd for $C_{28}H_{31}N_2O_2$: 427.2380, found: 427.2381. CHIRALPAK AD-H column, 96/4 hexane/isopropyl alcohol, 0.5 mL/min, major isomers: t_R = 15.1, 36.1 min, minor isomers: t_R = 12.4, 18.3 min.

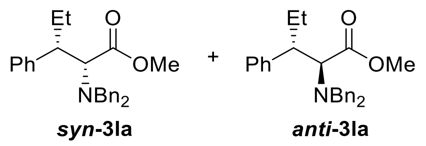

A 41:59 diastereomixture of Methyl (2*R*,3*S*)-2-(dibenzylamino)-3-phenylpentanoate (*syn*-3la) and Methyl (2*S*,3*S*)-2-(dibenzylamino)-3-phenylpentanoate (*anti*-3la)

It was purified by silica gel column chromatography with hexane/ethyl acetate (20/1, v/v) and GPC ($CHCl_3$): 49.9 mg (86%, 0.15 mmol scale); colorless oil; ^{1}H NMR ($CDCl_3$, 400 MHz): δ 7.43 (d, *J* = 7.2 Hz, 0.41 × 4H for ***syn*-3la**), 7.36 (t, *J* = 7.2 Hz, 0.41 × 4H for ***syn*-3la**), 7.32-7.25 [(m, 0.41 × 2H for ***syn*-3la** and 0.59 × 3H for ***anti*-3la**)], 7.21-7.16 [(m, 0.41 × 2H for ***syn*-3la** and 0.59 × 6H for ***anti*-3la**)], 7.15-7.11 (m, 0.41H for ***syn*-3la**), 6.99-6.97 (m, 0.41 × 2H for ***syn*-3la**), 6.88-6.83 (m, 0.59 × 6H for ***anti*-3la**), 4.10 (d, *J* = 14.0 Hz, 0.41 × 2H for ***syn*-3la**), 3.87 (s, 0.59 × 3H for ***anti*-3la**), 3.84 (d, *J* = 13.9 Hz, 0.59 × 2H for ***anti*-3la**), 3.53 [(d, *J* = 11.6 Hz, 0.41H for ***syn*-3la** and 0.59H for ***anti*-3la**)], 3.42 (s, 0.41 × 3H for ***syn*-3la**), 3.34 (d, *J* = 13.9 Hz, 0.41 × 2H for ***syn*-3la**), 3.17 (d, *J* = 13.7 Hz, 0.59 × 2H for ***anti*-3la**), 3.08 (td, *J* = 3.3, 10.9 Hz, 0.59H for ***anti*-3la**), 3.01 (td, *J* = 3.4, 11.3 Hz, 0.41H for ***syn*-3la**), 2.41-2.31 (m, 0.41H for ***syn*-3la**), 1.51-1.45 (m, 0.59H for ***anti*-3la**), 1.35-1.24 [(m, 0.41H for ***syn*-3la** and 0.59H for ***anti*-3la**)], 0.66 (t, *J* = 7.3 Hz, 0.59 × 3H for ***anti*-3la**), 0.63 (t, *J* = 7.4 Hz, 0.41 × 3H for ***syn*-3la**); ^{13}C{^{1}H} NMR ($CDCl_3$, 100 MHz): δ 172.5, 171.3, 141.4, 141.2, 139.5, 139.1, 129.3 (2C), 129.1, 128.7, 128.5, 128.3, 128.1, 128.0, 127.2, 127.0, 126.6, 126.4, 66.6, 65.6, 54.9, 54.3, 51.1, 50.6, 47.3, 46.9, 27.5, 25.0, 12.1, 11.6; HRMS (APCI) m/z ([M+H]$^+$) calcd for $C_{26}H_{30}NO_2$: 388.2271, found: 388.2277. CHIRALPAK AD-H column, 99/1 hexane/isopropyl alcohol, 0.5 mL/min, major isomers: t_R = 9.3, 16.2 min, minor isomers: t_R = 10.5, 12.1 min.

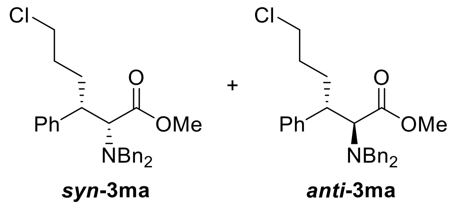

A 44:56 diastereomixture of Methyl (2*R*,3*S*)-6-chloro-2-(dibenzylamino)-3-phenylhexanoate (*syn*-3ma) and Methyl (2*S*,3*S*)-6-chloro-2-(dibenzylamino)-3-phenylhexanoate (*anti*-3ma)

It was purified by silica gel column chromatography with hexane/ethyl acetate (20/1, v/v) and GPC ($CHCl_3$): 57.6 mg (88%, 0.15 mmol scale); colorless oil; ^{1}H NMR ($CDCl_3$, 400 MHz): δ 7.44 (d, *J* = 7.2 Hz, 0.44 × 4H for ***syn*-3ma**), 7.37 (t, *J* = 7.2 Hz, 0.44 × 4H for ***syn*-3ma**), 7.33-7.25 [(m, 0.44 × 2H for ***syn*-3ma** and 0.56 × 3H for ***anti*-3ma**)], 7.22-7.18 [(m, 0.44 × 2H for ***syn*-3ma** and 0.56 × 6H for

***anti*-3ma**)], 7.16-7.13 (m, 0.44H for ***syn*-3ma**), 6.98-6.96 (m, 0.44 × 2H for ***syn* -3ma**), 6.87-6.83 (m, 0.56 × 6H for ***anti*-3ma**), 4.09 (d, *J* = 13.8 Hz, 0.44 × 2H for ***syn*-3ma**), 3.89 (s, 0.56 × 3H for ***anti*-3ma**), 3.83 (d, *J* = 13.6 Hz, 0.56 × 2H for ***anti*-3ma**), 3.533 (d, *J* = 11.4 Hz, 0.56H for ***anti* -3ma**), 3.530 (d, *J* = 11.4 Hz, 0.44H for ***syn*-3ma**), 3.46-3.33 [(m, 0.44 × 2H for ***syn*-3ma** and 0.56 × 2H for ***anti*-3ma**)], 3.44 (s, 0.44 × 3H for ***syn*-3ma**), 3.34 (d, *J* = 13.9 Hz, 0.44 × 2H for ***syn*-3ma**), 3.22-3.09 [(m, 0.44H for ***syn*-3ma** and 0.56H for ***anti*-3ma**)], 3.18 (d, *J* = 13.6 Hz, 0.56 × 2H for ***anti*-3ma**), 2.56-2.49 (m, 0.44H for ***syn*-3ma**), 1.62-1.36 [(m, 0.44 × 3H for ***syn*-3ma** and 0.56 × 4H for ***anti*-3ma**)]; $^{13}C\{^1H\}$ NMR ($CDCl_3$, 100 MHz): δ 172.2, 171.0, 141.0, 140.8, 139.3, 138.9, 129.3, 129.2, 129.1, 128.53, 128.50 (2C), 128.3, 128.0, 127.3, 127.1, 126.9, 126.8, 66.6, 65.5, 55.0, 54.3, 51.2, 50.7, 45.3, 45.0, 44.9, 44.4, 31.8, 30.5, 29.9, 29.4; HRMS (APCI) m/z ($[M+H]^+$) calcd for $C_{27}H_{31}ClNO_2$: 436.2038, found: 436.2024. CHIRALPAK AD-H column, 98.5/1.5 hexane/isopropyl alcohol, 0.5 mL/min, major isomers: t_R = 15.3, 37.2 min, minor isomers: t_R = 21.5, 22.3 min.

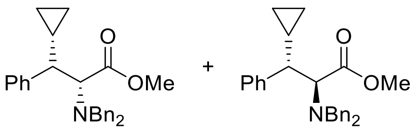

***syn*-3na** ***anti*-3na**

A 45:55 diastereomixture of Methyl (2*R*,3*S*)-3-cyclopropyl-2-(dibenzylamino)-3-phenylpropanoate (*syn*-3na) and Methyl (2*S*,3*S*)-3-cyclopropyl-2-(dibenzylamino)-3-phenylpropanoate (*anti*-3na)

It was purified by silica gel column chromatography with hexane/ethyl acetate (20/1, v/v) and GPC ($CHCl_3$): 36.6 mg (61%, 0.15 mmol scale); colorless oil; ^1H NMR ($CDCl_3$, 400 MHz): δ 7.50 (d, *J* = 7.4 Hz, 0.55 × 4H for ***anti*-3na**), 7.36 (t, *J* = 7.7 Hz, 0.55 × 4H for ***anti*-3na**), 7.32-7.27 [(m, 0.45 × 3H for ***syn*-3na** and 0.55 × 2H for ***anti*-3na**)], 7.22-7.16 [(m, 0.45 × 6H for ***syn*-3na** and 0.55 × 2H for ***anti*-3na**)], 7.15-7.11 (m, 0.55H for ***anti*-3na**), 6.97-6.94 (m, 0.55 × 2H for ***anti*-3na**), 6.91-6.89 (m, 0.45 × 2H for ***syn*-3na**), 6.86-6.84 (m, 0.45 × 4H for ***syn*-3na**), 4.14 (d, *J* = 14.0 Hz, 0.55 × 2H for ***anti*-3na**), 3.87 (s, 0.45 × 3H for ***syn*-3na**), 3.83 (d, *J* = 13.8 Hz, 0.45 × 2H for ***syn*-3na**), 3.76 (d, *J* = 11.4 Hz, 0.45H for ***syn*-3na**), 3.75 (d, *J* = 11.0 Hz, 0.55H for ***anti*-3na**), 3.47 (s, 0.55 × 3H for ***anti*-3na**), 3.39 (d, *J* = 14.0 Hz, 0.55 × 2H for ***anti*-3na**), 3.24 (d, *J* = 13.8 Hz, 0.45 × 2H for ***syn*-3na**), 2.78 (dd, *J* = 8.1, 11.0 Hz, 0.55H for ***anti*-3na**), 2.38 (dd, *J* = 9.7, 11.3 Hz, 0.45H for ***syn*-3na**), 1.23-1.15 (m, 0.55H for ***anti*-3na**), 0.80-0.71 [(m, 0.45H for ***syn*-3na** and 0.55H for ***anti*-3na**)], 0.50-0.42 (m, 0.45H for ***syn*-3na**), 0.29-0.18 [(m, 0.45 × 2H for ***syn*-3na** and 0.55 × 2H for ***anti*-3na**)],

0.07-0.02 (m, 0.45H for ***syn*-3na**), -0.16--0.23 (m, 0.55H for ***anti*-3na**); $^{13}C\{^{1}H\}$ NMR ($CDCl_3$, 100 MHz): δ 173.2, 171.2, 142.1, 141.7, 139.5, 139.2, 129.2, 128.9, 128.7, 128.4, 128.2, 128.1, 128.0 (2C), 127.1, 127.0, 126.6, 126.5, 67.2, 65.6, 55.1, 54.3, 51.2, 50.7, 50.5, 48.6, 16.3, 14.3, 7.4, 6.4, 3.1, 2.3; HRMS (APCI) m/z ($[M+H]^+$) calcd for $C_{27}H_{30}NO_2$: 400.2271, found: 400.2277. CHIRALPAK AD-H column, 98.5/1.5 hexane/isopropyl alcohol, 0.5 mL/min, major isomers: t_R = 12.3, 23.4 min, minor isomers: t_R = 12.9, 17.0 min.

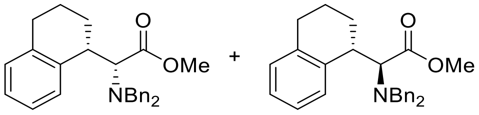

***syn*-3oa** ***anti*-3oa**

A 37:63 diastereomixture of Methyl (*R)-2-(dibenzylamino)-2-((*S**)-1,2,3,4-tetrahydronaphthalen-1-yl)acetate (*syn*-3oa) and Methyl (*S**)-2-(dibenzylamino)-2-((*S**)-1,2,3,4-tetrahydronaphthalen-1-yl)acetate (*anti*-3oa)**

It was purified by silica gel column chromatography with hexane/ethyl acetate (20/1, v/v) and GPC ($CHCl_3$): 42.0 mg (70%, 0.15 mmol scale); colorless oil; ^{1}H NMR ($CDCl_3$, 400 MHz): δ 7.41 (d, J = 7.4 Hz, 0.37 × 4H for ***syn*-3oa**), 7.34 (t, J = 7.2 Hz, 0.37 × 4H for ***syn*-3oa**), 7.28-6.97 [(m, 0.37 × 6H for ***syn*-3oa** and 0.63 × 14H for ***anti*-3oa**)], 4.18-4.11 [(m, 0.37 × 2H for ***syn*-3oa** and 0.63 × 2H for ***anti*-3oa**)], 3.83 (s, 0.63 × 3H for ***anti*-3oa**), 3.59 (s, 0.37 × 3H for ***syn*-3oa**), 3.55 (d, J = 10.9 Hz, 0.63H for ***anti*-3oa**), 3.42-3.34 [(m, 0.37 × 4H for ***syn*-3oa** and 0.63 × 3H for ***anti*-3oa**)], 2.68-2.50 [(m, 0.37 × 3H for ***syn*-3oa** and 0.63H for ***anti*-3oa**)], 2.35-2.27 (m, 0.63H for ***anti*-3oa**), 1.87-1.77 (m, 0.63H for ***anti*-3oa**), 1.64-1.51 [(m, 0.37 × 2H for ***syn*-3oa** and 0.63 × 3H for ***anti*-3oa**)], 1.30-1.18 (m, 0.37H for ***syn*-3oa**); $^{13}C\{^{1}H\}$ NMR ($CDCl_3$, 100 MHz): δ 172.8, 171.9, 139.5, 139.1, 137.9, 137.6, 137.5, 137.0, 131.6, 129.54, 129.47, 129.2, 128.7, 128.45, 128.41, 128.3, 127.2, 126.8, 126.6 (2C), 125.1, 124.7, 64.9, 64.6, 55.0, 54.7, 51.1, 50.7, 37.2, 37.1, 28.4, 27.5, 25.4, 23.2, 18.5, 17.4; HRMS (APCI) m/z ($[M+H]^+$) calcd for $C_{27}H_{30}NO_2$: 400.2271, found: 400.2272.

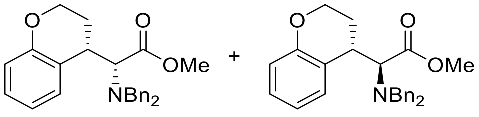

***syn*-3pa** ***anti*-3pa**

A 42:58 diastereomixture of Methyl (*R*)-2-((*S*)-chroman-4-yl)-2-(dibenzylamino)acetate (*syn*-3pa) and Methyl (*S*)-2-((*S*)-chroman-4-yl)-2-(dibenzylamino)acetate (*anti*-3pa)

It was purified by silica gel column chromatography with hexane/ethyl acetate (10/1, v/v) and GPC

($CHCl_3$): 31.3 mg (52%, 0.15 mmol scale); colorless oil; ^{1}H NMR ($CDCl_3$, 400 MHz): δ 7.35-7.14 [(m, 0.42 × 10H for ***syn*-3pa** and 0.58 × 12H for ***anti*-3pa**)], 7.06 (t, *J* = 7.7 Hz, 0.42H for ***syn*-3pa**), 6.97 (d, *J* = 7.8 Hz, 0.42H for ***syn*-3pa**), 6.87 (t, *J* = 7.4 Hz, 0.58H for ***anti*-3pa**), 6.77 (d, *J* = 8.0 Hz, 0.58H for ***anti*-3pa**), 6.73 (t, *J* = 7.5 Hz, 0.42H for ***syn*-3pa**), 6.97 (d, *J* = 8.1 Hz, 0.42H for ***syn*-3pa**), 4.19 (d, *J* = 14.6 Hz, 0.58 × 2H for ***anti*-3pa**), 4.09 (d, *J* = 13.4 Hz, 0.42 × 2H for ***syn*-3pa**), 4.09-4.04 (m, 0.58H for ***anti*-3pa**), 4.02-3.98 (m, 0.42H for ***syn*-3pa**), 3.82 (s, 0.58 × 3H for ***anti*-3pa**), 3.79 (td, *J* = 3.0, 11.4 Hz, 0.58H for ***anti*-3pa**), 3.68 (s, 0.42 × 3H for ***syn*-3pa**), 3.58 (d, *J* = 10.6 Hz, 0.58H for ***anti*-3pa**), 3.45 (d, *J* = 14.9 Hz, 0.58 × 2H for ***anti*-3pa**), 3.44 (d, *J* = 11.2 Hz, 0.42H for ***syn*-3pa**), 3.39-3.32 [(m, 0.42H for ***syn*-3pa** and 0.58H for ***anti*-3pa**)], 3.34 (d, *J* = 13.7 Hz, 0.42 × 2H for ***syn*-3pa**), 3.24 (td, *J* = 2.4, 12.3 Hz, 0.42H for ***syn*-3pa**), 2.54 (dq, *J* = 2.1, 13.7 Hz, 0.42H for ***syn*-3pa**), 2.15-2.06 (m, 0.58H for ***anti*-3pa**), 1.87 (tt, *J* = 4.6, 13.4 Hz, 0.42H for ***syn*-3pa**), 1.65 (dq, *J* = 3.4, 14.5 Hz, 0.58H for ***anti*-3pa**); ^{13}C{^{1}H} NMR ($CDCl_3$, 100 MHz): δ 171.9, 171.6, 154.6, 154.4, 139.1, 138.6, 132.4, 130.4, 129.2, 128.6, 128.4 (3C), 128.3, 127.5, 127.1, 122.5, 121.5, 119.6, 119.3, 117.0, 116.6, 65.4, 64.6, 62.7, 61.7, 55.2, 54.7, 51.2, 51.0, 32.8, 32.7, 25.3, 22.3; HRMS (APCI) m/z ([M+H]$^+$) calcd for $C_{26}H_{28}NO_3$: 402.2064, found: 402.2068. CHIRALCEL OD-H column, 99.4/0.6 hexane/isopropyl alcohol, 0.5 mL/min, major isomers: t_R = 18.0, 22.6 min, minor isomers: t_R = 15.7, 21.6 min.

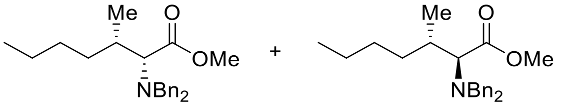

***syn*-3qa** ***anti*-3qa**

A 49:51 diastereomixture of Methyl (2*R*,3*S*)-2-(dibenzylamino)-3-methylheptanoate (*syn*-3qa) and Methyl (2*S*,3*S*)-2-(dibenzylamino)-3-methylheptanoate (*anti*-3qa)

It was purified by silica gel column chromatography with hexane/ethyl acetate (20/1, v/v) and GPC ($CHCl_3$): 35.5 mg (67%, 0.15 mmol scale); colorless oil; ^{1}H NMR ($CDCl_3$, 400 MHz): δ 7.40-7.37 [(m, 0.49 × 4H for ***syn*-3qa** and 0.51 × 4H for ***anti*-3qa**)], 7.32 [(t, *J* = 7.2 Hz, 0.49 × 4H for ***syn*-3qa** and 0.51 × 4H for ***anti*-3qa**)], 7.25-7.22 [(m, 0.49 × 2H for ***syn*-3qa** and 0.51 × 2H for ***anti*-3qa**)], 3.99 [(d, *J* = 13.9 Hz, 0.49 × 2H for ***syn*-3qa** and 0.51 × 2H for ***anti*-3qa**)], 3.78 [(s, 0.49 × 3H for ***syn*-3qa** and 0.51 × 3H for ***anti*-3qa**)], 3.28 (d, *J* = 13.9 Hz, 0.49 × 2H for ***syn*-3qa**), 3.27 (d, *J* = 14.0 Hz, 0.51 × 2H for ***anti*-3qa**), 2.99 (d, *J* = 10.9 Hz, 0.49H for ***syn*-3qa**), 2.96 (d, *J* = 10.9 Hz, 0.51H for ***anti*-3qa**), 2.09-1.97 (m, 0.51 × 2H for ***anti*-3qa**), 1.92-1.83 (m, 0.49H for ***syn*-3qa**), 1.34-1.03 [(m, 0.49 × 5H for ***syn*-3qa** and 0.51 × 5H for ***anti*-3qa**)], 1.01 (d, *J* = 6.6 Hz, 0.51 × 3H for ***anti*-3qa**), 0.91-0.81 (m, 0.49H for ***syn*-3qa**), 0.87 (t, *J* = 7.3 Hz, 0.51 × 3H for ***anti*-3qa**), 0.83 (t, *J* = 6.8 Hz, 0.49 × 3H for

***syn*-3qa**), 0.75 (d, *J* = 6.6 Hz, 0.49 × 3H for ***syn*-3qa**); $^{13}C\{^1H\}$ NMR ($CDCl_3$, 100 MHz): δ 172.7, 172.5, 139.7 (2C), 129.1, 129.0, 128.3 (2C), 127.1, 127.0, 67.2, 66.7, 54.83, 54.76, 50.7 (2C), 33.8, 32.2, 31.8, 31.7, 29.2, 27.9, 23.2, 22.9, 16.6, 16.2, 14.3, 14.1; HRMS (APCI) m/z ($[M+H]^+$) calcd for $C_{23}H_{32}NO_2$: 354.2428, found: 354.2436. CHIRALPAK AD-H column, 99.8/0.2 hexane/isopropyl alcohol, 0.5 mL/min, major isomers: t_R = 14.6, 28.6 min, minor isomers: t_R = 17.2, 20.9 min.

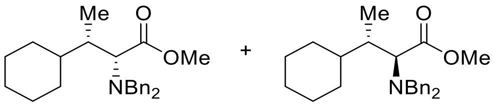

***syn*-3ra** ***anti*-3ra**

A 46:54 diastereomixture of Methyl (2*R*,3*S*)-3-cyclohexyl-2-(dibenzylamino)butanoate (*syn*-3ra) and Methyl (2*S*,3*S*)-3-cyclohexyl-2-(dibenzylamino)butanoate (*anti*-3ra)

It was purified by silica gel column chromatography with hexane/ethyl acetate (20/1, v/v) and GPC ($CHCl_3$): 29.0 mg (51%, 0.15 mmol scale); colorless oil; ^1H NMR ($CDCl_3$, 400 MHz): δ 7.40-7.36 [(m, 0.46 × 4H for ***syn*-3ra** and 0.54 × 4H for ***anti*-3ra**)], 7.34-7.30 [(m, 0.46 × 4H for ***syn*-3ra** and 0.54 × 4H for ***anti*-3ra**)], 7.25-7.21 [(m, 0.46 × 2H for ***syn*-3ra** and 0.54 × 2H for ***anti*-3ra**)], 4.00 (d, *J* = 13.9 Hz, 0.46 × 2H for ***syn*-3ra**), 3.98 (d, *J* = 13.6 Hz, 0.54 × 2H for ***anti*-3ra**), 3.788 (s, 0.54 × 3H for ***anti*-3ra**), 3.785 (s, 0.46 × 3H for ***syn*-3ra**), 3.27 (d, *J* = 14.0 Hz, 0.46 × 2H for ***syn*-3ra**), 3.26 (d, *J* = 13.8 Hz, 0.54 × 2H for ***anti*-3ra**), 3.22 (d, *J* = 11.4 Hz, 0.46H for ***syn*-3ra**), 3.12 (d, *J* = 11.3 Hz, 0.54H for ***anti*-3ra**), 2.10-1.89 (m, 0.54 × 3H for ***anti*-3ra**), 1.77-1.60 [(m, 0.46 × 3H for ***syn*-3ra** and 0.54 × 3H for ***anti*-3ra**)], 1.40-1.00 [(m, 0.46 × 8H for ***syn*-3ra** and 0.54 × 4H for ***anti*-3ra**)], 0.91 (d, *J* = 7.0 Hz, 0.46 × 3H for ***syn*-3ra**), 0.82-0.74 [(m, 0.46H for ***syn*-3ra** and 0.54H for ***anti*-3ra**)], 0.68-0.58 (m, 0.54H for ***anti*-3ra**), 0.63 (d, *J* = 6.8 Hz, 0.54 × 3H for ***anti*-3ra**); $^{13}C\{^1H\}$ NMR ($CDCl_3$, 100 MHz): δ 172.8, 172.6, 139.8, 139.7, 129.3, 128.9, 128.34, 128.30, 127.1, 127.0, 64.6, 64.3, 54.8, 54.7, 50.7 (2C), 39.9, 37.2, 36.7, 36.4, 32.4, 32.2, 27.5, 27.1, 27.0, 26.79 (2C), 26.76, 26.4, 25.4, 11.7, 11.6; HRMS (APCI) m/z ($[M+H]^+$) calcd for $C_{25}H_{34}NO_2$: 380.2584, found: 380.2579. CHIRALPAK AD-H column, 99.4/0.6 hexane/isopropyl alcohol, 0.5 mL/min, major isomers: t_R = 13.7, 27.1 min, minor isomers: t_R = 12.9, 21.9 min.

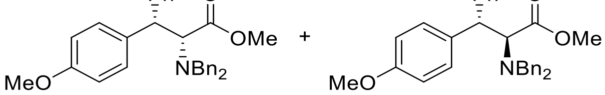

***syn*-3sa** ***anti*-3sa**

A 49:51 diastereomixture of Methyl

(2*R*,3*S*)-2-(dibenzylamino)-3-(4-methoxyphenyl)-3-phenylpropanoate (*syn*-3sa) and Methyl (2*S*,3*S*)-2-(dibenzylamino)-3-(4-methoxyphenyl)-3-phenylpropanoate (*anti*-3sa)

It was purified by silica gel column chromatography with hexane/ethyl acetate (10/1, v/v) and GPC ($CHCl_3$): 28.6 mg (41%, 0.15 mmol scale); white solid; mp 158.7-159.7 °C; ^{1}H NMR ($CDCl_3$, 400 MHz): δ 7.25-7.09 [(m, 0.49 × 10H for ***syn*-3sa** and 0.51 × 10H for ***anti*-3sa**)], 7.06 (d, *J* = 8.8 Hz, 0.49 × 2H for ***syn*-3sa**), 7.00-6.91 [(m, 0.49 × 5H for ***syn*-3sa** and 0.51 × 5H for ***anti*-3sa**)], 6.90 (d, *J* = 8.7 Hz, 0.51 × 2H for ***anti*-3sa**), 6.78 (d, *J* = 8.7 Hz, 0.51 × 2H for ***anti*-3sa**), 6.71 (d, *J* = 8.8 Hz, 0.49 × 2H for ***syn*-3sa**), 4.47 [(d, *J* = 12.1 Hz, 0.49H for ***syn*-3sa** and 0.51H for ***anti*-3sa**)], 4.093 (d, *J* = 12.1 Hz, 0.49H for ***syn*-3sa**), 4.088 (d, *J* = 12.0 Hz, 0.51H for ***anti*-3sa**), 3.98 (d, *J* = 13.5 Hz, 0.51 × 2H for ***anti*-3sa**), 3.97 (d, *J* = 13.6 Hz, 0.49 × 2H for ***syn*-3sa**), 3.82 (s, 0.51 × 3H for ***anti*-3sa**), 3.70 (s, 0.49 × 3H for ***syn*-3sa**), 3.61 (s, 0.49 × 3H for ***syn*-3sa**), 3.58 (s, 0.51 × 3H for ***anti*-3sa**), 3.28 (d, *J* = 13.6 Hz, 0.51 × 2H for ***anti*-3sa**), 3.26 (d, *J* = 13.6 Hz, 0.49 × 2H for ***syn*-3sa**); ^{13}C{^{1}H} NMR ($CDCl_3$, 100 MHz): δ 171.6 (2C), 158.42, 158.37, 141.9, 141.4, 139.03, 138.99, 133.7, 133.4, 130.0, 129.5 (2C), 129.1, 129.0, 128.6, 128.3, 128.14, 128.11, 128.0, 127.2 (2C), 126.7, 126.5, 114.0, 113.7, 64.74, 64.67, 55.5, 55.3, 54.5, 54.4, 51.03, 50.98, 50.59, 50.57; HRMS (APCI) m/z ([M+H]$^+$) calcd for $C_{31}H_{32}NO_3$: 466.2377, found: 466.2367. CHIRALPAK AD-H column, 98.5/1.5 hexane/isopropyl alcohol, 0.5 mL/min, major isomers: t_R = 21.6, 53.3 min, minor isomers: t_R = 34.7, 37.5 min.

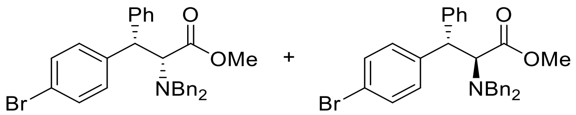

***syn*-3ta** ***anti*-3ta**

A 49:51 diastereomixture of Methyl (2*R*,3*S*)-3-(4-bromophenyl)-2-(dibenzylamino)-3-phenylpropanoate (*syn*-3ta) and Methyl (2*S*,3*S*)-3-(4-bromophenyl)-2-(dibenzylamino)-3-phenylpropanoate (*anti*-3ta)

It was purified by silica gel column chromatography with hexane/ethyl acetate (20/1, v/v) and GPC ($CHCl_3$): 37.0 mg (48%, 0.15 mmol scale); white solid; mp 128.8-129.8 °C; ^{1}H NMR ($CDCl_3$, 400 MHz): δ 7.33 (d, *J* = 8.4 Hz, 0.51 × 2H for ***anti*-3ta**), 7.29 (d, *J* = 8.4 Hz, 0.49 × 2H for ***syn*-3ta**), 7.27-7.07 [(m, 0.49 × 10H for ***syn*-3ta** and 0.51 × 10H for ***anti*-3ta**)], 7.02 (d, *J* = 8.5 Hz, 0.49 × 2H for ***syn*-3ta**), 6.97-6.91 [(m, 0.49 × 5H for ***syn*-3ta** and 0.51 × 5H for ***anti*-3ta**)], 6.81 (d, *J* = 8.4 Hz, 0.51 × 2H for ***anti*-3ta**), 4.48 (d, *J* = 12.1 Hz, 0.49H for ***syn*-3ta**), 4.47 (d, *J* = 12.1 Hz, 0.51H for ***anti*-3ta**), 4.11 (d, *J* = 12.1 Hz, 0.51H for ***anti*-3ta**), 4.09 (d, *J* = 12.1 Hz, 0.49H for ***syn*-3ta**), 3.97 (d, *J* = 13.6 Hz, 0.49 × 2H for ***syn*-3ta**), 3.96 (d, *J* = 13.6 Hz, 0.51 × 2H for ***anti*-3ta**), 3.64 (s, 0.49 × 3H for ***syn*-3ta**),

3.60 (s, 0.51 × 3H for ***anti*-3ta**), 3.28 (d, J = 13.6 Hz, 0.51 × 2H for ***anti*-3ta**), 3.26 (d, J = 13.6 Hz, 0.49 × 2H for ***syn*-3ta**); $^{13}C\{^1H\}$ NMR ($CDCl_3$, 100 MHz): δ 171.2, 171.1, 140.9, 140.6, 140.5, 140.3, 138.8, 138.7, 131.8, 131.3, 130.8, 129.8, 129.50, 129.48, 129.0, 128.8, 128.4, 128.3, 128.2, 128.0, 127.3, 127.2, 127.1, 126.8, 120.8, 120.3, 64.3, 64.2, 54.5, 54.4, 51.2, 51.1, 50.82, 50.77; HRMS (APCI) m/z ($[M+H]^+$) calcd for $C_{30}H_{29}BrNO_2$: 514.1376, found: 514.1390. CHIRALPAK AD-H column, 98.8/1.2 hexane/isopropyl alcohol, 0.5 mL/min, major isomers: t_R = 17.8, 45.8 min, minor isomers: t_R = 34.9, 35.8 min.

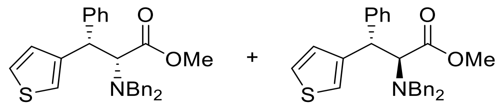

***syn*-3ua** ***anti*-3ua**

A 45:55 diastereomixture of Methyl (2*R*,3*S*)-2-(dibenzylamino)-3-phenyl-3-(thiophen-3-yl)propanoate (*syn*-3ua) and Methyl (2*S*,3*S*)-2-(dibenzylamino)-3-phenyl-3-(thiophen-3-yl)propanoate (*anti*-3ua)

It was purified by silica gel column chromatography with hexane/ethyl acetate (10/1, v/v) and GPC ($CHCl_3$): 22.5 mg (34%, 0.15 mmol scale); pale yellow solid; mp 109.7-110.7 °C; ^1H NMR ($CDCl_3$, 400 MHz): δ 7.29-7.16 [(m, 0.45 × 9H for ***syn*-3ua** and 0.55 × 9H for ***anti*-3ua**)], 7.13-7.10 [(m, 0.45 × 3H for ***syn*-3ua** and 0.55H for ***anti*-3ua**)], 7.03-7.01 (m, 0.45 × 4H for ***syn*-3ua**), 6.98-6.95 (m, 0.55 × 2H for ***anti*-3ua**), 6.93-6.87 [(m, 0.45H for ***syn*-3ua** and 0.55 × 5H for ***anti*-3ua**)], 6.81 (dd, J = 1.3, 5.0 Hz, 0.55H for ***anti*-3ua**), 6.66 (dd, J = 1.3, 5.0 Hz, 0.45H for ***syn*-3ua**), 4.66 (d, J = 12.0 Hz, 0.55H for ***anti*-3ua**), 4.65 (d, J = 12.0 Hz, 0.45H for ***syn*-3ua**), 4.05 (d, J = 12.0 Hz, 0.45H for ***syn*-3ua**), 4.04 (d, J = 12.0 Hz, 0.55H for ***anti*-3ua**), 4.00 (d, J = 13.6 Hz, 0.45 × 2H for ***syn*-3ua**), 3.94 (d, J = 13.6 Hz, 0.55 × 2H for ***anti*-3ua**), 3.69 (s, 0.55 × 3H for ***anti*-3ua**), 3.56 (s, 0.45 × 3H for ***syn*-3ua**), 3.29 (d, J = 13.6 Hz, 0.45 × 2H for ***syn*-3ua**), 3.25 (d, J = 13.6 Hz, 0.55 × 2H for ***anti*-3ua**); $^{13}C\{^1H\}$ NMR ($CDCl_3$, 100 MHz): δ 171.5, 171.3, 142.3, 141.6, 141.1, 140.7, 139.0, 138.9, 129.50, 129.45, 129.2, 128.6, 128.4, 128.3, 128.2 (2C), 128.1, 127.8, 127.22, 127.17, 126.9, 126.7, 125.7, 125.0, 122.0, 121.0, 65.4, 64.8, 54.5, 54.4, 51.2, 51.0, 46.8, 46.7; HRMS (APCI) m/z ($[M+H]^+$) calcd for $C_{28}H_{28}NO_2S$: 442.1835, found: 442.1832. CHIRALPAK AD-H column, 98.5/1.5 hexane/isopropyl alcohol, 0.5 mL/min, major isomers: t_R = 23.3, 37.9 min, minor isomers: t_R = 25.6, 45.2 min.

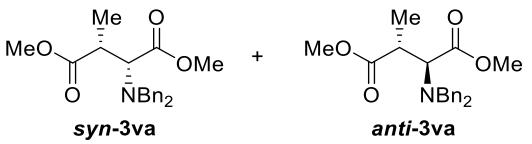

A 36:64 diastereomixture of dimethyl (2*R**,3*R**)-2-(dibenzylamino)-3-methylsuccinate (*syn*-3va) and dimethyl (2*S**,3*R**)-2-(dibenzylamino)-3-methylsuccinate (*anti*-3va)

It was purified by silica gel column chromatography with hexane/ethyl acetate (5/1, v/v) and GPC ($CHCl_3$): 24.5 mg (46%, 0.15 mmol scale); colorless oil; ^{1}H NMR ($CDCl_3$, 400 MHz): δ 7.36-7.21 [(m, 0.36 × 10H for ***syn*-3va** and 0.64 × 10H for ***anti*-3va**)], 3.98 (d, *J* = 13.6 Hz, 0.64 × 2H for ***anti*-3va**), 3.86 (d, *J* = 13.5 Hz, 0.36 × 2H for ***syn*-3va**), 3.82 (s, 0.64 × 3H for ***anti*-3va**), 3.81 (s, 0.36 × 3H for ***syn*-3va**), 3.61 (s, 0.64 × 3H for ***anti*-3va**), 3.60 (s, 0.36 × 3H for ***syn*-3va**), 3.54 (d, *J* = 11.4 Hz, 0.64H for ***anti*-3va**), 3.47 (d, *J* = 11.0 Hz, 0.36H for ***syn*-3va**), 3.41 (d, *J* = 13.5 Hz, 0.36 × 2H for ***syn*-3va**), 3.26 (d, *J* = 13.6 Hz, 0.64 × 2H for ***anti*-3va**), 3.16 (dq, *J* = 6.8, 11.4 Hz, 0.64H for ***anti*-3va**), 2.98 (dq, *J* = 7.2, 11.0 Hz, 0.36H for ***syn*-3va**), 1.19 (d, *J* = 7.2 Hz, 0.36 × 3H for ***syn*-3va**), 1.04 (d, *J* = 6.8 Hz, 0.64 × 3H for ***anti*-3va**); $^{13}C\{^1H\}$ NMR ($CDCl_3$, 100 MHz): δ 175.8, 174.2, 172.0, 170.5, 138.9, 138.8, 129.3 (2C), 128.5, 128.2, 127.4, 127.2, 64.5, 62.7, 55.1, 54.9, 51.9, 51.7, 51.4, 51.3, 40.6, 39.3, 15.2, 14.7; HRMS (APCI) m/z ($[M+H]^+$) calcd for $C_{21}H_{26}NO_4$: 356.1856, found: 356.1858.

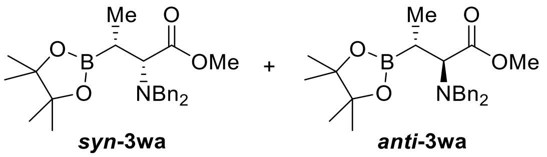

A 79:21 diastereomixture of Methyl (2*R*,3*R*)-2-(dibenzylamino)-3-(4,4,5,5-tetramethyl-1,3,2-dioxaborolan-2-yl)butanoate (*syn*-3wa) and Methyl (2*S*,3*R*)-2-(dibenzylamino)-3-(4,4,5,5-tetramethyl-1,3,2-dioxaborolan-2-yl)butanoate (*anti*-3wa)

It was purified by silica gel column chromatography with hexane/ethyl acetate (20/1, v/v) and GPC ($CHCl_3$): 36.8 mg (58%, 0.15 mmol scale); white solid; mp 96.9-97.9 °C; ^{1}H NMR ($CDCl_3$, 400 MHz): δ 7.38-7.34 [(m, 0.79 × 4H for ***syn*-3wa** and 0.21 × 4H for ***anti*-3wa**)], 7.31-7.27 [(m, 0.79 × 4H for ***syn*-3wa** and 0.21 × 4H for ***anti*-3wa**)], 7.23-7.20 [(m, 0.79 × 2H for ***syn*-3wa** and 0.21 × 2H for ***anti*-3wa**)], 4.00 (d, *J* = 13.8 Hz, 0.21 × 2H for ***anti*-3wa**), 3.90 (d, *J* = 13.7 Hz, 0.79 × 2H for ***syn*-3wa**), 3.75 (s, 0.79 × 3H for ***syn*-3wa**), 3.74 (s, 0.21 × 3H for ***anti*-3wa**), 3.50 (d, *J* = 11.6 Hz, 0.21H for ***anti*-3wa**), 3.41 (d, *J* = 13.7 Hz, 0.79 × 2H for ***syn*-3wa**), 3.32 (d, *J* = 13.9 Hz, 0.21 × 2H for ***anti*-3wa**),

3.27 (d, J = 11.8 Hz, 0.79H for ***syn*-3wa**), 1.83 (dq, J = 7.2, 11.5 Hz, 0.21H for ***anti*-3wa**), 1.59 (dq, J = 7.4, 11.8 Hz, 0.79H for ***syn*-3wa**), 1.26 (s, 0.21 × 6H for ***anti*-3wa**), 1.24 (s, 0.21 × 6H for ***anti*-3wa**), 1.18 (s, 0.79 × 6H for ***syn*-3wa**), 1.15 (s, 0.79 × 6H for ***syn*-3wa**), 1.00 (d, J = 7.4 Hz, 0.79 × 3H for ***syn*-3wa**), 0.86 (d, J = 7.2 Hz, 0.21 × 3H for ***anti*-3wa**); $^{13}C\{^1H\}$ NMR ($CDCl_3$, 100 MHz): δ 173.6, 172.8, 139.9, 139.5, 129.5, 129.2, 128.3, 128.0, 127.0, 126.9, 83.2, 83.1, 65.0, 63.8, 55.2, 54.5, 51.0, 50.7, 25.2, 25.0, 24.9, 24.4, 12.9, 12.6 (The carbon signal bound to boron was not observed due to quadrupolar relaxation.); ^{11}B NMR ($CDCl_3$, 128 MHz): δ 33.21; HRMS (APCI) m/z ($[M+H]^+$) calcd for $C_{25}H_{35}BNO_4$: 424.2658, found: 424.2655. CHIRALPAK AD-H column, 99/1 hexane/isopropyl alcohol, 0.5 mL/min, major isomers: t_R = 14.3, 17.2 min, minor isomers: t_R = 26.6, 29.0 min.

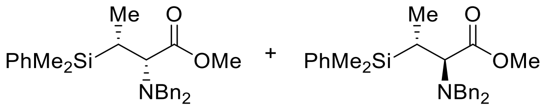

***syn*-3xa** ***anti*-3xa**

A 66:34 diastereomixture of Methyl (2*S*,3*R*)-2-(dibenzylamino)-3-(dimethyl(phenyl)silyl)butanoate (*syn*-3xa) and Methyl (2*R*,3*R*)-2-(dibenzylamino)-3-(dimethyl(phenyl)silyl)butanoate (*anti*-3xa)

It was purified by silica gel column chromatography with hexane/ethyl acetate (20/1, v/v) and GPC ($CHCl_3$): 39.5 mg (61%, 0.15 mmol scale); colorless oil; ^1H NMR ($CDCl_3$, 400 MHz): δ 7.38-7.20 [(m, 0.66 × 15H for ***syn*-3xa** and 0.34 × 15H for ***anti*-3xa**)], 3.98 (d, J = 14.0 Hz, 0.66 × 2H for ***syn*-3xa**), 3.95 (d, J = 13.9 Hz, 0.34 × 2H for ***anti*-3xa**), 3.70 (s, 0.34 × 3H for ***anti*-3xa**), 3.57 (d, J = 8.1 Hz, 0.34H for ***anti*-3xa**), 3.46 (d, J = 13.9 Hz, 0.34 × 2H for ***anti*-3xa**), 3.45 (s, 0.66 × 3H for ***syn*-3xa**), 3.28 (d, J = 12.4 Hz, 0.66H for ***syn*-3xa**), 3.18 (d, J = 14.0 Hz, 0.66 × 2H for ***syn*-3xa**), 1.68 (dq, J = 7.4, 12.4 Hz, 0.66H for ***syn*-3xa**), 1.64-1.56 (m, 0.34H for ***anti*-3xa**), 1.02 (d, J = 7.4 Hz, 0.66 × 3H for ***syn*-3xa**), 0.86 (d, J = 7.5 Hz, 0.34 × 3H for ***anti*-3xa**), 0.16 (s, 0.34 × 3H for ***anti*-3xa**), 0.123 (s, 0.66 × 3H for ***syn*-3xa**), 0.118 (s, 0.66 × 3H for ***syn*-3xa**), 0.10 (s, 0.34 × 3H for ***anti*-3xa**); $^{13}C\{^1H\}$ NMR ($CDCl_3$, 100 MHz): δ 172.8, 171.6, 139.6, 139.4, 138.8, 137.7, 134.1, 133.9, 129.3, 128.9 (3C), 128.32, 128.29, 127.8, 127.6, 127.03, 126.97, 64.3, 62.0, 55.2, 53.8, 50.61, 50.57, 20.4, 18.3, 12.9, 11.6, -3.4, -4.1, -4.3, -4.6; HRMS (APCI) m/z ($[M+H]^+$) calcd for $C_{27}H_{34}NO_2Si$: 432.2353, found: 432.2355. CHIRALPAK AD-H column, 99/1 hexane/isopropyl alcohol, 0.5 mL/min, major isomers: t_R = 13.0, 16.3 min, minor isomers: t_R = 14.8, 17.4 min.

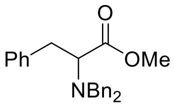

3ya

Methyl dibenzylphenylalaninate (3ya)

It was purified by silica gel column chromatography with hexane/ethyl acetate (20/1, v/v) and GPC ($CHCl_3$): 48.0 mg (89%, 0.15 mmol scale); white solid; mp 81.2-82.2 °C; ^{1}H NMR ($CDCl_3$, 400 MHz): δ 7.25-7.16 (m, 13H), 7.04-6.99 (m, 2H), 3.95 (d, *J* = 14.0 Hz, 2H), 3.73 (s, 3H), 3.67 (dd, *J* = 7.2, 8.2 Hz, 1H), 3.54 (d, *J* = 14.0 Hz, 2H), 3.12 (dd, *J* = 7.2, 14.0 Hz, 1H), 2.99 (dd, *J* = 8.3, 14.0 Hz, 1H); ^{13}C{^{1}H} NMR ($CDCl_3$, 100 MHz): δ 172.9, 139.4, 138.3, 129.5, 128.8, 128.3 (2C), 127.0, 126.4, 62.4, 54.5, 51.3, 35.9; HRMS (APCI) m/z ([M+H]$^+$) calcd for $C_{24}H_{26}NO_2$: 360.1958, found: 360.1952. CHIRALPAK AD-H column, 99/1 hexane/isopropyl alcohol, 0.5 mL/min, t_R = 16.6, 18.6 min.

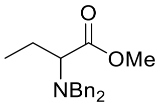

3za

Methyl 2-(dibenzylamino)butanoate (3za)

It was purified by silica gel column chromatography with hexane/ethyl acetate (20/1, v/v): 38.4 mg (86%, 0.15 mmol scale); colorless oil; ^{1}H NMR ($CDCl_3$, 400 MHz): δ 7.37 (d, *J* = 7.2 Hz, 4H), 7.31 (t, *J* = 7.2 Hz, 4H), 7.25-7.21 (m, 2H), 3.93 (d, *J* = 13.9 Hz, 2H), 3.75 (s, 3H), 3.51 (d, *J* = 14.0 Hz, 2H), 3.24 (dd, *J* = 7.0, 8.1 Hz, 1H), 1.81-1.70 (m, 2H), 0.91 (t, *J* = 7.4 Hz, 3H); ^{13}C{^{1}H} NMR ($CDCl_3$, 100 MHz): δ 173.7, 139.9, 128.9, 128.4, 127.1, 62.7, 54.6, 51.1, 22.9, 11.1; HRMS (APCI) m/z ([M+H]$^+$) calcd for $C_{19}H_{24}NO_2$: 298.1802, found: 298.1804.

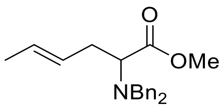

3Aa

Methyl (*E*)-2-(dibenzylamino)hex-4-enoate (3Aa)

It was purified by silica gel column chromatography with hexane/ethyl acetate (20/1, v/v): 24.3 mg (50%, 0.15 mmol scale); colorless oil; ^{1}H NMR ($CDCl_3$, 400 MHz): δ 7.36 (d, *J* = 7.1 Hz, 4H), 7.30 (t, *J* = 7.1 Hz, 4H), 7.25-7.21 (m, 2H), 5.44 (dq, *J* = 6.3, 15.2 Hz, 1H), 5.32 (dt, *J* = 6.8, 15.2 Hz, 1H), 3.91 (d, *J* = 13.9 Hz, 2H), 3.74 (s, 3H), 3.52 (d, *J* = 14.0 Hz, 2H), 3.38 (t, *J* = 7.9 Hz, 1H), 2.52-2.37 (m, 2H), 1.65 (d, *J* = 6.3 Hz, 3H); ^{13}C{^{1}H} NMR ($CDCl_3$, 100 MHz): δ 173.2, 139.8, 128.9, 128.3, 127.6,

127.5, 127.1, 61.3, 54.6, 51.1, 33.1, 18.1; HRMS (APCI) m/z ([M+H]$^+$) calcd for $C_{21}H_{26}NO_2$: 324.1958, found: 324. 1958.

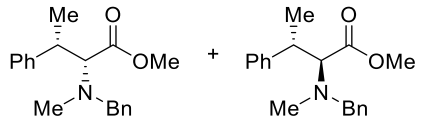

***syn*-3ab** ***anti*-3ab**

A 34:66 diastereomixture of Methyl (2*R*,3*S*)-2-(benzyl(methyl)amino)-3-phenylbutanoate (*syn*-3ab) and Methyl (2*S*,3*S*)-2-(benzyl(methyl)amino)-3-phenylbutanoate (*anti*-3ab)

It was purified by silica gel column chromatography with hexane/ethyl acetate (20/1, v/v) and GPC ($CHCl_3$): 32.1 mg (72%, 0.15 mmol scale); colorless oil; ^{1}H NMR ($CDCl_3$, 400 MHz): δ 7.39-7.09 [(m, 0.34 × 10H for ***syn*-3ab** and 0.66 × 8H for ***anti*-3ab**)], 6.77-6.75 (m, 0.66 × 2H for ***anti*-3ab**), 4.11 (d, *J* = 14.0 Hz, 0.34H for ***syn*-3ab**), 3.81 (s, 0.66 × 3H for ***anti*-3ab**), 3.72 (d, *J* = 13.7 Hz, 0.66H for ***anti*-3ab**), 3.56 (d, *J* = 13.8 Hz, 0.34H for ***syn*-3ab**), 3.48 (d, *J* = 11.3 Hz, 0.66H for ***anti*-3ab**), 3.43 (s, 0.34 × 3H for ***syn*-3ab**), 3.40 (d, *J* = 11.4 Hz, 0.34H for ***syn*-3ab**), 3.36 (d, *J* = 13.7 Hz, 0.66H for ***anti*-3ab**), 3.31-3.19 [(m, 0.34H for ***syn*-3ab** and 0.66H for ***anti*-3ab**)], 2.30 (s, 0.34 × 3H for ***syn*-3ab**), 2.13 (s, 0.66 × 3H for ***anti*-3ab**), 1.39 (d, *J* = 6.9 Hz, 0.34 × 3H for ***syn*-3ab**), 1.18 (d, *J* = 6.8 Hz, 0.66 × 3H for ***anti*-3ab**); $^{13}C\{^1H\}$ NMR ($CDCl_3$, 100 MHz): δ 172.1, 171.2, 144.2, 143.8, 139.7, 139.4, 128.7, 128.6, 128.5, 128.4, 128.4, 128.0, 127.95, 127.88, 127.1, 126.8, 126.7, 126.3, 72.3, 71.5, 58.8, 58.3, 51.0, 50.6, 39.53, 39.51, 38.0, 37.9, 20.5, 19.0; HRMS (APCI) m/z ([M+H]$^+$) calcd for $C_{19}H_{24}NO_2$: 298.1802, found: 298.1798. CHIRALPAK AD-H column, 99.8/0.2 hexane/isopropyl alcohol, 0.5 mL/min, major isomers: t_R = 17.0, 21.9 min, minor isomers: t_R = 16.6, 24.0 min.

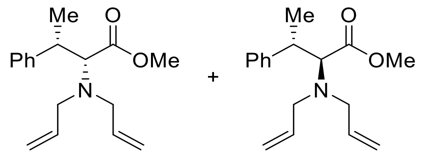

***syn*-3ac** ***anti*-3ac**

A 33:67 diastereomixture of Methyl (2*R*,3*S*)-2-(diallylamino)-3-phenylbutanoate (*syn*-3ac) and Methyl (2*S*,3*S*)-2-(diallylamino)-3-phenylbutanoate (*anti*-3ac)

It was purified by silica gel column chromatography with hexane/ethyl acetate (20/1, v/v) and GPC ($CHCl_3$): 32.0 mg (78%, 0.15 mmol scale); colorless oil; ^{1}H NMR ($CDCl_3$, 400 MHz): δ 7.30-7.11 [(m, 0.33 × 5H for ***syn*-3ac** and 0.67 × 5H for ***anti*-3ac**)], 5.87-5.77 (m, 0.33 × 2H for ***syn*-3ac**), 5.42-5.32

(m, 0.67 × 2H for ***anti*-3ac**), 5.26-5.21 (m, 0.67H for ***anti*-3ac**), 5.17-5.14 (m, 0.67H for ***anti*-3ac**), 4.99-4.94 [(m, 0.33 × 4H for ***syn*-3ac** and 0.67 × 2H for ***anti*-3ac**)], 3.76 (s, 0.67 × 3H for ***anti*-3ac**), 3.56 (d, J = 11.2 Hz, 0.67H for ***anti*-3ac**), 3.55-3.49 (m, 0.33 × 2H for ***syn*-3ac**), 3.51 (d, J = 11.3 Hz, 0.33H for ***syn*-3ac**), 3.38 (s, 0.33 × 3H for ***syn*-3ac**), 3.34-3.29 (m, 0.67 × 2H for ***anti*-3ac**), 3.24-3.11 [(m, 0.33H for ***syn*-3ac** and 0.67H for ***anti*-3ac**)], 2.89 (dd, J = 8.1, 14.6 Hz, 0.33 × 2H for ***syn*-3ac**), 2.76 (dd, J = 8.2, 14.5 Hz, 0.67 × 2H for ***anti*-3ac**), 1.31 (d, J = 7.0 Hz, 0.33 × 3H for ***syn*-3ac**), 1.18 (d, J = 6.8 Hz, 0.67 × 3H for ***anti*-3ac**); $^{13}C\{^1H\}$ NMR ($CDCl_3$, 100 MHz): δ 172.8, 172.0, 144.3, 144.0, 136.6, 136.5, 128.5, 128.1, 128.0, 127.9, 126.7, 126.3, 117.3, 117.0, 68.2, 67.5, 53.4, 53.2, 51.0, 50.6, 39.8, 39.7, 20.0, 19.2; HRMS (APCI) m/z ($[M+H]^+$) calcd for $C_{17}H_{24}NO_2$: 274.1802, found: 274.1809. CHIRALPAK AD-H column, 99/1 hexane/isopropyl alcohol, 0.5 mL/min, major isomers: t_R = 8.7, 13.7 min, minor isomers: t_R = 9.5, 10.1 min.

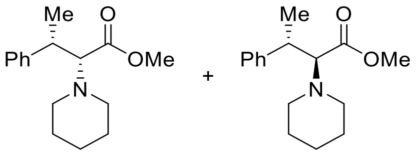

***syn*-3ad** ***anti*-3ad**

A 35:65 diastereomixture of Methyl (2*R*,3*S*)-3-phenyl-2-(piperidin-1-yl)butanoate (*syn*-3ad) and Methyl (2*S*,3*S*)-3-phenyl-2-(piperidin-1-yl)butanoate (*anti*-3ad)

It was purified by silica gel column chromatography with hexane/ethyl acetate (20/1, v/v) and GPC ($CHCl_3$): 32.9 mg (84%, 0.15 mmol scale); colorless oil; ^1H NMR ($CDCl_3$, 400 MHz): δ 7.30-7.15 [(m, 0.35 × 5H for ***syn*-3ad** and 0.65 × 5H for ***anti*-3ad**)], 3.73 (s, 0.65 × 3H for ***anti*-3ad**), 3.38 (s, 0.35 × 3H for ***syn*-3ad**), 3.28 (d, J = 11.0 Hz, 0.65H for ***anti*-3ad**), 3.23 (d, J = 11.0 Hz, 0.35H for ***syn*-3ad**), 3.25-3.13 [(m, 0.35H for ***syn*-3ad** and 0.65H for ***anti*-3ad**)], 2.67-2.61 (m, 0.35 × 2H for ***syn*-3ad**), 2.55-2.51 (m, 0.65 × 2H for ***anti*-3ad**), 2.46-2.42 (m, 0.35 × 2H for ***syn*-3ad**), 2.30-2.26 (m, 0.65 × 2H for ***anti*-3ad**), 1.67-1.43 [(m, 0.35 × 2H for ***syn*-3ad** and 0.65 × 2H for ***anti*-3ad**)], 1.31-1.166 [(m, 0.35 × 4H for ***syn*-3ad** and 0.65 × 4H for ***anti*-3ad**)], 1.30 (d, J = 6.6 Hz, 0.35 × 3H for ***syn*-3ad**), 1.174 (d, J = 6.5 Hz, 0.65 × 3H for ***anti*-3ad**); $^{13}C\{^1H\}$ NMR ($CDCl_3$, 100 MHz): δ 172.0, 171.2, 144.5, 144.0, 128.4, 128.1, 128.0, 127.7, 126.6, 126.1, 74.5, 74.1, 51.0 (2C), 50.8, 50.4, 38.9, 38.8, 26.8, 26.4, 24.9, 24.7, 19.9, 18.8; HRMS (APCI) m/z ($[M+H]^+$) calcd for $C_{16}H_{24}NO_2$: 262.1802, found: 262.1798. CHIRALPAK AD-H column, 99.2/0.8 hexane/isopropyl alcohol, 0.5 mL/min, major isomers: t_R = 10.4, 19.8 min, minor isomers: t_R = 11.5, 12.4 min.

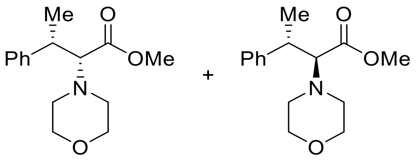

***syn*-3ae** ***anti*-3ae**

A 34:66 diastereomixture of Methyl (2*R*,3*S*)-2-morpholino-3-phenylbutanoate (*syn*-3ae) and Methyl (2*S*,3*S*)-2-morpholino-3-phenylbutanoate (*anti*-3ae)

It was purified by silica gel column chromatography with hexane/ethyl acetate (10/1, v/v): 27.6 mg (70%, 0.15 mmol scale); colorless oil; ^{1}H NMR ($CDCl_3$, 400 MHz): δ 7.31-7.14 [(m, 0.34 × 5H for ***syn*-3ae** and 0.66 × 5H for ***anti*-3ae**)], 3.79-3.74 (m, 0.34 × 2H for ***syn*-3ae**), 3.75 (s, 0.66 × 3H for ***anti*-3ae**), 3.71-3.66 (m, 0.34 × 2H for ***syn*-3ae**), 3.47-3.42 (m, 0.66 × 2H for ***anti*-3ae**), 3.40 (s, 0.34 × 3H for ***syn*-3ae**), 3.37-3.31 (m, 0.66 × 2H for ***anti*-3ae**), 3.33 (d, J = 11.0 Hz, 0.66H for ***anti*-3ae**), 3.26 (d, J = 11.4 Hz, 0.34H for ***syn*-3ae**), 3.25-3.14 [(m, 0.34H for ***syn*-3ae** and 0.66H for ***anti*-3ae**)], 2.73-2.68 (m, 0.34 × 2H for ***syn*-3ae**), 2.61-2.54 [(m, 0.34 × 2H for ***syn*-3ae** and 0.66 × 2H for ***anti*-3ae**)], 2.43-2.38 (m, 0.66 × 2H for ***anti*-3ae**), 1.33 (d, J = 6.8 Hz, 0.34 × 3H for ***syn*-3ae**), 1.19 (d, J = 6.8 Hz, 0.66 × 3H for ***anti*-3ae**); $^{13}C\{^1H\}$ NMR ($CDCl_3$, 100 MHz): δ 171.5, 170.7, 144.0, 143.4, 128.5, 128.3, 127.9, 127.6, 126.8, 126.3, 74.0, 73.5, 67.7, 67.3, 51.1, 50.7, 50.0 (2C), 38.61, 38.55, 19.9, 18.8; HRMS (APCI) m/z ($[M+H]^+$) calcd for $C_{15}H_{22}NO_3$: 264.1594, found: 264.1591. CHIRALCEL OD-H column, 99.6/0.4 hexane/isopropyl alcohol, 0.5 mL/min, major isomers: t_R = 25.1, 36.3 min, minor isomers: t_R = 28.5, 31.5 min.

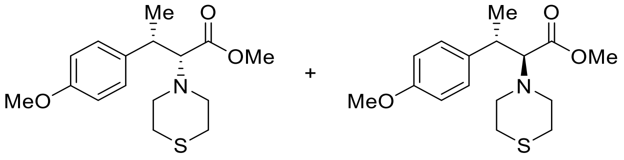

***syn*-3bf** ***anti*-3bf**

A 34:66 diastereomixture of Methyl (2*R*,3*S*)-3-(4-methoxyphenyl)-2-thiomorpholinobutanoate (*syn*-3bf) and Methyl (2*S*,3*S*)-3-(4-methoxyphenyl)-2-thiomorpholinobutanoate (*anti*-3bf)

It was purified by silica gel column chromatography with hexane/ethyl acetate (20/1, v/v) and GPC ($CHCl_3$): 37.1 mg (80%, 0.15 mmol scale); colorless oil; ^{1}H NMR ($CDCl_3$, 400 MHz): δ 7.09 (d, J = 8.7 Hz, 0.34 × 2H for ***syn*-3bf**), 7.04 (d, J = 8.7 Hz, 0.66 × 2H for ***anti*-3bf**), 6.83 (d, J = 8.7 Hz, 0.66 × 2H for ***anti*-3bf**), 6.80 (d, J = 8.8 Hz, 0.34 × 2H for ***syn*-3bf**), 3.81 (s, 0.66 × 3H for ***anti*-3bf**), 3.77 (s, 0.34 × 3H for ***syn*-3bf**), 3.75 (s, 0.66 × 3H for ***anti*-3bf**), 3.43 (s, 0.34 × 3H for ***syn*-3bf**), 3.21-3.11 [(m, 0.34 × 2H for ***syn*-3bf** and 0.66 × 2H for ***anti*-3bf**)], 3.02-2.96 (m, 0.34 × 2H for ***syn*-3bf**), 2.91-2.86 (m,

0.66 × 2H for ***anti*-3bf**), 2.79-2.70 (m, 0.34 × 4H for ***syn*-3bf**), 2.67-2.58 [(m, 0.34 × 2H for ***syn*-3bf** and 0.66 × 2H for ***anti*-3bf**)], 2.40-2.35 (m, 0.66 × 2H for ***anti*-3bf**), 2.28-2.23 (m, 0.66 × 2H for ***anti*-3bf**), 1.27 (d, *J* = 6.6 Hz, 0.34 × 3H for ***syn*-3bf**), 1.14 (d, *J* = 6.3 Hz, 0.66 × 3H for ***anti*-3bf**); $^{13}C\{^1H\}$ NMR ($CDCl_3$, 100 MHz): δ 171.8, 170.8, 158.3, 158.0, 136.1, 135.4, 128.9, 128.5, 113.9, 113.6, 75.4, 74.8, 55.32, 55.30, 52.4, 52.3, 51.2, 50.7, 38.0, 37.8, 28.8, 28.4, 20.3, 18.8; HRMS (APCI) m/z ($[M+H]^+$) calcd for $C_{16}H_{24}NO_3S$: 310.1471, found: 310.1471. CHIRALCEL OD-H column, 99.8/0.2 hexane/isopropyl alcohol, 0.5 mL/min, major isomers: t_R = 31.4, 44.0 min, minor isomers: t_R = 36.0, 39.5 min.

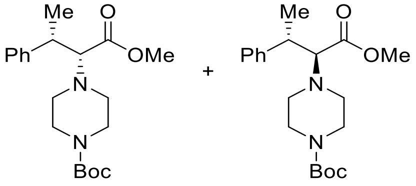

syn-3ag *anti*-3ag

A 34:66 diastereomixture of *tert*-Butyl 4-((2*R*,3*S*)-1-methoxy-1-oxo-3-phenylbutan-2-yl)piperazine-1-carboxylate (*syn*-3ag) and *tert*-Butyl 4-((2*S*,3*S*)-1-methoxy-1-oxo-3-phenylbutan-2-yl)piperazine-1-carboxylate (*anti*-3ag)

It was purified by silica gel column chromatography with hexane/ethyl acetate (10/1, v/v): 33.2 mg (61%, 0.15 mmol scale); colorless oil; ^1H NMR ($CDCl_3$, 400 MHz, at 55 °C): δ 7.29-7.24 [(m, 0.34 × 2H for ***syn*-3ag** and 0.66 × 2H for ***anti*-3ag**)], 7.21-7.14 [(m, 0.34 × 3H for ***syn*-3ag** and 0.66 × 3H for ***anti*-3ag**)], 3.74 (s, 0.66 × 3H for ***anti*-3ag**), 3.52-3.46 (m, 0.34 × 2H for ***syn*-3ag**), 3.44-3.38 (m, 0.34 × 2H for ***syn*-3ag**), 3.38 (s, 0.34 × 3H for ***syn*-3ag**), 3.36 (d, *J* = 11.2 Hz, 0.66H for ***anti*-3ag**), 3.30 (d, *J* = 11.6 Hz, 0.34H for ***syn*-3ag**), 3.25-3.12 [(m, 0.34H for ***syn*-3ag** and 0.66 × 3H for ***anti*-3ag**)], 3.07-3.03 (m, 0.66 × 2H for ***anti*-3ag**), 2.71-2.65 (m, 0.34 × 2H for ***syn*-3ag**), 2.59-2.47 [(m, 0.34 × 2H for ***syn*-3ag** and 0.66 × 2H for ***anti*-3ag**)], 2.36-2.30 (m, 0.66 × 2H for ***anti*-3ag**), 1.47 (s, 0.34 × 9H for ***syn*-3ag**), 1.40 (s, 0.66 × 9H for ***anti*-3ag**), 1.34 (d, *J* = 7.2 Hz, 0.34 × 3H for ***syn*-3ag**), 1.20 (d, *J* = 6.8 Hz, 0.66 × 3H for ***anti*-3ag**); $^{13}C\{^1H\}$ NMR ($CDCl_3$, 100 MHz, at 55 °C): δ 171.5, 170.7, 154.91, 154.86, 144.2, 143.6, 128.5, 128.3, 128.0, 127.6, 126.8, 126.4, 79.7, 79.5, 74.0, 73.5, 51.0, 50.5, 49.7, 49.5, 44.5, 44.2, 39.1, 39.0, 28.61, 28.57, 20.0, 18.9; HRMS (APCI) m/z ($[M+H]^+$) calcd for $C_{20}H_{31}N_2O_4$: 363.2278, found: 363.2270. CHIRALCEL OD-H column, 98.8/1.2 hexane/isopropyl alcohol, 0.5 mL/min, major isomers: t_R = 16.0, 17.8 min, minor isomers: t_R = 15.1, 17.0 min.

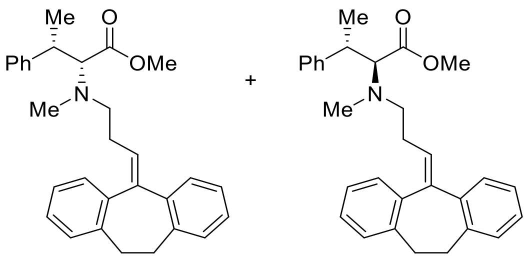

***syn*-3ah** ***anti*-3ah**

A 34:66 diastereomixture of Methyl (2*R*,3*S*)-2-((3-(10,11-dihydro-5*H*-dibenzo[*a*,*d*][7]annulen-5-ylidene)propyl)(methyl)amino)-3-phenylbutanoate (*syn*-3ah) and Methyl (2*S*,3*S*)-2-((3-(10,11-dihydro-5*H*-dibenzo[*a*,*d*][7]annulen-5-ylidene)propyl)(methyl)amino)-3-phenylbutanoate (*anti*-3ah)

It was purified by silica gel column chromatography with hexane/ethyl acetate (20/1, v/v) and GPC ($CHCl_3$): 25.1 mg (38%, 0.15 mmol scale); colorless oil; ^1H NMR ($CDCl_3$, 400 MHz): δ 7.31-6.98 [(m, 0.34 × 13H for ***syn*-3ah** and 0.66 × 13H for ***anti*-3ah**)], 5.93 (t, J = 7.4 Hz, 0.34H for ***syn*-3ah**), 5.48 (br, 0.66H for ***anti*-3ah**), 3.71 (s, 0.66 × 3H for ***anti*-3ah**), 3.43-3.12 [(m, 0.34 × 3H for ***syn*-3ah** and 0.66 × 3H for ***anti*-3ah**)], 3.42 (d, J = 11.2 Hz, 0.66H for ***anti*-3ah**), 3.34 (s, 0.34 × 3H for ***syn*-3ah**), 3.28 (d, J = 11.3 Hz, 0.34H for ***syn*-3ah**), 2.96-2.29 [(m, 0.34 × 2H for ***syn*-3ah** and 0.66 × 6H for ***anti*-3ah**)], 2.29 (s, 0.34 × 3H for ***syn*-3ah**), 2.07 (s, 0.66 × 3H for ***anti*-3ah**), 2.07-1.95 (m, 0.34 × 4H for ***syn*-3ah**), 1.28 (br, 0.34 × 3H for ***syn*-3ah**), 1.14 (d, J = 6.8 Hz, 0.66 × 3H for ***anti*-3ah**); $^{13}C\{^1H\}$ NMR ($CDCl_3$, 100 MHz): δ 172.1, 171.4, 144.1, 143.8, 143.6, 142.9, 141.53, 141.46, 140.4, 140.3, 139.5 (2C), 137.2, 137.1, 130.1, 130.0, 129.8, 129.7, 128.9, 128.7, 128.5, 128.4, 128.2, 128.1, 128.0 (2C), 127.5 (3C), 127.3, 127.1, 126.9, 126.7, 126.2, 126.1, 126.0, 125.9, 125.7, 73.0, 72.2, 54.4, 53.6, 50.9, 50.5, 39.6, 39.4, 37.6, 36.8, 33.93, 33.90, 32.2, 32.0, 28.5, 27.9, 20.6, 18.9; HRMS (APCI) m/z ($[M+H]^+$) calcd for $C_{30}H_{34}NO_2$: 440.2584, found: 440.2577. CHIRALPAK AD-H column, 99.8/0.2 hexane/isopropyl alcohol, 0.5 mL/min, major isomers: t_R = 20.2, 34.0 min, minor isomers: t_R = 21.9, 25.4 min.

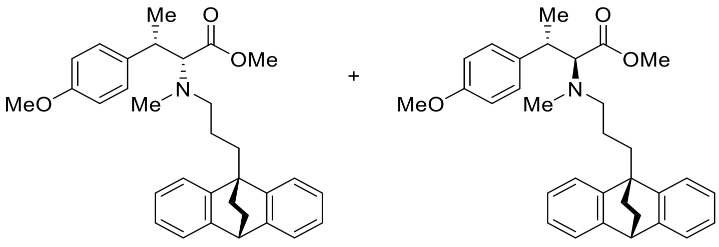

syn-3bi   *anti*-3bi

A 36:64 diastereomixture of Methyl (2*R*,3*S*)-2-((3-(9,10-ethanoanthracen-9(10*H*)-yl)propyl)(methyl)amino)-3-(4-methoxyphenyl)butanoate (*syn*-3bi) and Methyl (2*S*,3*S*)-2-((3-(9,10-ethanoanthracen-9(10*H*)-yl)propyl)(methyl)amino)-3-(4-methoxyphenyl)butanoate (*anti*-3bi)

It was purified by silica gel column chromatography with hexane/ethyl acetate (20/1, v/v) and GPC ($CHCl_3$): 31.2 mg (43%, 0.15 mmol scale); colorless oil; ^{1}H NMR ($CDCl_3$, 400 MHz): δ 7.29-6.97 [(m, 0.36 × 10H for ***syn*-3bi** and 0.64 × 10H for ***anti*-3bi**)], 6.83 (d, *J* = 8.6 Hz, 0.36 × 2H for ***syn*-3bi**), 6.77 (d, *J* = 8.6 Hz, 0.64 × 2H for ***anti*-3bi**), 4.29 (t, *J* = 2.6 Hz, 0.36H for ***syn*-3bi**), 4.22 (t, *J* = 2.6 Hz, 0.64H for ***anti*-3bi**), 3.80 (s, 0.64 × 3H for ***anti*-3bi**), 3.79 (s, 0.36 × 3H for ***syn*-3bi**), 3.53 (d, *J* = 10.6 Hz, 0.64H for ***anti*-3bi**), 3.51 (s, 0.64 × 3H for ***anti*-3bi**), 3.45 (s, 0.36 × 3H for ***syn*-3bi**), 3.40 (d, *J* = 11.4 Hz, 0.36H for ***syn*-3bi**), 3.29-3.18 [(m, 0.36H for ***syn*-3bi** and 0.64H for ***anti*-3bi**)], 2.92-2.86 (m, 0.36H for ***syn*-3bi**), 2.78-2.67 [(m, 0.36H for ***syn*-3bi** and 0.64H for ***anti*-3bi**)], 2.61-2.50 (m, 0.64H for ***anti*-3bi**), 2.52 (t, *J* = 8.2 Hz, 0.36 × 2H for ***syn*-3bi**), 2.46 (s, 0.36 × 3H for ***syn*-3bi**), 2.30 (s, 0.64 × 3H for ***anti*-3bi**), 2.12 (t, *J* = 7.8 Hz, 0.64 × 2H for ***anti*-3bi**), 2.05-1.91 (m, 0.36 × 2H for ***syn*-3bi**), 1.87-1.82 (m, 0.36 × 2H for ***syn*-3bi**), 1.80-1.73 [(m, 0.36 × 2H for ***syn*-3bi** and 0.64 × 2H for ***anti*-3bi**)], 1.65-1.58 (m, 0.64 × 2H for ***anti*-3bi**), 1.48-1.40 (m, 0.64 × 2H for ***anti*-3bi**), 1.38 (d, *J* = 6.9 Hz, 0.36 × 3H for ***syn*-3bi**), 1.19 (d, *J* = 6.8 Hz, 0.64 × 3H for ***anti*-3bi**); $^{13}C\{^1H\}$ NMR ($CDCl_3$, 100 MHz): δ 172.3, 171.5, 158.3, 158.1, 145.83, 145.80, 145.6, 145.5, 145.20, 145.17, 145.10 (2C), 136.4, 135.8, 128.9, 128.5, 125.4, 125.34, 125.30 (4C), 125.2, 125.1, 123.50, 123.47, 123.3, 123.2, 121.8, 121.53, 121.51, 121.4, 114.0, 113.9, 73.3, 72.6, 56.0, 55.6, 55.3, 55.1, 51.0, 50.6, 44.9, 44.74, 44.69, 44.65, 38.8, 38.7, 37.7, 37.4, 29.8, 29.6, 28.9, 28.3, 27.83, 27.78, 23.6, 23.0, 20.9, 19.1 (^{13}C signals are complicated because of rotamers associated with the ethanoanthracene moiety.); HRMS (APCI) m/z ($[M+H]^+$) calcd for $C_{32}H_{38}NO_3$: 484.2846, found: 484.2850. CHIRALCEL OD-H column, 99.8/0.2 hexane/isopropyl alcohol, 0.5 mL/min, major isomers: t_R = 44.0, 86.9 min, minor isomers: t_R = 41.5, 99.6 min.

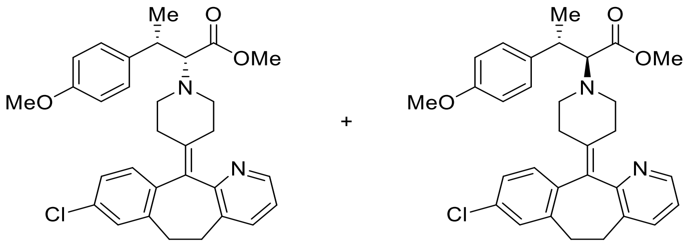

syn-**3bj** *anti*-**3bj**

A 35:65 diastereomixture of Methyl (2*R*,3*S*)-2-(4-(8-chloro-5,6-dihydro-11*H*-benzo[5,6]cyclohepta[1,2-*b*]pyridin-11-ylidene)piperidin-1-yl)-3-(4-methoxyphenyl)butanoate (*syn*-3bj) and Methyl (2*S*,3*S*)-2-(4-(8-chloro-5,6-dihydro-11*H*-benzo[5,6]cyclohepta[1,2-*b*]pyridin-11-ylidene)piperidin-1-yl)-3-(4-methoxyphenyl)butanoate (*anti*-3bj)

It was purified by silica gel column chromatography with hexane/ethyl acetate (10/1, v/v) and GPC ($CHCl_3$): 41.9 mg (54%, 0.15 mmol scale); colorless oil; ^{1}H NMR ($CDCl_3$, 400 MHz): δ 8.40 (d, J = 4.6 Hz, 0.35H for ***syn*-3bj**), 8.35 (td, J = 1.6, 4.6 Hz, 0.65H for ***anti*-3bj**), 7.42 (d, J = 7.8 Hz, 0.35H for ***syn*-3bj**), 7.39 (d, J = 7.7 Hz, 0.65H for ***anti*-3bj**), 7.15-7.01 [(m, 0.35 × 6H for ***syn*-3bj** and 0.65 × 6H for ***anti*-3bj**)], 6.82 (d, J = 8.7 Hz, 0.65 × 2H for ***anti*-3bj**), 6.79 (d, J = 8.7 Hz, 0.35 × 2H for ***syn*-3bj**), 3.81 (s, 0.65 × 3H for ***anti*-3bj**), 3.76 (s, 0.35 × 3H for ***syn*-3bj**), 3.70 (s, 0.65 × 3H for ***anti*-3bj**), 3.42-3.22 [(m, 0.35 × 3H for ***syn*-3bj** and 0.65 × 3H for ***anti*-3bj**)], 3.37 (d, J = 2.5 Hz, 0.35 × 3H for ***syn*-3bj**), 3.18-3.08 [(m, 0.35H for ***syn*-3bj** and 0.65H for ***anti*-3bj**)], 2.95-2.92 (m, 0.35H for ***syn*-3bj**), 2.84-2.70 [(m, 0.35 × 3H for ***syn*-3bj** and 0.65 × 3H for ***anti*-3bj**)], 2.61-2.54 (m, 0.35 × 2H for ***syn*-3bj**), 2.49-1.92 [(m, 0.35 × 4H for ***syn*-3bj** and 0.65 × 7H for ***anti*-3bj**)], 1.31 (d, J = 6.9 Hz, 0.35 × 3H for ***syn*-3bj**), 1.15 (d, J = 6.7 Hz, 0.65 × 3H for ***anti*-3bj**); $^{13}C\{^1H\}$ NMR ($(CD_3)_2SO$, 100 MHz, at 120 °C): δ 170.5, 169.5, 157.5, 157.2, 156.90, 156.85, 145.7, 145.6, 139.5, 139.4, 137.7, 137.6, 137.3, 137.2, 136.43, 136.36, 135.8, 135.2, 132.54, 132.47, 132.0, 131.8, 131.03, 130.95, 130.1, 130.0, 128.2, 128.1, 128.0, 127.8, 125.0, 124.9, 121.44, 121.37, 113.3, 113.1, 72.6, 72.2, 54.60, 54.58, 49.8, 49.3, 49.24, 49.17, 37.2, 37.1, 30.8, 30.64, 30.58, 30.4, 30.11, 30.06, 19.0, 18.3 (Two sp^3 C signals are overlapped with other signals.) (note: at r.t., more complicated ^{13}C signals were obtained because of conformers associated with the two ring systems.); HRMS (APCI) m/z ([M+H]$^+$) calcd for $C_{31}H_{34}ClN_2O_3$: 517.2252, found: 517.2259. CHIRAL ART Amylose-SA (3 μm) column, 98/2 hexane/isopropyl alcohol, 0.5 mL/min, major isomers: t_R = 43.2 min, minor isomers: t_R = 48.3 min for ***syn*-3bj**, CHIRAL ART Amylose-SA (3 μm) column, 92/8 hexane/isopropyl alcohol, 0.5 mL/min, major isomers: t_R = 17.4 min, minor isomers: t_R = 18.8 min for ***anti*-3bj**.

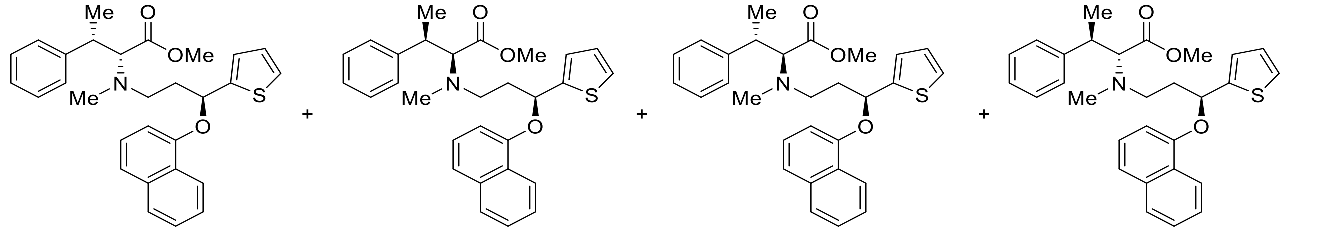

(2*R*,3*S*)-3ak (2*S*,3*R*)-3ak (2*S*,3*S*)-3ak (2*R*,3*R*)-3ak

A 30:1:66:3 diastereomixture of Methyl (2*R*,3*S*)-2-(methyl((*S*)-3-(naphthalen-1-yloxy)-3-(thiophen-2-yl)propyl)amino)-3-phenylbutanoate ((2*R*,3*S*)-3ak) and (2*S*,3*R*)-2-(methyl((*S*)-3-(naphthalen-1-yloxy)-3-(thiophen-2-yl)propyl)amino)-3-phenylbutanoate ((2*S*,3*R*)-3ak) and (2*S*,3*S*)-2-(methyl((*S*)-3-(naphthalen-1-yloxy)-3-(thiophen-2-yl)propyl)amino)-3-phenylbutanoate ((2*S*,3*S*)-3ak) and (2*R*,3*R*)-2-(methyl((*S*)-3-(naphthalen-1-yloxy)-3-(thiophen-2-yl)propyl)amino)-3-phenylbutanoate ((2*R*,3*R*)-3ak)

It was purified by silica gel column chromatography with hexane/ethyl acetate (20/1, v/v): 39.1 mg (55%, 0.15 mmol scale); colorless oil; ^{1}H NMR (CDCl$_3$, 400 MHz) for **(2*R*,3*S*)-3ak** and **(2*S*,3*S*)-3ak**: δ 8.43-8.41 (m, 0.30H for **(2*R*,3*S*)-3ak**), 8.24-8.22 (m, 0.66H for **(2*S*,3*S*)-3ak**), 7.83-7.81 (m, 0.30H for **(2*R*,3*S*)-3ak**), 7.75-7.73 (m, 0.66H for **(2*S*,3*S*)-3ak**), 7.53-7.51 (m, 0.30 × 2H for **(2*R*,3*S*)-3ak**), 7.45-7.10 [(m, 0.30 × 9H for **(2*R*,3*S*)-3ak** and 0.66 × 9H for **(2*S*,3*S*)-3ak**)], 6.98-6.96 (m, 0.30H for **(2*R*,3*S*)-3ak**), 6.92-6.89 [(m, 0.30H for **(2*R*,3*S*)-3ak** and 0.66H for **(2*S*,3*S*)-3ak**)], 6.85-6.82 (m, 0.66 × 2H for **(2*S*,3*S*)-3ak**), 6.27 (d, *J* = 7.7 Hz, 0.66H for **(2*S*,3*S*)-3ak**), 5.84 (dd, *J* = 4.6, 8.8 Hz, 0.30H for **(2*R*,3*S*)-3ak**), 5.22 (t, *J* = 6.5 Hz, 0.66H for **(2*S*,3*S*)-3ak**), 3.74 (s, 0.66 × 3H for **(2*S*,3*S*)-3ak**), 3.43 (d, *J* = 11.3 Hz, 0.66H for **(2*S*,3*S*)-3ak**), 3.33 (s, 0.30 × 3H for **(2*R*,3*S*)-3ak**), 3.26-3.18 (m, 0.66H for **(2*S*,3*S*)-3ak**), 3.13 (d, *J* = 11.4 Hz, 0.30H for **(2*R*,3*S*)-3ak**), 3.09-3.03 (m, 0.30H for **(2*R*,3*S*)-3ak**), 2.96-2.89 (m, 0.30H for **(2*R*,3*S*)-3ak**), 2.76-2.70 (m, 0.30H for **(2*R*,3*S*)-3ak**), 2.67-2.60 (m, 0.66H for **(2*S*,3*S*)-3ak**), 2.55-2.49 (m, 0.66H for **(2*S*,3*S*)-3ak**), 2.43-2.35 (m, 0.30H for **(2*R*,3*S*)-3ak**), 2.35 (s, 0.30 × 3H for **(2*R*,3*S*)-3ak**), 2.29-2.16 [(m, 0.30H for **(2*R*,3*S*)-3ak** and 0.66H for **(2*S*,3*S*)-3ak**)], 2.20 (s, 0.66 × 3H for **(2*S*,3*S*)-3ak**), 1.99-1.90 (m, 0.66H for **(2*S*,3*S*)-3ak**), 1.21 (d, *J* = 6.8 Hz, 0.66 × 3H for **(2*S*,3*S*)-3ak**), 1.05 (d, *J* = 6.7 Hz, 0.30 × 3H for **(2*R*,3*S*)-3ak**); ^{13}C{^{1}H} NMR (CDCl$_3$, 100 MHz) for **(2*R*,3*S*)-3ak** and **(2*S*,3*S*)-3ak**: δ 171.6, 171.2, 154.1, 153.3, 146.0, 145.6, 144.6, 143.5, 134.8, 134.6, 128.6, 128.3, 127.8, 127.7 (2C), 127.5, 126.7, 126.62, 126.56, 126.49, 126.44, 126.3, 126.22,

126.19, 125.9 (2C), 125.4, 125.1, 124.9, 124.8, 124.7, 124.4, 122.3 (2C), 120.7, 120.2, 107.3, 107.1, 74.5, 73.0, 72.7, 71.6, 52.0, 51.4, 51.0, 50.5, 39.4, 39.2, 37.8, 37.0, 36.3, 36.2, 19.8, 18.6; HRMS (APCI) m/z ([M+H]$^+$) calcd for $C_{29}H_{32}NO_3S$: 474.2097, found: 474.2097.

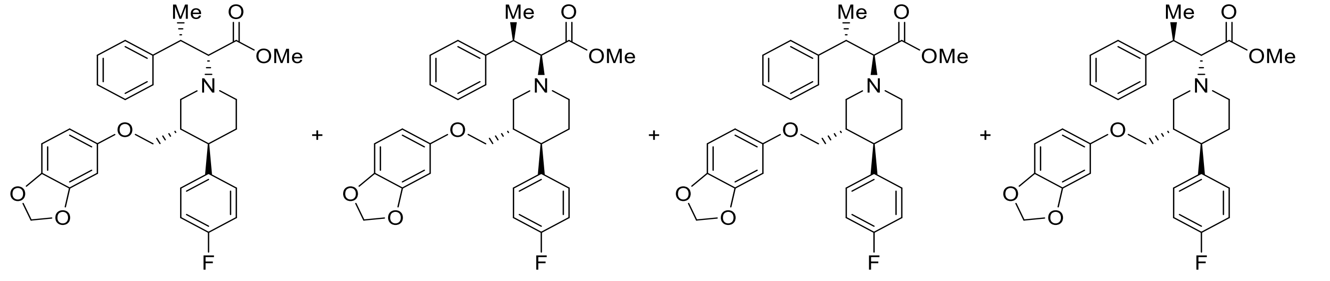

(2*R*,3*S*)-3al **(2*S*,3*R*)-3al** **(2*S*,3*S*)-3al** **(2*R*,3*R*)-3al**

A 31:2:64:3 diastereomixture of Methyl (2*R*,3*S*)-2-((3*S*,4*R*)-3-((benzo[*d*][1,3]dioxol-5-yloxy)methyl)-4-(4-fluorophenyl)piperidin-1-yl)-3-phenylbutanoate ((2*R*,3*S*)-3al) and Methyl (2*S*,3*R*)-2-((3*S*,4*R*)-3-((benzo[*d*][1,3]dioxol-5-yloxy)methyl)-4-(4-fluorophenyl)piperidin-1-yl)-3-phenylbutanoate ((2*S*,3*R*)-3al) and Methyl (2*S*,3*S*)-2-((3*S*,4*R*)-3-((benzo[*d*][1,3]dioxol-5-yloxy)methyl)-4-(4-fluorophenyl)piperidin-1-yl)-3-phenylbutanoate ((2*S*,3*S*)-3al) and Methyl (2*R*,3*S*)-2-((3*S*,4*R*)-3-((benzo[*d*][1,3]dioxol-5-yloxy)methyl)-4-(4-fluorophenyl)piperidin-1-yl)-3-phenylbutanoate ((2*R*,3*R*)-3al)

It was purified by silica gel column chromatography with hexane/ethyl acetate (10/1, v/v): 52.3 mg (69%, 0.15 mmol scale); colorless oil; ^{1}H NMR ($CDCl_3$, 400 MHz) for **(2*R*,3*S*)-3al** and **(2*S*,3*S*)-3al**: δ 7.35-7.16 [(m, 0.31 × 7H for **(2*R*,3*S*)-3al** and 0.64 × 5H for **(2*S*,3*S*)-3al**)], 7.00-6.96 [(m, 0.31 × 2H for **(2*R*,3*S*)-3al** and 0.64 × 2H for **(2*S*,3*S*)-3al**)], 6.89 (t, *J* = 8.7 Hz, 0.64 × 2H for **(2*S*,3*S*)-3al**), 6.64 (d, *J* = 8.5 Hz, 0.31H for **(2*R*,3*S*)-3al**), 6.61 (d, *J* = 8.5 Hz, 0.64H for **(2*S*,3*S*)-3al**), 6.37 (d, *J* = 2.4 Hz, 0.31H for **(2*R*,3*S*)-3al**), 6.31 (d, *J* = 2.5 Hz, 0.64H for **(2*S*,3*S*)-3al**), 6.16 (dd, *J* = 2.5, 8.5 Hz, 0.31H for **(2*R*,3*S*)-3al**), 6.09 (dd, *J* = 2.5, 8.5 Hz, 0.64H for **(2*S*,3*S*)-3al**), 5.89 (s, 0.31 × 2H for **(2*R*,3*S*)-3al**), 5.87 (s, 0.64 × 2H for **(2*S*,3*S*)-3al**), 3.76 (s, 0.64 × 3H for **(2*S*,3*S*)-3al**), 3.58 (dd, *J* = 2.9, 9.3 Hz, 0.31H for **(2*R*,3*S*)-3al**), 3.50-3.22 [(m, 0.31 × 4H for **(2*R*,3*S*)-3al** and 0.64 × 4H for **(2*S*,3*S*)-3al**)], 3.41 (s, 0.31 × 3H for **(2*R*,3*S*)-3al**), 3.09-3.06 (m, 0.64H for **(2*S*,3*S*)-3al**), 3.01-2.98 (m, 0.64H for **(2*S*,3*S*)-3al**), 2.96-2.93 (m, 0.31H for **(2*R*,3*S*)-3al**), 2.52-2.41 (m, 0.64H for **(2*S*,3*S*)-3al**), 2.35-2.13 [(m, 0.31 × 3H for **(2*R*,3*S*)-3al** and 0.64 × 2H for **(2*S*,3*S*)-3al**)], 1.92-1.81 (m, 0.64 × 2H for **(2*S*,3*S*)-3al**), 1.62-1.57

(m, 0.31 × 2H for **(2*R*,3*S*)-3al**), 1.38 (d, *J* = 6.8 Hz, 0.31 × 3H for ***syn*-3al**), 1.28-1.18 [(m, 0.31H for **(2*R*,3*S*)-3al** and 0.64H for **(2*S*,3*S*)-3al**)], 1.22 (d, *J* = 6.8 Hz, 0.64 × 3H for ***anti*-3al**); $^{13}C\{^1H\}$ NMR ($CDCl_3$, 100 MHz) for **(2*R*,3*S*)-3al** and **(2*S*,3*S*)-3al**: δ 172.1, 171.1, 161.6 (d, *J* = 242.9 Hz), 161.5 (d, *J* = 242.8 Hz), 154.5 (2C), 148.2 (2C), 144.3, 143.7, 141.7, 141.6, 139.9 (d, *J* = 2.9 Hz), 139.8 (d, *J* = 2.9 Hz), 128.9 (d, *J* = 7.5 Hz), 128.8 (d, *J* = 7.5 Hz), 128.5, 128.2, 128.0, 127.7, 126.8, 126.3, 115.5 (d, *J* = 20.7 Hz), 115.4 (d, *J* = 20.7 Hz), 107.9 (2C), 105.8, 105.6, 101.2 (2C), 98.2, 98.0, 74.1, 73.5, 69.8, 69.6, 57.1, 54.2, 51.1, 50.6, 50.1, 47.1, 44.7, 44.4, 42.9, 42.5, 39.1, 39.0, 35.3, 34.6, 20.1, 18.9; $^{19}F\{^1H\}$ NMR ($CDCl_3$, 376 MHz): δ −116.62 (s), −116.80 (s); HRMS (APCI) m/z ($[M+H]^+$) calcd for $C_{30}H_{33}FNO_5$: 506.2337, found: 506.2336.

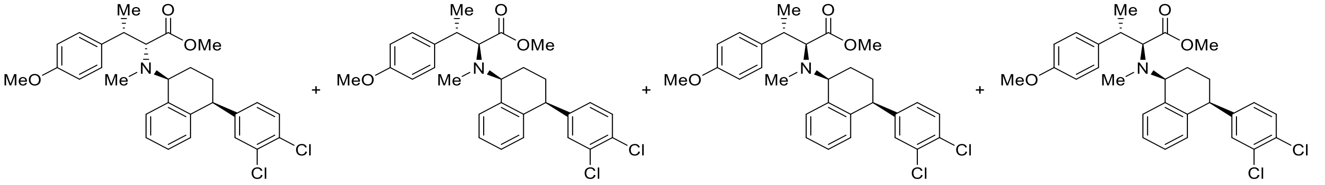

A 31:2:64:3 diastereomixture of Methyl (2*R*,3*S*)-2-(((1*S*,4*S*)-4-(3,4-dichlorophenyl)-1,2,3,4-tetrahydronaphthalen-1-yl)(methyl)amino)-3-(4-methoxyphenyl)butanoate ((2*R*,3*S*)-3bm) and Methyl (2*S*,3*R*)-2-(((1*S*,4*S*)-4-(3,4-dichlorophenyl)-1,2,3,4-tetrahydronaphthalen-1-yl)(methyl)amino)-3-(4-methoxyphenyl)butanoate ((2*S*,3*R*)-3bm) and Methyl (2*S*,3*S*)-2-(((1*S*,4*S*)-4-(3,4-dichlorophenyl)-1,2,3,4-tetrahydronaphthalen-1-yl)(methyl)amino)-3-(4-methoxyphenyl)butanoate ((2*S*,3*S*)-3bm) and Methyl (2*R*,3*R*)-2-(((1*S*,4*S*)-4-(3,4-dichlorophenyl)-1,2,3,4-tetrahydronaphthalen-1-yl)(methyl)amino)-3-(4-methoxyphenyl)butanoate ((2*R*,3*R*)-3bm)

It was purified by silica gel column chromatography with hexane/ethyl acetate (10/1, v/v) and GPC ($CHCl_3$): 32.3 mg (42%, 0.15 mmol scale); colorless oil; ^1H NMR ($CDCl_3$, 400 MHz) for **(2*R*,3*S*)-3bm** and **(2*S*,3*S*)-3bm**: δ 7.57 (d, *J* = 7.6 Hz, 0.31H for **(2*R*,3*S*)-3bm**), 7.30 (d, *J* = 8.2 Hz, 0.31H for **(2*R*,3*S*)-3bm**), 7.27-7.24 (m, 0.31H for **(2*R*,3*S*)-3bm**), 7.26 (d, *J* = 8.3 Hz, 0.64H for **(2*S*,3*S*)-3bm**), 7.21 (d, *J* = 8.7 Hz, 0.64 × 2H for **(2*S*,3*S*)-3bm**), 7.18 (t, *J* = 7.5 Hz, 0.31H for **(2*R*,3*S*)-3bm**), 7.14 (d, *J* = 2.0 Hz, 0.31H for **(2*R*,3*S*)-3bm**), 7.07 (d, *J* = 8.7 Hz, 0.31 × 2H for **(2*R*,3*S*)-3bm**), 7.00 (t, *J* = 7.8 Hz, 0.64H for **(2*S*,3*S*)-3bm**), 6.98 (d, *J* = 1.8 Hz, 0.64H for **(2*S*,3*S*)-3bm**), 6.94 (d, *J* = 8.7 Hz, 0.64 × 2H for **(2*S*,3*S*)-3bm**), 6.91 (dd, *J* = 2.0, 8.2 Hz, 0.31H for **(2*R*,3*S*)-3bm**), 6.90 (d, *J* = 7.6 Hz, 0.31H for **(2*R*,3*S*)-3bm**), 6.85 (t, *J* = 7.9 Hz, 0.64H for **(2*S*,3*S*)-3bm**), 6.79 (d, *J* = 8.8 Hz, 0.31 × 2H for

(**2*R*,3*S*)-3bm**), 6.76 (d, *J* = 7.9 Hz, 0.64H for (**2*S*,3*S*)-3bm**), 6.70 (dd, *J* = 1.8, 8.3 Hz, 0.64H for (**2*S*,3*S*)-3bm**), 6.31 (d, *J* = 7.9 Hz, 0.64H for (**2*S*,3*S*)-3bm**), 4.10 (t, *J* = 5.8 Hz, 0.31H for (**2*R*,3*S*)-3bm**), 4.04-4.03 (m, 0.64H for (**2*S*,3*S*)-3bm**), 3.99 (t, *J* = 6.4 Hz, 0.31H for (**2*R*,3*S*)-3bm**), 3.86 (s, 0.64 × 3H for (**2*S*,3*S*)-3bm**), 3.83-3.80 (m, 0.64H for (**2*S*,3*S*)-3bm**), 3.77 (s, 0.31 × 3H for (**2*R*,3*S*)-3bm**), 3.72 (s, 0.64 × 3H for (**2*S*,3*S*)-3bm**), 3.55 (d, *J* = 11.3 Hz, 0.64H for (**2*S*,3*S*)-3bm**), 3.42 (d, *J* = 11.2 Hz, 0.31H for (**2*R*,3*S*)-3bm**), 3.29-3.18 [(m, 0.31H for (**2*R*,3*S*)-3bm** and 0.64H for (**2*S*,3*S*)-3bm**)], 3.22 (s, 0.31 × 3H for (**2*R*,3*S*)-3bm**), 2.54 (s, 0.31 × 3H for (**2*R*,3*S*)-3bm**), 2.14-2.01 (m, 0.64 × 2H for (**2*S*,3*S*)-3bm**), 2.11 (s, 0.64 × 3H for (**2*S*,3*S*)-3bm**), 1.92-1.86 (m, 0.31 × 2H for (**2*R*,3*S*)-3bm**), 1.78-1.73 (m, 0.64H for (**2*S*,3*S*)-3bm**), 1.65-1.58 (m, 0.31 × 2H for (**2*R*,3*S*)-3bm**), 1.51-1.45 (m, 0.64H for (**2*S*,3*S*)-3bm**), 1.37 (d, *J* = 7.0 Hz, 0.31 × 3H for (**2*R*,3*S*)-3bm**), 1.20 (d, *J* = 6.9 Hz, 0.64 × 3H for (**2*S*,3*S*)-3bm**); $^{13}C\{^{1}H\}$ NMR ($CDCl_3$, 100 MHz) for (**2*R*,3*S*)-3bm** and (**2*S*,3*S*)-3bm**: δ 174.2, 173.6, 158.5, 158.3, 147.9, 147.5, 139.3, 139.0, 138.5, 137.8, 136.9, 135.6, 132.21, 132.17, 131.0, 130.8, 130.7, 130.1, 130.00, 129.98, 129.93, 129.90, 129.6, 129.0, 128.52, 128.45, 128.4, 128.2, 127.4, 127.0, 126.8, 126.5, 114.1, 113.8, 73.6, 68.1, 64.6, 61.2, 55.6, 55.3, 51.4, 50.6, 44.2, 43.2, 39.6, 39.5, 35.0, 30.3, 29.8, 28.3, 21.5, 19.5, 19.4, 18.0; HRMS (APCI) m/z ($[M+H]^+$) calcd for $C_{29}H_{32}Cl_2NO_3$: 512.1754, found: 512.1746.

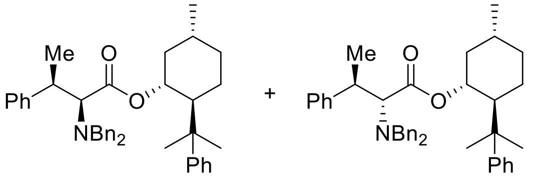

***syn*-3Ga** ***anti*-3Ga**

A 9:91 diastereomixture of (1*R*,2*S*,5*R*)-5-methyl-2-(2-phenylpropan-2-yl)cyclohexyl (2*S*,3*R*)-2-(dibenzylamino)-3-phenylbutanoate (*syn*-3Ga) and (1*R*,2*S*,5*R*)-5-methyl-2-(2-phenylpropan-2-yl)cyclohexyl (2*R*,3*R*)-2-(dibenzylamino)-3-phenylbutanoate (*anti*-3Ga)

It was purified by silica gel column chromatography with hexane/ethyl acetate (20/1, v/v) and GPC ($CHCl_3$): 55.9 mg (65%, 0.15 mmol scale); colorless oil; ^1H NMR ($CDCl_3$, 400 MHz): δ 7.44 (d, *J* = 7.4 Hz, 0.09 × 4H for ***syn*-3Ga**), 7.36-7.13 [(m, 0.09 × 14H for ***syn*-3Ga** and 0.91 × 14H for ***anti*-3Ga**)], 7.08-7.05 (m, 0.09 × 2H for ***syn*-3Ga**), 6.90-6.88 (m, 0.91 × 4H for ***anti*-3Ga**), 6.85-6.82 (m, 0.91 × 2H for ***anti*-3Ga**), 5.00 (td, *J* = 4.1, 10.6 Hz, 0.91H for ***anti*-3Ga**), 4.61 (td, *J* = 4.1, 10.5 Hz, 0.09H for ***syn*-3Ga**), 4.21 (d, *J* = 14.8 Hz, 0.09 × 2H for ***syn*-3Ga**), 3.82 (d, *J* = 13.7 Hz, 0.91 × 2H for ***anti*-3Ga**), 3.76 (d, *J* = 14.8 Hz, 0.09 × 2H for ***syn*-3Ga**), 3.57 (d, *J* = 11.0 Hz, 0.09H for ***syn*-3Ga**), 3.38-3.30 [(m,

0.09H for ***syn*-3Ga** and 0.91H for ***anti*-3Ga**)], 3.24 (d, *J* = 13.7 Hz, 0.91 × 2H for ***anti*-3Ga**), 3.16 (d, *J* = 11.4 Hz, 0.91H for ***anti*-3Ga**), 2.33-2.29 (m, 0.91H for ***anti*-3Ga**), 2.16 (td, *J* = 3.2, 11.3 Hz, 0.91H for ***anti*-3Ga**), 1.66-1.57 [(m, 0.09H for ***syn*-3Ga** and 0.91 × 2H for ***anti*-3Ga**)], 1.49-1.43 [(m, 0.09H for ***syn*-3Ga** and 0.91H for ***anti*-3Ga**)], 1.45 (s, 0.09 × 3H for ***syn*-3Ga**), 1.38 (s, 0.91 × 3H for ***anti*-3Ga**), 1.30-1.20 [(m, 0.09 × 4H for ***syn*-3Ga** and 0.91H for ***anti*-3Ga**)], 1.28 (s, 0.91 × 3H for ***anti*-3Ga**), 1.22 (s, 0.09 × 3H for ***syn*-3Ga**), 1.12-1.02 [(m, 0.09 × 4H for ***syn*-3Ga** and 0.91H for ***anti*-3Ga**)], 1.09 (d, *J* = 6.7 Hz, 0.91 × 3H for ***anti*-3Ga**), 0.99-0.88 [(m, 0.09H for ***syn*-3Ga** and 0.91H for ***anti*-3Ga**)], 0.99 (d, *J* = 6.3 Hz, 0.91 × 3H for ***anti*-3Ga**), 0.64 (d, *J* = 6.5 Hz, 0.09 × 3H for ***syn*-3Ga**); $^{13}C\{^1H\}$ NMR ($CDCl_3$, 100 MHz): δ 172.7, 170.6, 151.0, 150.7, 143.9, 143.5, 140.0, 139.0, 129.4, 128.70, 128.66, 128.5, 128.41, 128.39, 128.2, 128.13, 128.11, 128.0, 127.0 (2C), 126.9, 126.3, 125.9, 125.7, 125.44, 125.38, 76.4, 76.0, 67.6, 65.8, 55.0, 54.3, 50.7, 50.2, 43.7 (2C), 41.4, 40.5, 40.3, 39.3, 34.8, 34.4, 31.7, 31.4, 31.2, 28.9, 27.5, 27.4, 25.2, 23.3, 22.1, 21.7, 21.2, 20.7; HRMS (APCI) m/z ($[M+H]^+$) calcd for $C_{40}H_{48}NO_2$: 574.3680, found: 574.3692.

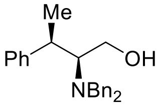

***syn*-5**

(2*S*,3*R*)-2-(dibenzylamino)-3-phenylbutan-1-ol (*syn*-5)

It was purified by silica gel column chromatography with hexane/ethyl acetate (10/1, v/v): 1.0 mg (3%, 0.097 mmol scale); colorless oil; ^1H NMR ($CDCl_3$, 400 MHz): δ 7.35-7.23 (m, 12H), 7.17 (t, *J* = 7.2 Hz, 1H), 7.10-7.08 (m, 2H), 3.91 (s, 4H), 3.28 (dd, *J* = 8.5, 11.0 Hz, 1H), 3.18-3.15 (m, 1H), 3.08-3.01 (m, 1H), 2.98 (qd, *J* = 4.0, 8.5 Hz, 1H), 2.40 (br, 1H), 1.49 (d, *J* = 6.7 Hz, 3H); $^{13}C\{^1H\}$ NMR ($CDCl_3$, 100 MHz): δ 145.0, 139.9, 129.4, 128.8, 128.6, 127.5, 127.4, 126.7, 64.5, 60.6, 54.6, 41.3, 21.8; HRMS (APCI) m/z ($[M+H]^+$) calcd for $C_{24}H_{28}NO$: 346.2165, found: 346.2164.

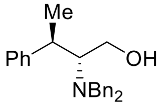

***anti*-5**

(2*R*,3*R*)-2-(dibenzylamino)-3-phenylbutan-1-ol (*anti*-5)

It was purified by silica gel column chromatography with hexane/ethyl acetate (10/1, v/v): 23.8 mg (71%, 0.097 mmol scale); colorless oil; ^1H NMR ($CDCl_3$, 400 MHz): δ 7.40 (t, *J* = 7.6 Hz, 2H), 7.34-7.23 (m, 5H), 7.24 (d, *J* = 7.2 Hz, 2H), 7.20 (t, *J* = 7.3 Hz, 2H), 7.07-7.05 (m, 4H), 3.75 (dd, *J* =

4.6, 10.5 Hz, 1H), 3.75 (d, J = 13.3 Hz, 2H), 3.55 (t, J = 9.8 Hz, 1H), 3.31 (br, 1H), 3.26 (d, J = 13.3 Hz, 2H), 3.19-3.12 (m, 1H), 3.09 (qd, J = 4.5, 9.6 Hz, 1H), 1.16 (d, J = 6.8 Hz, 3H); $^{13}C\{^1H\}$ NMR ($CDCl_3$, 100 MHz): δ 146.5, 139.5, 129.3, 128.8, 128.5, 128.2, 127.2, 126.8, 63.7, 59.6, 53.4, 39.5, 20.0; HRMS (APCI) m/z ($[M+H]^+$) calcd for $C_{24}H_{28}NO$: 346.2165, found: 346.2167.

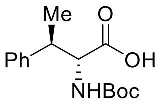

***anti*-6**

(2*R*,3*R*)-2-((*tert*-butoxycarbonyl)amino)-3-phenylbutanoic acid (*anti*-6)

It was purified by silica gel column chromatography with hexane/ethyl acetate (1/1, v/v): 12.2 mg (63% in 3 steps, 0.054 mmol scale); colorless oil; The spectra date were matched with the reported values.[S12] CHIRAL ART Amylose-SA (3 μm) column, 94/6 (hexane + 0.1vol% TFA)/isopropyl alcohol, 0.3 mL/min, major isomer: t_R = 31.3 min, minor isomers: t_R = 56.5 min.

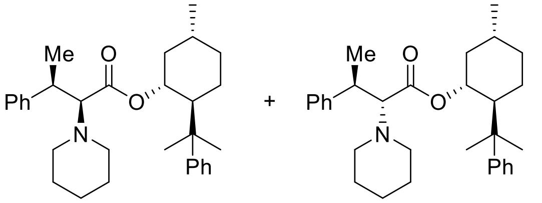

***syn*-3Gd** ***anti*-3Gd**

A 5:95 diastereomixture of (1*R*,2*S*,5*R*)-5-methyl-2-(2-phenylpropan-2-yl)cyclohexyl (2*S*,3*R*)-3-phenyl-2-(piperidin-1-yl)butanoate (*syn*-3Gd) and (1*R*,2*S*,5*R*)-5-methyl-2-(2-phenylpropan-2-yl)cyclohexyl (2*R*,3*R*)-3-phenyl-2-(piperidin-1-yl)butanoate (*anti*-3Gd)

It was purified by silica gel column chromatography with hexane/ethyl acetate (20/1, v/v) and GPC ($CHCl_3$): 61.6 mg (89%, 0.15 mmol scale); colorless oil; ^1H NMR ($CDCl_3$, 400 MHz): δ 7.37-7.32 (m, 0.95 × 4H for ***anti*-3Gd**), 7.36-7.13 [(m, 0.05 × 10H for ***syn*-3Gd** and 0.95 × 3H for ***anti*-3Gd**)], 7.16 (t, J = 7.3 Hz, 0.95H for ***anti*-3Gd**), 7.04 (d, J = 7.0 Hz, 0.95 × 2H for ***anti*-3Gd**), 4.76 (td, J = 4.1, 10.7 Hz, 0.95H for ***anti*-3Gd**), 4.43 (td, J = 4.3, 10.7 Hz, 0.05H for ***syn*-3Gd**), 3.15-3.07 [(m, 0.05H for ***syn*-3Gd** and 0.95H for ***anti*-3Gd**)], 2.77-2.72 (m, 0.05H for ***syn*-3Gd**), 2.67-2.63 (m, 0.05H for ***syn*-3Gd**), 2.45-2.41 (m, 0.95 × 2H for ***anti*-3Gd**), 2.36 [(d, J = 11.2 Hz, 0.05H for ***syn*-3Gd** and 0.95H for ***anti*-3Gd**)], 2.20-2.13 [(m, 0.05H for ***syn*-3Gd** and 0.95H for ***anti*-3Gd**)], 2.10-2.06 [(m, 0.05H for ***syn*-3Gd** and 0.95H for ***anti*-3Gd**)], 1.92-1.87 [(m, 0.05 × 2H for ***syn*-3Gd** and 0.95 × 2H for

***anti*-3Gd**)], 1.76-1.66 [(m, 0.05 × 4H for ***syn*-3Gd** and 0.95 × 2H for ***anti*-3Gd**)], 1.58-1.49 (m, 0.95 × 2H for ***anti*-3Gd**), 1.34 (s, 0.95 × 3H for ***anti*-3Gd**), 1.32 (s, 0.05 × 3H for ***syn*-3Gd**), 1.21 [(s, 0.05 × 3H for ***syn*-3Gd** and 0.95 × 3H for ***anti*-3Gd**)], 1.18-0.94 [(m, 0.05 × 11H for ***syn*-3Gd** and 0.95 × 8H for ***anti*-3Gd**)], 1.13 (d, J = 6.8 Hz, 0.95 × 3H for ***anti*-3Gd**), 0.91 (d, J = 6.5 Hz, 0.95 × 3H for ***anti*-3Gd**), 0.61 (d, J = 6.5 Hz, 0.05 × 3H for ***syn*-3Gd**); $^{13}C\{^{1}H\}$ NMR ($CDCl_3$, 100 MHz): for ***anti*-3Gd** δ 170.3, 152.4, 145.3, 128.2, 127.84, 127.80, 125.7, 125.6, 125.0, 75.4, 72.6, 50.8, 50.3, 43.0, 39.7, 38.7, 34.9, 31.6, 28.6, 26.7, 26.5, 24.8, 24.6, 22.1, 20.5; HRMS (APCI) m/z ([M+H]$^+$) calcd for $C_{31}H_{44}NO_2$: 462.3367, found: 462.3367.

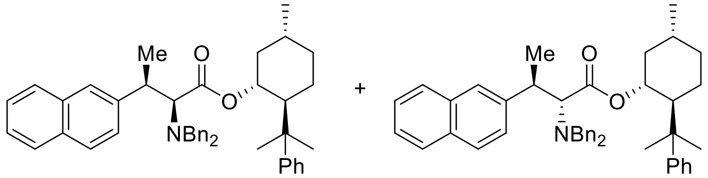

***syn*-3Ha** ***anti*-3Ha**

An 8:92 diastereomixture of (1*R*,2*S*,5*R*)-5-methyl-2-(2-phenylpropan-2-yl)cyclohexyl (2*S*,3*R*)-2-(dibenzylamino)-3-(naphthalen-2-yl)butanoate (*syn*-3Ha) and (1*R*,2*S*,5*R*)-5-methyl-2-(2-phenylpropan-2-yl)cyclohexyl (2*R*,3*R*)-2-(dibenzylamino)-3-(naphthalen-2-yl)butanoate (*anti*-3Ha)

It was purified by silica gel column chromatography with hexane/ethyl acetate (20/1, v/v) and GPC ($CHCl_3$): 57.1 mg (61%, 0.15 mmol scale); colorless oil; ^{1}H NMR ($CDCl_3$, 400 MHz): δ 7.89-7.87 (m, 0.92H for ***anti*-3Ha**), 7.75-7.65 [(m, 0.08 × 4H for ***syn*-3Ha** and 0.92H for ***anti*-3Ha**)], 7.72 (d, J = 8.4 Hz, 0.92H for ***anti*-3Ha**), 7.54-7.44 [(m, 0.08 × 8H for ***syn*-3Ha** and 0.92 × 2H for ***anti*-3Ha**)], 7.42-7.39 (m, 0.08 × 4H for ***syn*-3Ha**), 7.34 (t, J = 7.5 Hz, 0.92 × 2H for ***anti*-3Ha**), 7.32 (s, 0.92H for ***anti*-3Ha**), 7.28 (t, J = 7.4 Hz, 0.92 × 2H for ***anti*-3Ha**), 7.21-7.13 [(m, 0.08 × 6H for ***syn*-3Ha** and 0.92H for ***anti*-3Ha**)], 7.16 (t, J = 7.7 Hz, 0.92 × 2H for ***anti*-3Ha**), 7.08 (t, J = 7.5 Hz, 0.92 × 4H for ***anti*-3Ha**), 6.92 (d, J = 8.5 Hz, 0.92H for ***anti*-3Ha**), 6.81 (d, J = 7.2 Hz, 0.92 × 4H for ***anti*-3Ha**), 5.02 (td, J = 4.1, 10.7 Hz, 0.92H for ***anti*-3Ha**), 4.52 (td, J = 4.2, 10.6 Hz, 0.08H for ***syn*-3Ha**), 4.26 (d, J = 14.6 Hz, 0.08 × 2H for ***syn*-3Ha**), 3.84 (d, J = 13.8 Hz, 0.92 × 2H for ***anti*-3Ha**), 3.80 (d, J = 14.8 Hz, 0.08 × 2H for ***syn*-3Ha**), 3.67 (d, J = 10.8 Hz, 0.08H for ***syn*-3Ha**), 3.54-3.46 [(m, 0.08H for ***syn*-3Ha** and 0.92H for ***anti*-3Ha**)], 3.259 (d, J = 11.3 Hz, 0.92H for ***anti*-3Ha**), 3.256 (d, J = 13.8 Hz, 0.92 × 2H for ***anti*-3Ha**), 2.33-2.30 (m, 0.92H for ***anti*-3Ha**), 2.18 (td, J = 3.3, 11.9 Hz, 0.92H for ***anti*-3Ha**), 1.67-1.58 [(m, 0.08 × 2H for ***syn*-3Ha** and 0.92 × 2H for ***anti*-3Ha**)], 1.52-1.47 [(m, 0.08H for ***syn*-3Ha** and 0.92H for ***anti*-3Ha**)], 1.52 (s, 0.08 × 3H for ***syn*-3Ha**), 1.41 (s, 0.92 × 3H for ***anti*-3Ha**), 1.29-1.23

[(m, 0.08 × 6H for ***syn*-3Ha** and 0.92H for ***anti*-3Ha**)], 1.29 (s, 0.92 × 3H for ***anti*-3Ha**), 1.16-1.04 [(m, 0.08 × 4H for ***syn*-3Ha** and 0.92H for ***anti*-3Ha**)], 1.15 (d, *J* = 6.8 Hz, 0.92 × 3H for ***anti*-3Ha**), 0.99-0.91 [(m, 0.08H for ***syn*-3Ha** and 0.92H for ***anti*-3Ha**)], 0.99 (d, *J* = 6.3 Hz, 0.92 × 3H for ***anti*-3Ha**), 0.07 (d, *J* = 6.5 Hz, 0.08 × 3H for ***syn*-3Ha**); $^{13}C\{^{1}H\}$ NMR ($CDCl_3$, 100 MHz): δ 172.7, 170.6, 151.1, 150.7, 141.6, 140.8, 140.0, 138.9, 133.6, 133.5, 132.8, 132.5, 129.3, 128.7, 128.4, 128.3, 128.2, 128.1, 128.0, 127.84, 127.77, 127.74, 127.68, 127.62, 127.5, 127.0, 126.9 (2C), 126.5, 126.4, 126.0, 125.84, 125.75 (2C), 125.6, 125.4 (2C), 125.3, 76.4, 75.9, 67.8, 65.9, 55.1, 54.5, 50.6, 50.1, 43.8, 41.6, 40.4, 40.3, 40.1, 39.4, 34.8, 34.2, 31.7, 31.4, 30.8, 28.8, 27.4, 25.4, 24.8, 23.3, 22.1, 21.02, 20.95, 20.5; HRMS (APCI) m/z ($[M+H]^+$) calcd for $C_{44}H_{50}NO_2$: 624.3836, found: 624.3859.

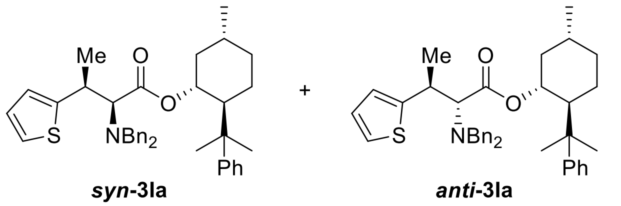

***syn*-3Ia** ***anti*-3Ia**

An 8:92 diastereomixture of (1*R*,2*S*,5*R*)-5-methyl-2-(2-phenylpropan-2-yl)cyclohexyl (2*S*,3*S*)-2-(dibenzylamino)-3-(thiophen-2-yl)butanoate (*syn*-3Ia) and (1*R*,2*S*,5*R*)-5-methyl-2-(2-phenylpropan-2-yl)cyclohexyl (2*R*,3*S*)-2-(dibenzylamino)-3-(thiophen-2-yl)butanoate (*anti*-3Ia)

It was purified by silica gel column chromatography with hexane/ethyl acetate (20/1, v/v) and GPC ($CHCl_3$): 57.4 mg (66%, 0.15 mmol scale); colorless oil; ^{1}H NMR ($CDCl_3$, 400 MHz): δ 7.39 (d, *J* = 7.2 Hz, 0.08 × 4H for ***syn*-3Ia**), 7.34-7.09 [(m, 0.08 × 12H for ***syn*-3Ia** and 0.92 × 11H for ***anti*-3Ia**)], 7.11 (t, *J* = 7.1 Hz, 0.92H for ***anti*-3Ia**), 7.02-7.00 (m, 0.92 × 4H for ***anti*-3Ia**), 6.93 (dd, *J* = 3.4, 5.1 Hz, 0.92H for ***anti*-3Ia**), 6.83 (dd, *J* = 3.4, 5.1 Hz, 0.08H for ***syn*-3Ia**), 6.72 (d, *J* = 3.6 Hz, 0.08H for ***syn*-3Ia**), 6.55 (d, *J* = 3.5 Hz, 0.92H for ***anti*-3Ia**), 4.96 (td, *J* = 4.1, 10.7 Hz, 0.92H for ***anti*-3Ia**), 4.72 (td, *J* = 4.1, 10.6 Hz, 0.08H for ***syn*-3Ia**), 4.11 (d, *J* = 14.5 Hz, 0.08 × 2H for ***syn*-3Ia**), 3.83 (d, *J* = 13.7 Hz, 0.92 × 2H for ***anti*-3Ia**), 3.71 (d, *J* = 14.6 Hz, 0.08 × 2H for ***syn*-3Ia**), 3.67-3.59 [(m, 0.08H for ***syn*-3Ha** and 0.92H for ***anti*-3Ha**)], 3.49 (d, *J* = 10.4 Hz, 0.08H for ***syn*-3Ia**), 3.26 (d, *J* = 13.8 Hz, 0.92 × 2H for ***anti*-3Ia**), 3.03 (d, *J* = 11.1 Hz, 0.92H for ***anti*-3Ia**), 2.29-2.25 (m, 0.92H for ***anti*-3Ia**), 2.14 (td, *J* = 3.3, 11.4 Hz, 0.92H for ***anti*-3Ia**), 1.77-1.56 [(m, 0.08 × 2H for ***syn*-3Ia** and 0.92 × 2H for ***anti*-3Ia**)], 1.50 [(dq, *J* = 3.2, 13.4 Hz, 0.08H for ***syn*-3Ia** and 0.92H for ***anti*-3Ia**)], 1.45 (d, *J* = 6.8 Hz, 0.08 × 3H for ***syn*-3Ia**), 1.34 (s, 0.92 × 3H for ***anti*-3Ia**), 1.32 (s, 0.08 × 3H for ***syn*-3Ia**), 1.28 (s, 0.08 × 3H for ***syn*-3Ia**), 1.25 (s, 0.92 × 3H for ***anti*-3Ia**), 1.25-1.16 [(m, 0.08 × 3H for ***syn*-3Ia** and 0.92H for

***anti*-3Ia**)], 1.16 (d, J = 6.8 Hz, 0.92 × 3H for ***anti*-3Ia**), 1.07 [(qd, J = 3.1, 13.2 Hz, 0.08H for ***syn*-3Ia** and 0.92H for ***anti*-3Ia**)], 0.97 (d, J = 6.4 Hz, 0.92 × 3H for ***anti*-3Ia**), 0.98-0.90 [(m, 0.08H for ***syn*-3Ia** and 0.92H for ***anti*-3Ia**)], 0.72 (d, J = 6.5 Hz, 0.08 × 3H for ***syn*-3Ia**); $^{13}C\{^{1}H\}$ NMR ($CDCl_3$, 100 MHz): δ 172.2, 170.3, 151.1, 150.7, 147.5, 147.1, 139.7, 139.0, 129.3, 128.8, 128.4, 128.2 (2C), 128.0, 127.1, 126.9, 126.4, 126.3, 125.9, 125.6, 125.5, 125.4, 125.2, 124.8, 123.5, 122.8, 76.4, 76.2, 68.6, 67.4, 55.1, 54.6, 50.6, 50.3, 43.6, 40.8, 40.5, 40.2, 36.5, 34.8, 34.7, 34.4, 31.7, 31.3, 31.1, 28.3, 27.5, 27.3, 25.7, 23.7, 22.1 (2C), 21.9, 21.8; HRMS (APCI) m/z ([M+H]$^+$) calcd for $C_{38}H_{46}NO_2S$: 580.3244, found: 580.3244.

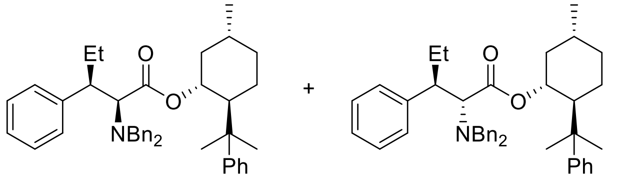

***syn*-3Ja** ***anti*-3Ja**

A 9:91 diastereomixture of (1*R*,2*S*,5*R*)-5-methyl-2-(2-phenylpropan-2-yl)cyclohexyl (2*S*,3*R*)-2-(dibenzylamino)-3-phenylpentanoate (*syn*-3Ja) and (1*R*,2*S*,5*R*)-5-methyl-2-(2-phenylpropan-2-yl)cyclohexyl (2*R*,3*R*)-2-(dibenzylamino)-3-phenylpentanoate (*anti*-3Ja)

It was purified by silica gel column chromatography with hexane/ethyl acetate (20/1, v/v) and GPC ($CHCl_3$): 67.0 mg (76%, 0.15 mmol scale); colorless oil; ^{1}H NMR ($CDCl_3$, 400 MHz): δ 7.42 (d, J = 7.6 Hz, 0.09 × 4H for ***syn*-3Ja**), 7.33-7.13 [(m, 0.09 × 14H for ***syn*-3Ja** and 0.91 × 14H for ***anti*-3Ja**)], 7.01 (d, J = 7.4 Hz, 0.09 × 2H for ***syn*-3Ja**), 6.86 (br, 0.91 × 4H for ***anti*-3Ja**), 6.78 (d, J = 7.2 Hz, 0.91 × 2H for ***anti*-3Ja**), 4.98 (td, J = 4.0, 10.6 Hz, 0.91H for ***anti*-3Ja**), 4.58 (td, J = 4.0, 10.6 Hz, 0.09H for ***syn*-3Ja**), 4.20 (d, J = 14.7 Hz, 0.09 × 2H for ***syn*-3Ja**), 3.78 (br, 0.91 × 2H for ***anti*-3Ja**), 3.74 (d, J = 14.7 Hz, 0.09 × 2H for ***syn*-3Ja**), 3.62 (d, J = 11.0 Hz, 0.09H for ***syn*-3Ja**), 3.222 (d, J = 11.0 Hz, 0.91H for ***anti*-3Ja**), 3.217 (d, J = 13.9 Hz, 0.91 × 2H for ***anti*-3Ja**), 3.09 (td, J = 2.6, 11.0 Hz, 0.91H for ***anti*-3Ja**), 2.98 (td, J = 2.5, 11.0 Hz, 0.09H for ***syn*-3Ja**), 2.33-2.30 (m, 0.91H for ***anti*-3Ja**), 2.15 (td, J = 3.0, 11.6 Hz, 0.91H for ***anti*-3Ja**), 1.65-1.42 [(m, 0.09 × 7H for ***syn*-3Ja** and 0.91 × 4H for ***anti*-3Ja**)], 1.38 (s, 0.91 × 3H for ***anti*-3Ja**), 1.30-1.22 [(m, 0.09 × 6H for ***syn*-3Ja** and 0.91H for ***anti*-3Ja**)], 1.28 (s, 0.91 × 3H for ***anti*-3Ja**), 1.19-1.01 [(m, 0.09 × 5H for ***syn*-3Ja** and 0.91 × 2H for ***anti*-3Ja**)], 0.98-0.90 [(m, 0.09H for ***syn*-3Ja** and 0.91H for ***anti*-3Ja**)], 0.98 (d, J = 6.2 Hz, 0.91 × 3H for ***anti*-3Ja**), 0.66 (t, J = 7.2 Hz, 0.91 × 3H for ***anti*-3Ja**), 0.66-0.62 (m, 0.09 × 3H for ***syn*-3Ja**); $^{13}C\{^{1}H\}$ NMR ($CDCl_3$, 100 MHz): δ 172.7, 170.8, 151.0, 150.8, 141.4, 141.1, 140.1, 139.1, 129.6, 129.5 (2C),

128.7, 128.4, 128.24, 128.18, 128.09, 127.96, 127.92, 127.0, 126.9, 126.3, 125.9, 125.7, 125.44, 125.40, 76.5, 76.0, 67.2, 65.0, 55.1, 54.2, 50.7, 50.2, 48.9, 47.1, 43.7, 40.45, 40.37, 34.8, 34.4, 31.7, 31.4, 31.2, 29.2, 27.7, 27.5, 27.44, 27.39, 26.2, 25.0, 23.4, 22.1, 21.7, 12.3, 11.9 (One sp^2 C signal overlaps with others.); HRMS (APCI) m/z ($[M+H]^+$) calcd for $C_{41}H_{50}NO_2$: 588.3836, found: 588.3846.

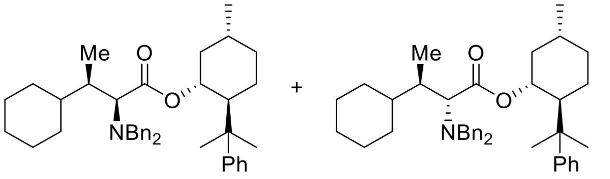

***syn*-3Ka** ***anti*-3Ka**

A 17:83 diastereomixture of (1*R*,2*S*,5*R*)-5-methyl-2-(2-phenylpropan-2-yl)cyclohexyl (2*S*,3*R*)-3-cyclohexyl-2-(dibenzylamino)butanoate (*syn*-3Ka) and (1*R*,2*S*,5*R*)-5-methyl-2-(2-phenylpropan-2-yl)cyclohexyl (2*R*,3*R*)-3-cyclohexyl-2-(dibenzylamino)butanoate (*anti*-3Ka)

It was purified by silica gel column chromatography with hexane/ethyl acetate (20/1, v/v) and GPC ($CHCl_3$): 37.4 mg (43%, 0.15 mmol scale); colorless oil; ^1H NMR ($CDCl_3$, 400 MHz): δ 7.39-7.19 [(m, 0.17 × 14H for ***syn*-3Ka** and 0.83 × 14H for ***anti*-3Ka**)], 7.15-7.09 [(m, 0.17H for ***syn*-3Ka** and 0.83H for ***anti*-3Ka**)], 4.91 [(td, *J* = 4.0, 10.7 Hz, 0.17H for ***syn*-3Ka** and 0.83H for ***anti*-3Ka**)], 4.05 (d, *J* = 14.7 Hz, 0.17 × 2H for ***syn*-3Ka**), 3.88 (d, *J* = 13.6 Hz, 0.83 × 2H for ***anti*-3Ka**), 3.66 (d, *J* = 14.7 Hz, 0.17 × 2H for ***syn*-3Ka**), 3.30 (d, *J* = 11.0 Hz, 0.17H for ***syn*-3Ka**), 3.26 (d, *J* = 13.6 Hz, 0.83 × 2H for ***anti*-3Ka**), 2.81 (d, *J* = 11.4 Hz, 0.83H for ***anti*-3Ka**), 2.28-2.24 [(m, 0.17H for ***syn*-3Ka** and 0.83H for ***anti*-3Ka**)], 2.13-1.85 [(m, 0.17 × 3H for ***syn*-3Ka** and 0.83 × 3H for ***anti*-3Ka**)], 1.75-1.59 [(m, 0.17 × 3H for ***syn*-3Ka** and 0.83 × 3H for ***anti*-3Ka**)], 1.53-1.50 [(m, 0.17H for ***syn*-3Ka** and 0.83H for ***anti*-3Ka**)], 1.43-0.89 [(m, 0.17 × 18H for ***syn*-3Ka** and 0.83 × 10H for ***anti*-3Ka**)], 1.34 (s, 0.17 × 3H for ***syn*-3Ka**), 1.32 (s, 0.83 × 3H for ***anti*-3Ka**), 1.23 (s, 0.83 × 3H for ***anti*-3Ka**), 0.96 (d, *J* = 6.4 Hz, 0.83 × 3H for ***anti*-3Ka**), 0.89 (d, *J* = 6.4 Hz, 0.17 × 3H for ***syn*-3Ka**), 0.64 (d, *J* = 6.8 Hz, 0.83 × 3H for ***anti*-3Ka**), 0.58-0.53 (m, 0.83 × 2H for ***anti*-3Ka**); $^{13}C\{^1H\}$ NMR ($CDCl_3$, 100 MHz): δ 173.4, 170.6, 151.1, 150.9, 140.1, 139.7, 129.5, 128.7, 128.3, 128.2, 128.1 (2C), 127.1, 126.9, 125.8, 125.7, 125.5, 125.4, 76.7, 76.0, 65.8, 63.7, 55.0, 54.7, 51.0, 50.8, 43.8, 42.6, 40.4, 40.3, 39.2, 38.2, 36.4, 36.0, 34.8, 34.7, 32.5, 32.2, 31.7, 31.6, 30.9, 29.0, 27.8, 27.6, 27.4, 27.2, 27.0, 26.9, 26.8, 26.7, 26.2, 25.6, 24.9, 23.4, 22.1, 22.0, 12.2 (2C); HRMS (APCI) m/z ($[M+H]^+$) calcd for $C_{40}H_{54}NO_2$: 580.4149, found: 580.4151.

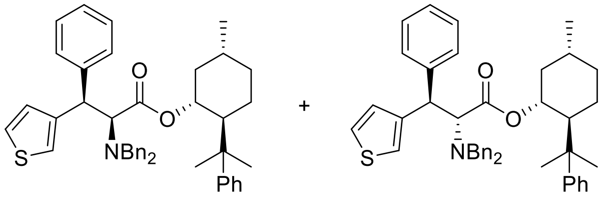

***syn*-3La** | ***anti*-3La**

A 28:72 diastereomixture of (1*R*,2*S*,5*R*)-5-methyl-2-(2-phenylpropan-2-yl)cyclohexyl (2*S*,3*R*)-2-(dibenzylamino)-3-phenyl-3-(thiophen-3-yl)propanoate (*syn*-3La) and (1*R*,2*S*,5*R*)-5-methyl-2-(2-phenylpropan-2-yl)cyclohexyl (2*R*,3*R*)-2-(dibenzylamino)-3-phenyl-3-(thiophen-3-yl)propanoate (*anti*-3La)

It was purified by silica gel column chromatography with hexane/ethyl acetate (20/1, v/v) and GPC ($CHCl_3$): 28.2 mg (22%, 0.15 mmol scale); colorless oil; ^1H NMR ($CDCl_3$, 400 MHz): δ 7.30-6.99 [(m, 0.28 × 20H for ***syn*-3La** and 0.72 × 20H for ***anti*-3La**)], 6.92-6.90 [(m, 0.28 × 2H for ***syn*-3La** and 0.72H for ***anti*-3La**)], 6.82-6.81 [(m, 0.28H for ***syn*-3La** and 0.72H for ***anti*-3La**)], 6.62 (d, *J* = 5.1 Hz, 0.72H for ***anti*-3La**), 4.77 (td, *J* = 4.0, 10.5 Hz, 0.28H for ***syn*-3La**), 4.71 (d, *J* = 11.8 Hz, 0.72H for ***anti*-3La**), 4.68 (td, *J* = 4.0, 10.7 Hz, 0.72H for ***anti*-3La**), 4.56 (d, *J* = 12.0 Hz, 0.28H for ***syn*-3La**), 4.16 (d, *J* = 11.9 Hz, 0.28H for ***syn*-3La**), 3.98 (d, *J* = 14.2 Hz, 0.28 × 2H for ***syn*-3La**), 3.95 (d, *J* = 13.4 Hz, 0.72 × 2H for ***anti*-3La**), 3.90 (d, *J* = 11.8 Hz, 0.72H for ***anti*-3La**), 3.59 (d, *J* = 14.2 Hz, 0.28 × 2H for ***syn*-3La**), 3.32 (d, *J* = 13.4 Hz, 0.72 × 2H for ***anti*-3La**), 2.25-2.22 (m, 0.72H for ***anti*-3La**), 1.92-1.81 [(m, 0.28H for ***syn*-3La** and 0.72H for ***anti*-3La**)], 1.66-1.63 (m, 0.28H for ***syn*-3La**), 1.49-1.48 [(m, 0.28H for ***syn*-3La** and 0.72 × 2H for ***anti*-3La**)], 1.32 (s, 0.28 × 3H for ***anti*-3La**), 1.26-1.22 (m, 0.72H for ***anti*-3La**), 1.22 (s, 0.28 × 3H for ***anti*-3La**), 1.16-1.04 [(m, 0.28H for ***syn*-3La** and 0.72 × 2H for ***anti*-3La**)], 0.95 (s, 0.72 × 3H for ***anti*-3La**), 0.91 (d, *J* = 6.3 Hz, 0.72 × 3H for ***anti*-3La**), 0.85-0.67 [(m, 0.28 × 3H for ***syn*-3La** and 0.72H for ***anti*-3La**)], 0.80 (d, *J* = 6.5 Hz, 0.28 × 3H for ***syn*-3La**), 0.73 (s, 0.72 × 3H for ***anti*-3La**), 0.58-0.49 (m, 0.28H for ***syn*-3La**); $^{13}C\{^1H\}$ NMR ($CDCl_3$, 100 MHz): δ 172.2, 169.4, 150.74, 150.69, 142.0, 141.63, 141.58, 141.4, 139.3, 138.9, 129.8, 129.2, 129.1, 129.0, 128.7, 128.6, 128.4, 128.3, 128.24, 128.15 (2C), 128.10, 128.06, 127.3, 127.0, 126.9, 126.7, 126.0, 125.9, 125.5, 125.4, 124.7, 122.1, 121.6, 77.0, 65.6, 65.0, 54.5, 54.4, 51.0, 50.5, 47.7, 46.5, 43.4, 41.4, 40.4, 40.2, 34.7, 34.6, 31.7, 31.4, 31.0, 30.7, 27.7, 27.6, 23.9, 22.3, 22.0, 21.9 (One sp^3 C signal overlaps with others.); HRMS (APCI) m/z ($[M+H]^+$) calcd for $C_{43}H_{48}NO_2S$: 642.3400, found: 642.3407.

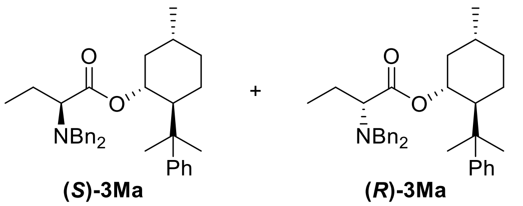

A 31:69 diastereomixture of (1*R*,2*S*,5*R*)-5-methyl-2-(2-phenylpropan-2-yl)cyclohexyl (*S*)-2-(dibenzylamino)butanoate ((*S*)-3Ma) and (1*R*,2*S*,5*R*)-5-methyl-2-(2-phenylpropan-2-yl)cyclohexyl (*R*)-2-(dibenzylamino)butanoate ((*R*)-3Ma)

It was purified by silica gel column chromatography with hexane/ethyl acetate (20/1, v/v) and GPC ($CHCl_3$): 68.7 mg (92%, 0.15 mmol scale); colorless oil; ^{1}H NMR ($CDCl_3$, 400 MHz): δ 7.38 (d, *J* = 7.0 Hz, 0.69 × 2H for **(*R*)-3Ma**), 7.33-7.29 [(m, 0.31 × 8H for **(*S*)-3Ma** and 0.69 × 6H for **(*R*)-3Ma**)], 7.25-7.21 [(m, 0.31 × 4H for **(*S*)-3Ma** and 0.69 × 3H for **(*R*)-3Ma**)], 7.13-7.08 (m, 0.31 × 2H for **(*S*)-3Ma**), 7.10 (t, *J* = 7.4 Hz, 0.69 × 2H for **(*R*)-3Ma**), 7.01-6.99 [(m, 0.31H for **(*S*)-3Ma** and 0.69H for **(*R*)-3Ma**)], 6.94 (t, *J* = 7.3 Hz, 0.69H for **(*R*)-3Ma**), 4.93 (td, *J* = 4.4, 10.7 Hz, 0.69H for **(*R*)-3Ma**), 4.81 (td, *J* = 4.2, 10.7 Hz, 0.31H for **(*S*)-3Ma**), 3.87 (d, *J* = 14.2 Hz, 0.31 × 2H for **(*S*)-3Ma**), 3.82 (d, *J* = 13.8 Hz, 0.69 × 2H for **(*R*)-3Ma**), 3.81 (d, *J* = 14.2 Hz, 0.31 × 2H for **(*S*)-3Ma**), 3.36 (d, *J* = 13.9 Hz, 0.69 × 2H for **(*R*)-3Ma**), 2.88 (dd, *J* = 5.1, 10.2 Hz, 0.31H for **(*S*)-3Ma**), 2.49 (dd, *J* = 5.8, 9.2 Hz, 0.69H for **(*R*)-3Ma**), 2.11-2.05 (m, 0.69 × 2H for **(*R*)-3Ma**), 2.01-1.95 (m, 0.31 × 2H for **(*S*)-3Ma**), 1.68-1.41 [(m, 0.31 × 5H for **(*S*)-3Ma** and 0.69 × 5H for **(*R*)-3Ma**)], 1.27 (s, 0.69 × 3H for **(*R*)-3Ma**), 1.22 (s, 0.31 × 3H for **(*S*)-3Ma**), 1.20 [(s, 0.31 × 3H for **(*S*)-3Ma** and 0.69 × 3H for **(*R*)-3Ma**)], 1.16-1.00 [(m, 0.31 × 2H for **(*S*)-3Ma** and 0.69 × 2H for **(*R*)-3Ma**)], 0.99-0.87 [(m, 0.31H for **(*S*)-3Ma** and 0.69H for **(*R*)-3Ma**)], 0.95 (d, *J* = 6.5 Hz, 0.69 × 3H for **(*R*)-3Ma**), 0.88 (d, *J* = 6.5 Hz, 0.31 × 3H for **(*S*)-3Ma**), 0.84 (t, *J* = 7.3 Hz, 0.31 × 3H for **(*S*)-3Ma**), 0.82 (t, *J* = 7.4 Hz, 0.69 × 3H for **(*R*)-3Ma**); ^{13}C{^{1}H} NMR ($CDCl_3$, 100 MHz): δ 173.1, 171.9, 151.6, 151.3, 140.4, 139.9, 129.0, 128.8, 128.3, 128.2, 128.0 (2C), 126.9 (2C), 125.4 (2C), 125.1, 125.0, 75.0, 74.7, 62.6, 61.9, 54.6, 54.3, 50.63, 50.57, 43.5, 42.1, 39.9, 39.8, 34.8, 34.7, 31.6, 31.5, 27.5, 27.04, 26.97, 26.89, 26.4, 26.1, 22.6, 22.1, 22.0 (2C), 11.6, 10.8; HRMS (APCI) m/z ($[M+H]^+$) calcd for $C_{34}H_{44}NO_2$: 498.3367, found: 498.3373.

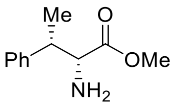

***syn*-3aa-NH$_2$**

Methyl (2*R*,3*S*)-2-amino-3-phenylbutanoate (*syn*-3aa-NH$_2$)

It was purified by filtration with celite: 12.8 mg (85%, 0.078 mmol scale); colorless oil; $[\alpha]_D^{25}$ -37.5 (*c* 0.23, $CHCl_3$, 97:3 er); ^1H NMR ($CDCl_3$, 400 MHz): δ 7.33-7.30 (m, 2H), 7.26-7.21 (m, 3H), 3.64 (d, *J* = 5.4 Hz, 1H), 3.63 (s, 3H), 3.20 (dq, *J* = 5.5, 7.1 Hz, 1H), 1.40 (brs, 2H), 1.31 (d, *J* = 7.1 Hz, 3H); $^{13}C\{^1H\}$ NMR ($CDCl_3$, 100 MHz): δ 175.2, 143.1, 128.6, 127.8, 126.9, 60.6, 52.0, 43.5, 14.9; HRMS (APCI) m/z ($[M+H]^+$) calcd for $C_{11}H_{16}NO_2$: 194.1176, found: 194.1173.

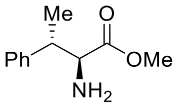

***anti*-3aa-NH$_2$**

Methyl (2*S*,3*S*)-2-amino-3-phenylbutanoate (*anti*-3aa-NH$_2$)

It was purified by filtration with celite: 16.6 mg (87%, 0.099 mmol scale); colorless oil; $[\alpha]_D^{25}$ -1.60 (*c* 0.83, $CHCl_3$, 97:3 er); ^1H NMR ($CDCl_3$, 400 MHz): δ 7.34-7.30 (m, 2H), 7.25-7.22 (m, 1H), 7.20-7.18 (m, 2H), 3.73 (s, 3H), 3.57 (d, *J* = 7.1 Hz, 1H), 3.10 (quin, *J* = 7.1 Hz, 1H), 1.34 (brs, 2H), 1.33 (d, *J* = 7.1 Hz, 3H); $^{13}C\{^1H\}$ NMR ($CDCl_3$, 100 MHz): δ 175.4, 142.2, 128.7, 128.0, 127.1, 60.7, 52.0, 44.5, 18.6; HRMS (APCI) m/z ($[M+H]^+$) calcd for $C_{11}H_{16}NO_2$: 194.1176, found: 194.1172.

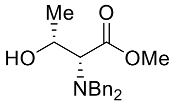

***syn*-3wa-OH**

Methyl (2*R,3*R**)-2-(dibenzylamino)-3-hydroxybutanoate (*syn*-3wa-OH)**

It was purified by silica gel column chromatography with hexane/ethyl acetate (5/1, v/v): 14.7 mg (85% , 0.13 mmol scale from **3wa**; 57%, 0.08 mmol scale from **3xa**); colorless oil; ^1H NMR ($CDCl_3$, 400 MHz): δ 7.36-7.30 (m, 8H), 7.27-7.23 (m, 2H), 4.22-4.13 (m, 1H), 3.88 (d, *J* = 13.5 Hz, 2H), 3.86 (s, 3H), 3.44 (d, *J* = 13.5 Hz, 2H), 3.13 (d, *J* = 9.0 Hz, 1H), 2.34 (d, *J* = 4.5 Hz, 1H), 1.20 (d, *J* = 6.3 Hz, 3H); $^{13}C\{^1H\}$ NMR ($CDCl_3$, 100 MHz): δ 173.2, 138.9, 129.2, 128.45, 127.39, 67.1, 66.3, 55.7, 51.4, 20.3; HRMS (APCI) m/z ($[M+H]^+$) calcd for $C_{19}H_{24}NO_3$: 314.1751, found: 314.1753.

NMR Spectra for Products

[¹H and ¹³C{¹H} NMR Spectra of **3aa**]

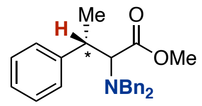

3aa

syn/anti = 43:57

¹H NMR
(400 MHz, $CDCl_3$)

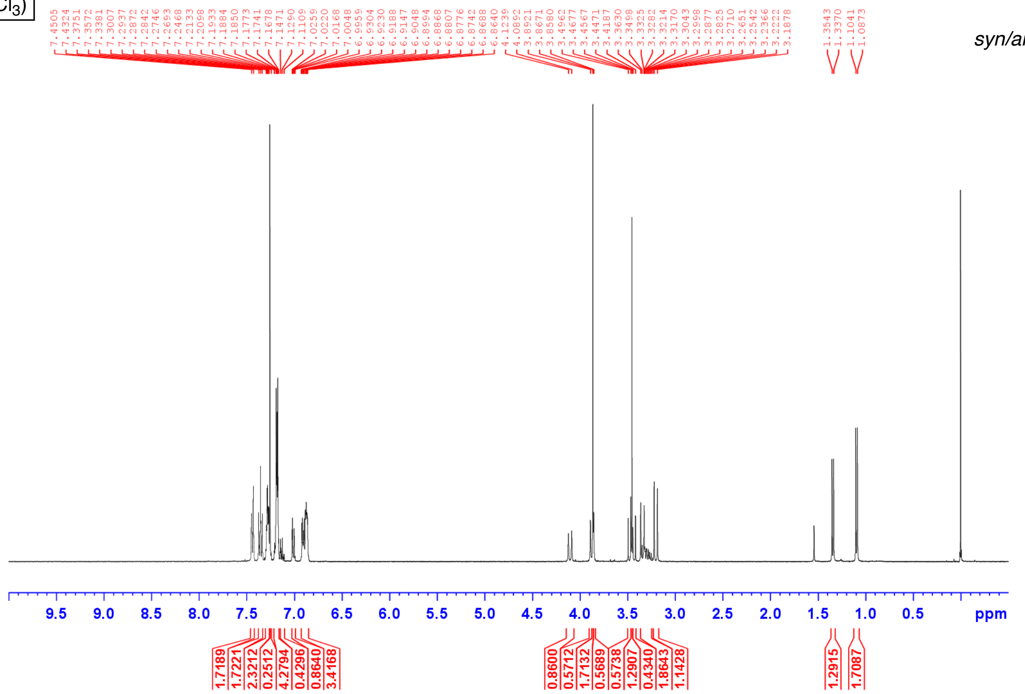

¹³C{¹H} NMR
(100 MHz, $CDCl_3$)

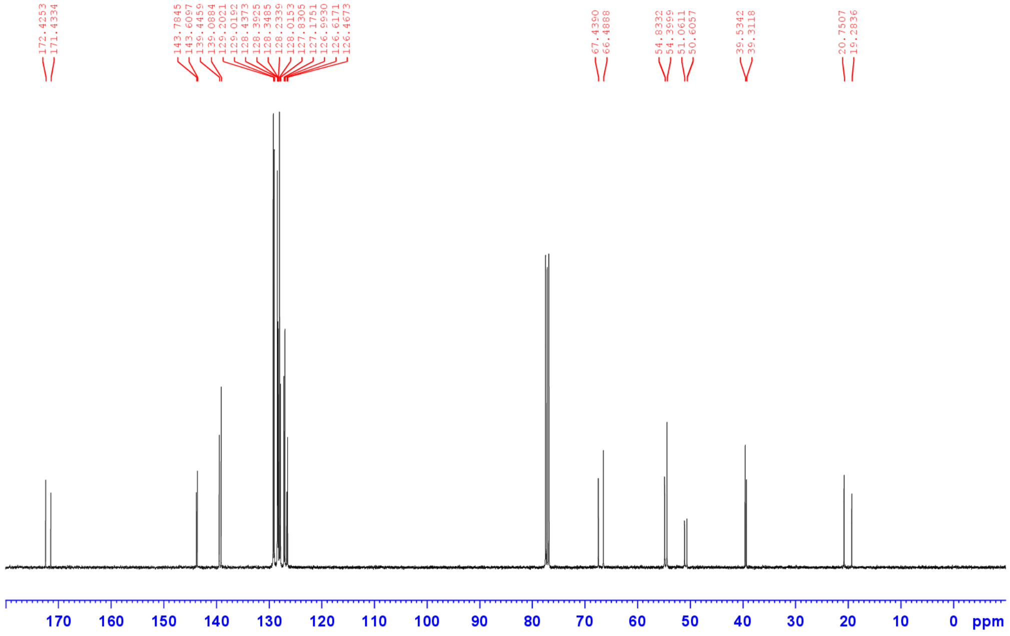

[^{1}H and ^{13}C{^{1}H} NMR Spectra of *syn*-**3aa**]

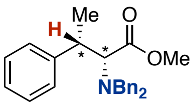

syn-**3aa**

^{1}H NMR
(400 MHz, $CDCl_3$)

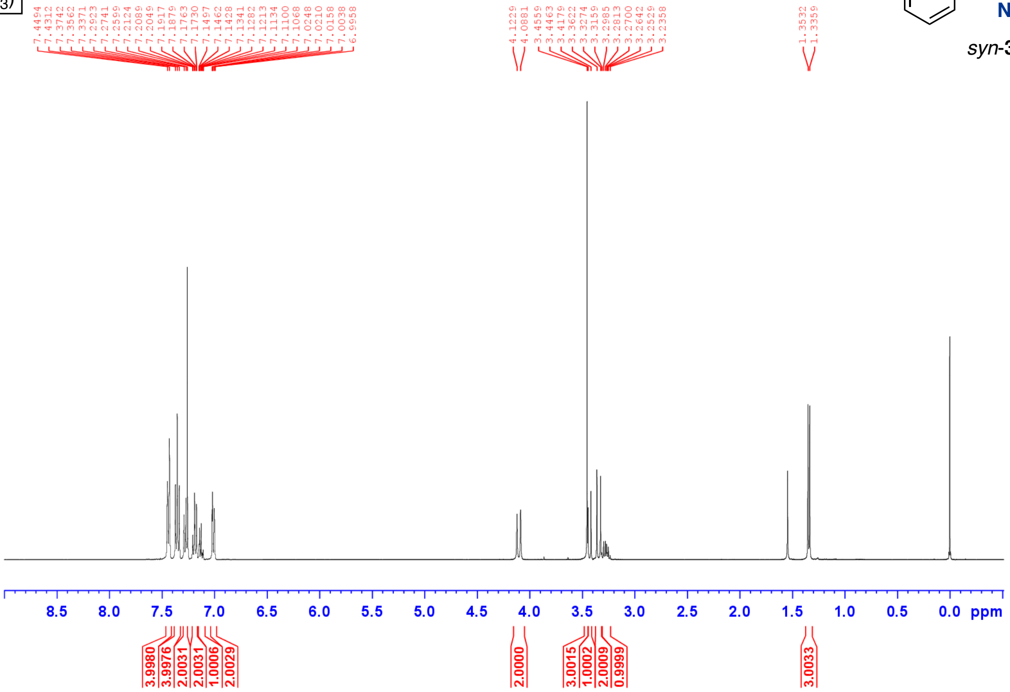

^{13}C{^{1}H} NMR
(100 MHz, $CDCl_3$)

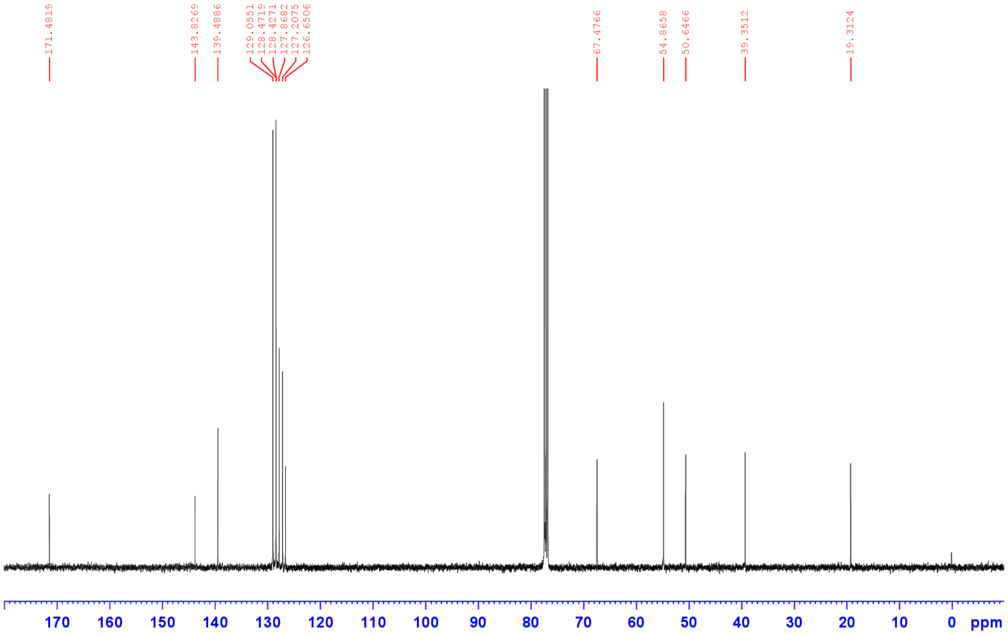

[^{1}H and ^{13}C{^{1}H} NMR Spectra of *anti*-**3aa**]

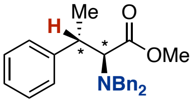

anti-**3aa**

^{1}H NMR
(400 MHz, $CDCl_3$)

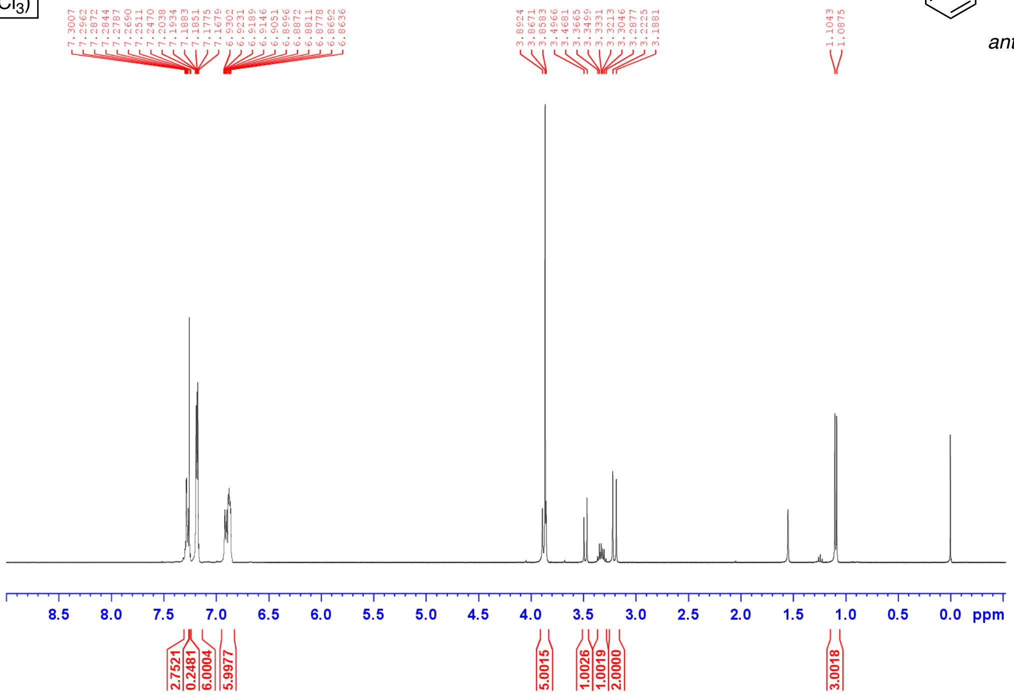

^{13}C{^{1}H} NMR
(100 MHz, $CDCl_3$)

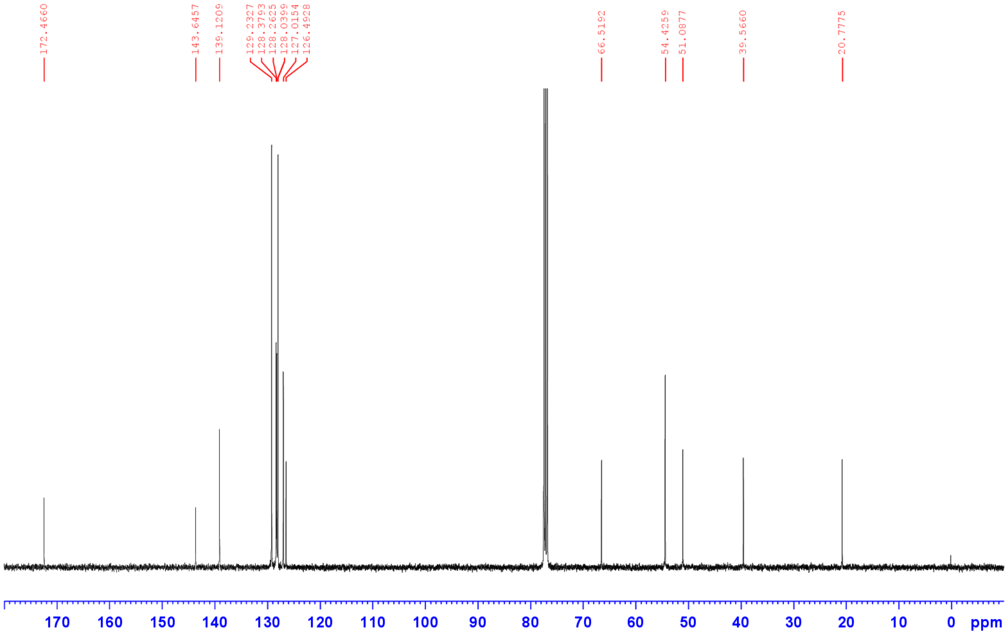

[^{1}H and ^{13}C{^{1}H} NMR Spectra of **3ba**]

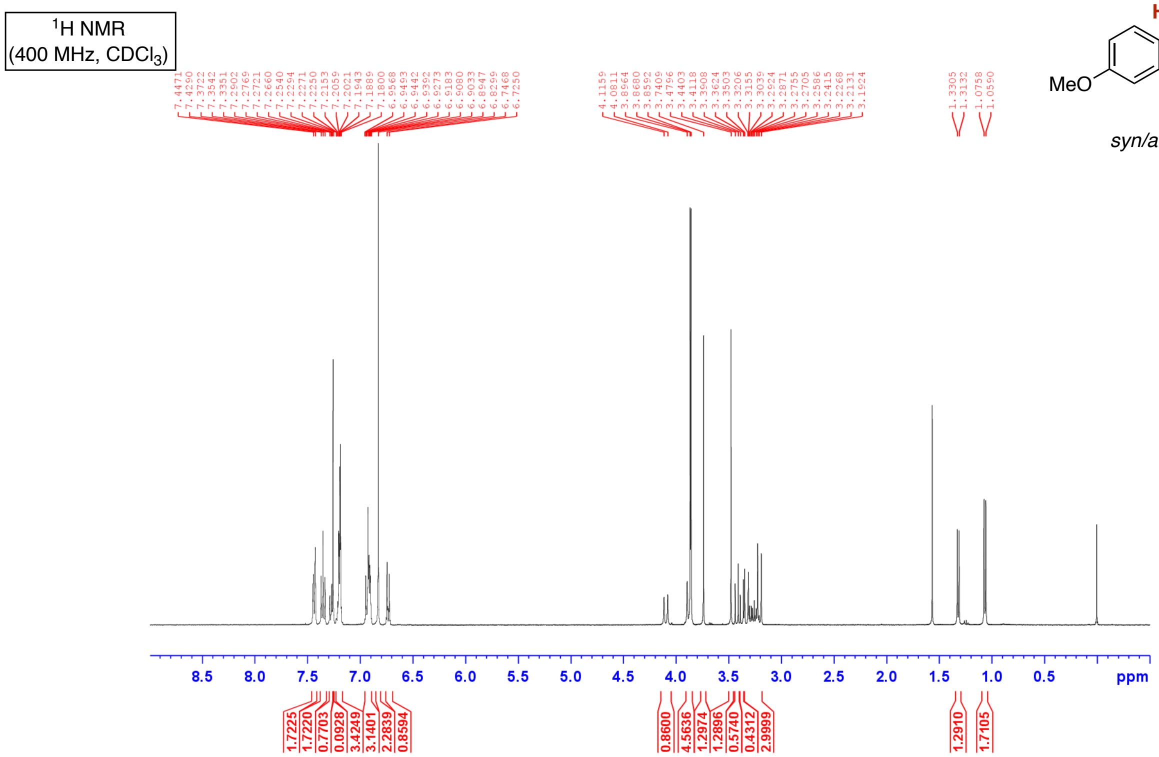

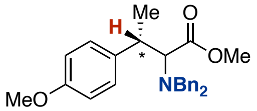

3ba

syn/anti = 43:57

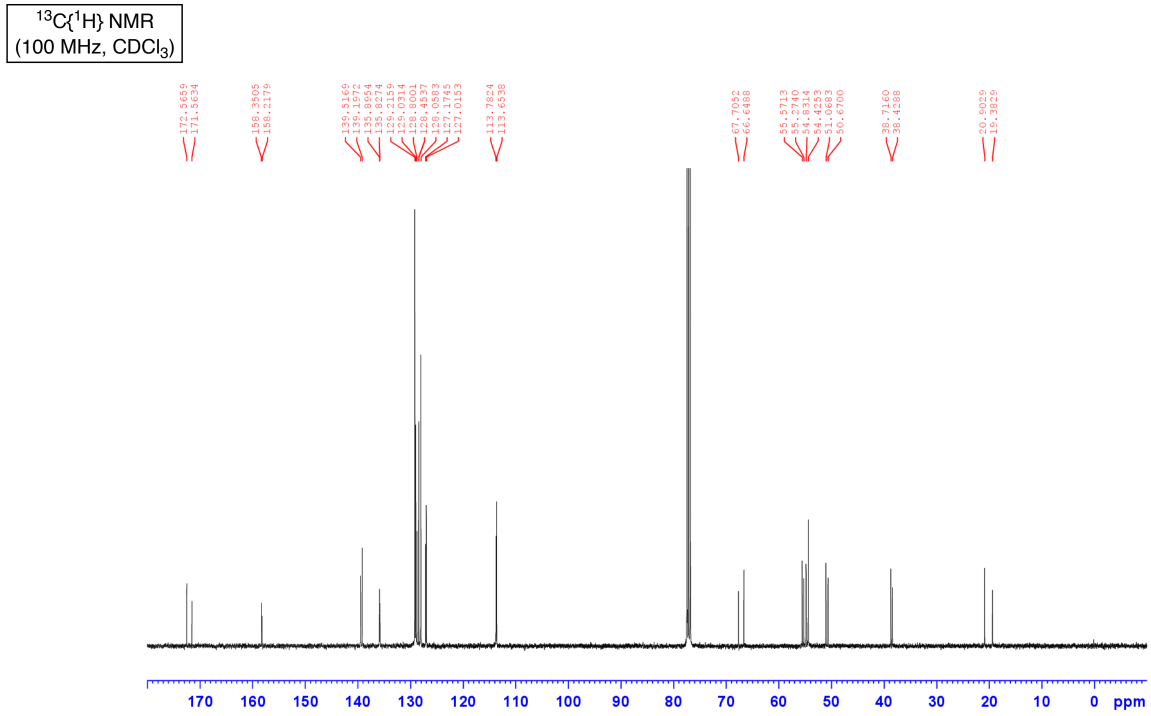

[^{1}H, $^{13}C\{^{1}H\}$, and $^{19}F\{^{1}H\}$ NMR Spectra of **3ca**]

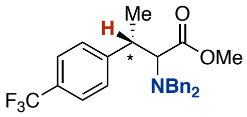

3ca

syn/anti = 45:55

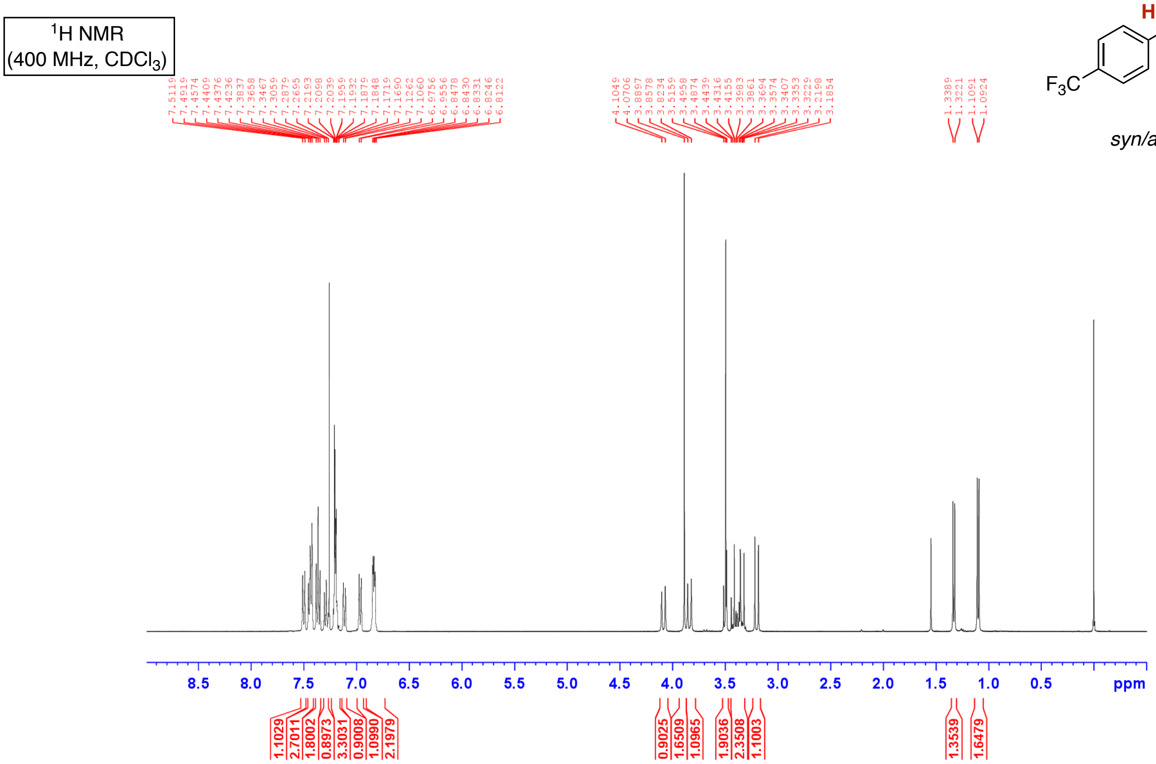

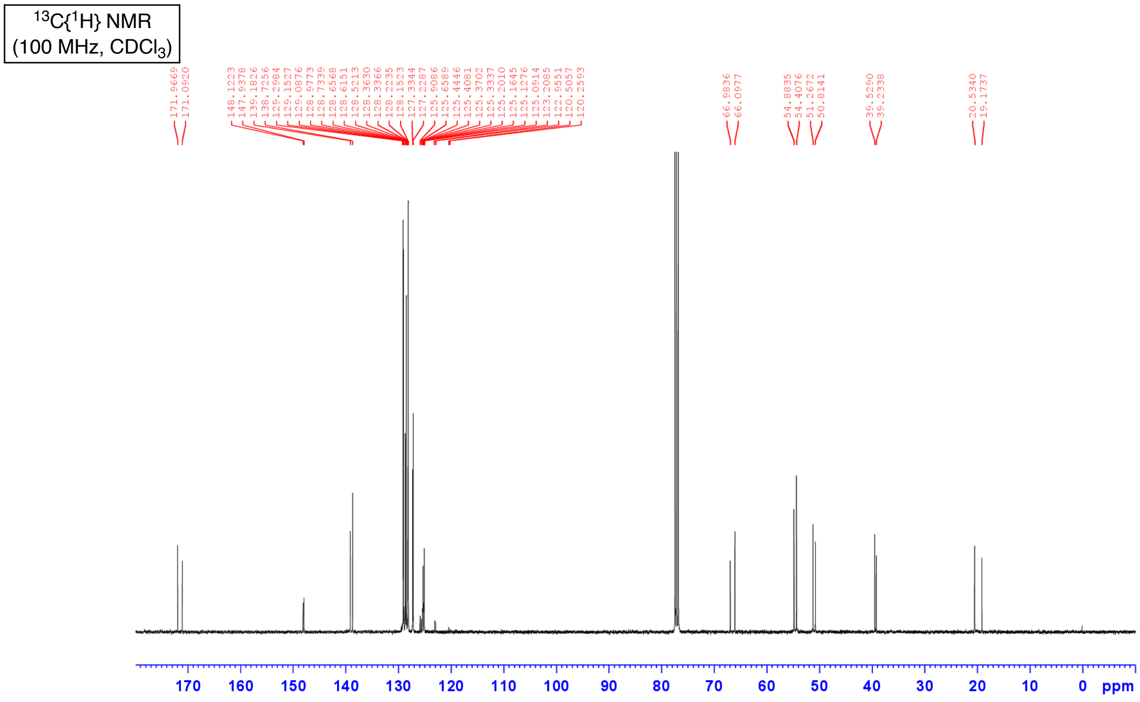

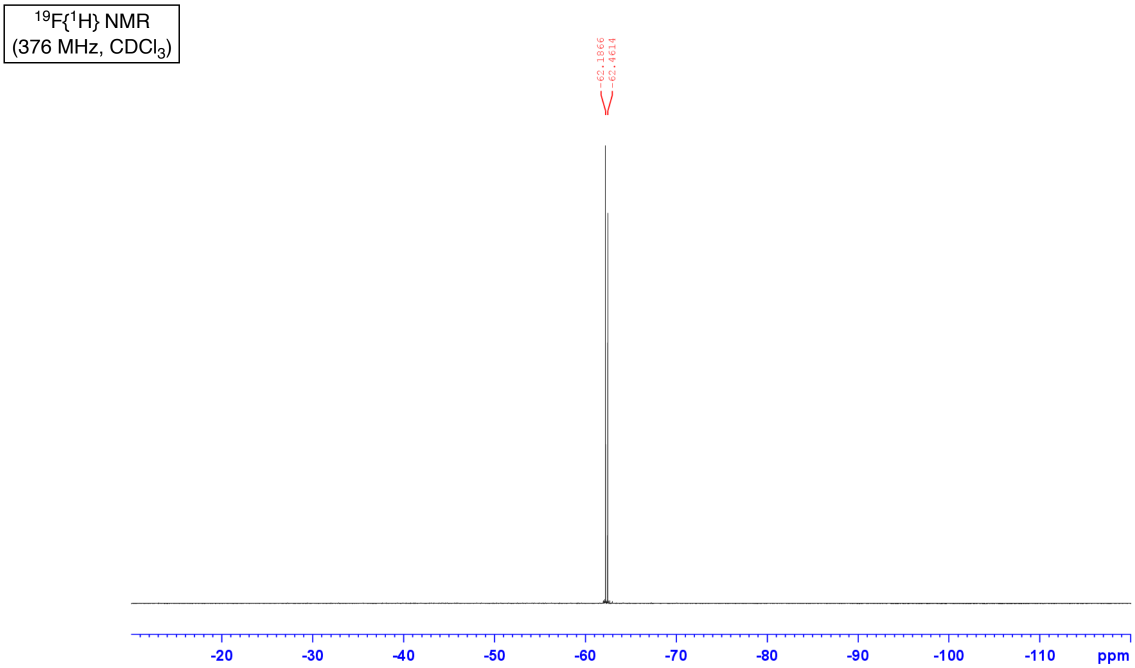

$^{19}F\{^1H\}$ NMR
(376 MHz, $CDCl_3$)
-62.1866
-62.4614
-20
-30
-40
-50
-60
-70
-80
-90
-100
-110
ppm

[^{1}H and ^{13}C{^{1}H} NMR Spectra of **3da**]

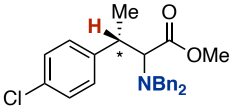

3da

syn/anti = 47:53

^{1}H NMR
(400 MHz, $CDCl_3$)

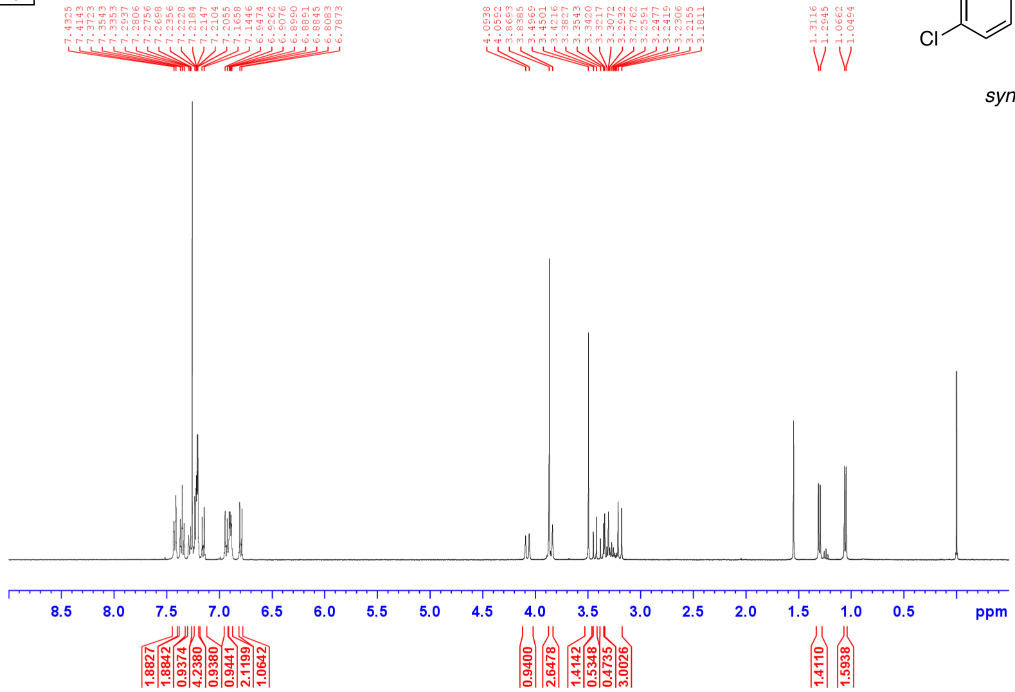

^{13}C{^{1}H} NMR
(100 MHz, $CDCl_3$)

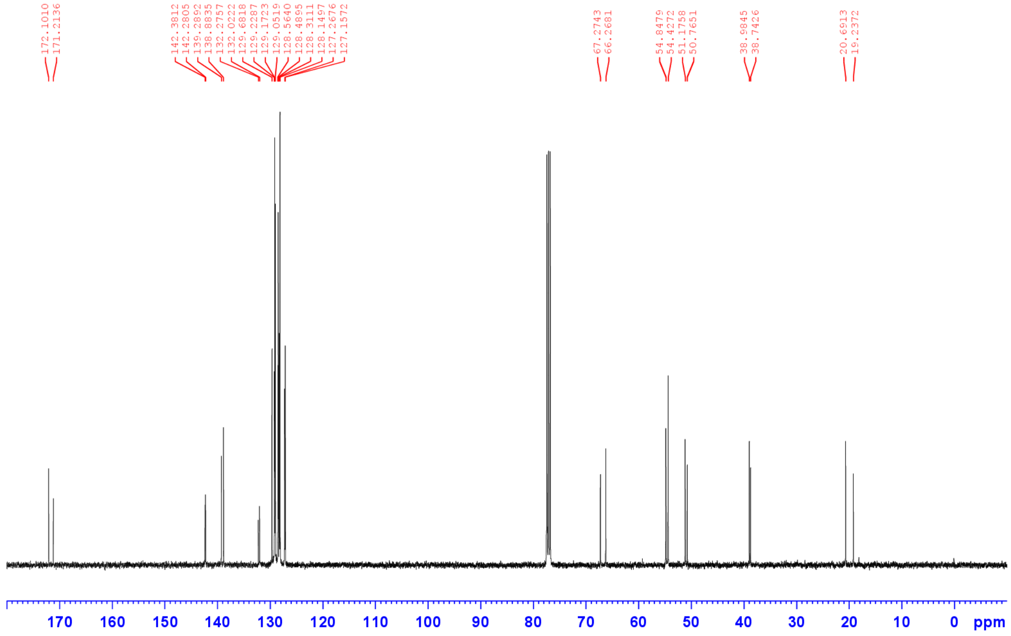

[^{1}H and ^{13}C{^{1}H} NMR Spectra of **3ea**]

^{1}H NMR (400 MHz, $CDCl_3$)

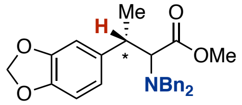

3ea

syn/anti = 45:55

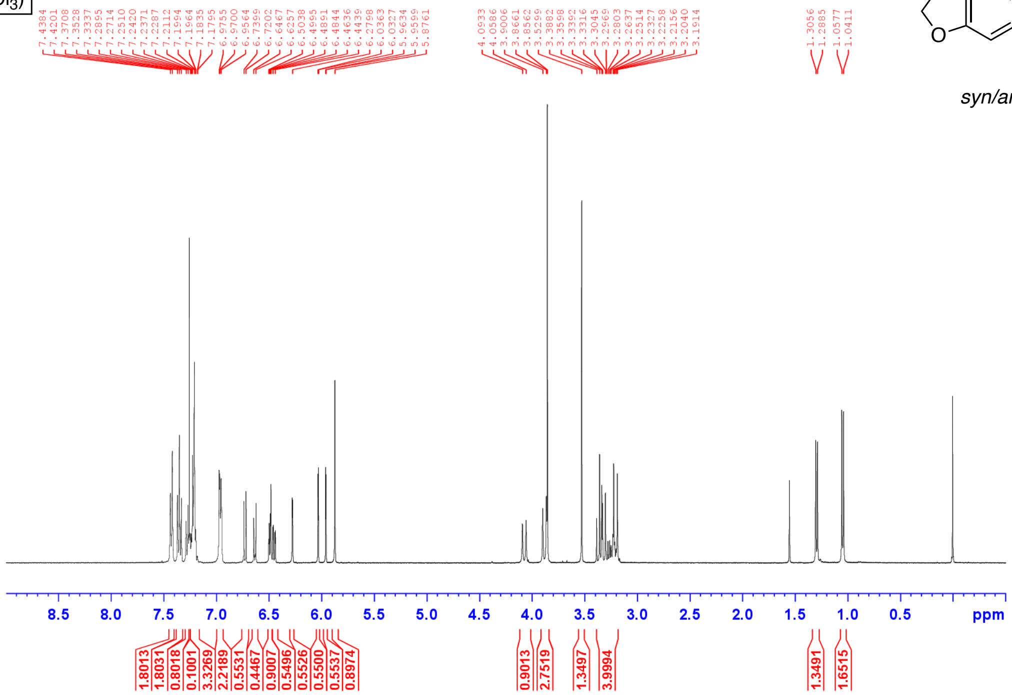

^{13}C{^{1}H} NMR (100 MHz, $CDCl_3$)

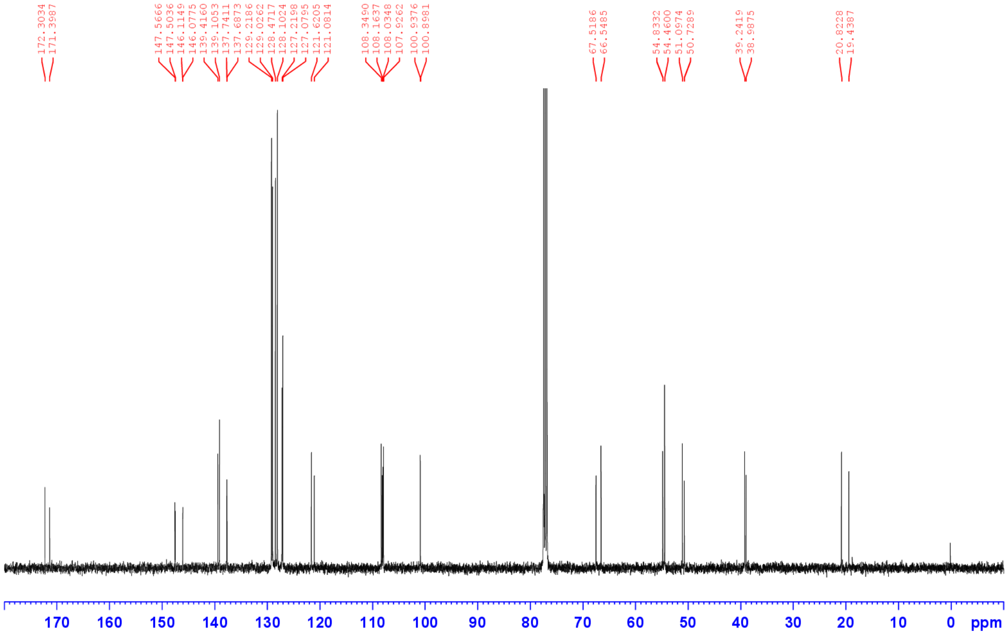

[^{1}H and ^{13}C{^{1}H} NMR Spectra of **3fa**]

^{1}H NMR
(400 MHz, $CDCl_3$)

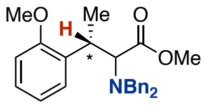

3fa
syn/anti = 37:63

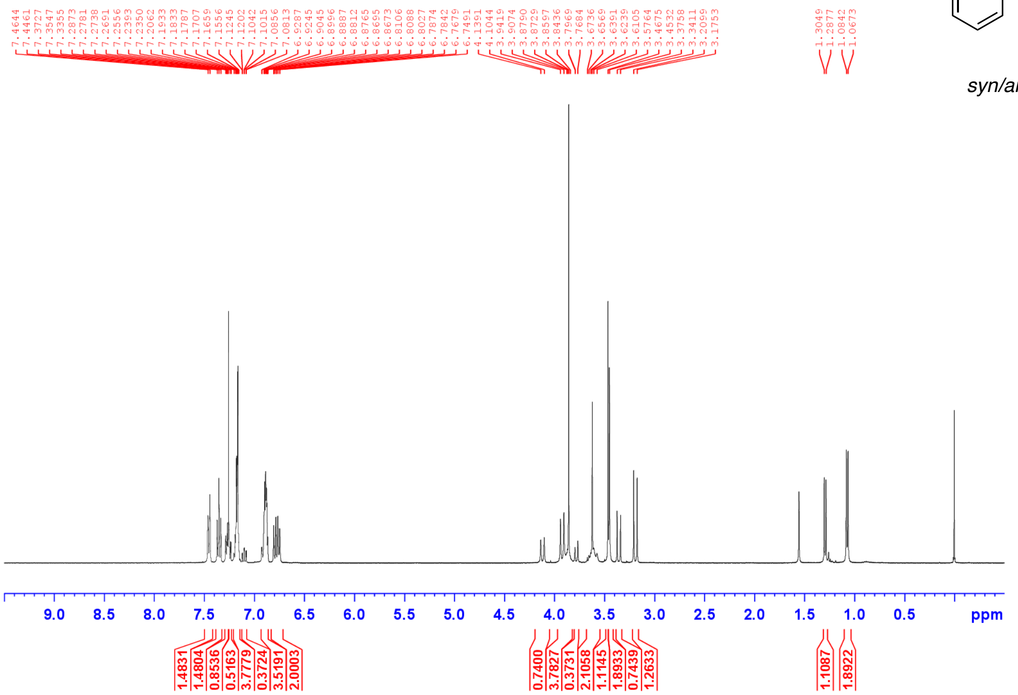

^{13}C{^{1}H} NMR
(100 MHz, $CDCl_3$)

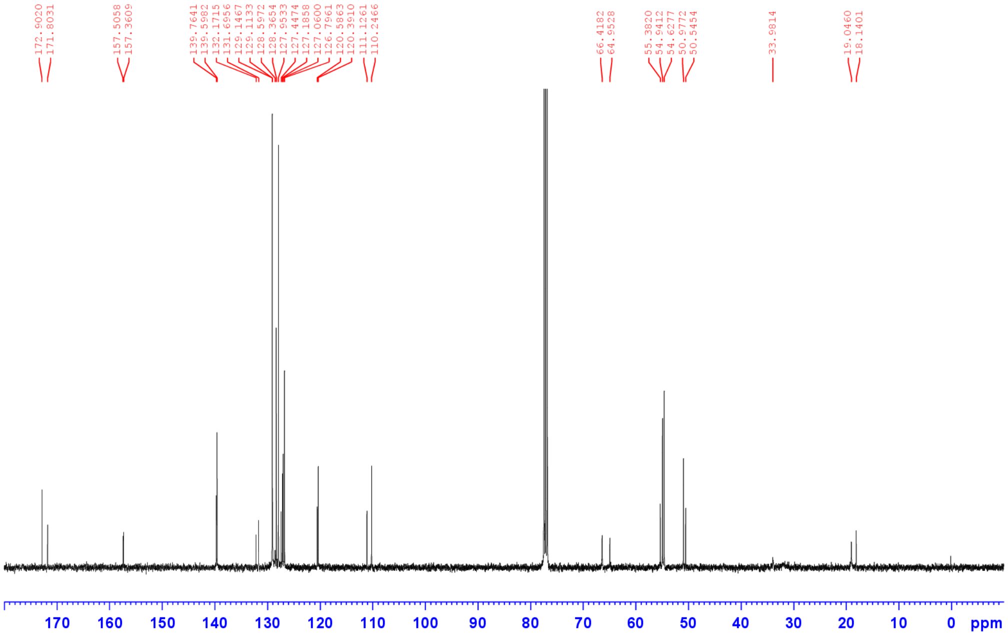

[^{1}H and ^{13}C{^{1}H} NMR Spectra of **3ga**]

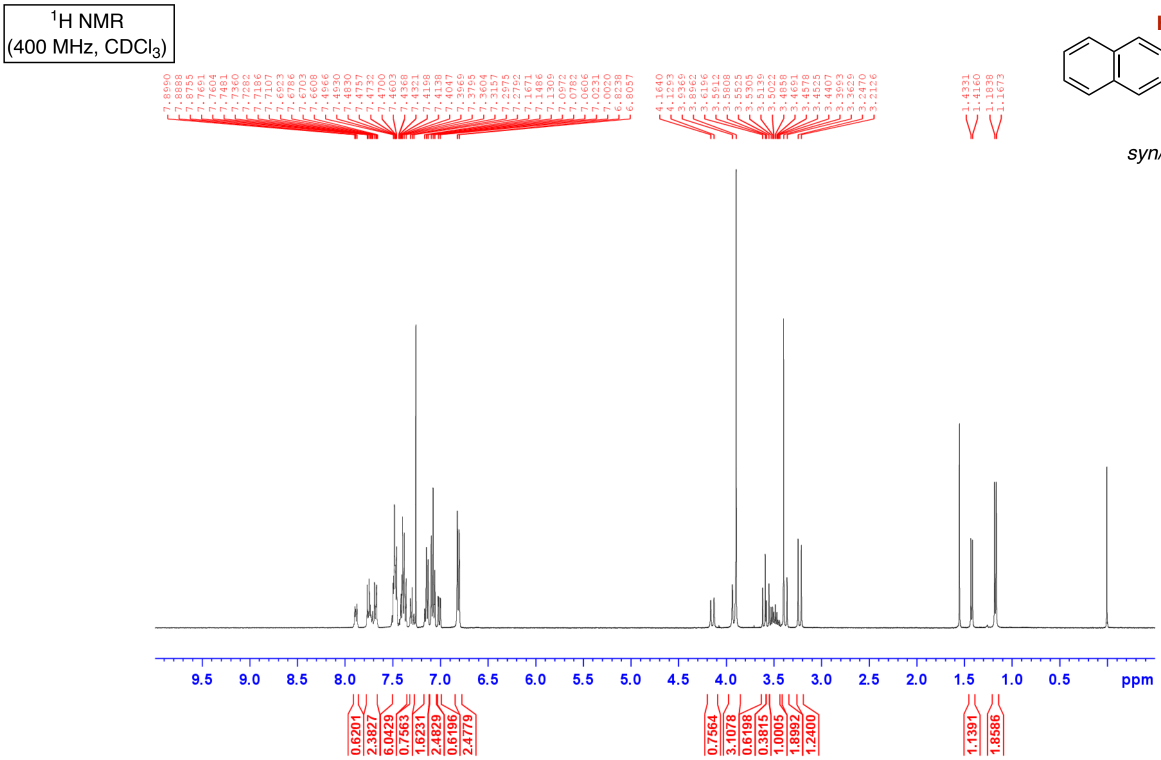

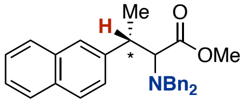

3ga

syn/anti = 38:62

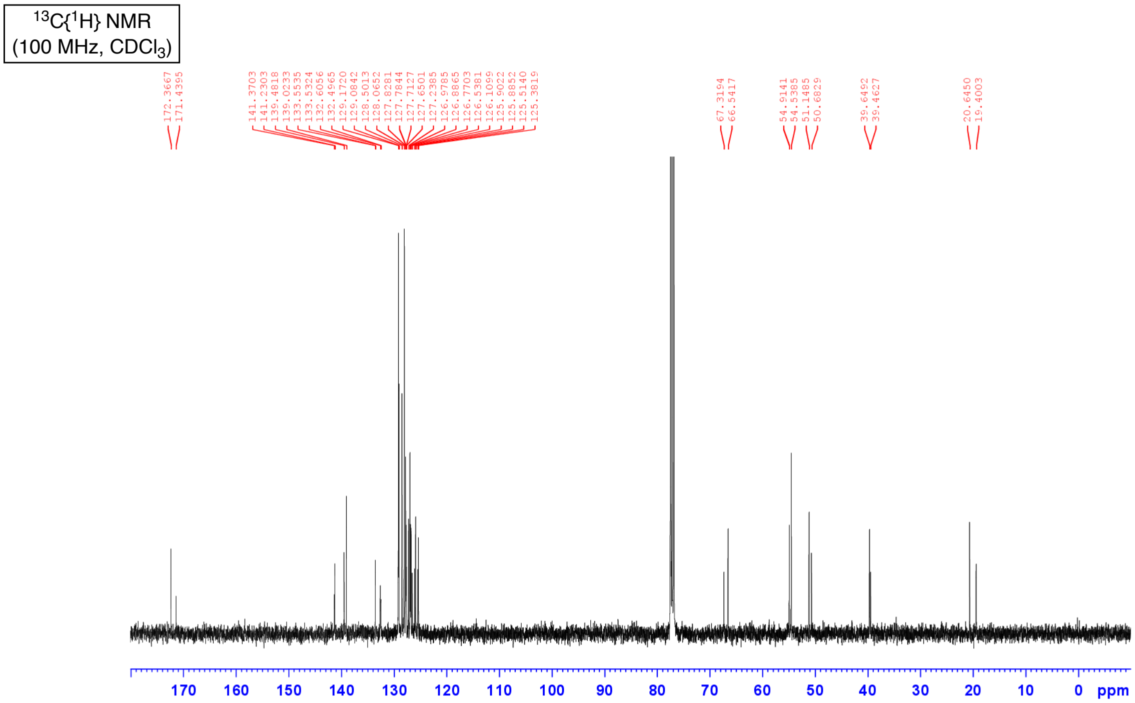

[^{1}H and ^{13}C{^{1}H} NMR Spectra of **3ha**]

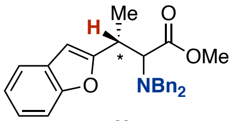

3ha

syn/anti = 37:63

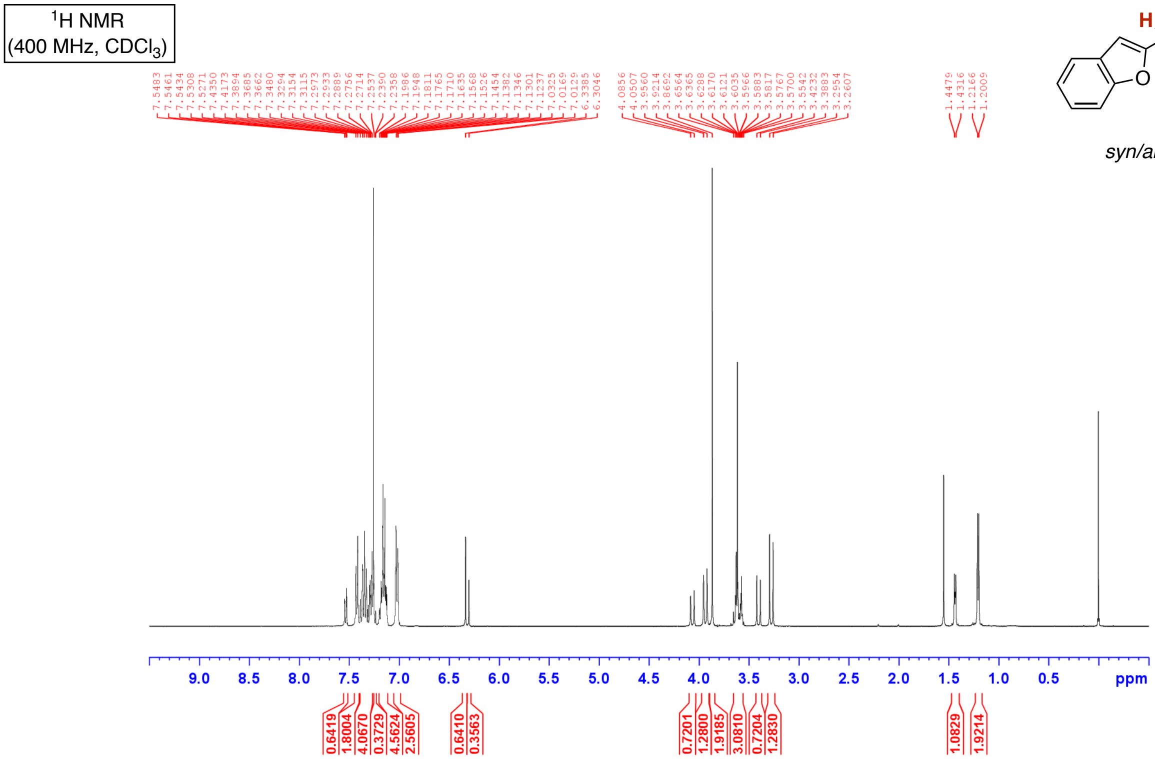

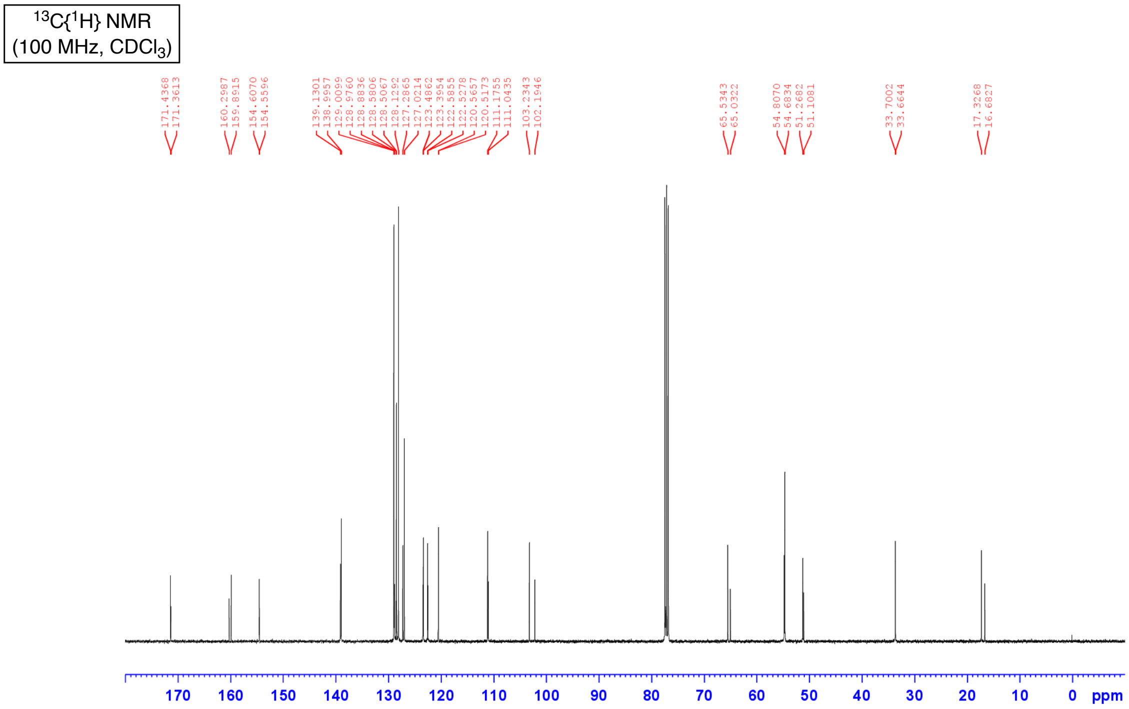

[^{1}H and ^{13}C{^{1}H} NMR Spectra of **3ia**]

^{1}H NMR
(400 MHz, $CDCl_3$)

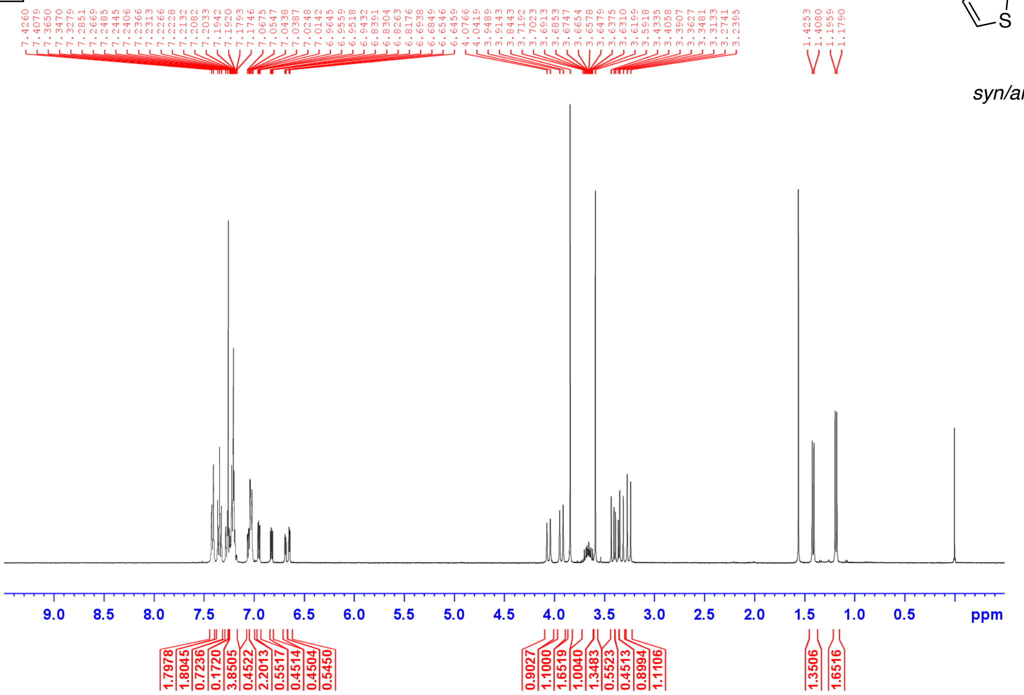

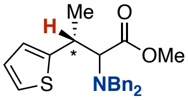

3ia
syn/anti = 45:55

^{13}C{^{1}H} NMR
(100 MHz, $CDCl_3$)

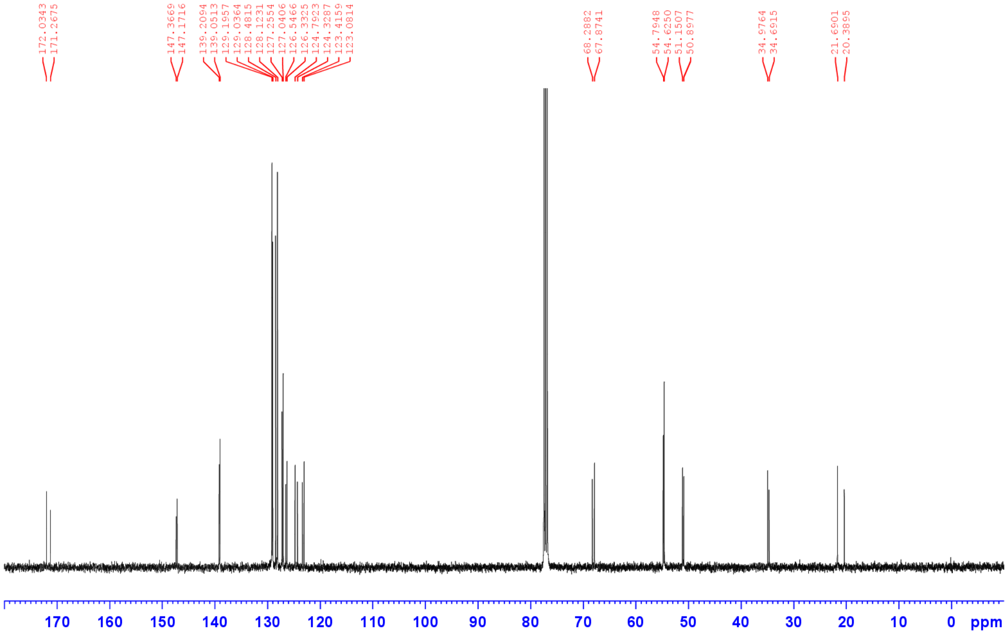

[^{1}H and ^{13}C{^{1}H} NMR Spectra of **3ja**]

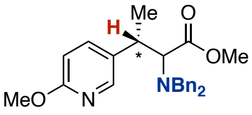

3ja

syn/anti = 47:53

^{1}H NMR
(400 MHz, $CDCl_3$)

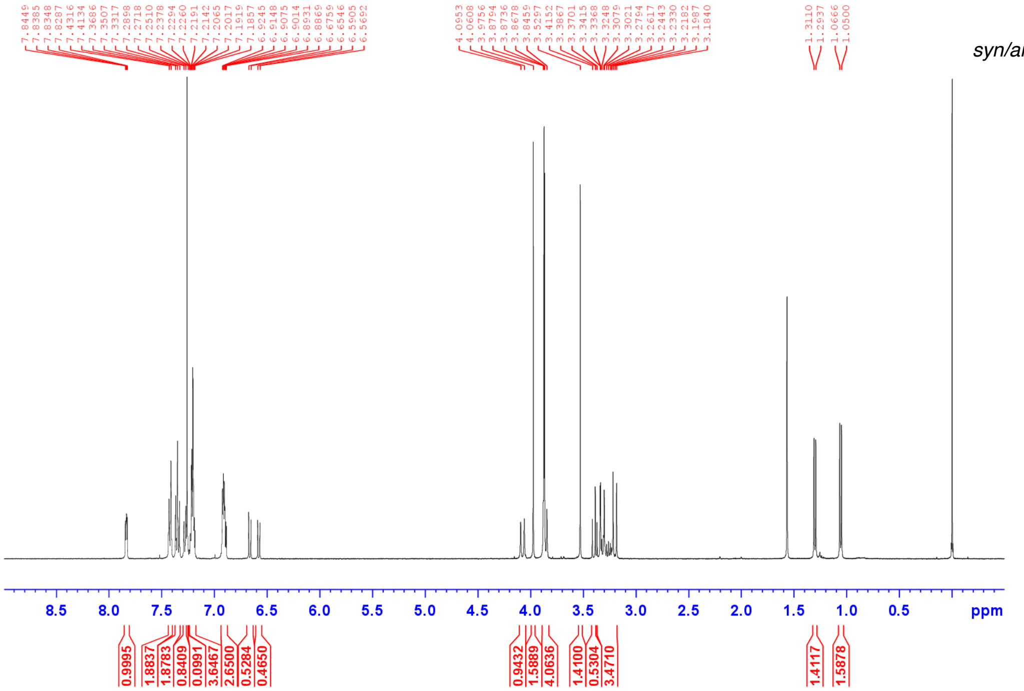

^{13}C{^{1}H} NMR
(100 MHz, $CDCl_3$)

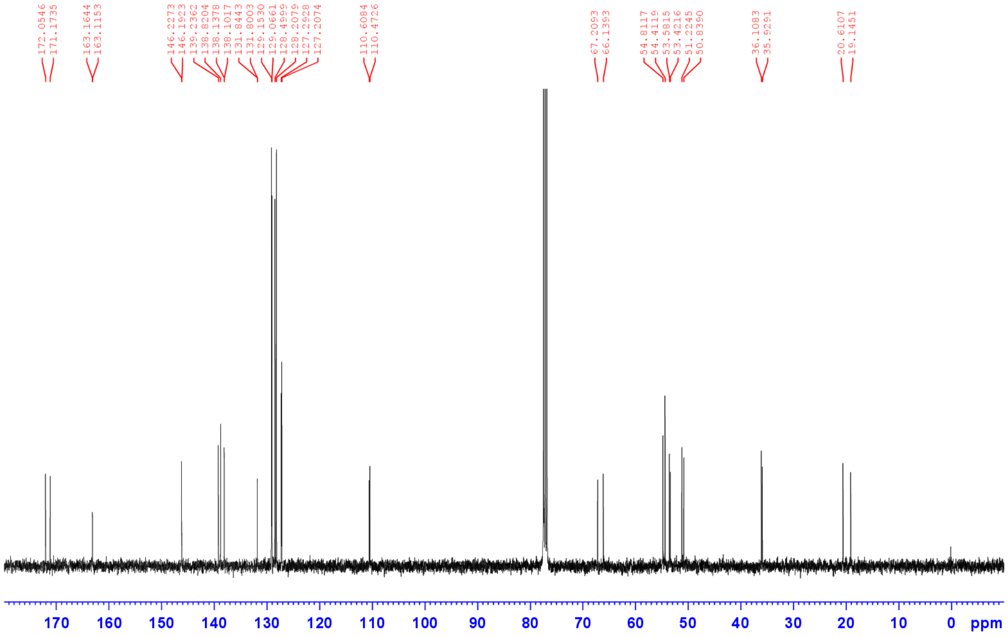

[^{1}H and ^{13}C{^{1}H} NMR Spectra of **3ka**]

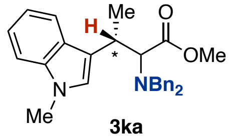

3ka

syn/anti = 36:64

^{1}H NMR
(400 MHz, $CDCl_3$)

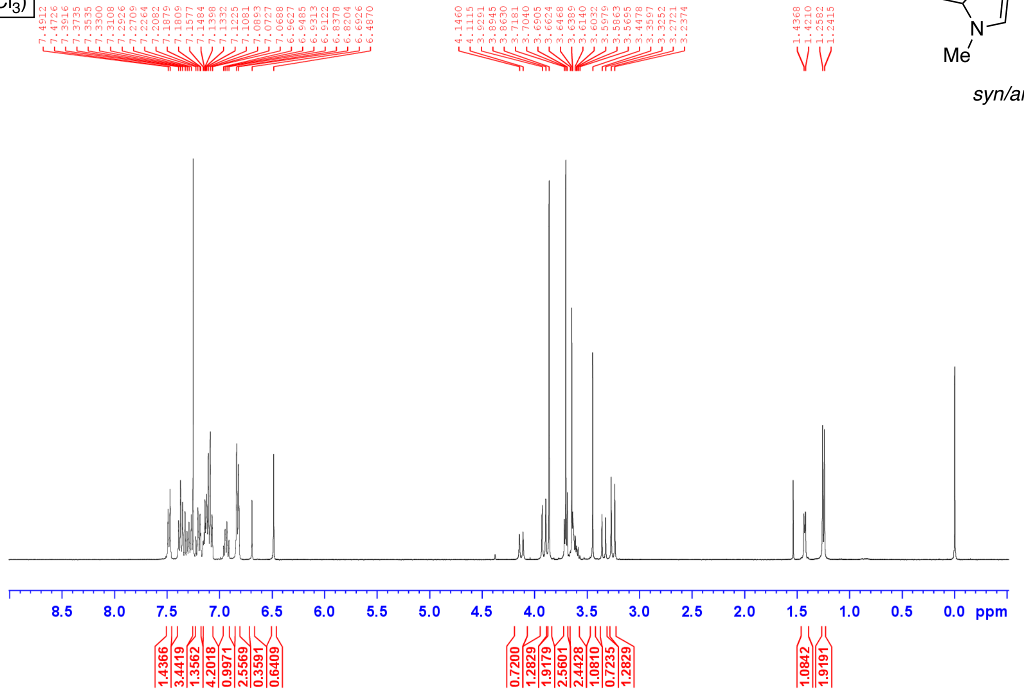

^{13}C{^{1}H} NMR
(100 MHz, $CDCl_3$)

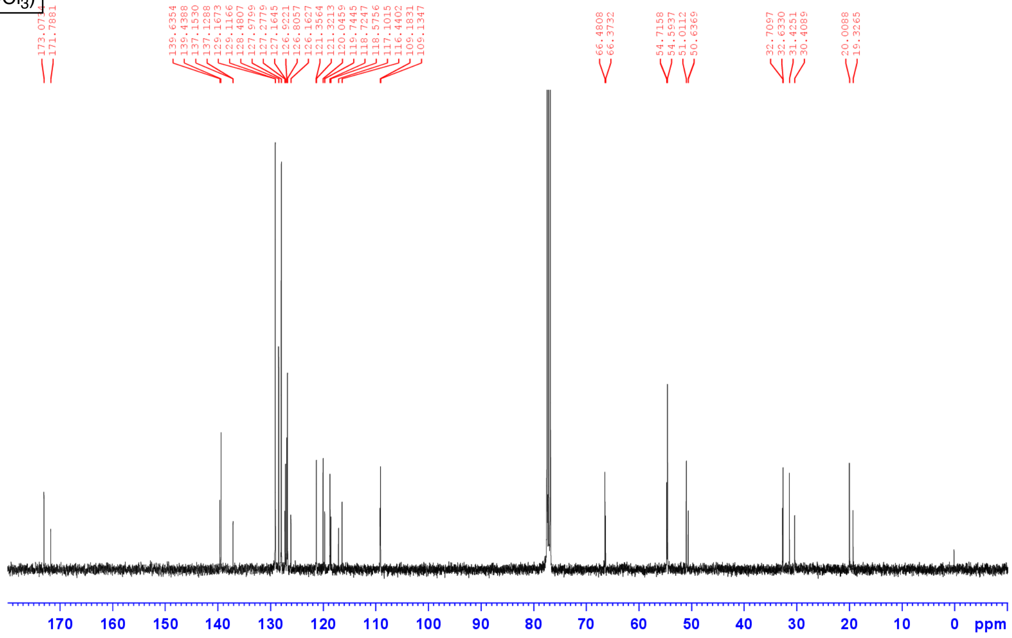

[^{1}H and ^{13}C{^{1}H} NMR Spectra of **3la**]

^{1}H NMR
(400 MHz, $CDCl_3$)

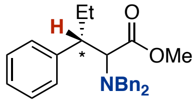

3la
syn/anti = 41:59

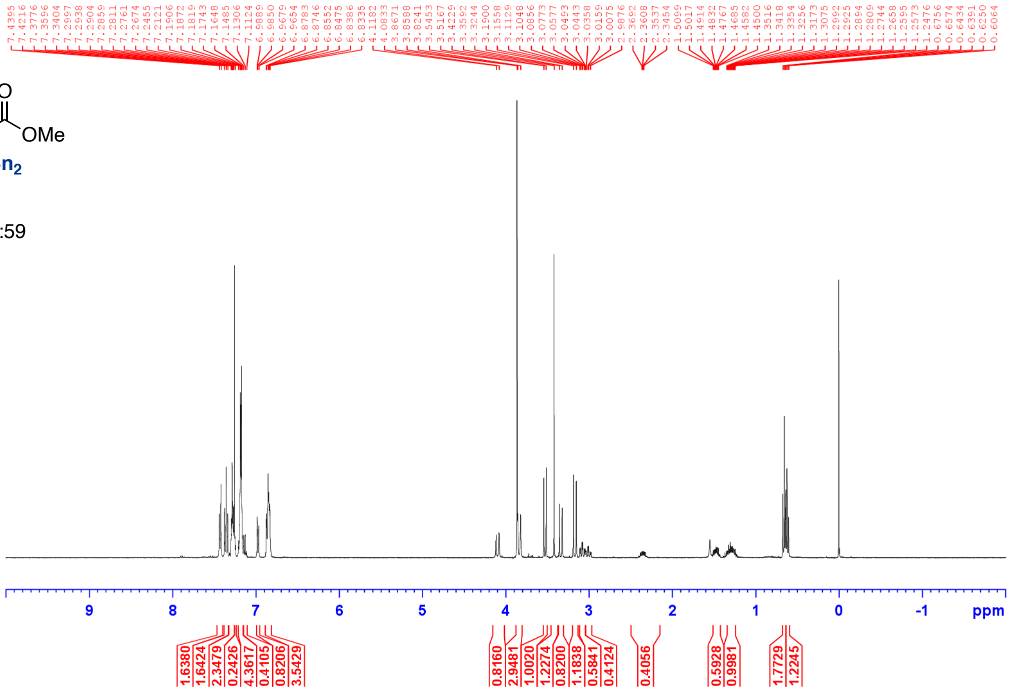

^{13}C{^{1}H} NMR
(100 MHz, $CDCl_3$)

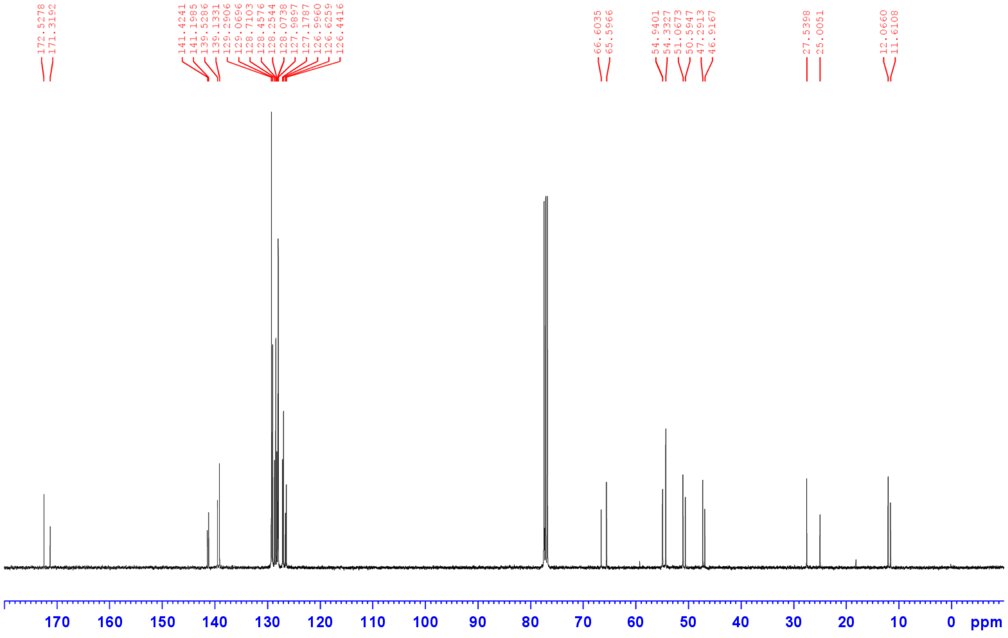

[^{1}H and ^{13}C{^{1}H} NMR Spectra of **3ma**]

^{1}H NMR
(400 MHz, $CDCl_3$)

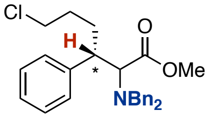

3ma

syn/anti = 44:56

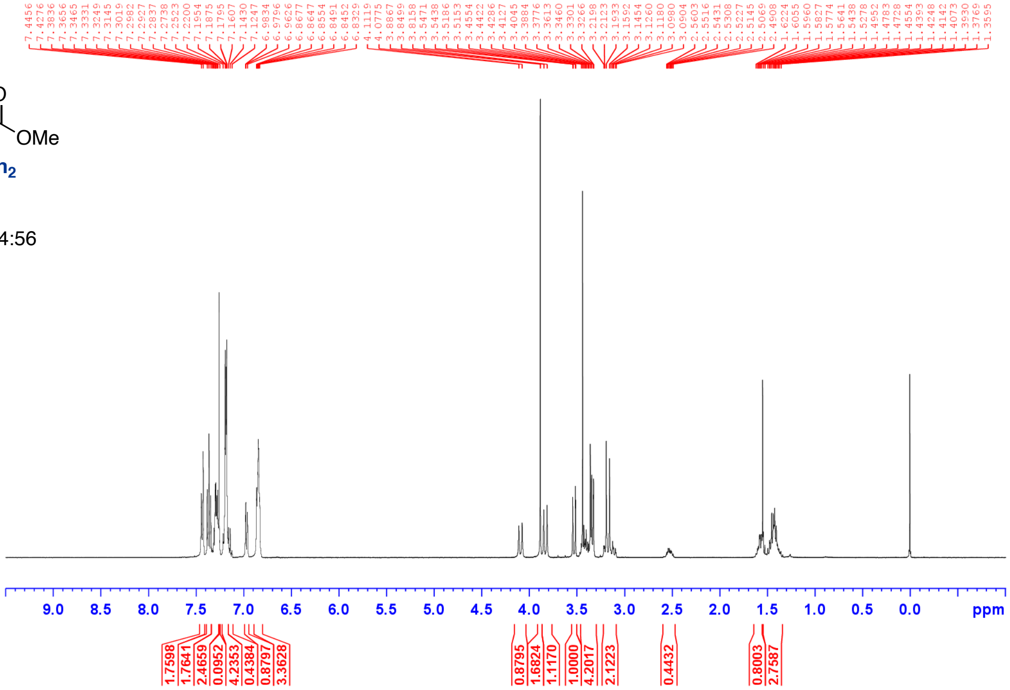

^{13}C{^{1}H} NMR
(100 MHz, $CDCl_3$)

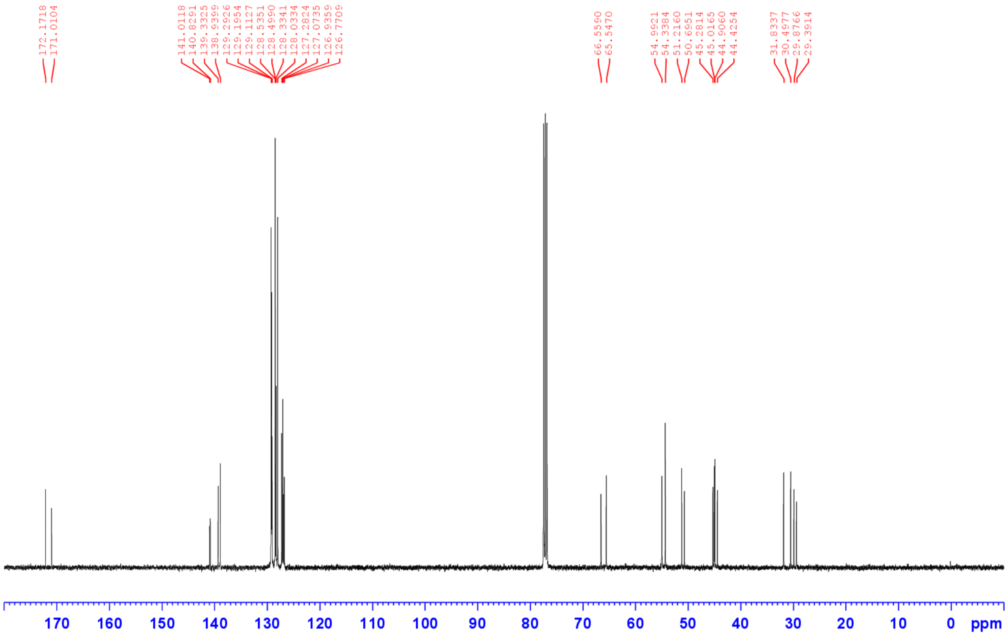

[^{1}H and ^{13}C{^{1}H} NMR Spectra of **3na**]

^{1}H NMR (400 MHz, $CDCl_3$)

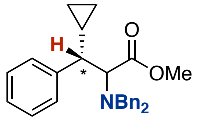

3na

syn/anti = 45:55

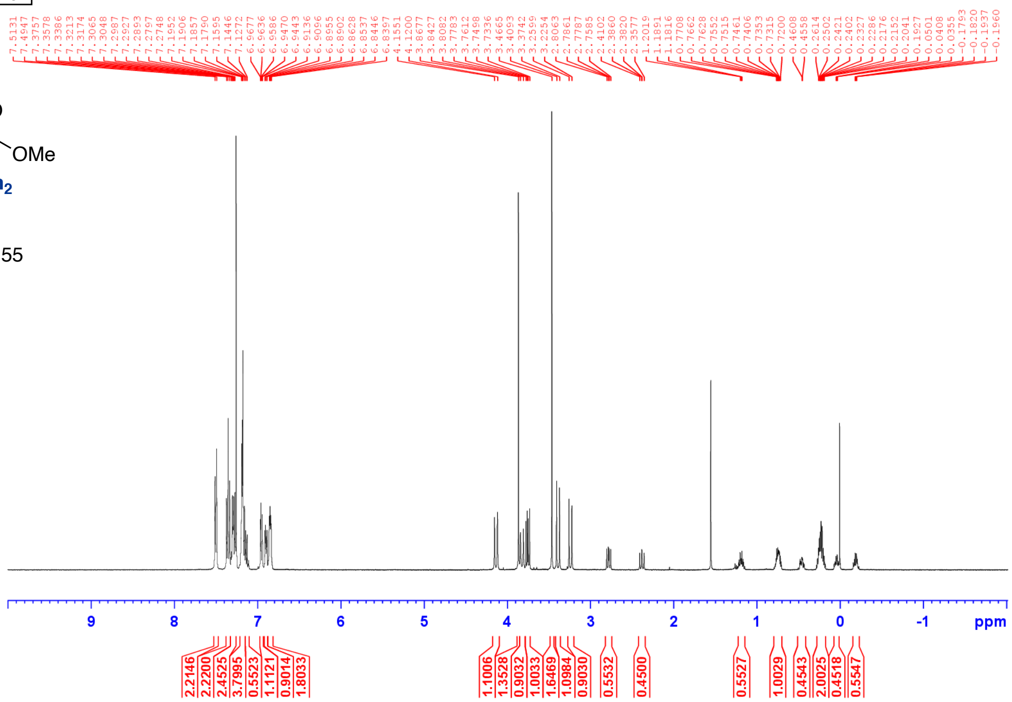

^{13}C{^{1}H} NMR (100 MHz, $CDCl_3$)

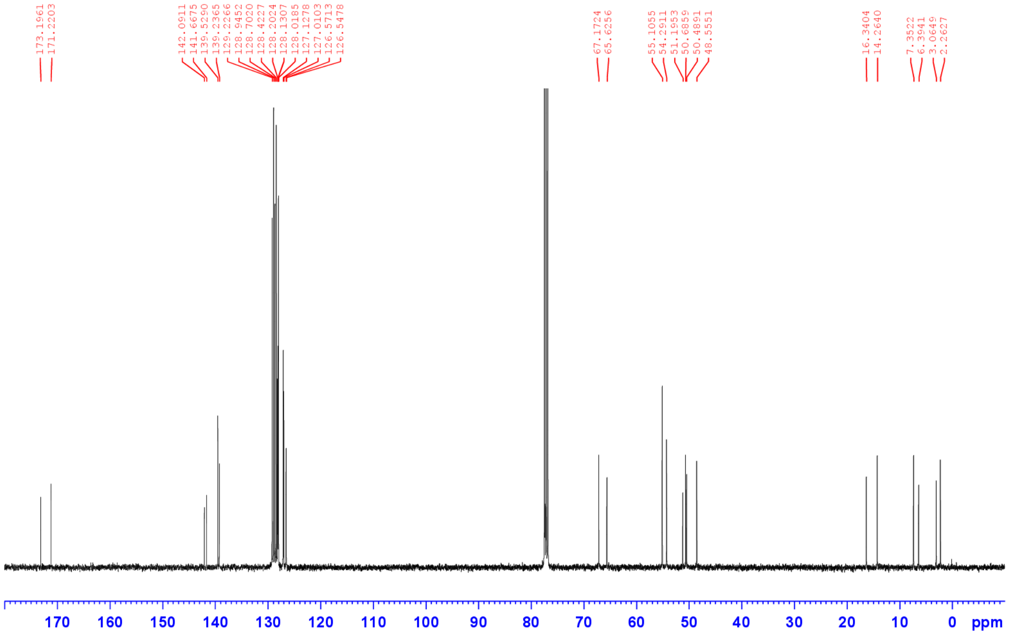

[^{1}H and ^{13}C{^{1}H} NMR Spectra of **3oa**]

^{1}H NMR
(400 MHz, $CDCl_3$)

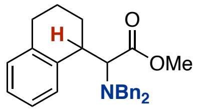

3oa

syn/anti = 37:63

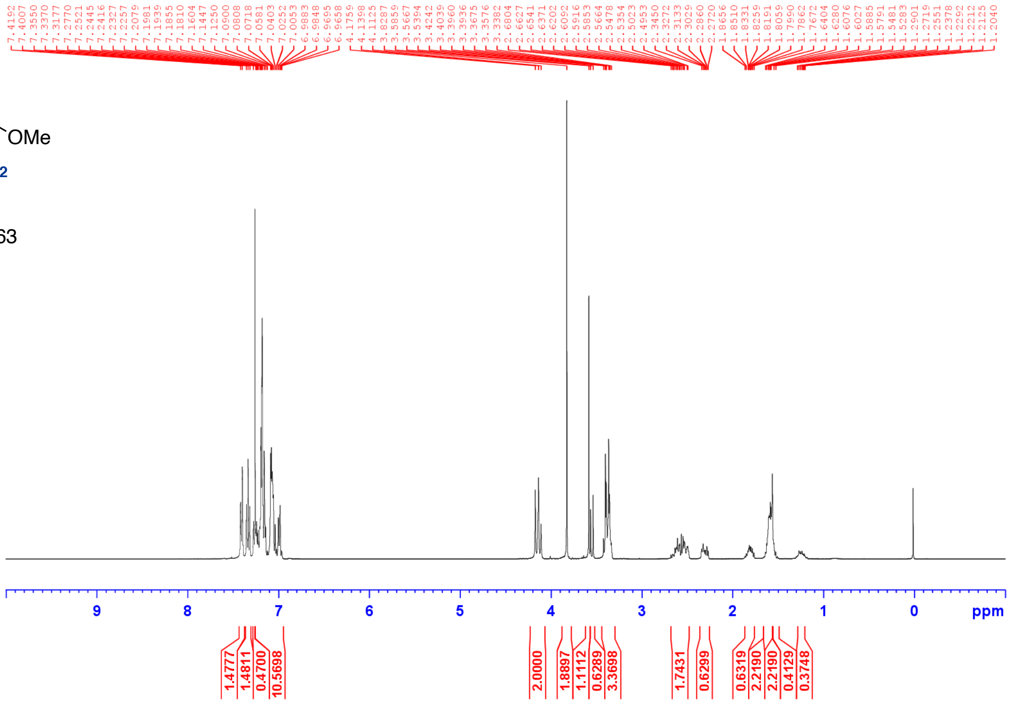

^{13}C{^{1}H} NMR
(100 MHz, $CDCl_3$)

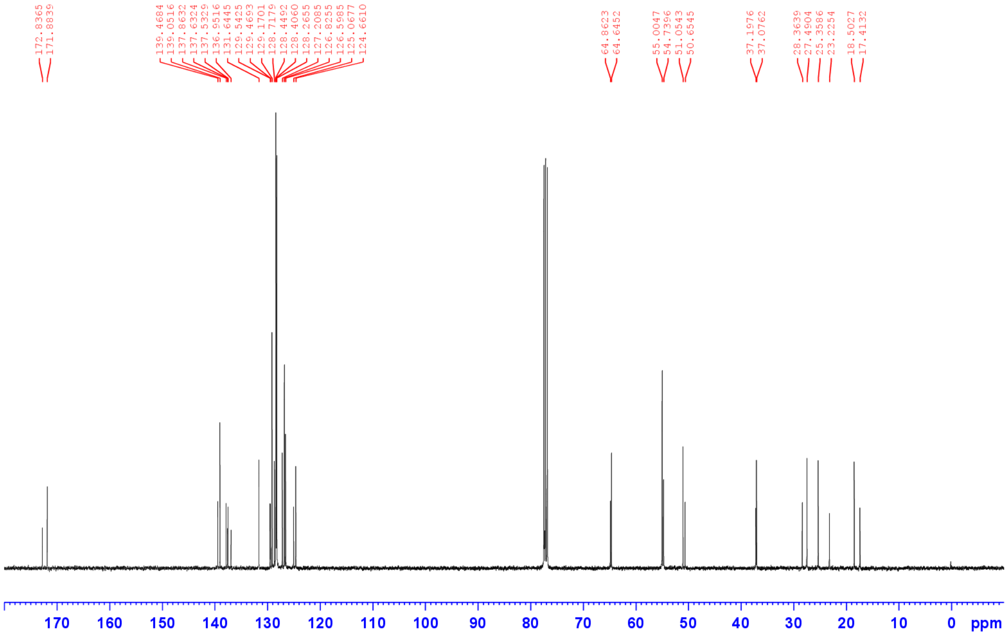

[^{1}H and ^{13}C{^{1}H} NMR Spectra of **3pa**]

^{1}H NMR
(400 MHz, $CDCl_3$)

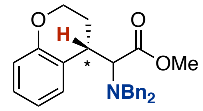

3pa
syn/anti = 42:58

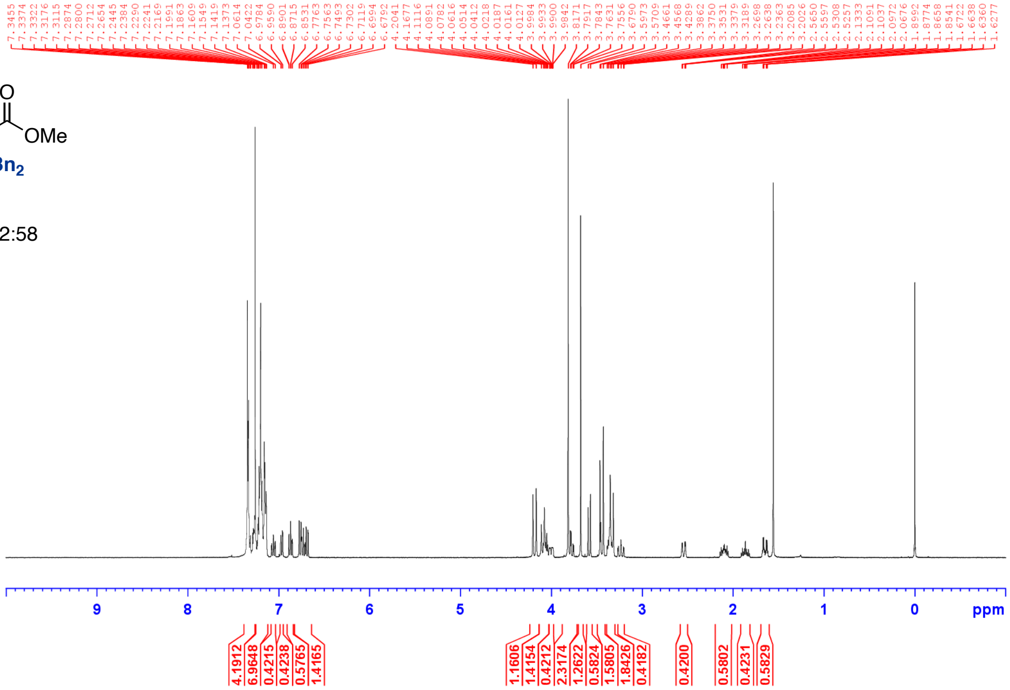

^{13}C{^{1}H} NMR
(100 MHz, $CDCl_3$)

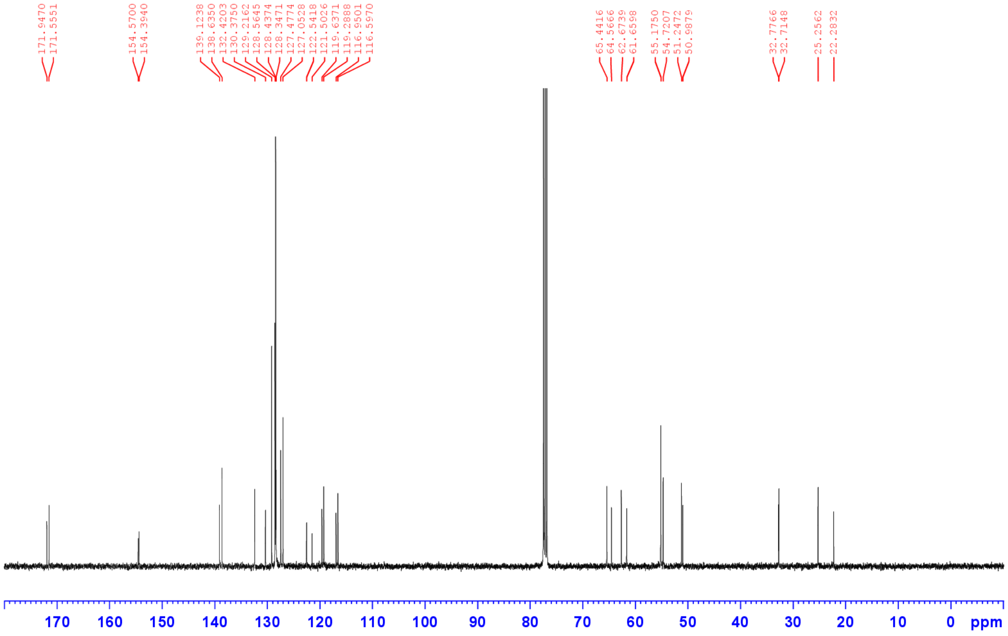

[^{1}H and ^{13}C{^{1}H} NMR Spectra of **3qa**]

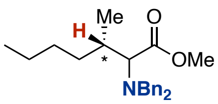

3qa

syn/anti = 49:51

^{1}H NMR
(400 MHz, $CDCl_3$)

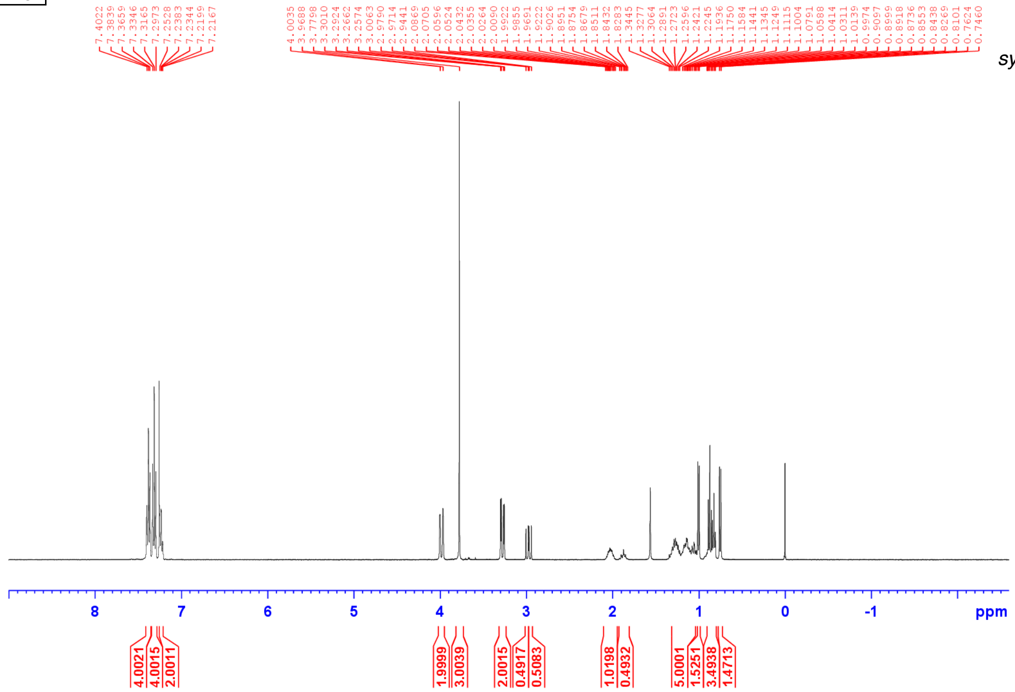

^{13}C{^{1}H} NMR
(100 MHz, $CDCl_3$)

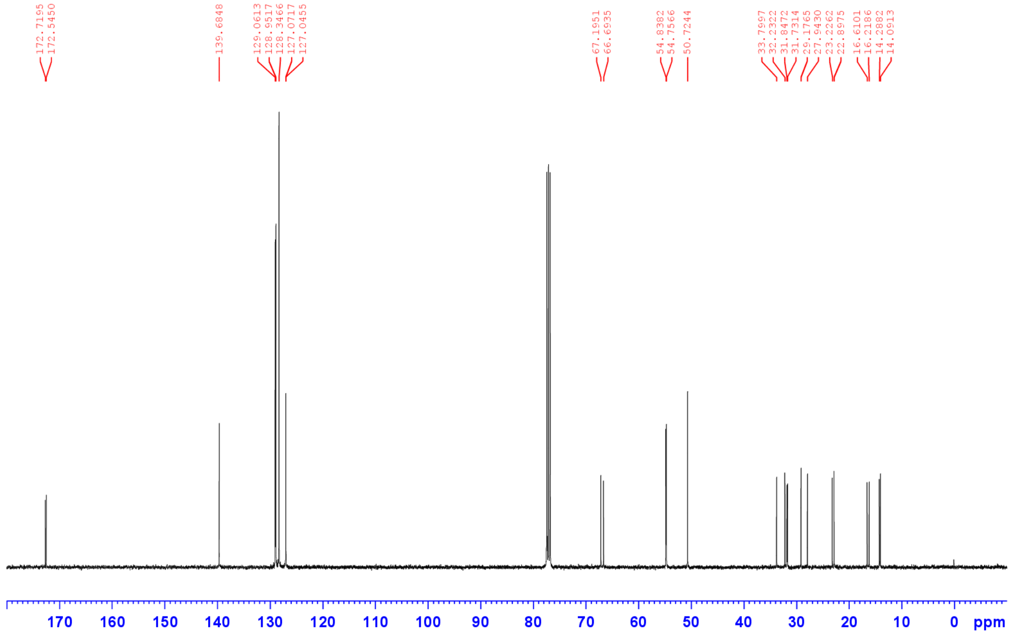

[^{1}H and ^{13}C{^{1}H} NMR Spectra of **3ra**]

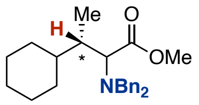

3ra

syn/anti = 46:54

^{1}H NMR
(400 MHz, $CDCl_3$)

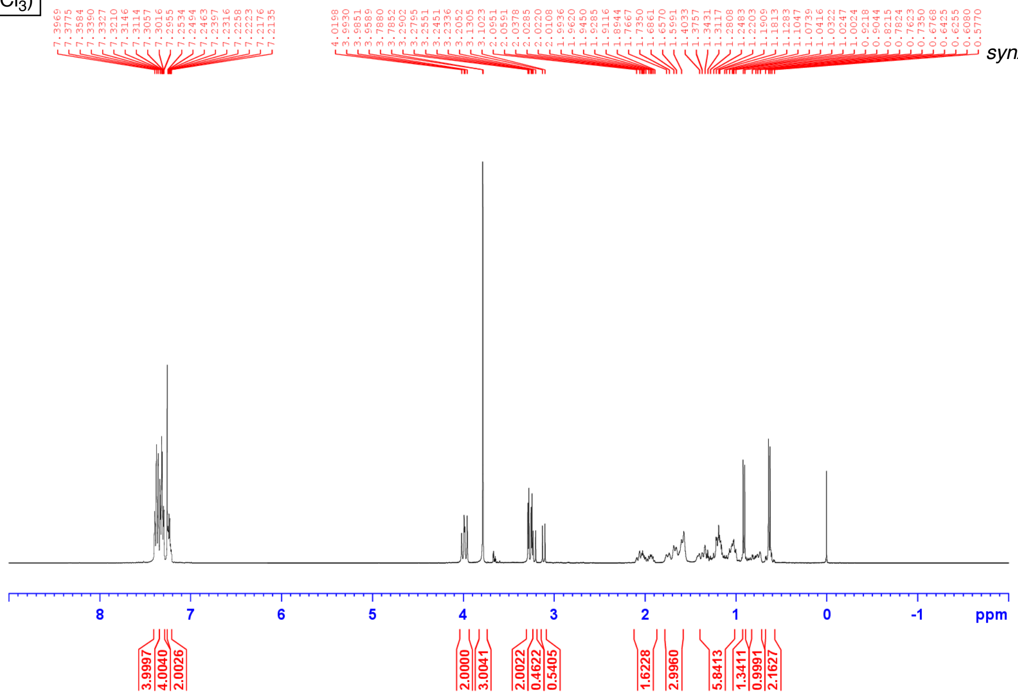

^{13}C{^{1}H} NMR
(100 MHz, $CDCl_3$)

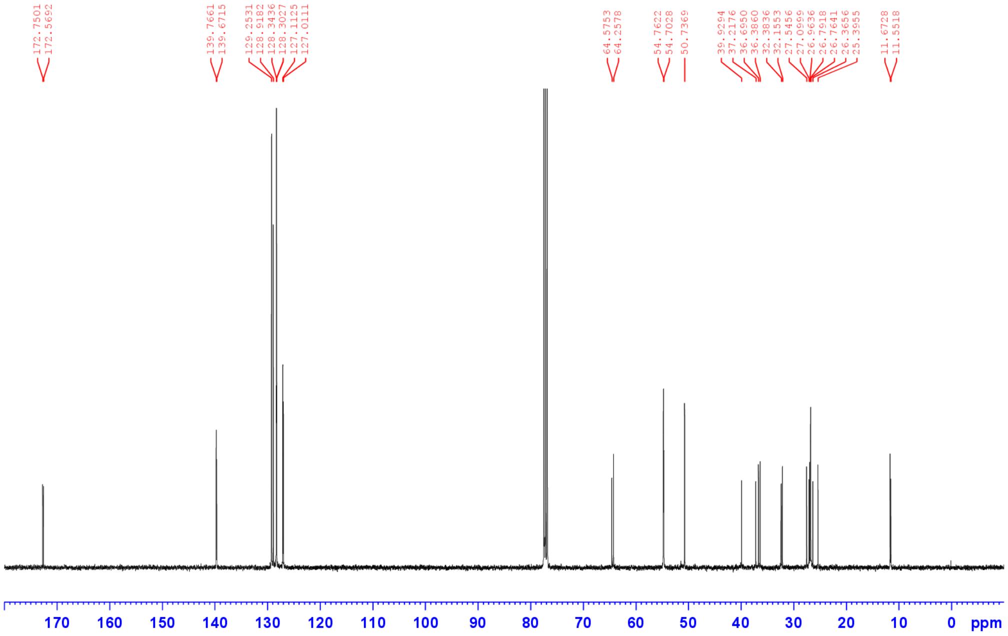

[^{1}H and $^{13}C\{^{1}H\}$ NMR Spectra of **3sa**]

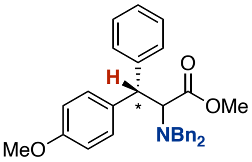

3sa

syn/anti = 49:51

^{1}H NMR
(400 MHz, $CDCl_3$)

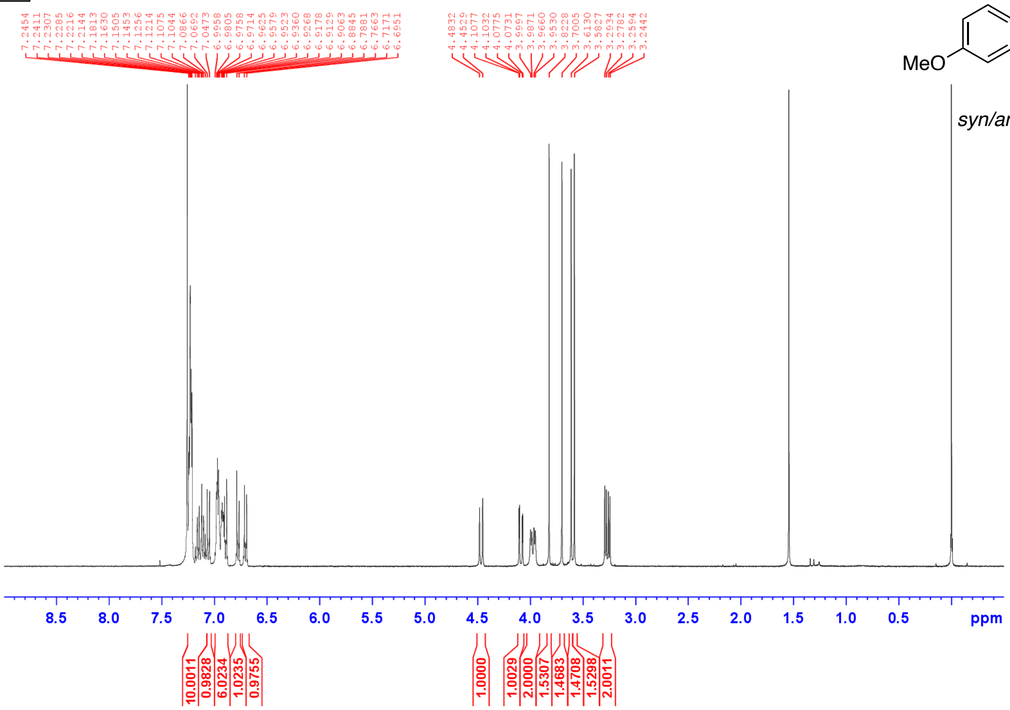

$^{13}C\{^{1}H\}$ NMR
(100 MHz, $CDCl_3$)

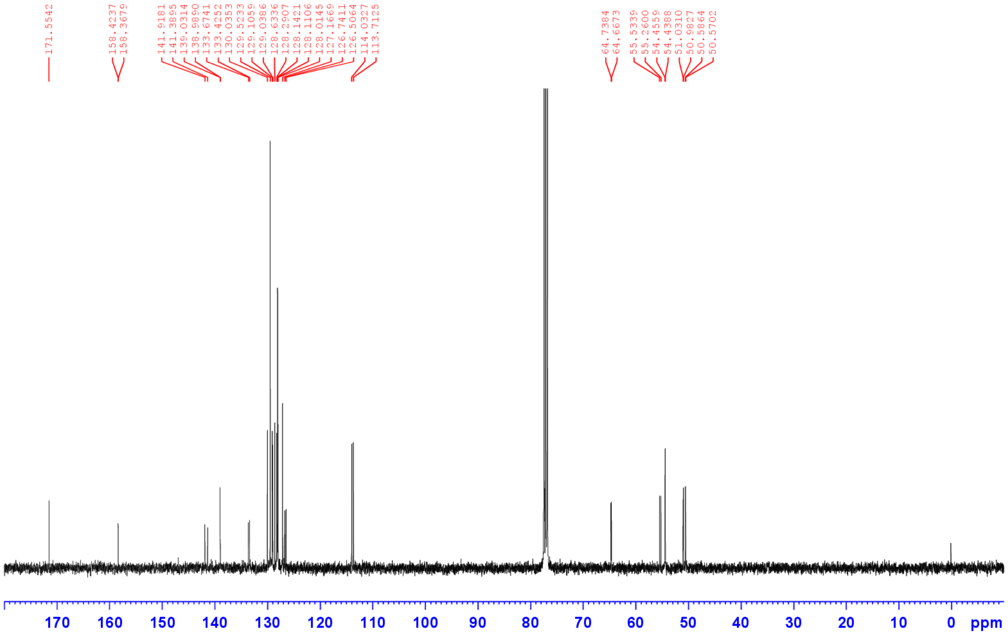

[^{1}H and $^{13}C\{^{1}H\}$ NMR Spectra of **3ta**]

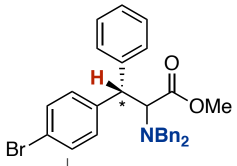

3ta

syn/anti = 49:51

^{1}H NMR
(400 MHz, $CDCl_3$)

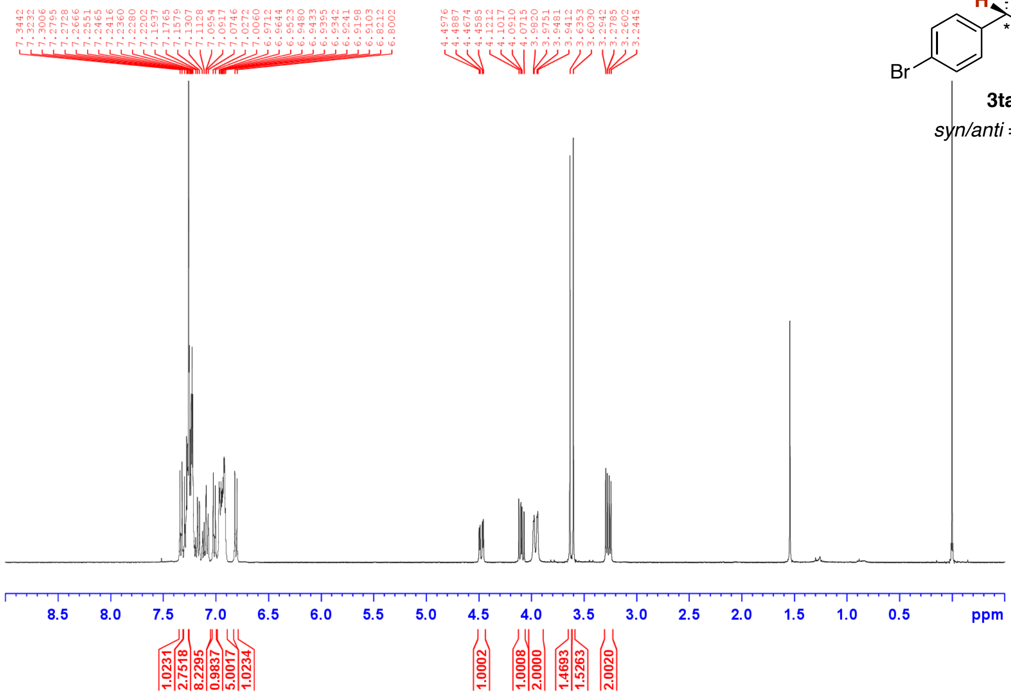

$^{13}C\{^{1}H\}$ NMR
(100 MHz, $CDCl_3$)

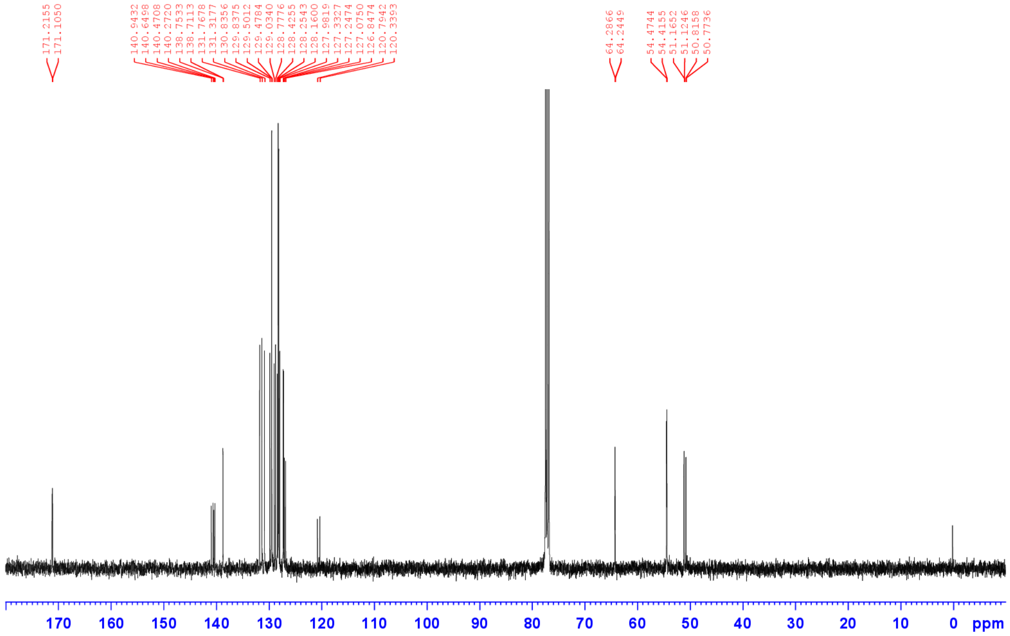

[^{1}H and ^{13}C{^{1}H} NMR Spectra of **3ua**]

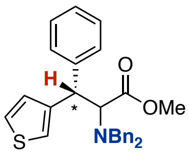

3ua

syn/anti = 45:55

^{1}H NMR
(400 MHz, $CDCl_3$)

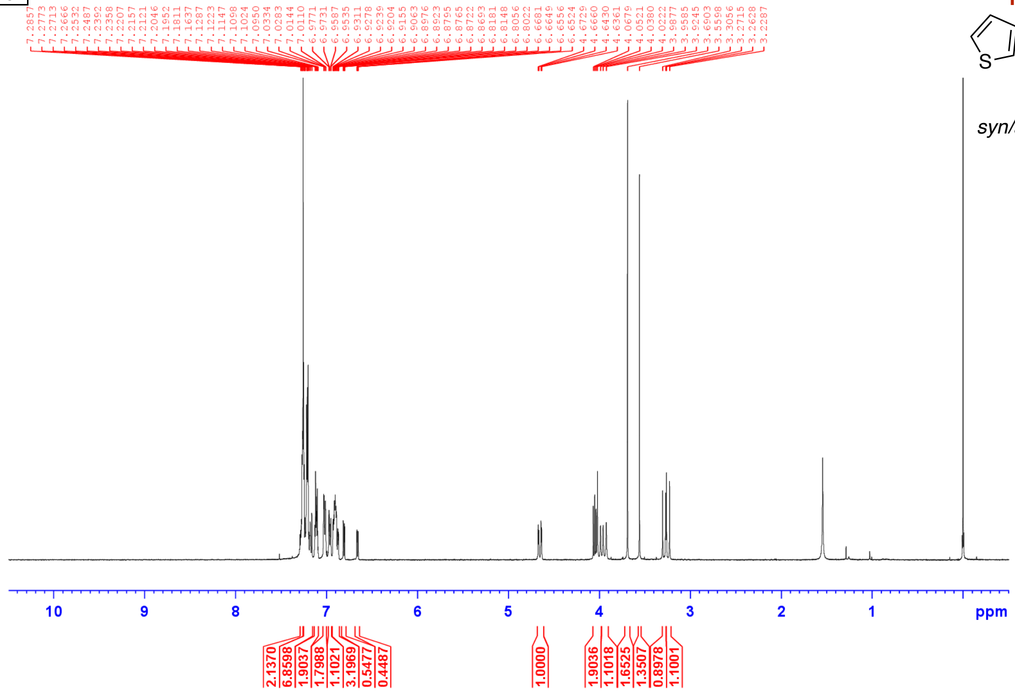

^{13}C{^{1}H} NMR
(100 MHz, $CDCl_3$)

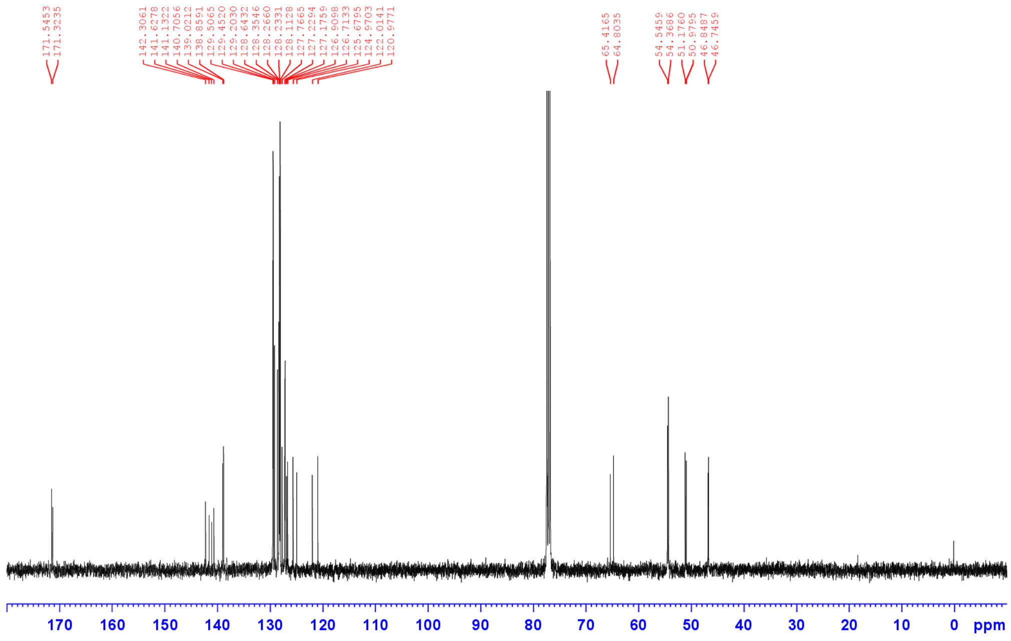

[^{1}H and $^{13}C\{^{1}H\}$ NMR Spectra of **3va**]

^{1}H NMR
(400 MHz, $CDCl_3$)

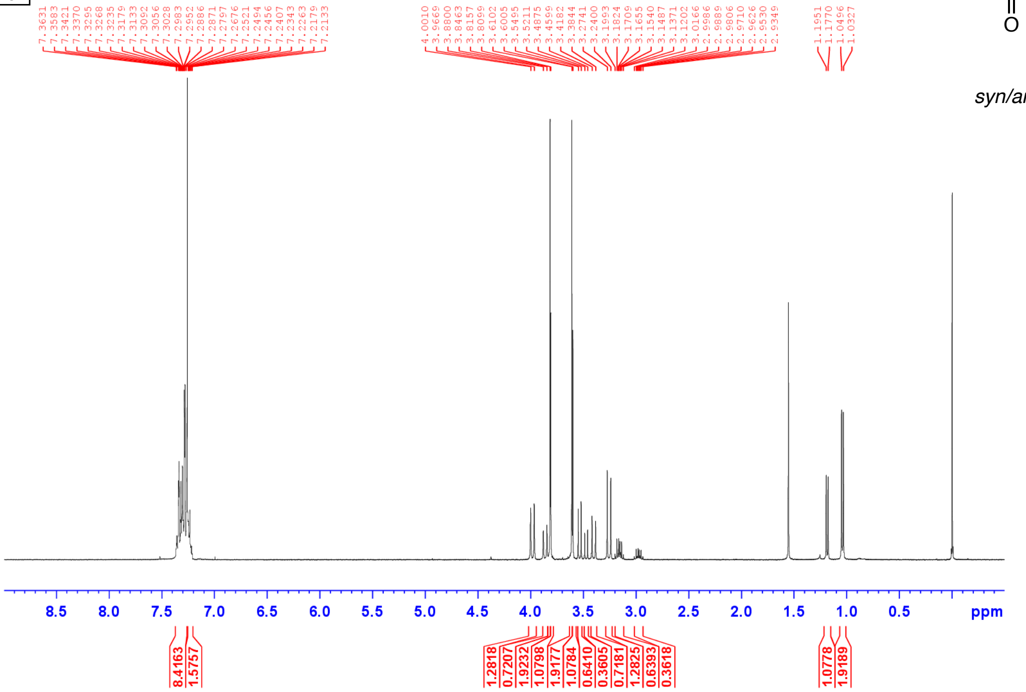

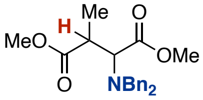

3va
syn/anti = 36:64

$^{13}C\{^{1}H\}$ NMR
(100 MHz, $CDCl_3$)

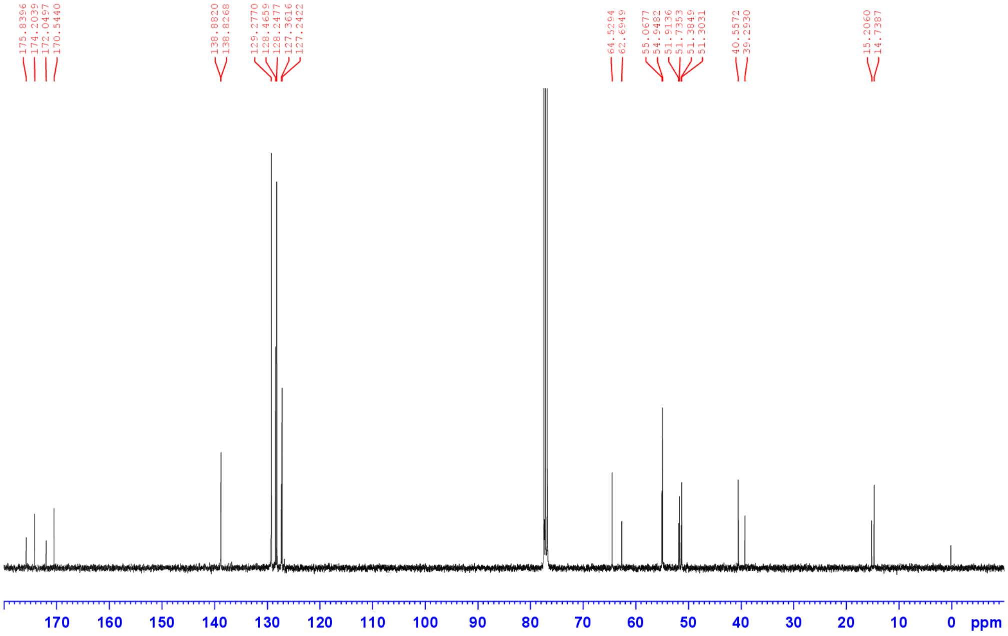

[^{1}H, $^{13}C\{^{1}H\}$, and ^{11}B NMR Spectra of **3wa**]

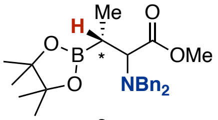

3wa

syn/anti = 79:21

^{1}H NMR
(400 MHz, $CDCl_3$)

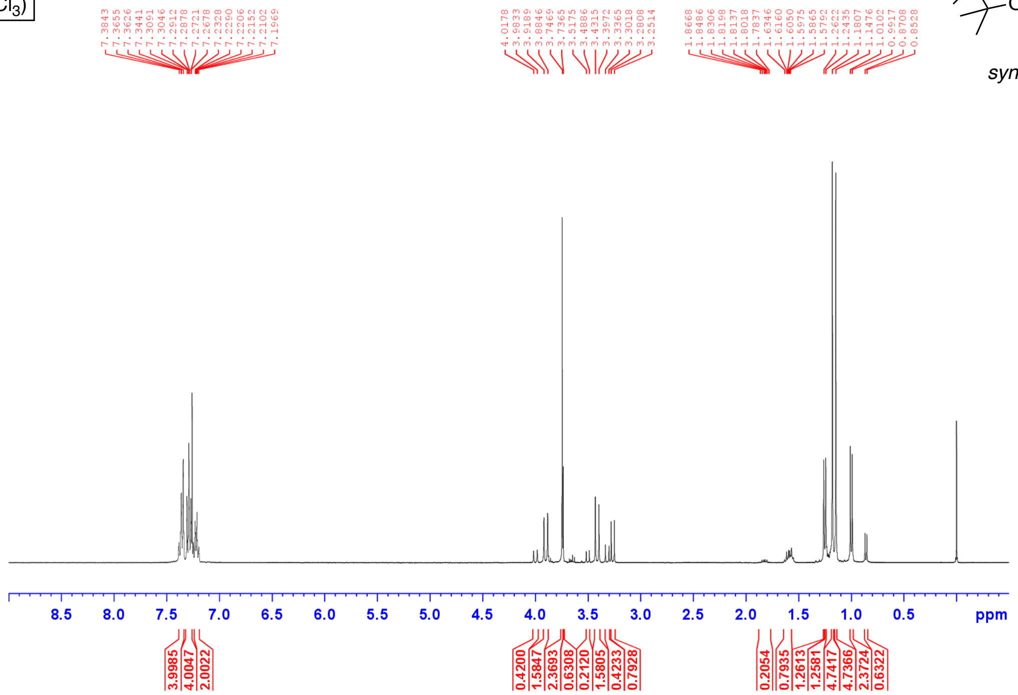

$^{13}C\{^{1}H\}$ NMR
(100 MHz, $CDCl_3$)

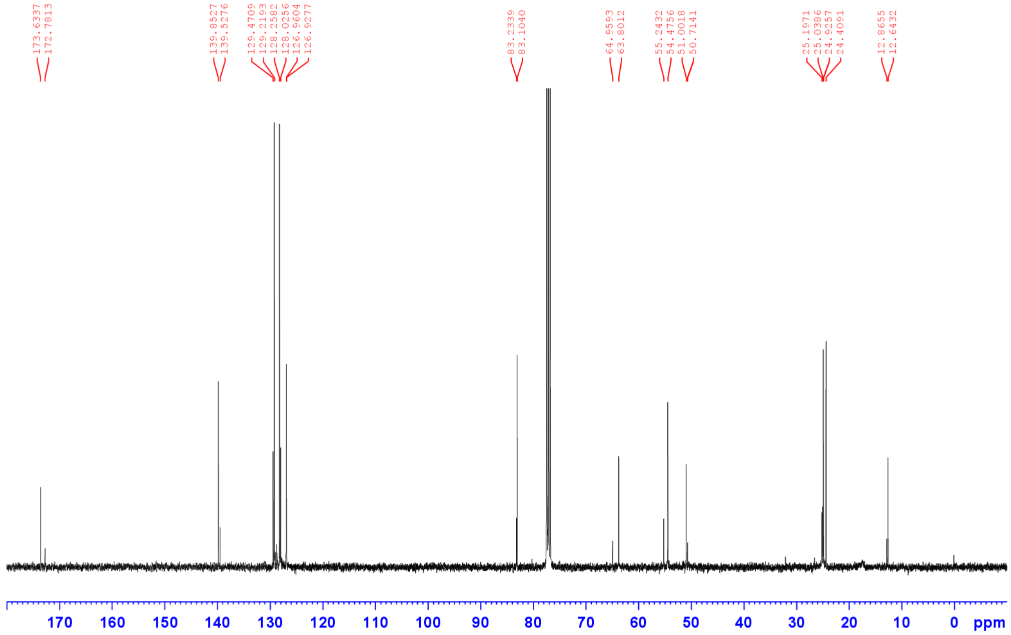

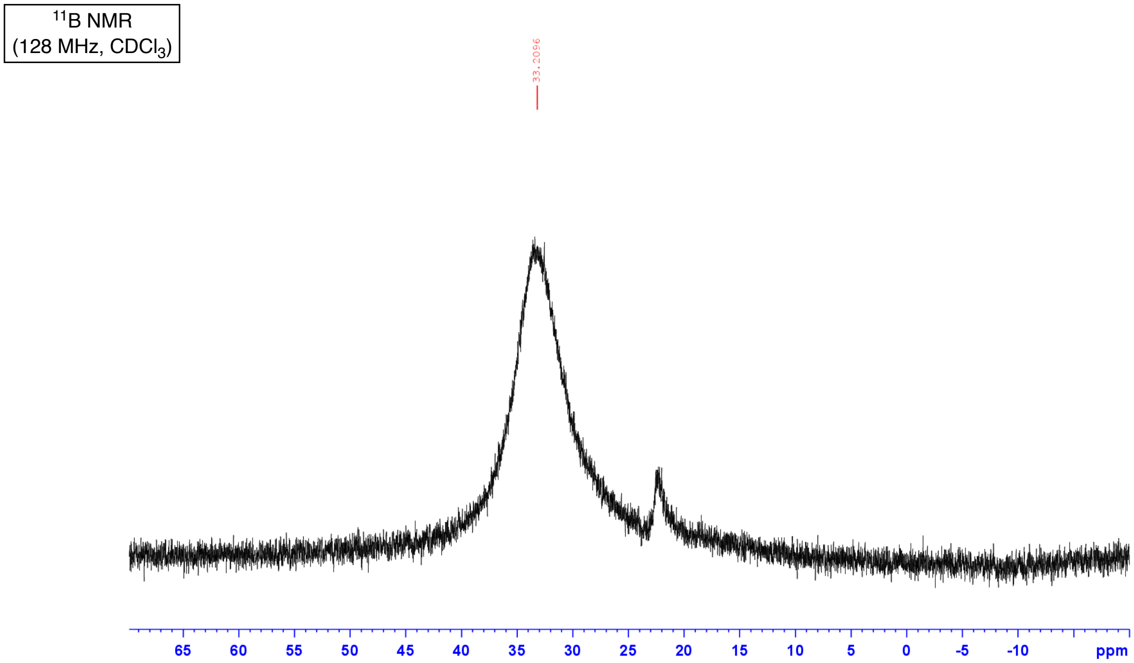
^{11}B NMR
(128 MHz, $CDCl_3$)
33.2096
65 60 55 50 45 40 35 30 25 20 15 10 5 0 -5 -10 ppm

[^{1}H and ^{13}C{^{1}H} NMR Spectra of **3xa**]

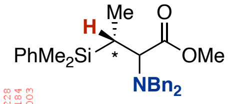

3xa

syn/anti = 66:34

^{1}H NMR
(400 MHz, $CDCl_3$)

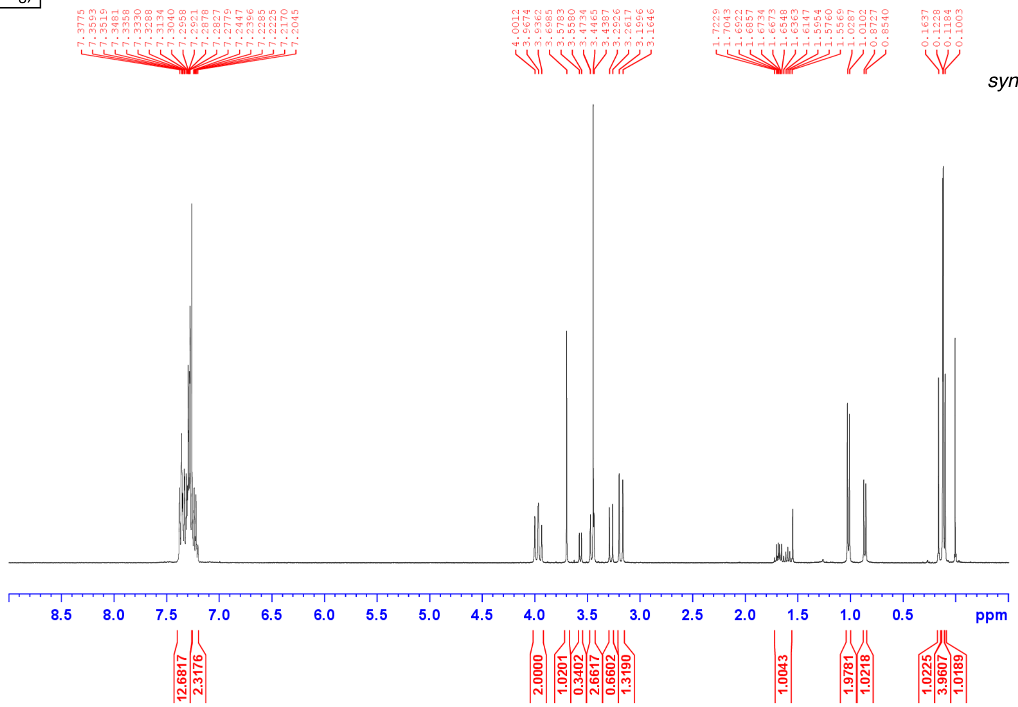

^{13}C{^{1}H} NMR
(100 MHz, $CDCl_3$)

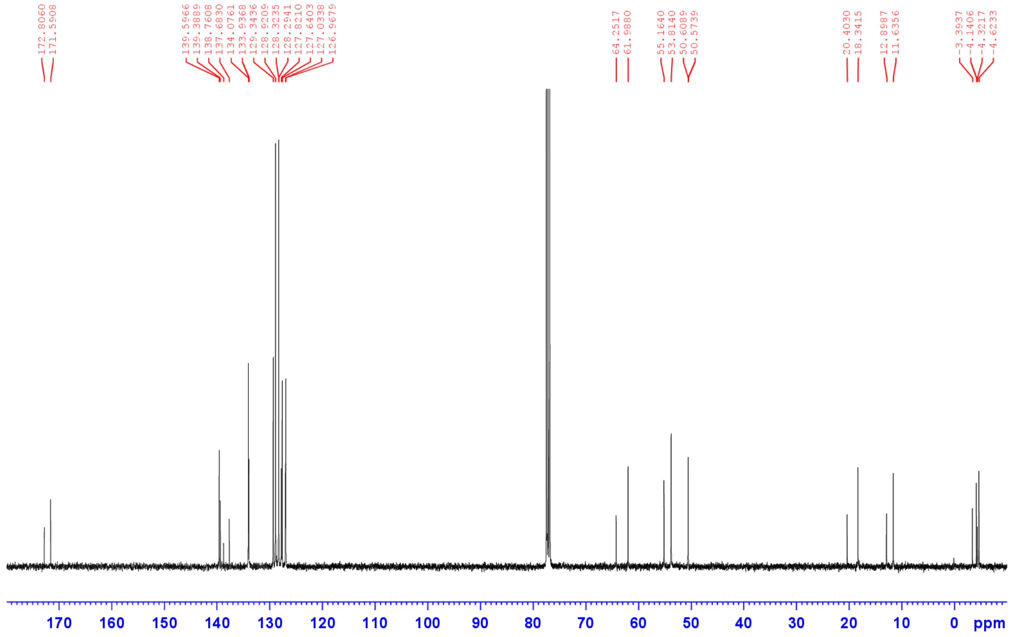

[^{1}H and ^{13}C{^{1}H} NMR Spectra of **3ya**]

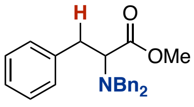

3ya

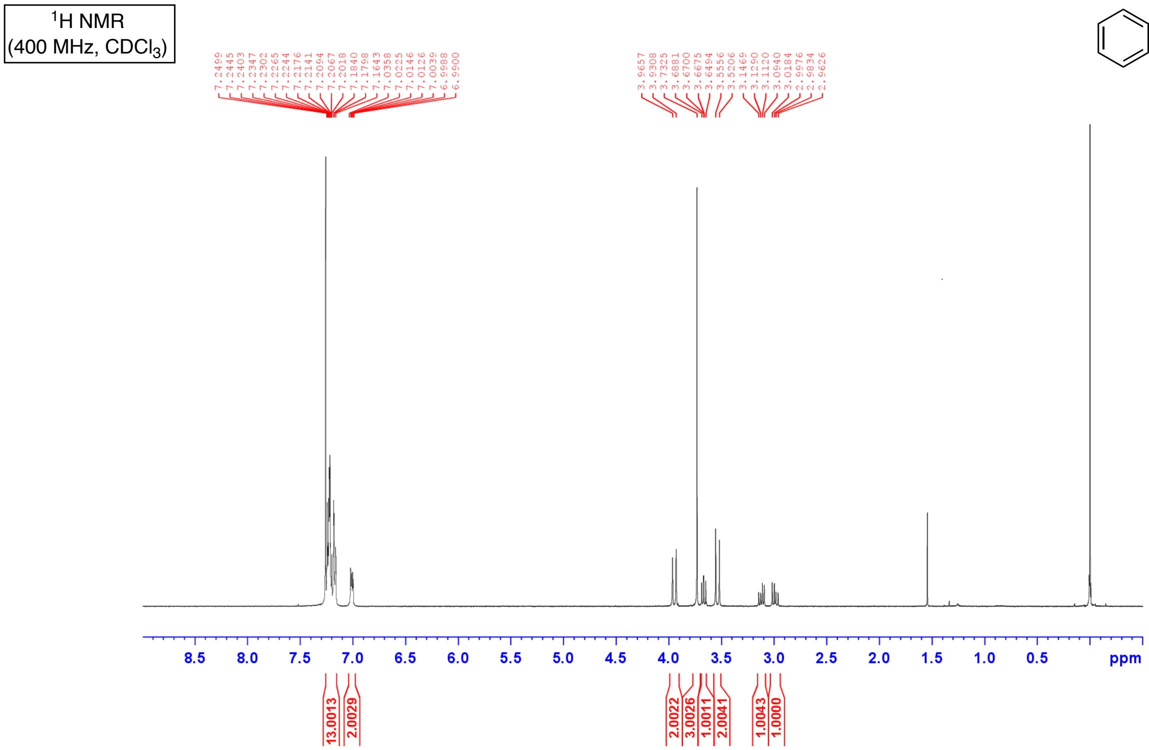

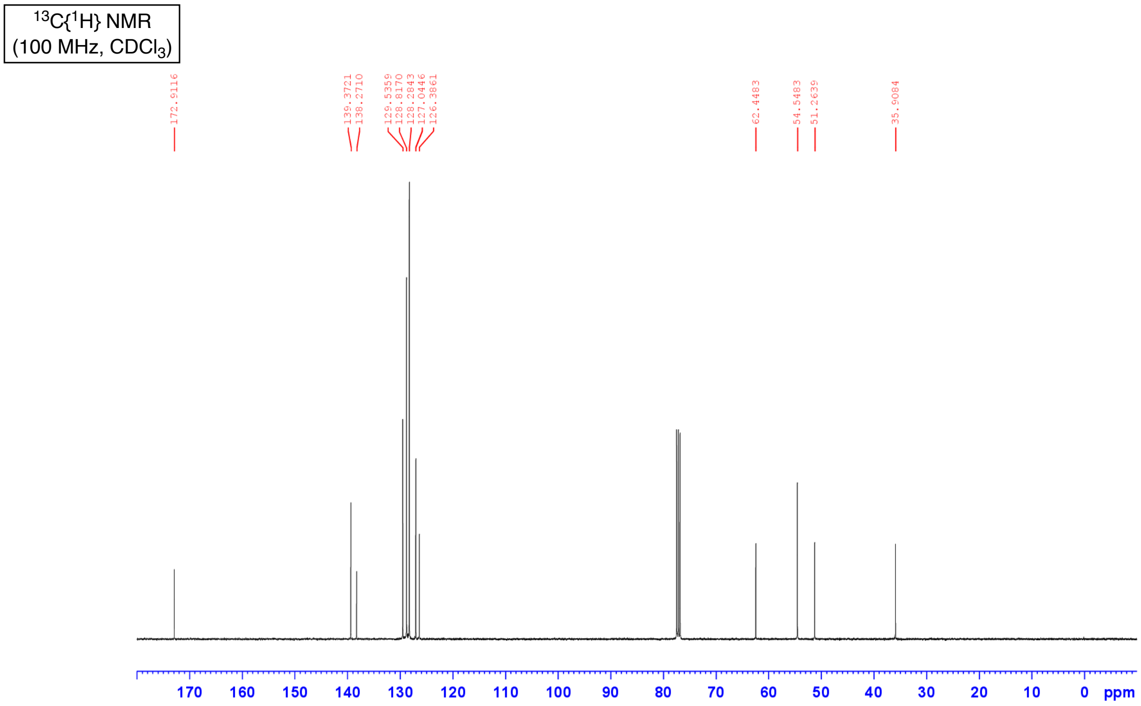

[^{1}H and ^{13}C{^{1}H} NMR Spectra of **3za**]

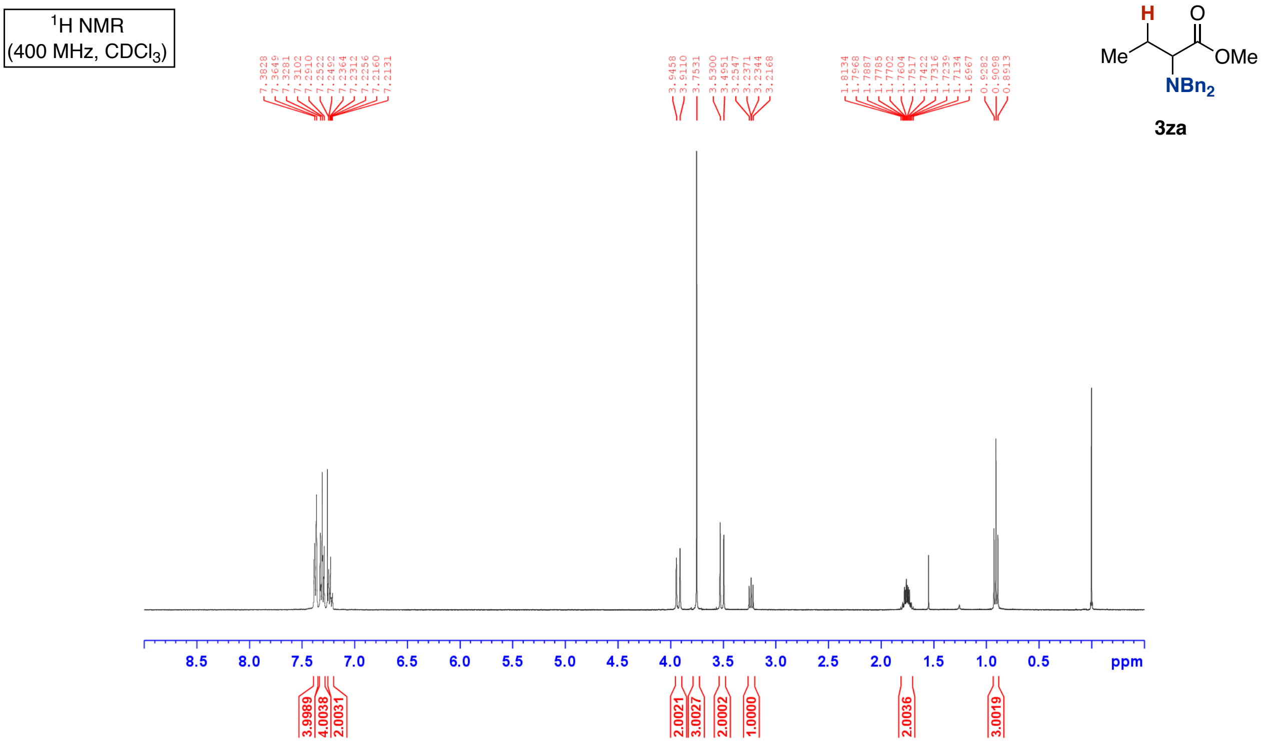

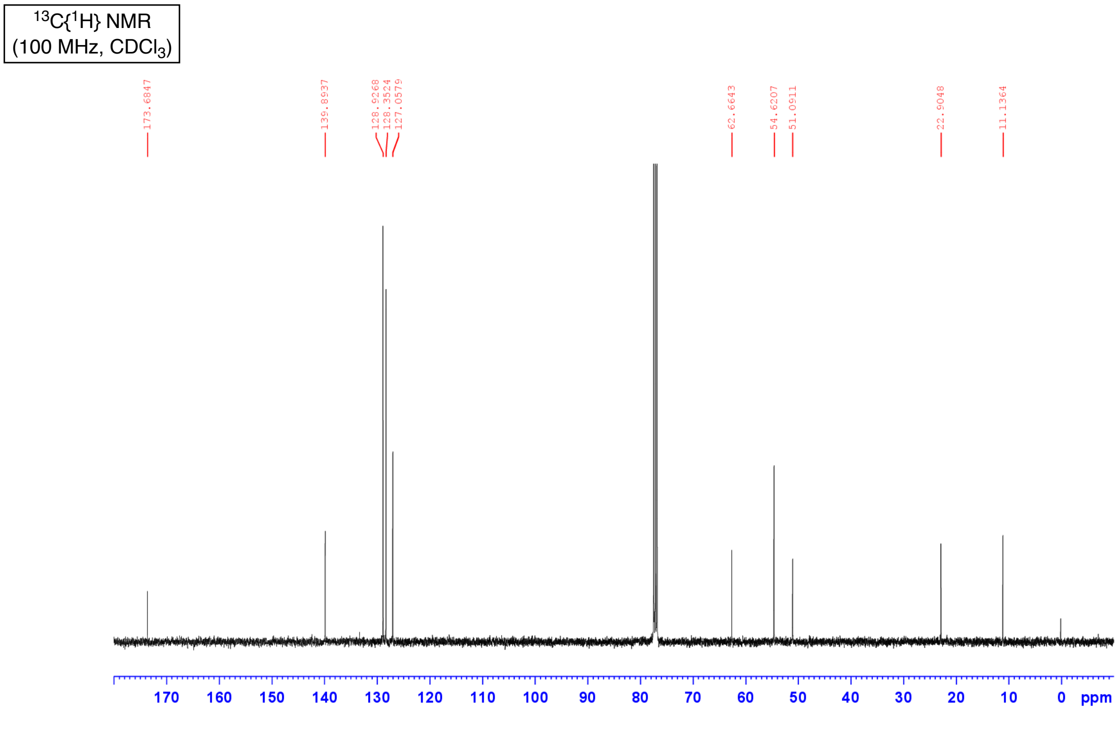

[^{1}H and ^{13}C{^{1}H} NMR Spectra of **3Aa**]

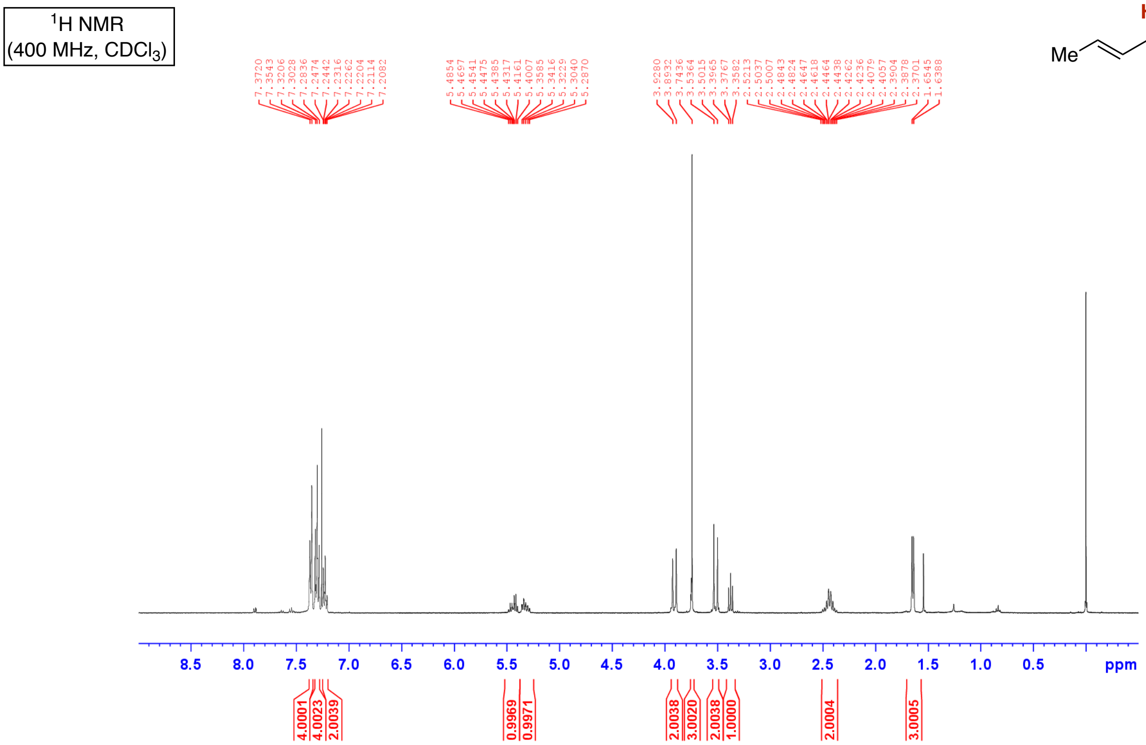

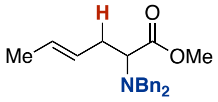

3Aa

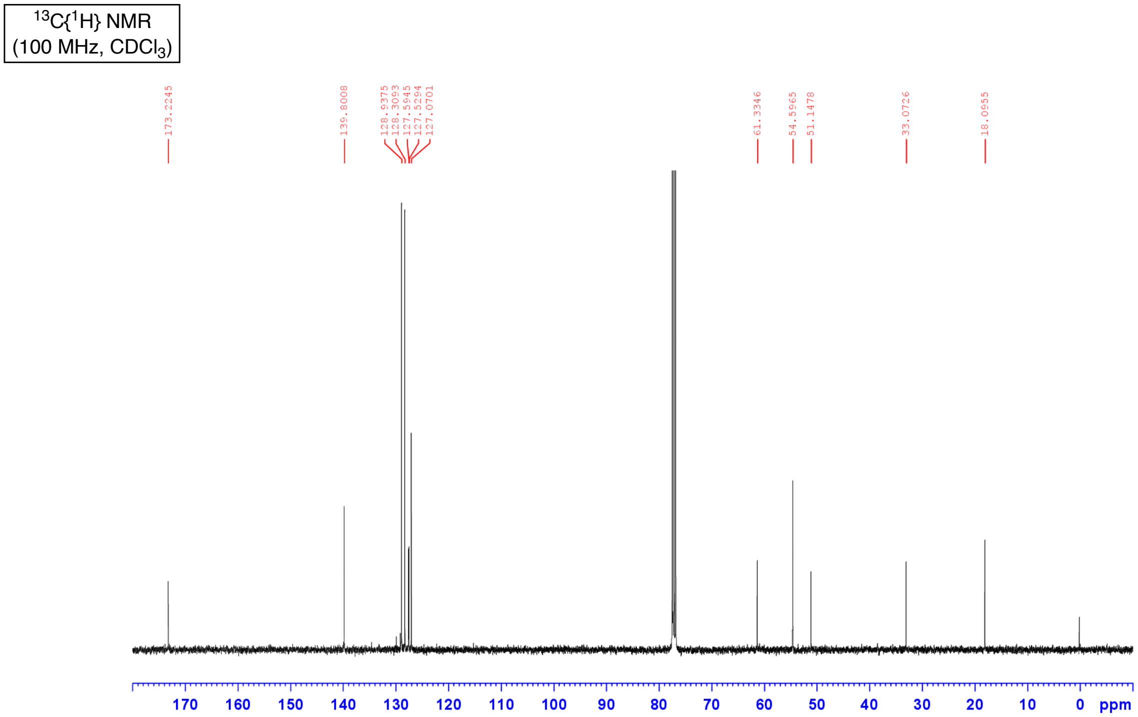

[^{1}H and ^{13}C{^{1}H} NMR Spectra of **3ab**]

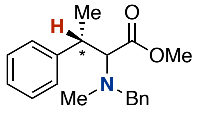

3ab

syn/anti = 34:66

^{1}H NMR
(400 MHz, $CDCl_3$)

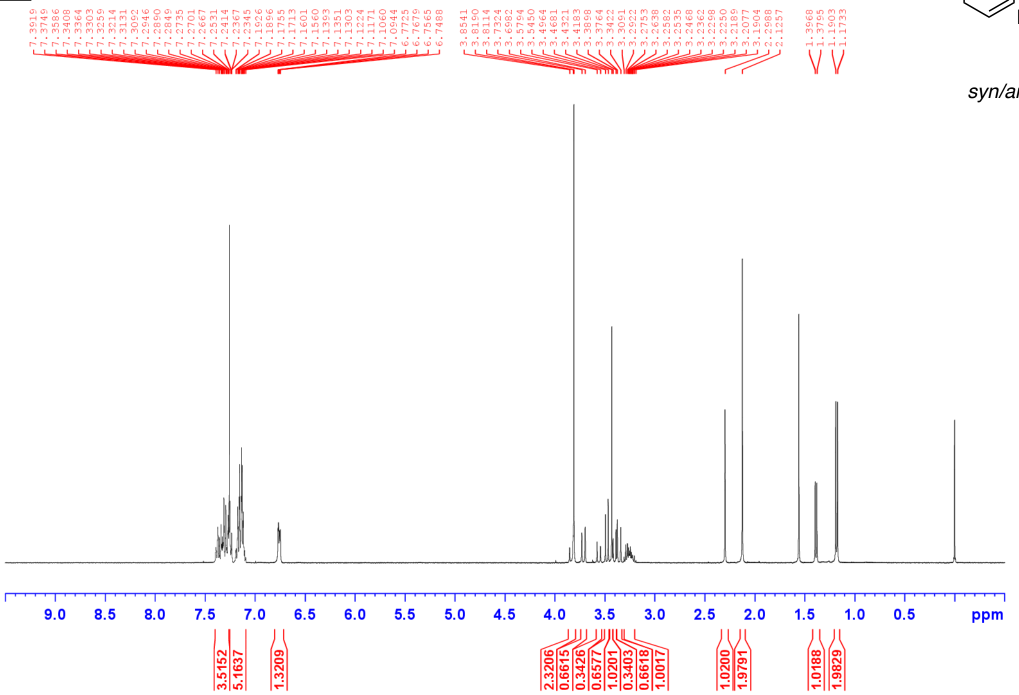

^{13}C{^{1}H} NMR
(100 MHz, $CDCl_3$)

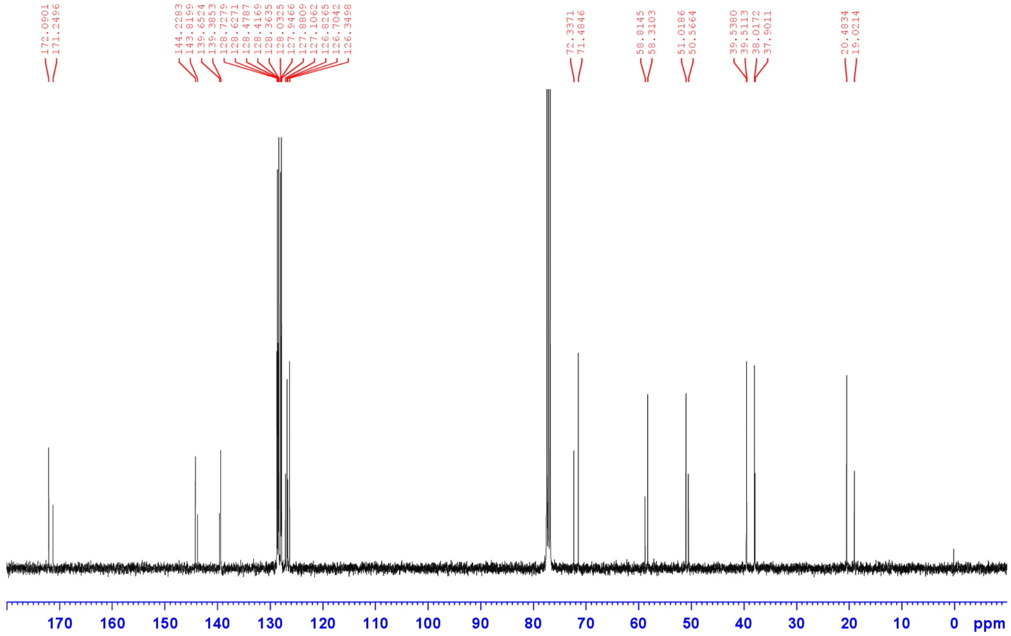

[^{1}H and ^{13}C{^{1}H} NMR Spectra of **3ac**]

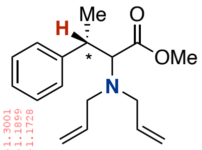

3ac

syn/anti = 33:67

^{1}H NMR
(400 MHz, $CDCl_3$)

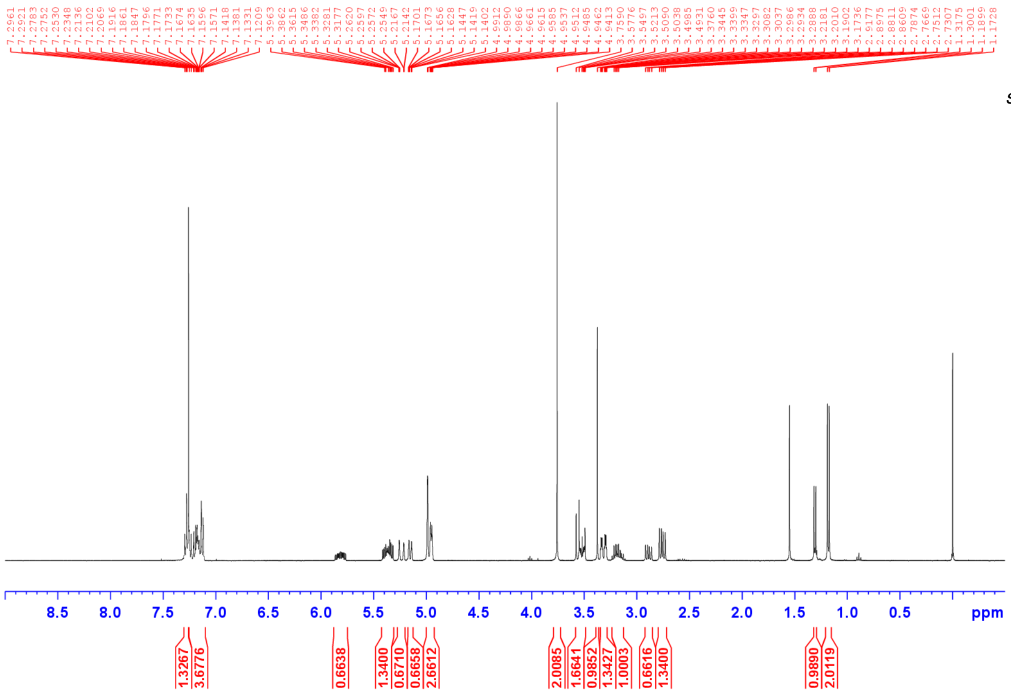

^{13}C{^{1}H} NMR
(100 MHz, $CDCl_3$)

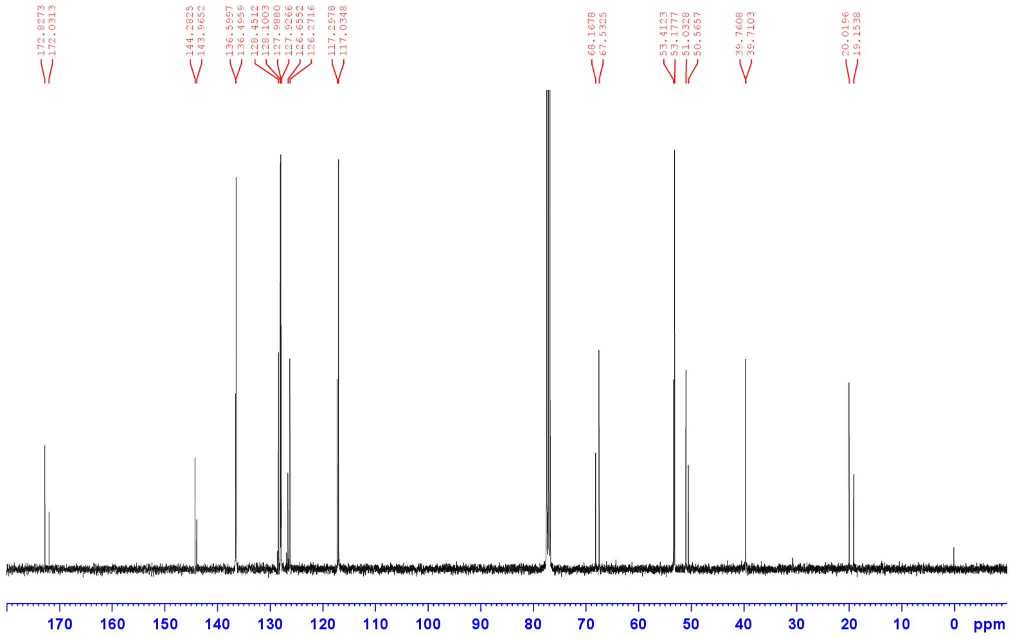

[^{1}H and ^{13}C{^{1}H} NMR Spectra of **3ad**]

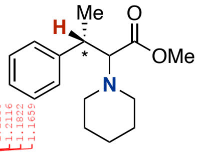

3ad

syn/anti = 35:65

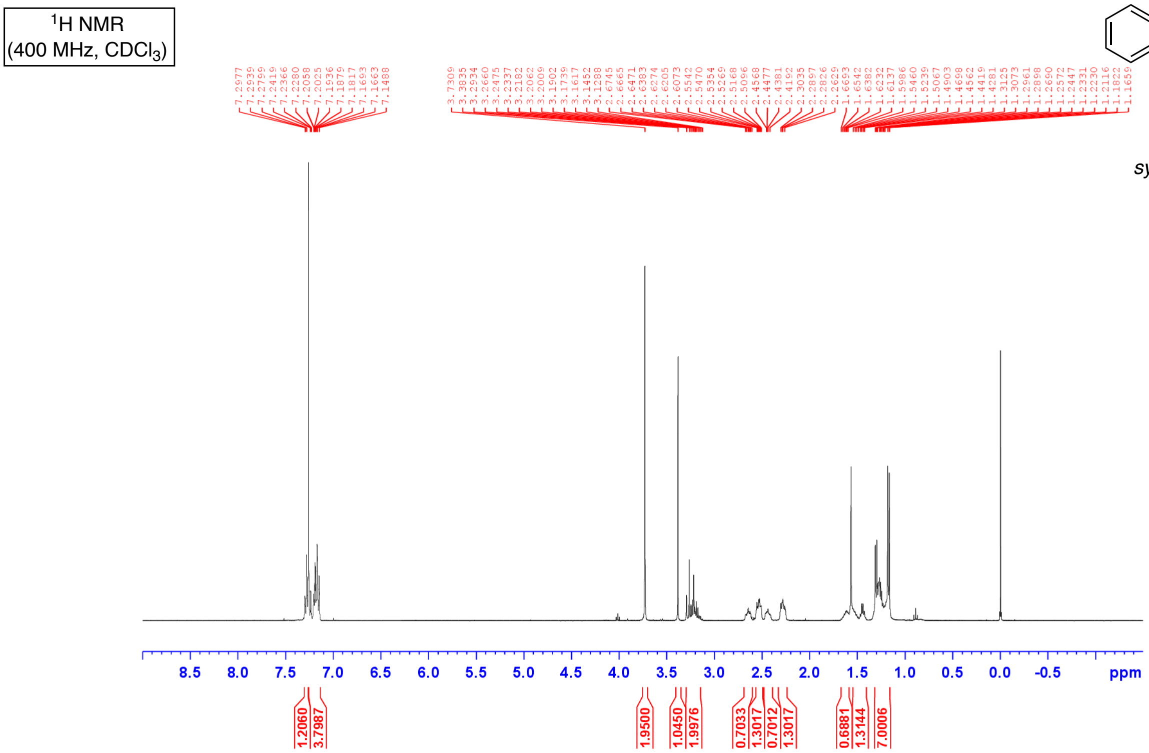

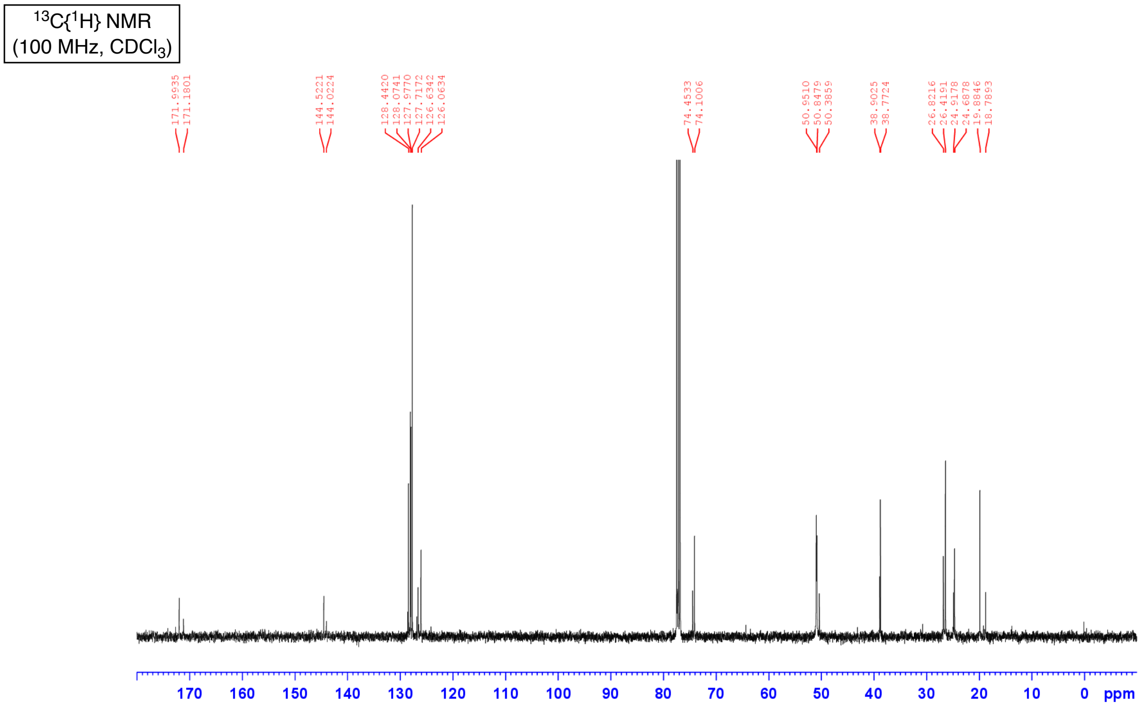

[^{1}H and $^{13}C\{^{1}H\}$ NMR Spectra of **3ae**]

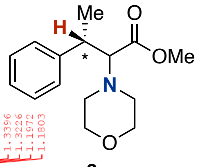

3ae

syn/anti = 34:66

^{1}H NMR
(400 MHz, $CDCl_3$)

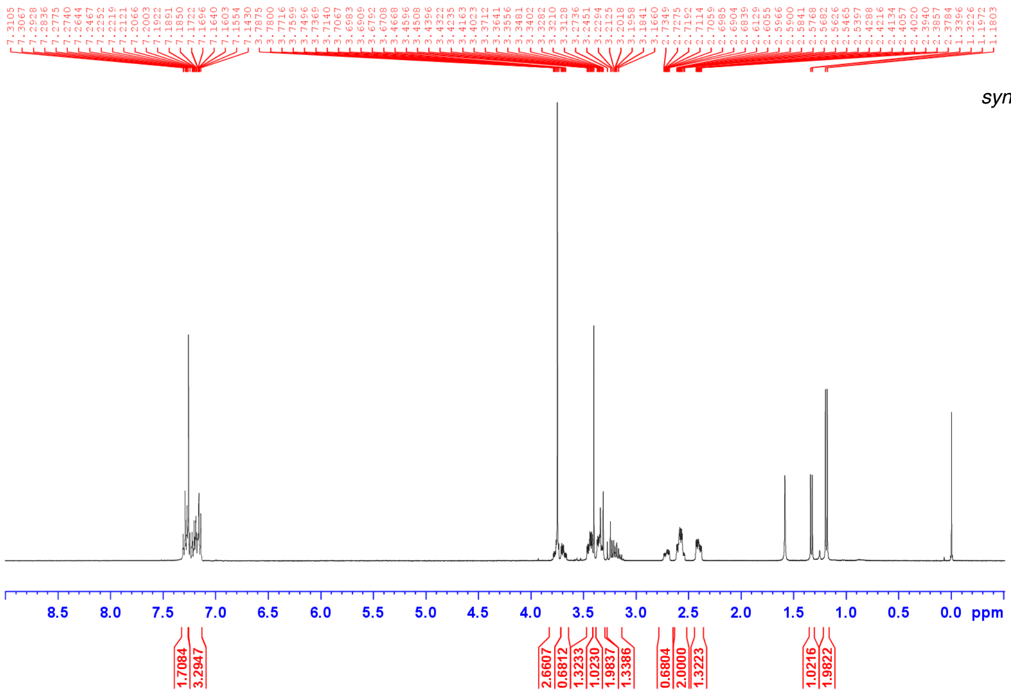

$^{13}C\{^{1}H\}$ NMR
(100 MHz, $CDCl_3$)

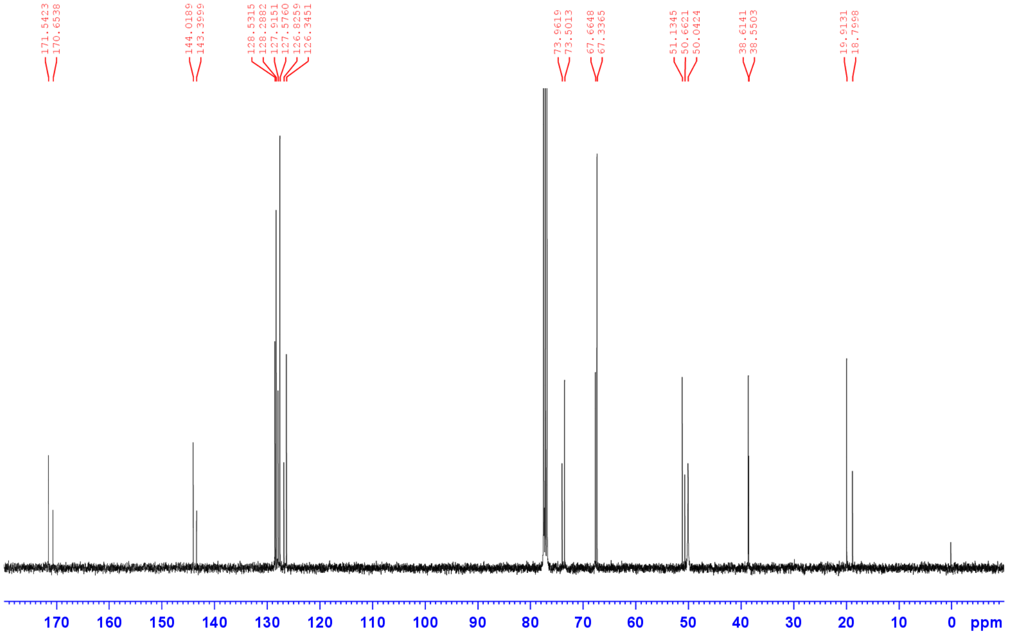

[^{1}H and ^{13}C{^{1}H} NMR Spectra of **3bf**]

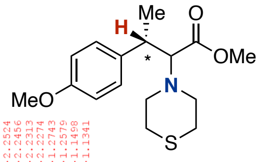

3bf

syn/anti = 34:66

^{1}H NMR
(400 MHz, $CDCl_3$)

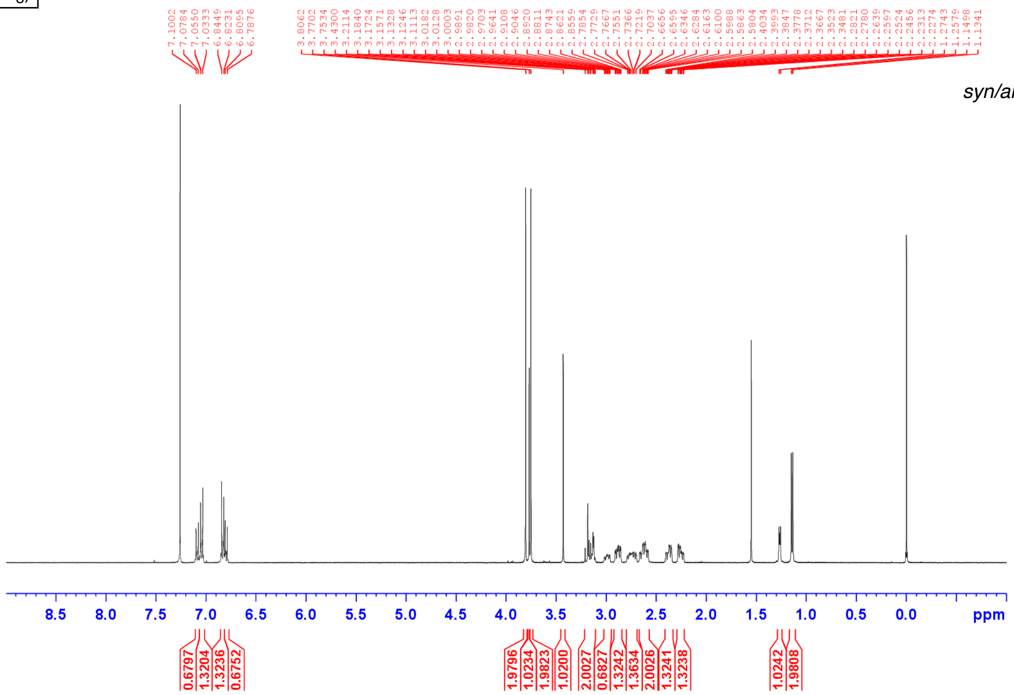

^{13}C{^{1}H} NMR
(100 MHz, $CDCl_3$)

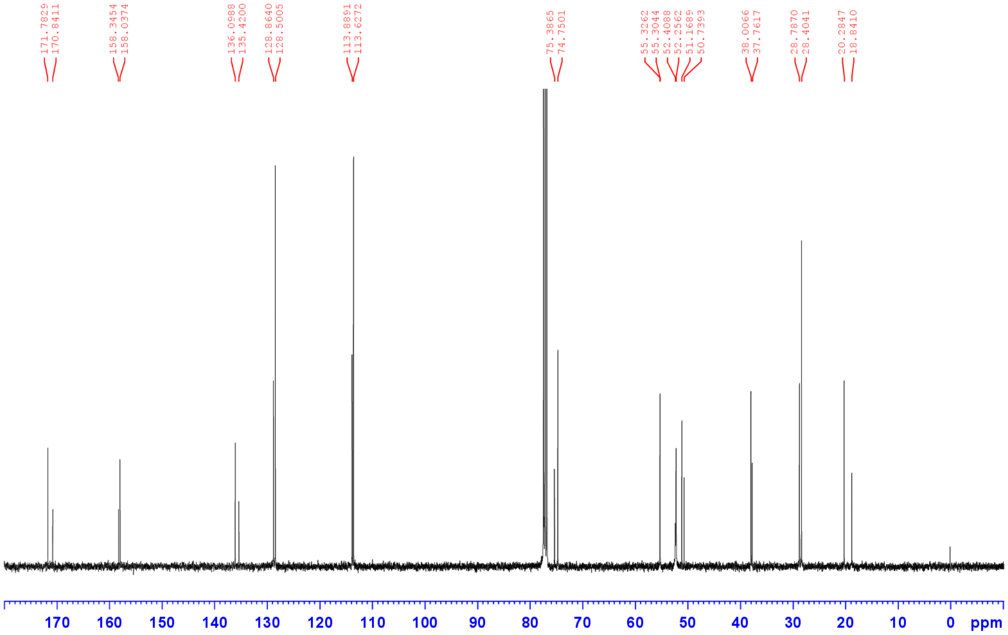

[^{1}H and ^{13}C{^{1}H} NMR Spectra of **3ag**]

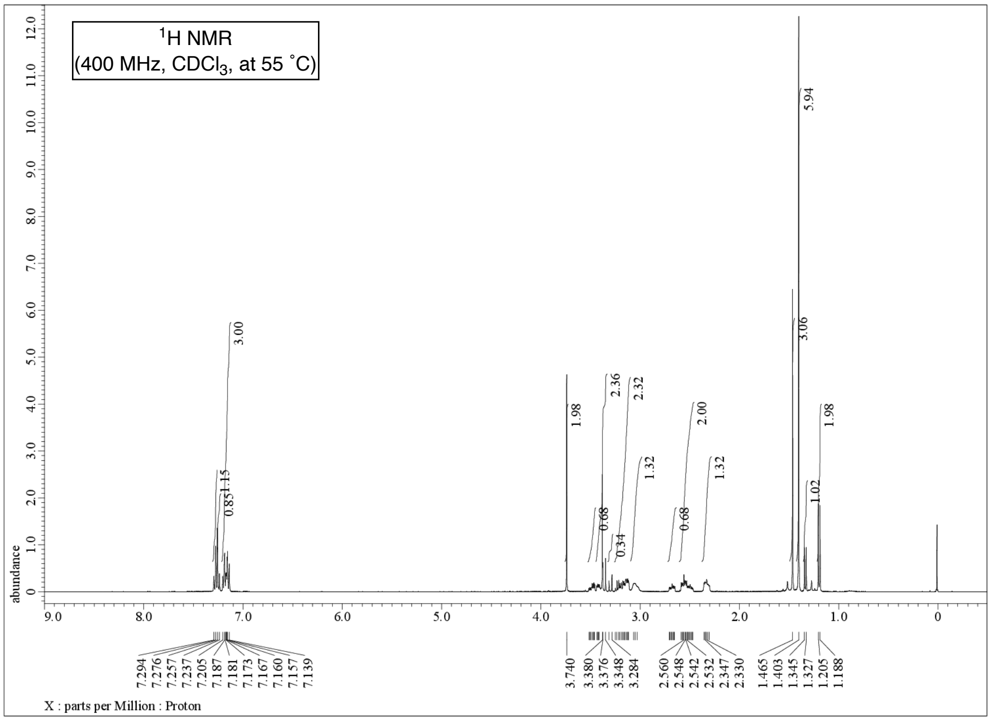

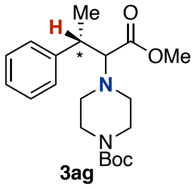

3ag

syn/anti = 34:66

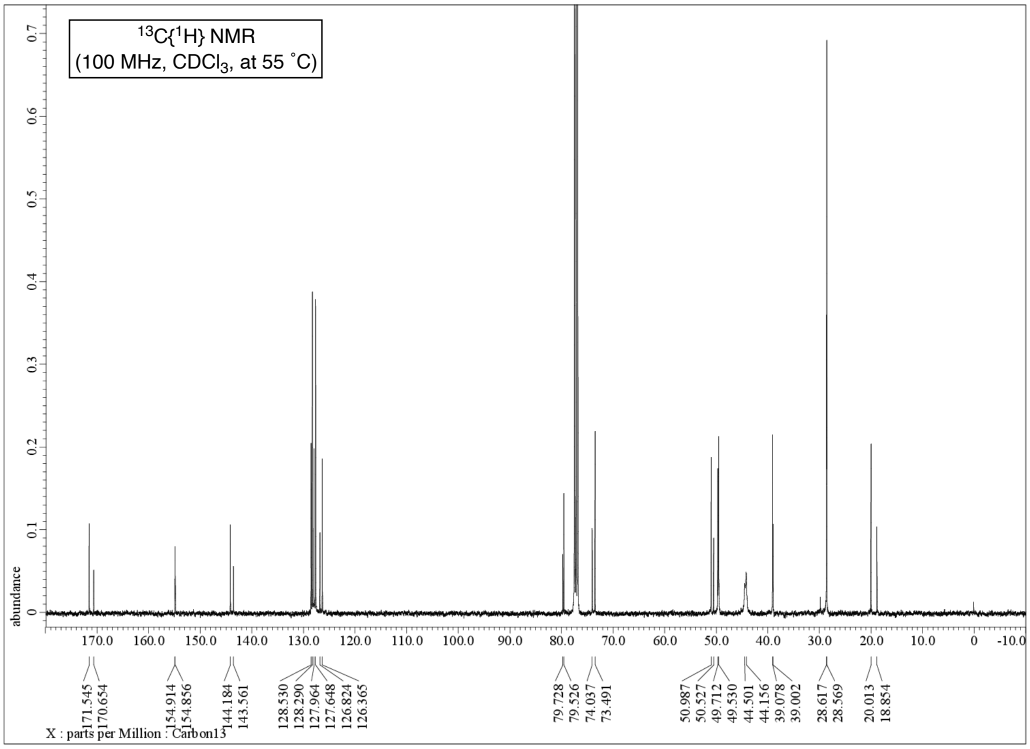

[^{1}H and ^{13}C{^{1}H} NMR Spectra of **3ah**]

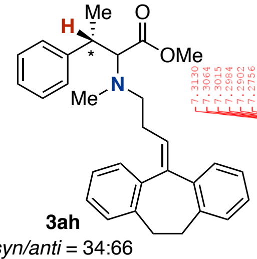

3ah

syn/anti = 34:66

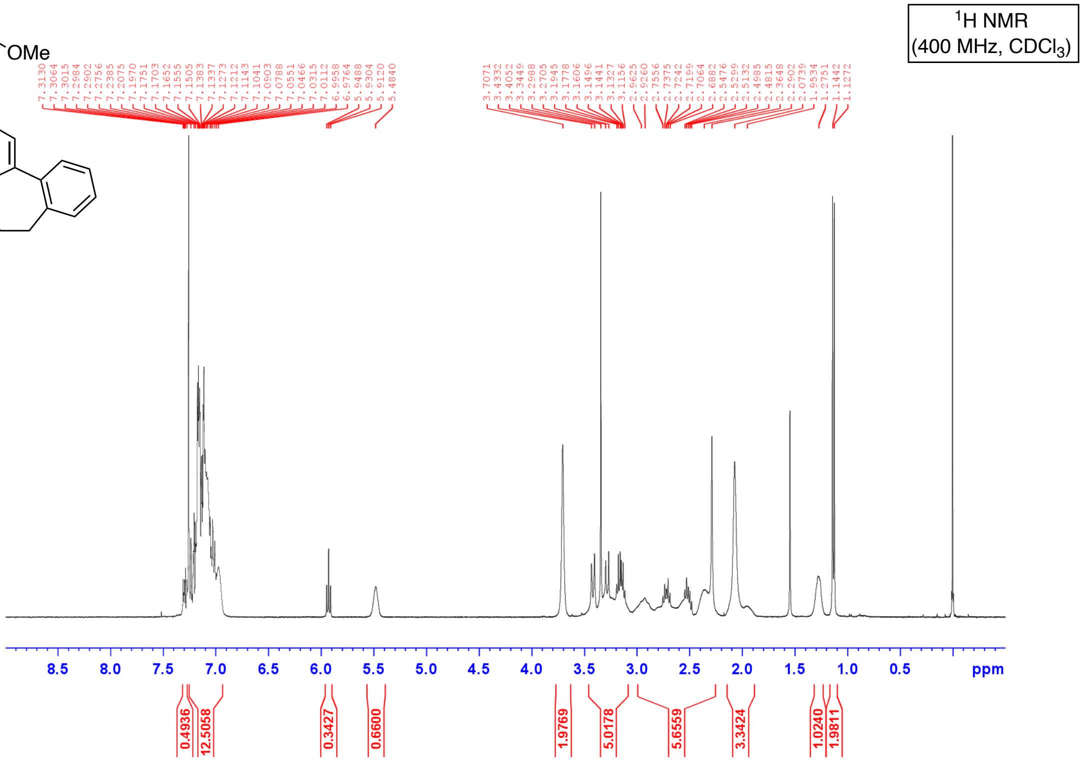

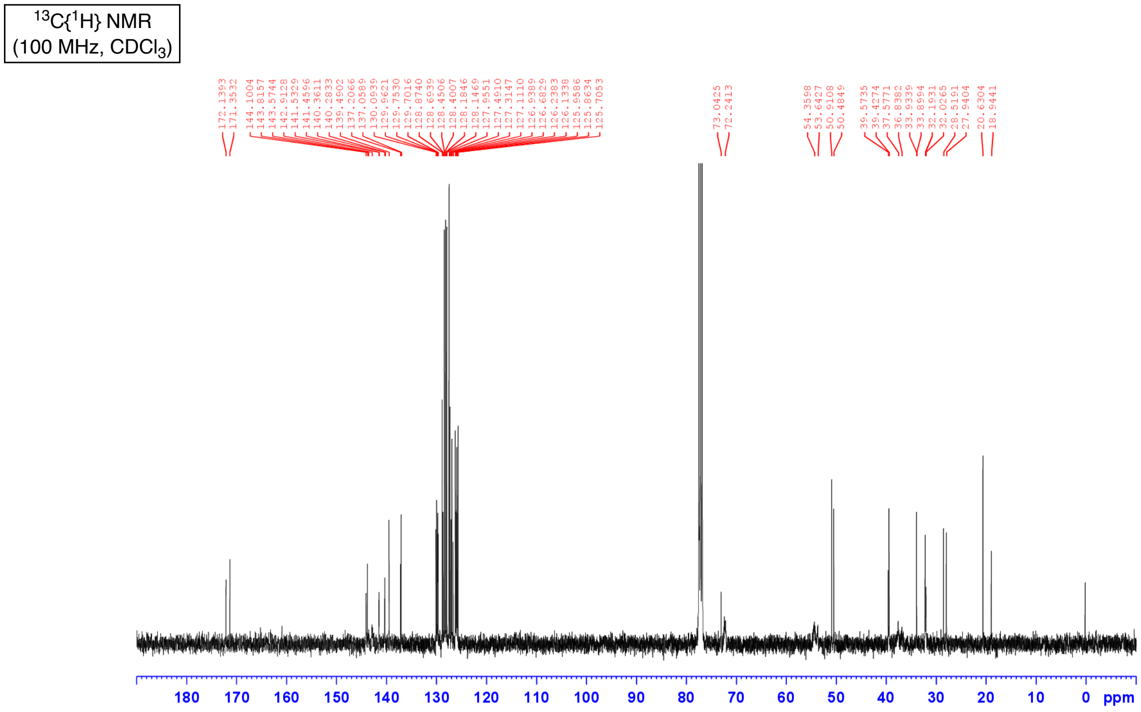

[^{1}H and ^{13}C{^{1}H} NMR Spectra of **3bi**]

^{1}H NMR
(400 MHz, $CDCl_3$)

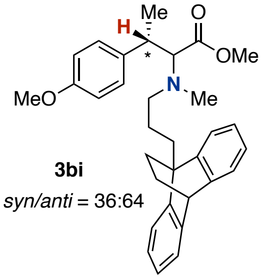

3bi

syn/anti = 36:64

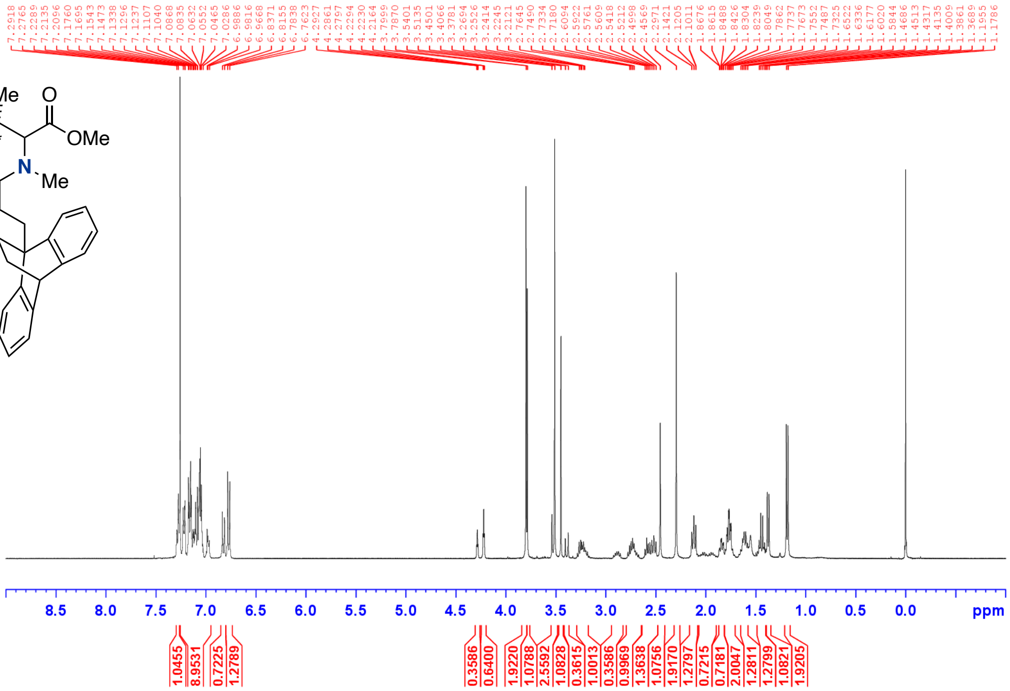

^{13}C{^{1}H} NMR
(100 MHz, $CDCl_3$)

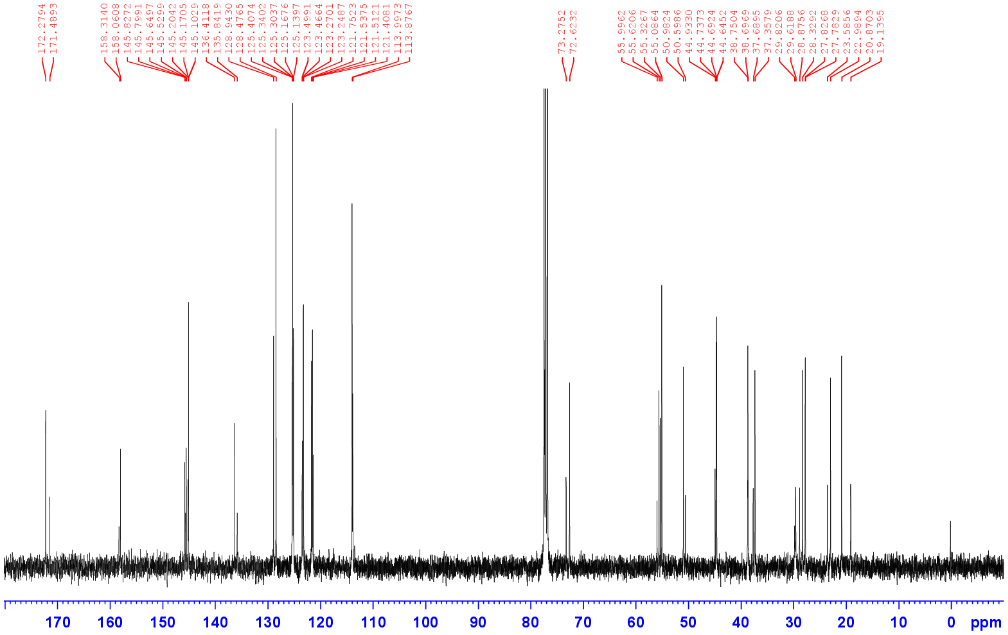

[^{1}H and ^{13}C{^{1}H} NMR Spectra of **3bj**]

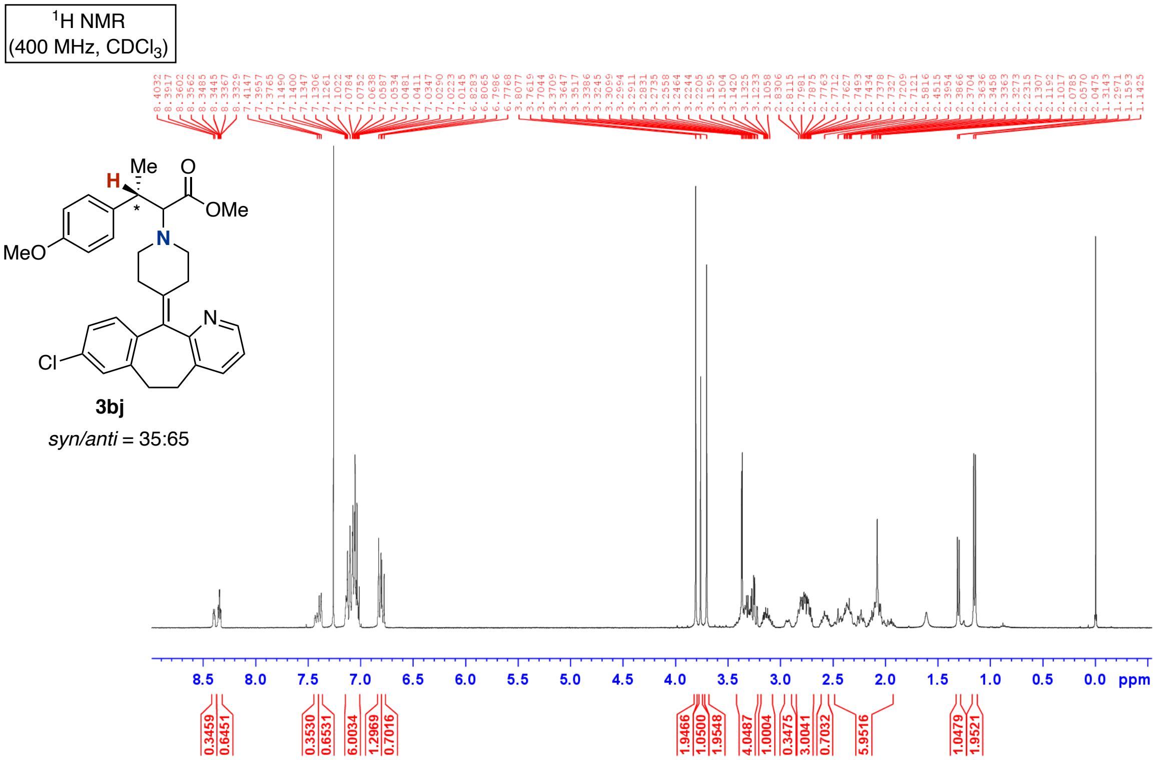

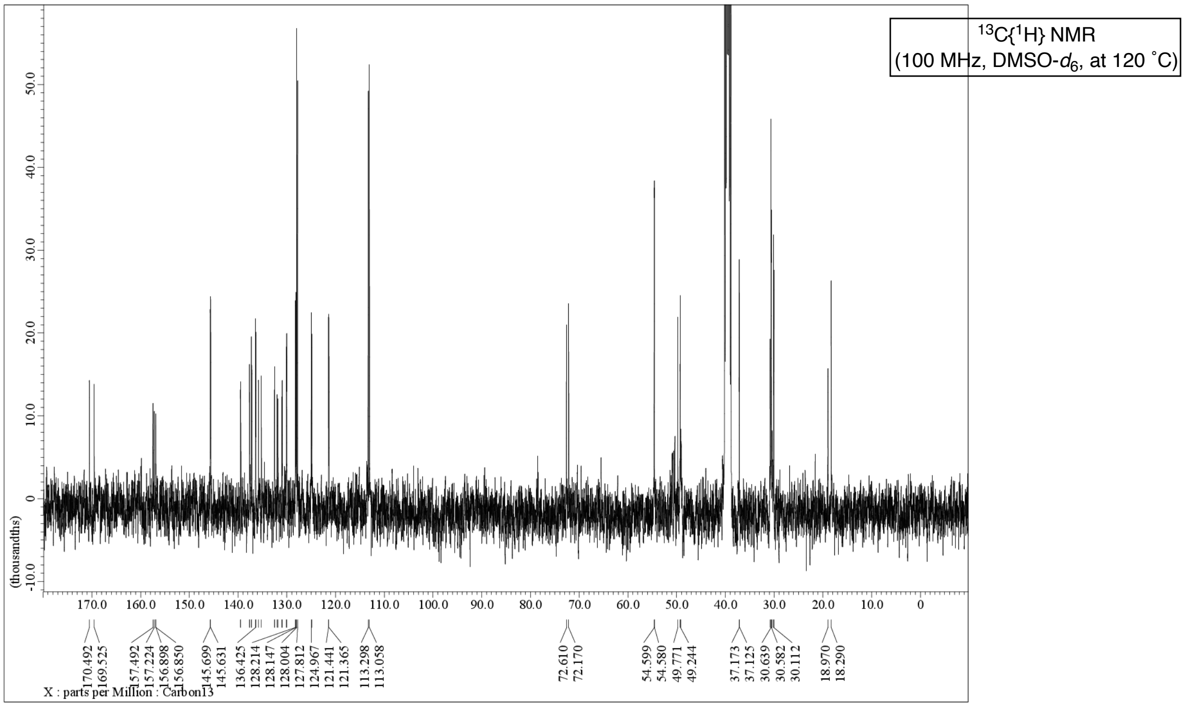

[^{1}H and ^{13}C{^{1}H} NMR Spectra of **3ak**]

^{1}H NMR (400 MHz, $CDCl_3$)

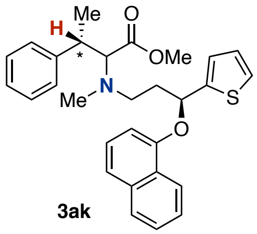

3ak

30:1:66:3 dr

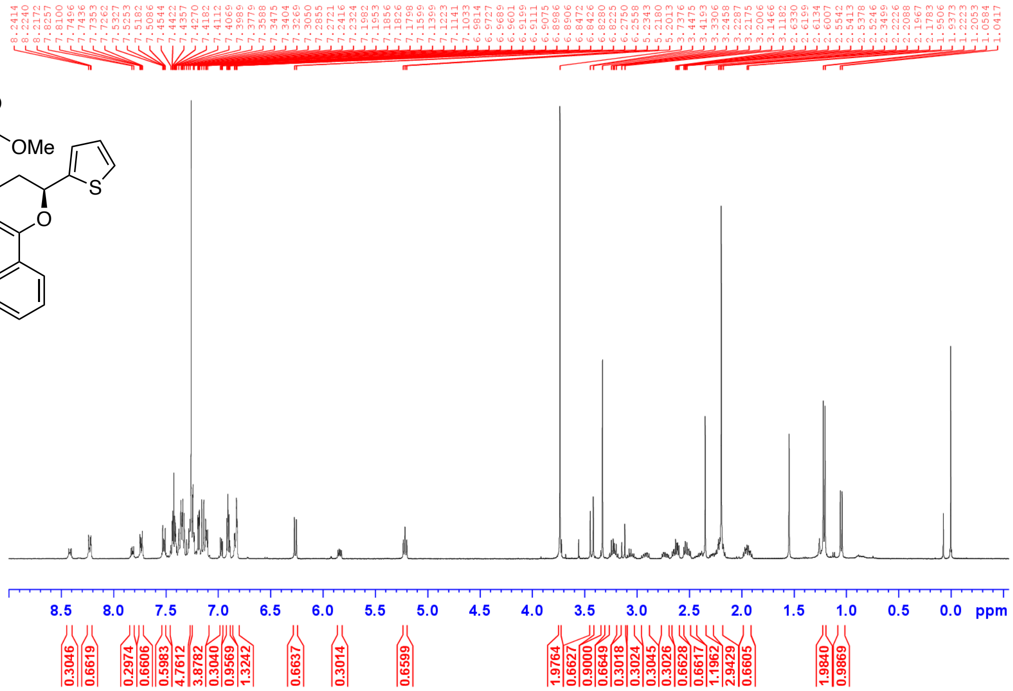

^{13}C{^{1}H} NMR (100 MHz, $CDCl_3$)

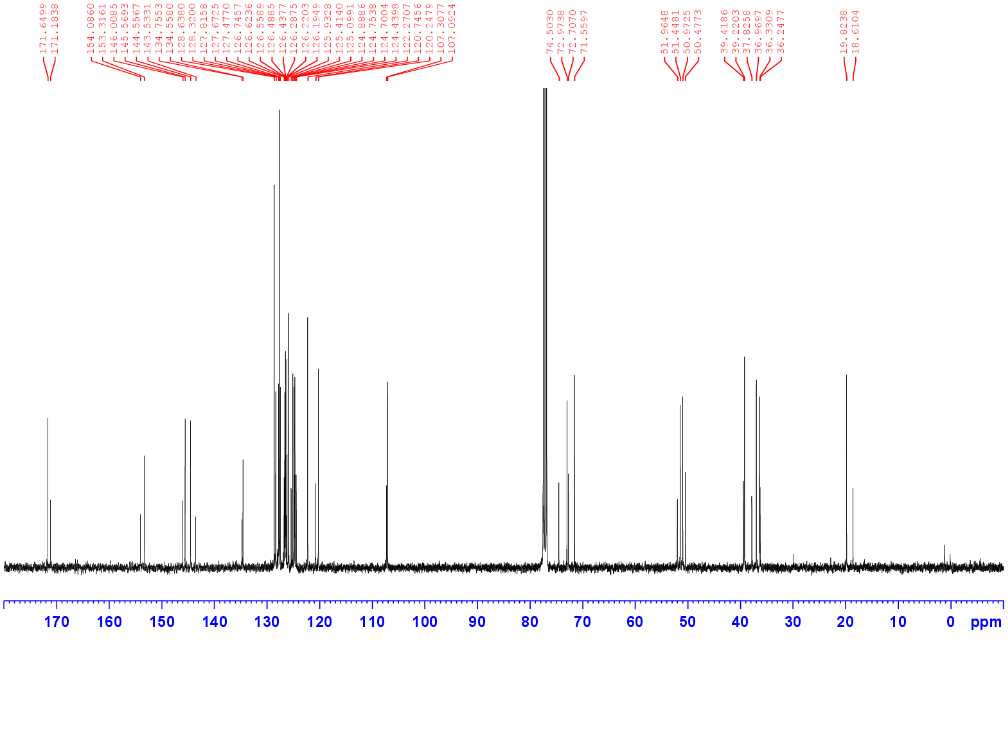

[^{1}H, ^{13}C{^{1}H}, and ^{19}F{^{1}H} NMR Spectra of **3al**]

^{1}H NMR
(400 MHz, $CDCl_3$)

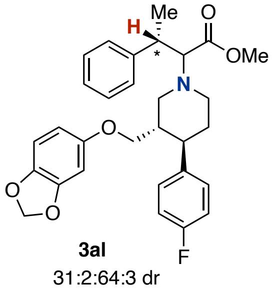

3al
31:2:64:3 dr

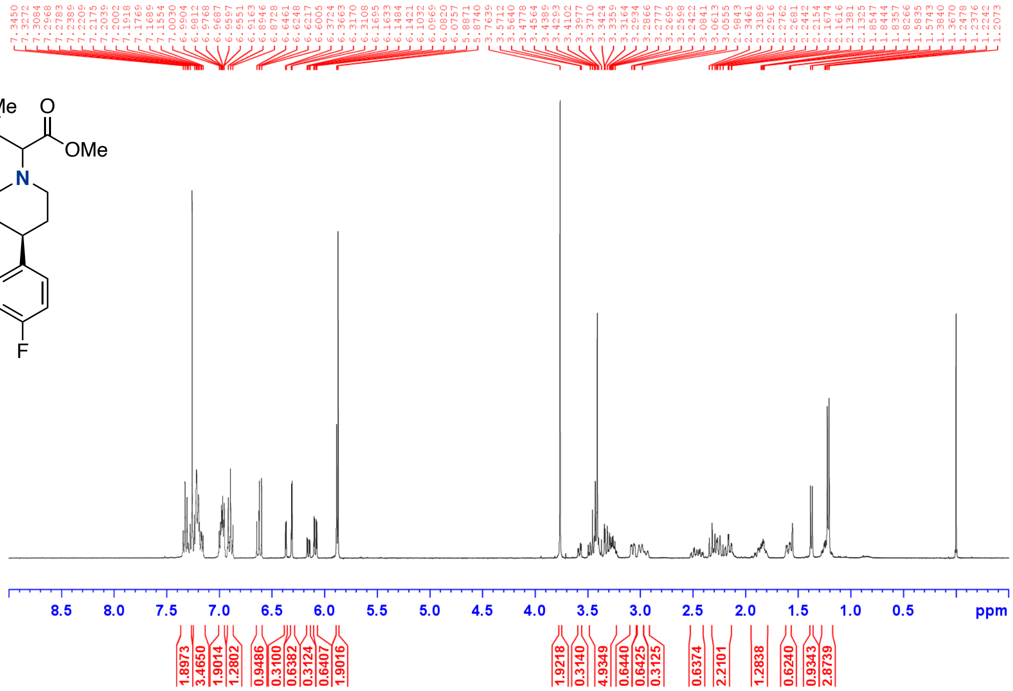

^{13}C{^{1}H} NMR
(100 MHz, $CDCl_3$)

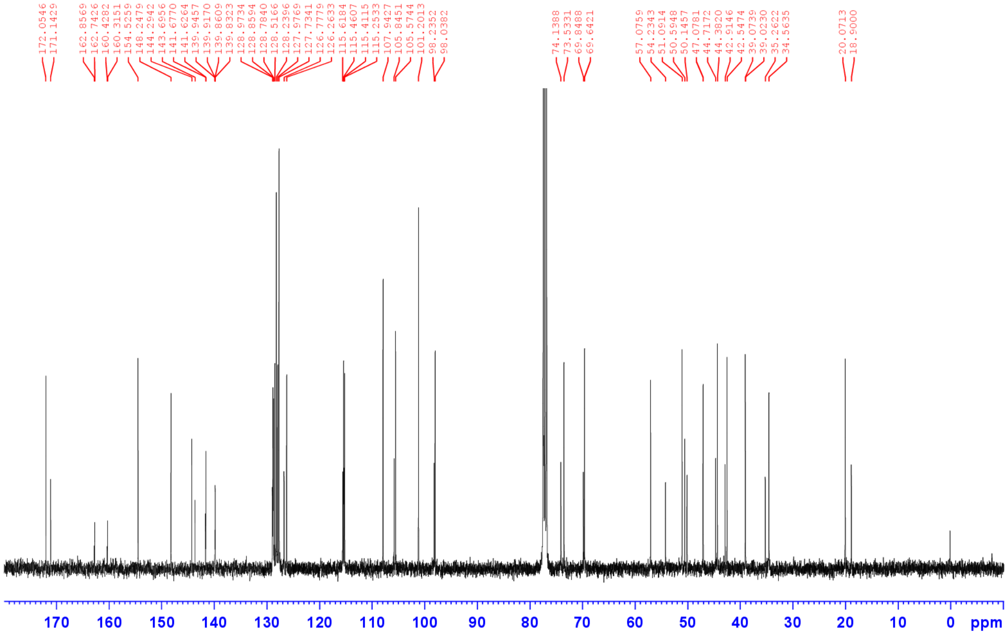

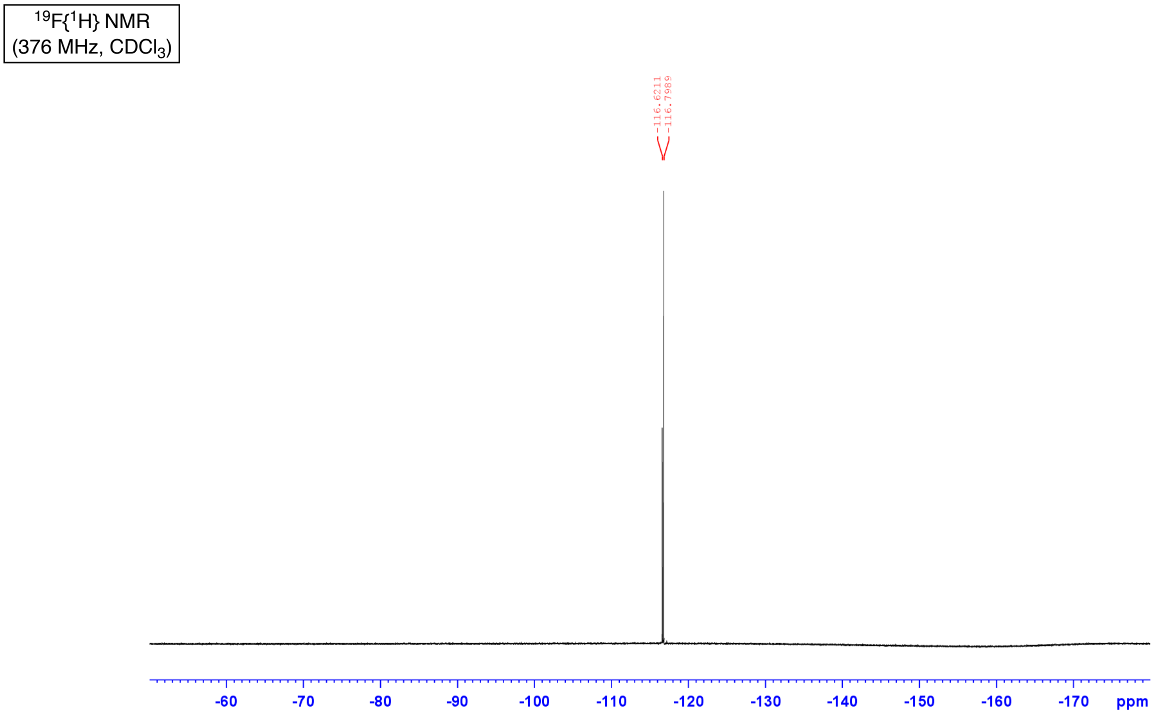
$^{19}F\{^1H\}$ NMR
(376 MHz, $CDCl_3$)
-116.6211
-116.7989
-60
-70
-80
-90
-100
-110
-120
-130
-140
-150
-160
-170
ppm

[^{1}H and ^{13}C{^{1}H} NMR Spectra of **3bm**]

^{1}H NMR
(400 MHz, $CDCl_3$)

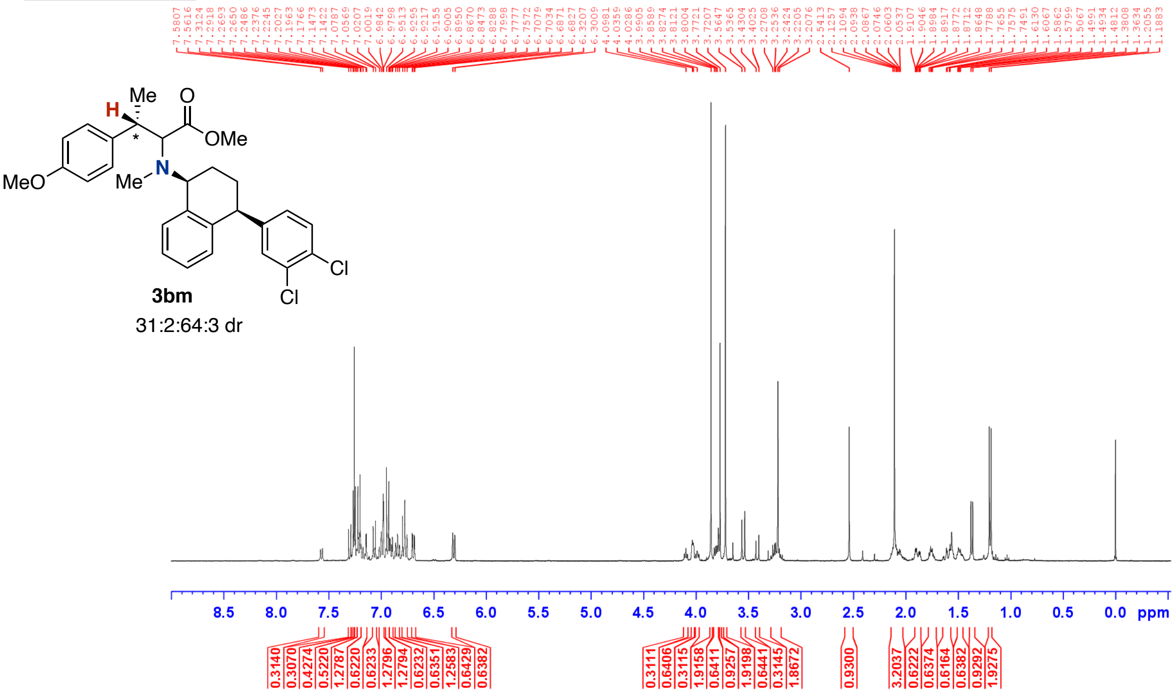

^{13}C{^{1}H} NMR
(100 MHz, $CDCl_3$)

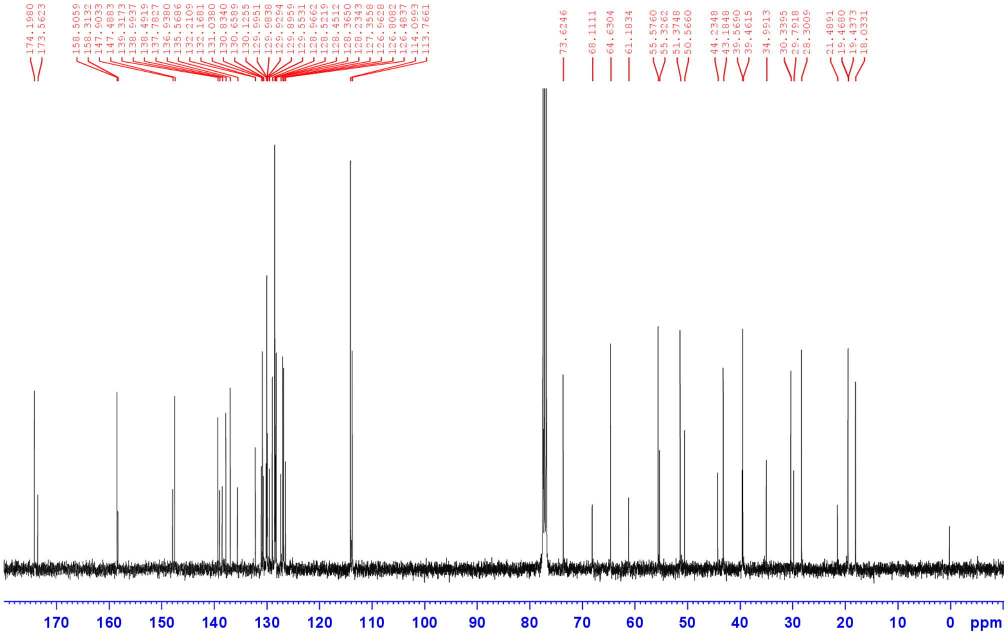

[^{1}H and $^{13}C\{^{1}H\}$ NMR Spectra of **3Ga**]

^{1}H NMR
(400 MHz, $CDCl_3$)

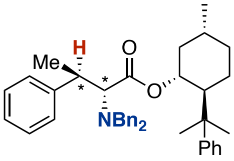

3Ga

<1:9:<1:91 dr

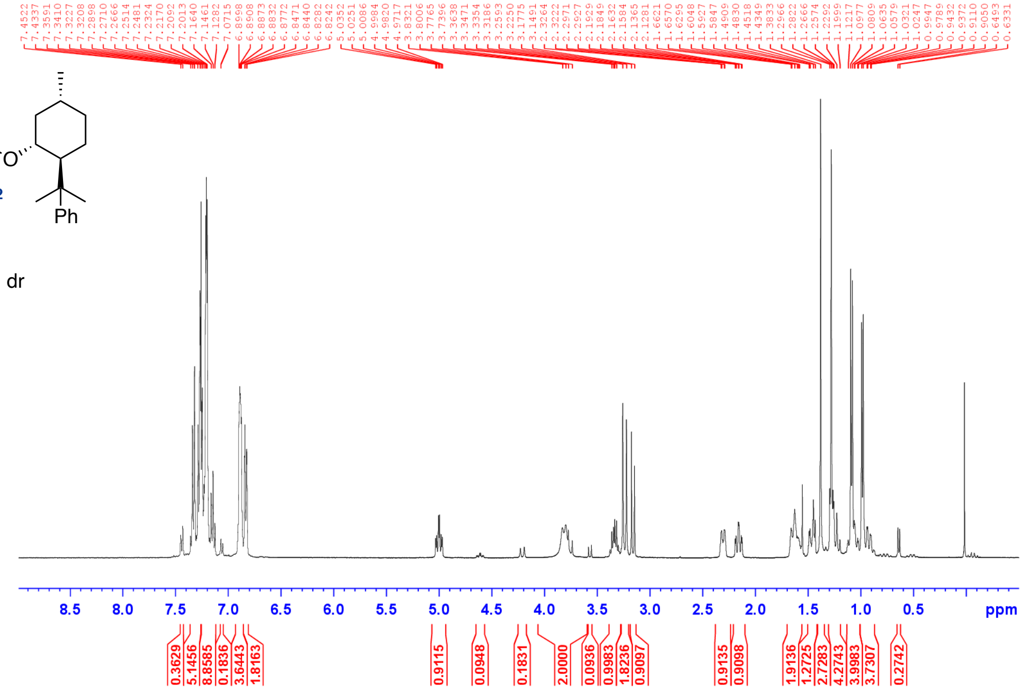

$^{13}C\{^{1}H\}$ NMR
(100 MHz, $CDCl_3$)

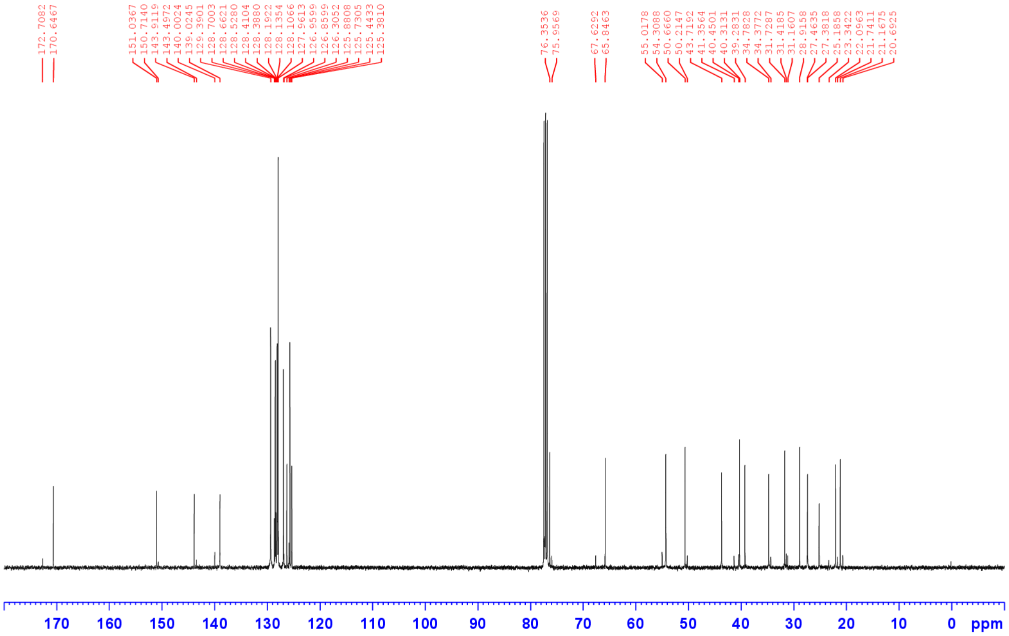

[^{1}H and ^{13}C{^{1}H} NMR Spectra of *syn*-**5**]

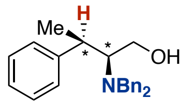

syn-5

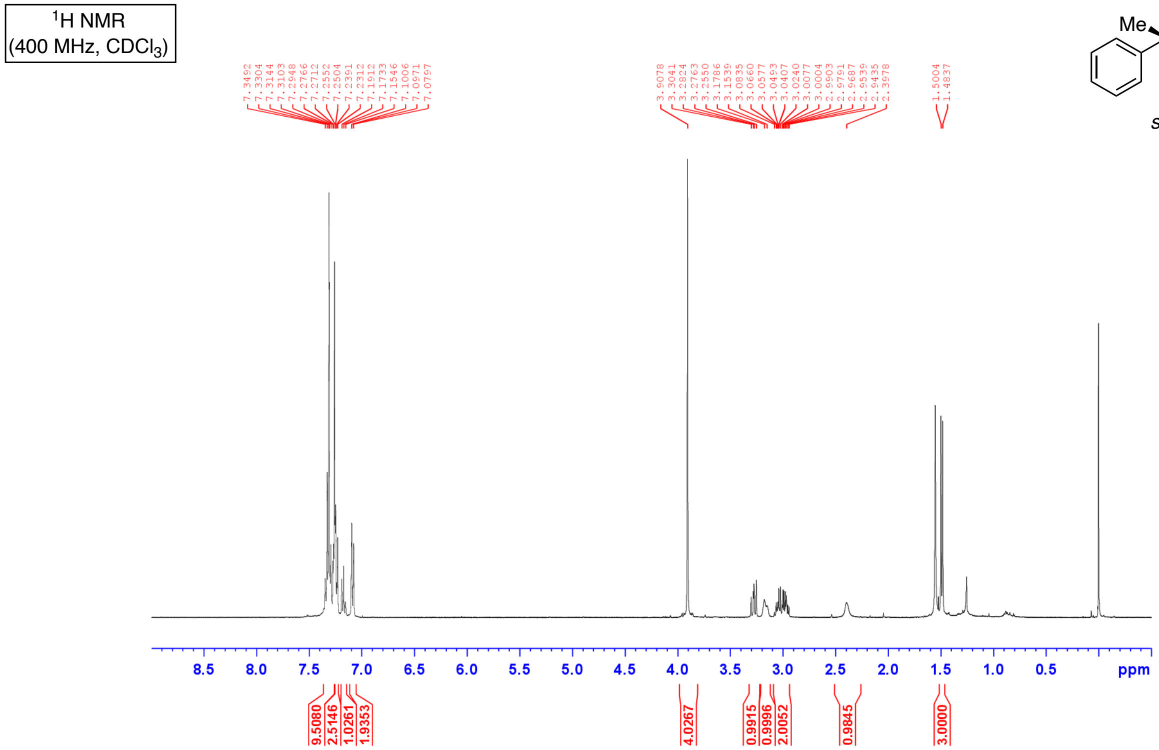

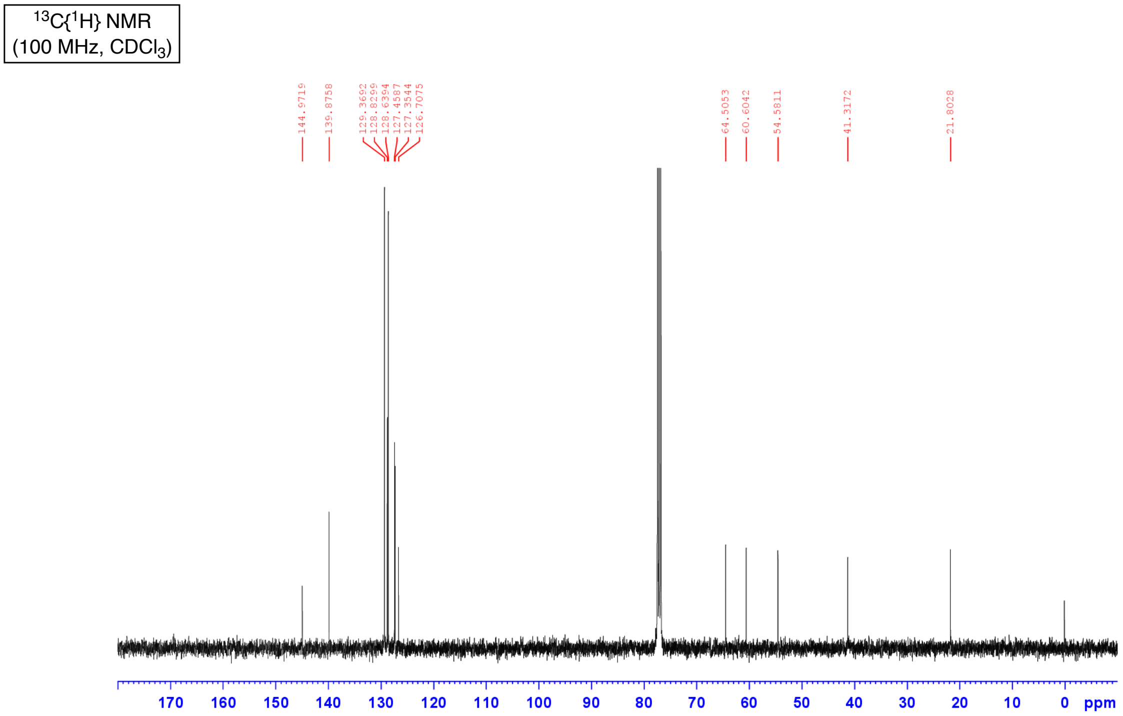

[^{1}H and $^{13}C\{^{1}H\}$ NMR Spectra of *anti*-**5**]

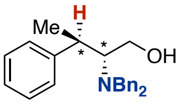

anti-**5**

^{1}H NMR
(400 MHz, $CDCl_3$)

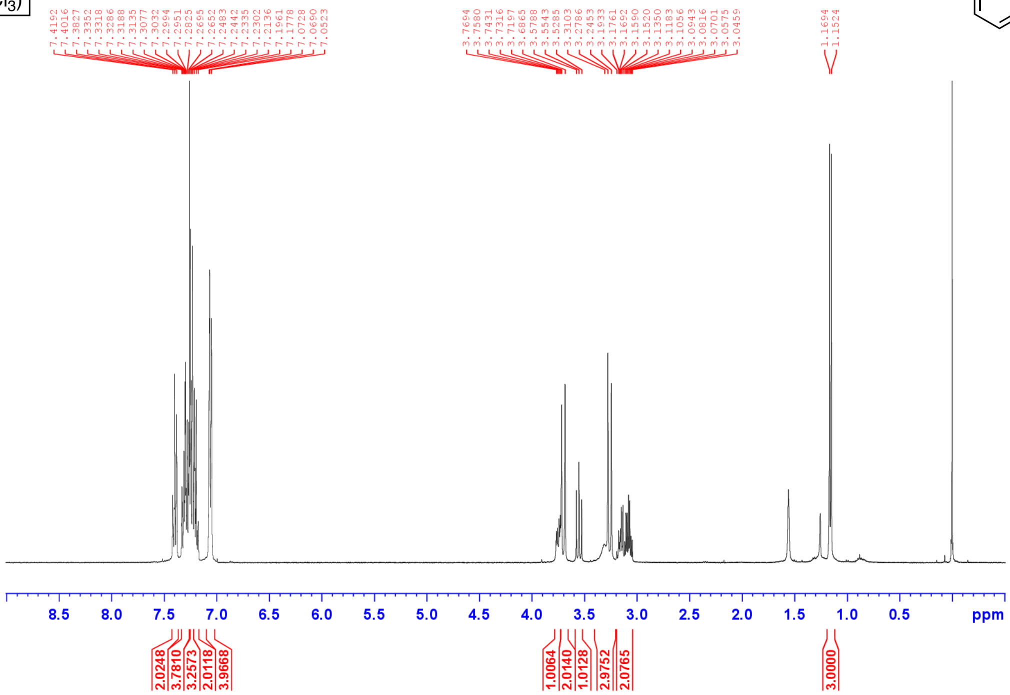

$^{13}C\{^{1}H\}$ NMR
(100 MHz, $CDCl_3$)

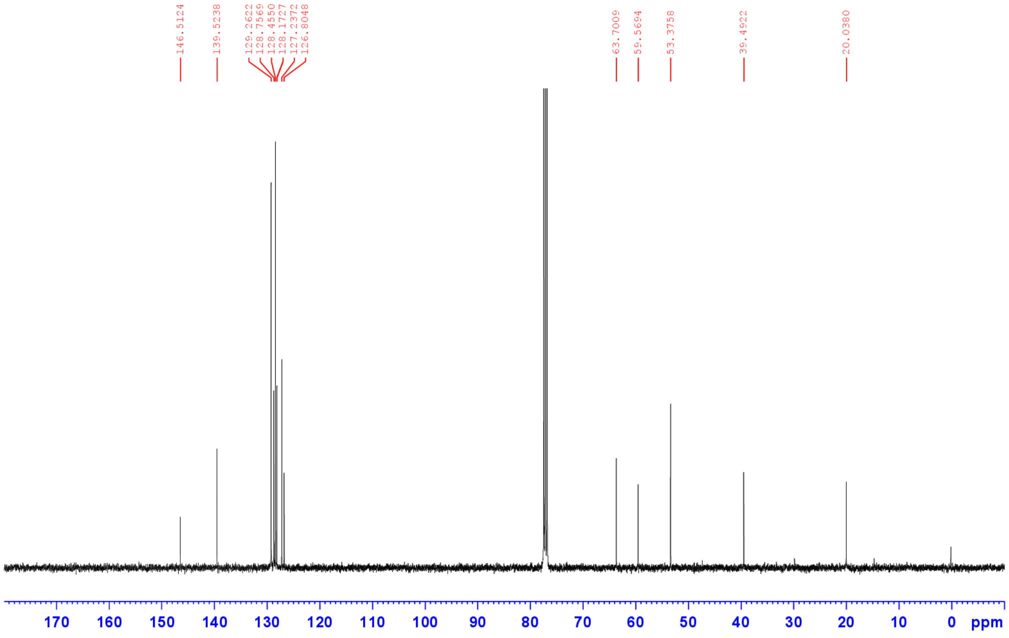

[^{1}H and ^{13}C{^{1}H} NMR Spectra of *anti*-**6**]

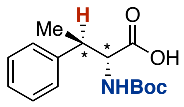

anti-**6**

^{1}H NMR
(400 MHz, $CDCl_3$)

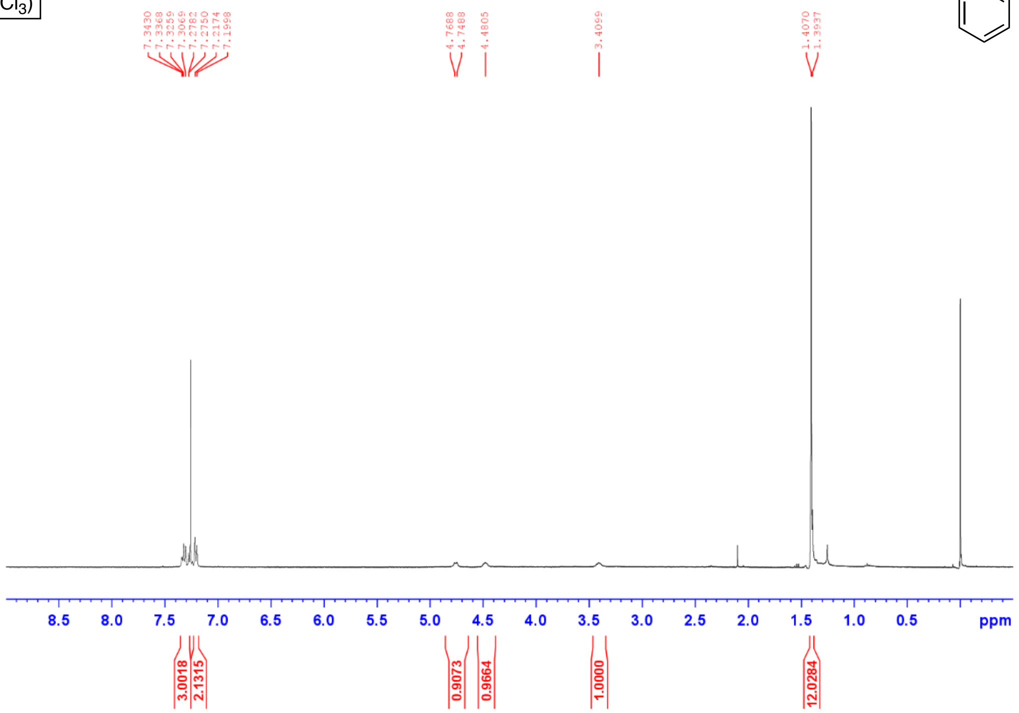

^{13}C{^{1}H} NMR
(100 MHz, $CDCl_3$)

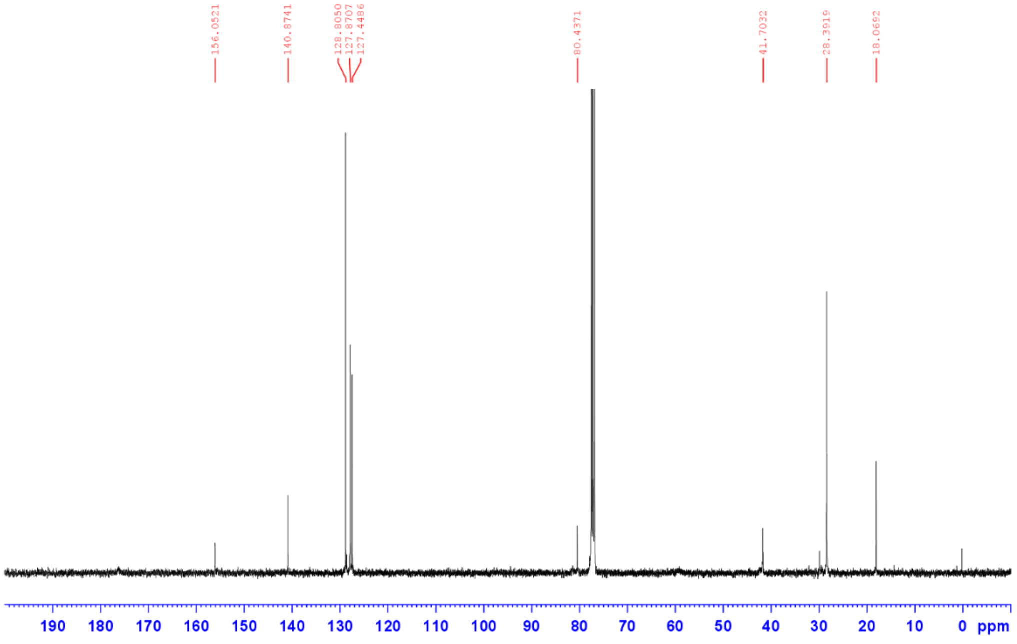

[^{1}H and ^{13}C{^{1}H} NMR Spectra of **3Gd**]

^{1}H NMR
(400 MHz, $CDCl_3$)

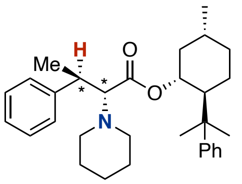

3Gd

<1:5:<1:95 dr

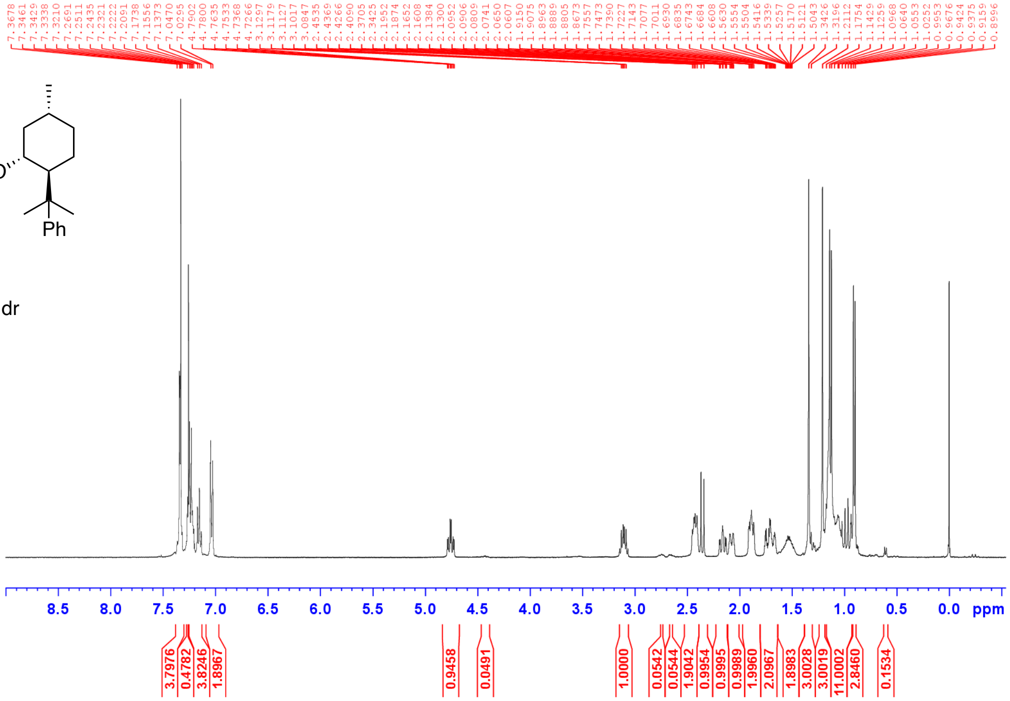

^{13}C{^{1}H} NMR
(100 MHz, $CDCl_3$)

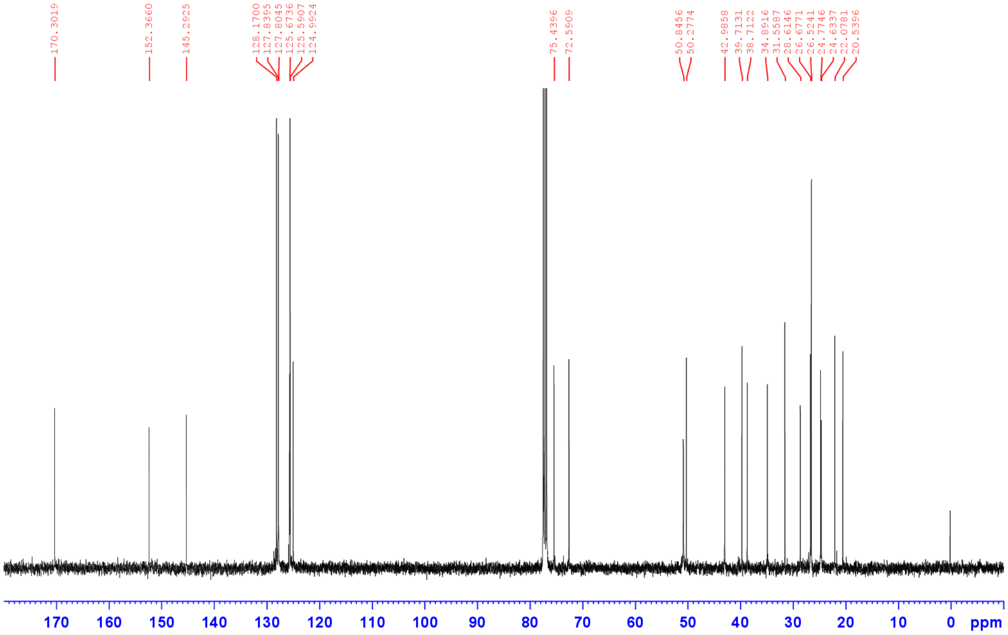

[^{1}H and ^{13}C{^{1}H} NMR Spectra of **3Ha**]

^{1}H NMR
(400 MHz, $CDCl_3$)

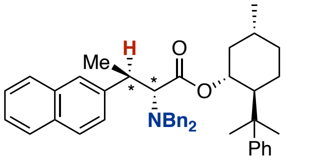

3Ha
<1:8:<1:92 dr

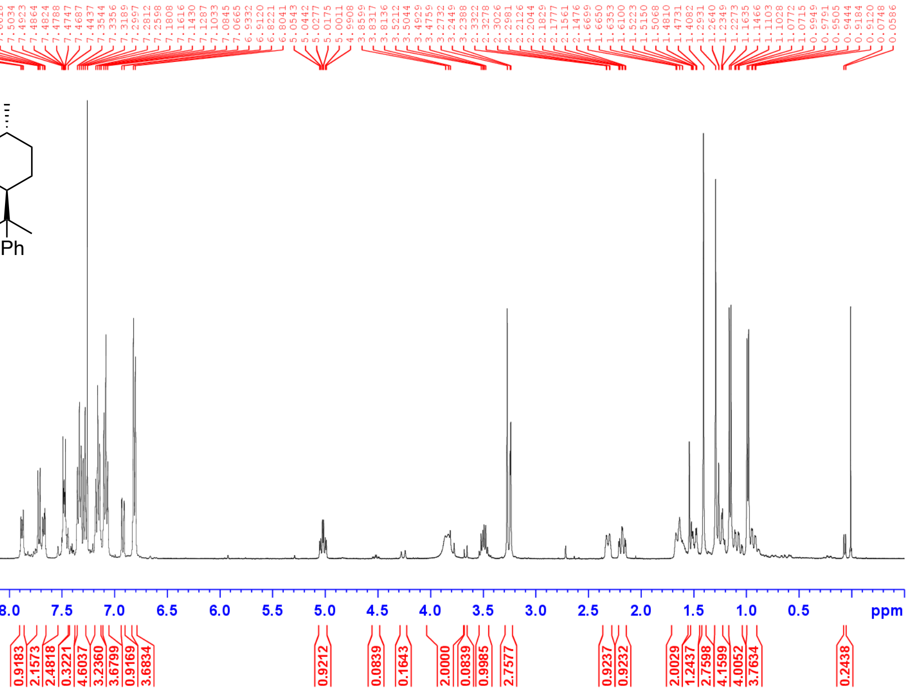

^{13}C{^{1}H} NMR
(100 MHz, $CDCl_3$)

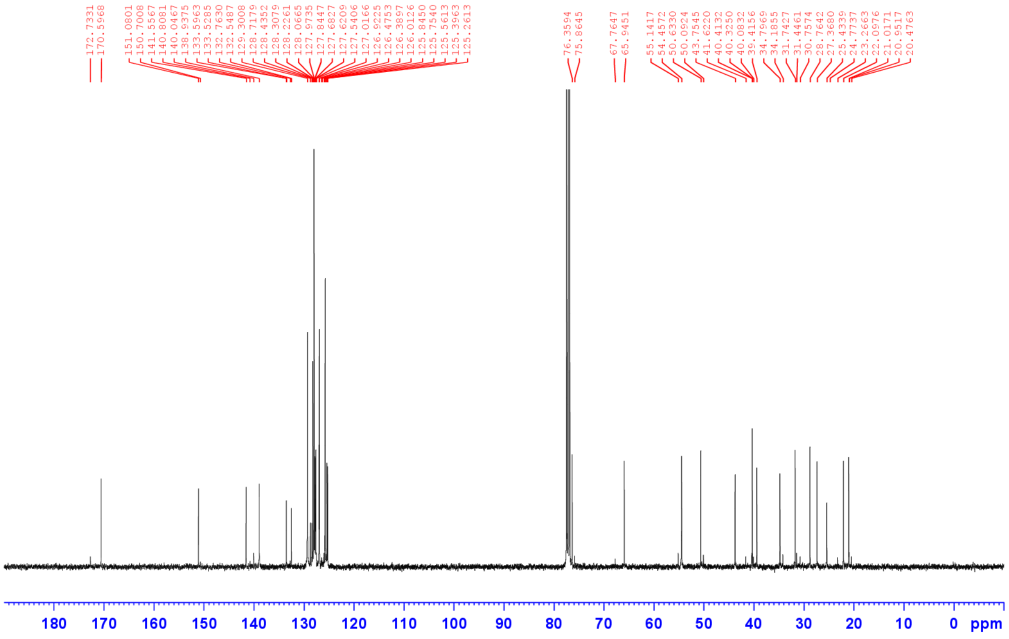

[^{1}H and ^{13}C{^{1}H} NMR Spectra of **3Ia**]

^{1}H NMR
(400 MHz, $CDCl_3$)

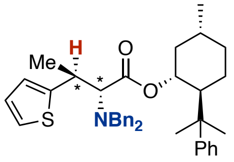

3Ia

<1:8:<1:92 dr

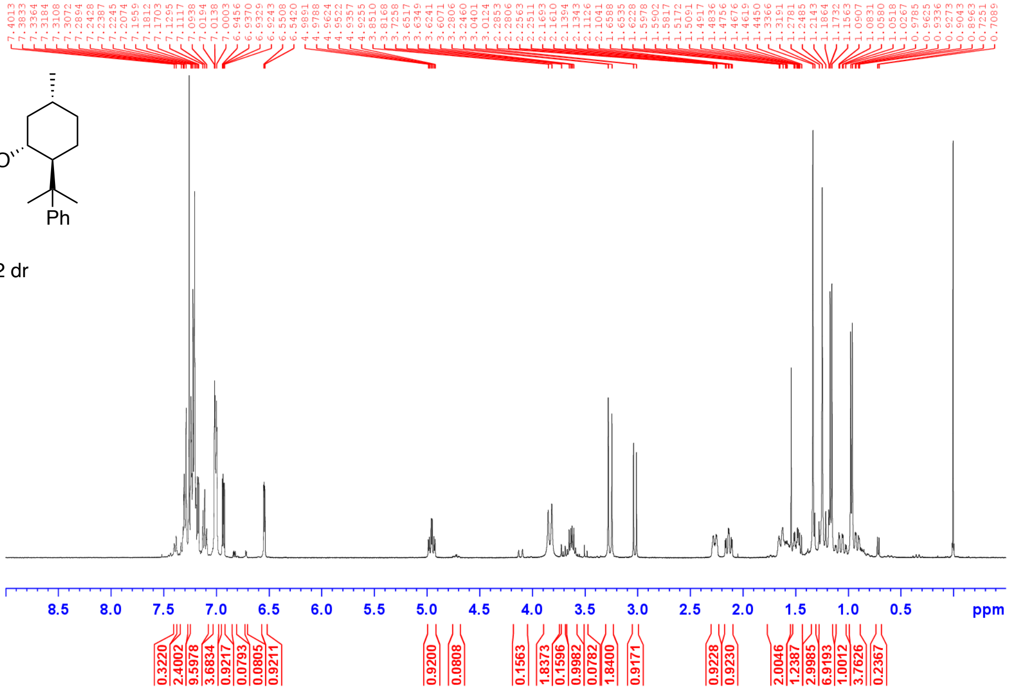

^{13}C{^{1}H} NMR
(100 MHz, $CDCl_3$)

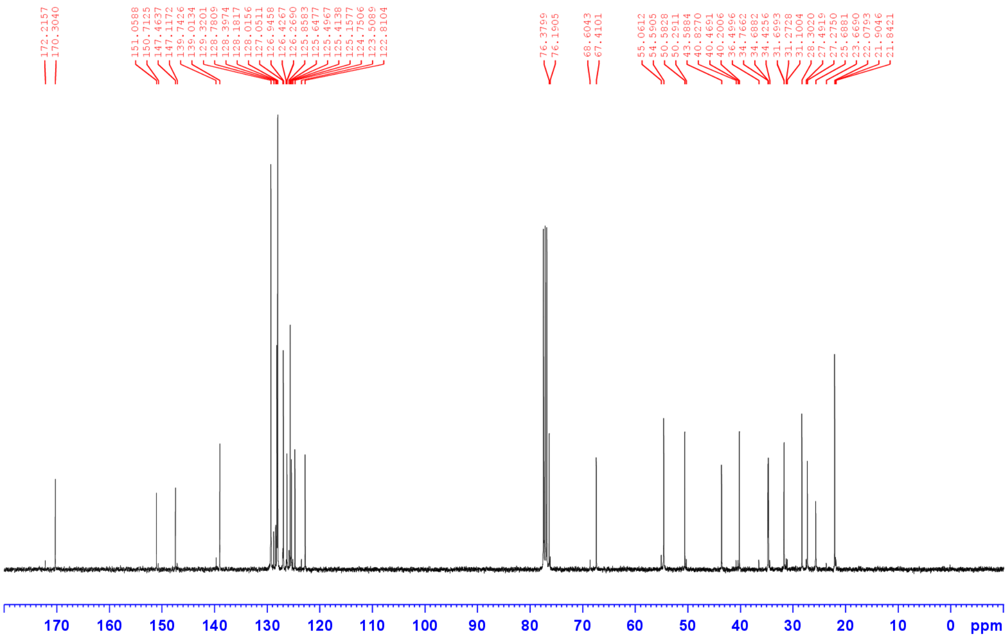

[^{1}H and ^{13}C{^{1}H} NMR Spectra of **3Ja**]

^{1}H NMR
(400 MHz, $CDCl_3$)

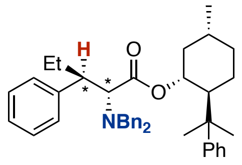

3Ja
<1:9:<1:91 dr

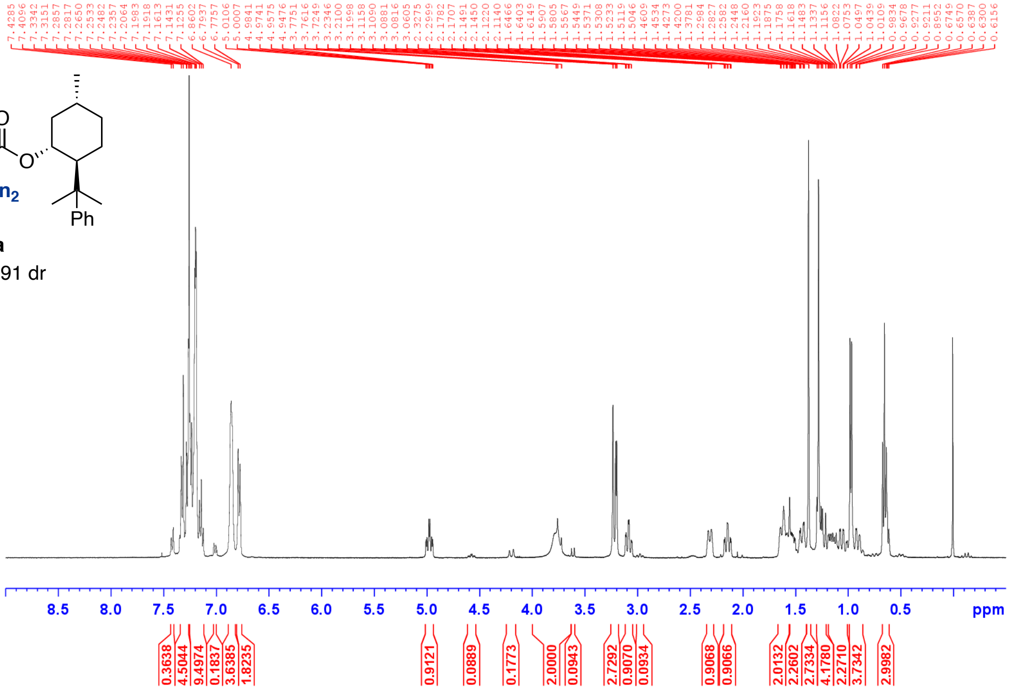

^{13}C{^{1}H} NMR
(100 MHz, $CDCl_3$)

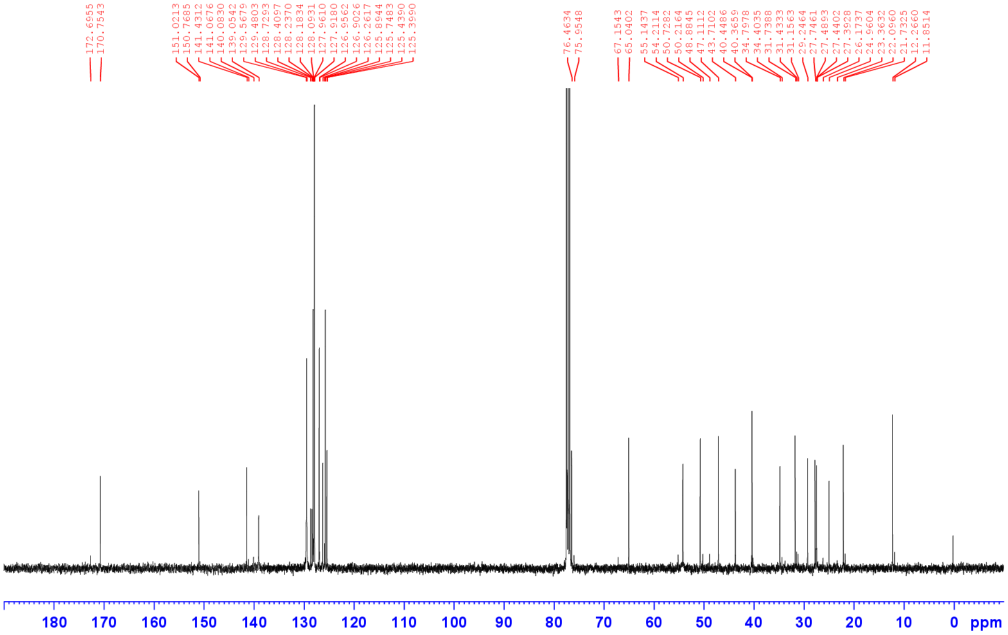

[^{1}H and $^{13}C\{^{1}H\}$ NMR Spectra of **3Ka**]

^{1}H NMR
(400 MHz, $CDCl_3$)

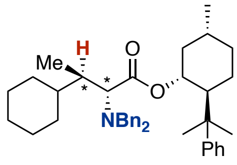

3Ka

<1:17:<1:83 dr

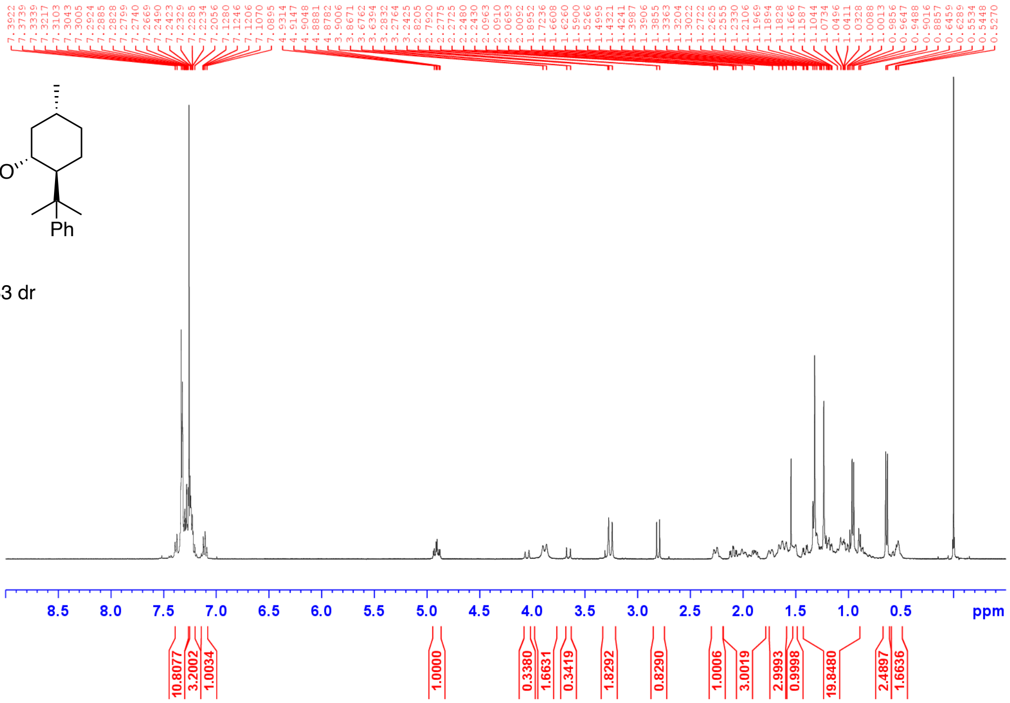

$^{13}C\{^{1}H\}$ NMR
(100 MHz, $CDCl_3$)

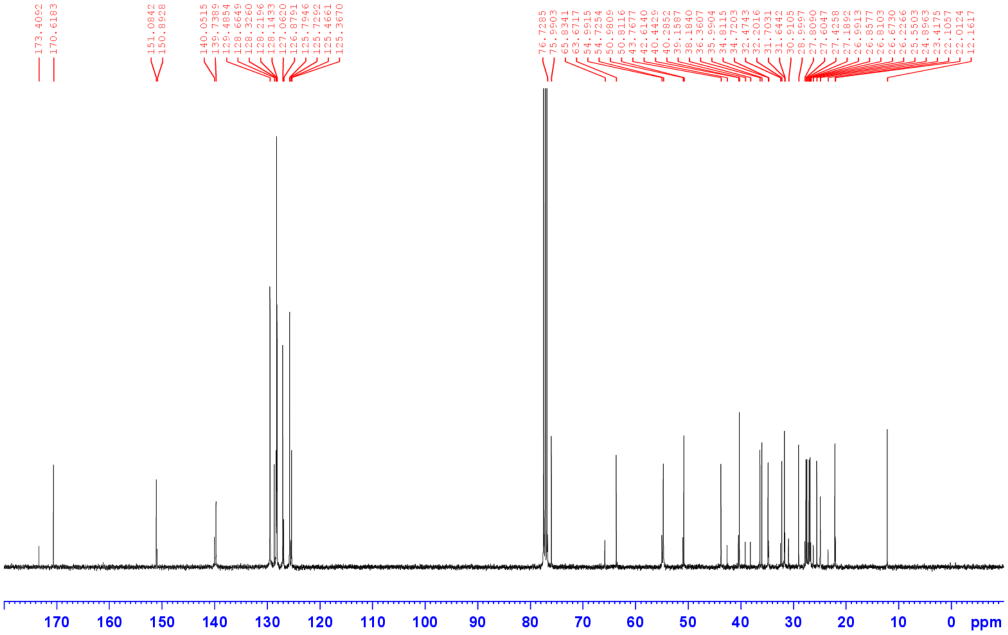

[^{1}H and ^{13}C{^{1}H} NMR Spectra of **3La**]

^{1}H NMR
(400 MHz, $CDCl_3$)

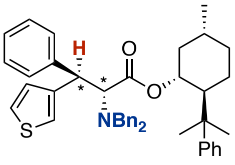

3La
<1:28:<1:72 dr

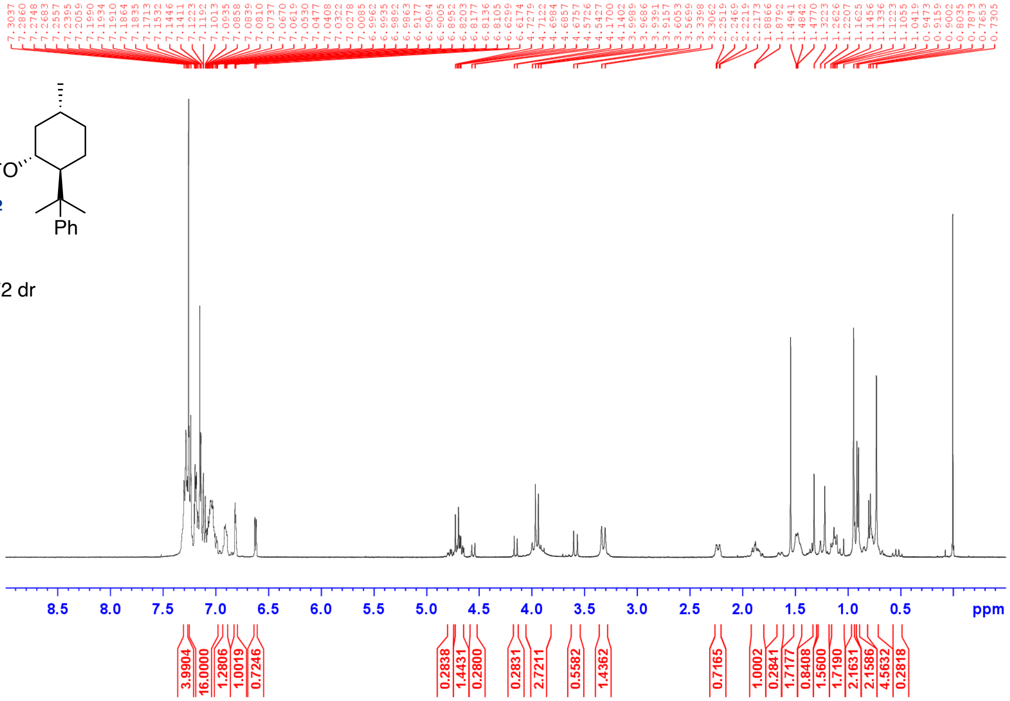

^{13}C{^{1}H} NMR
(100 MHz, $CDCl_3$)

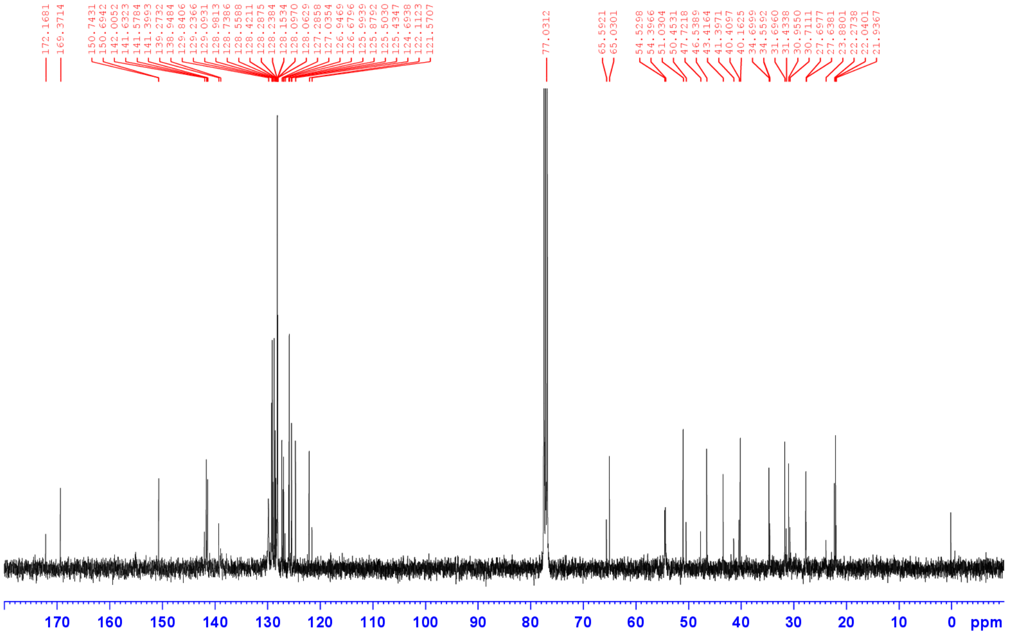

[^{1}H and ^{13}C{^{1}H} NMR Spectra of **3Ma**]

^{1}H NMR
(400 MHz, $CDCl_3$)

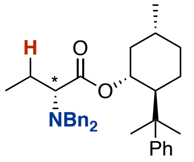

3Ma
31:69 dr

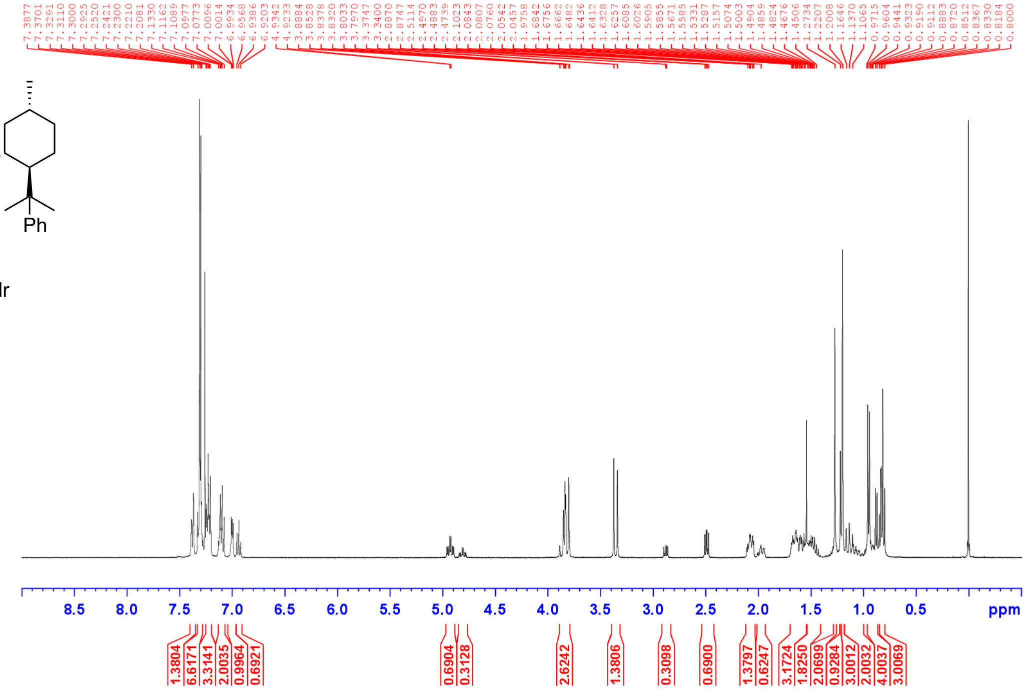

^{13}C{^{1}H} NMR
(100 MHz, $CDCl_3$)

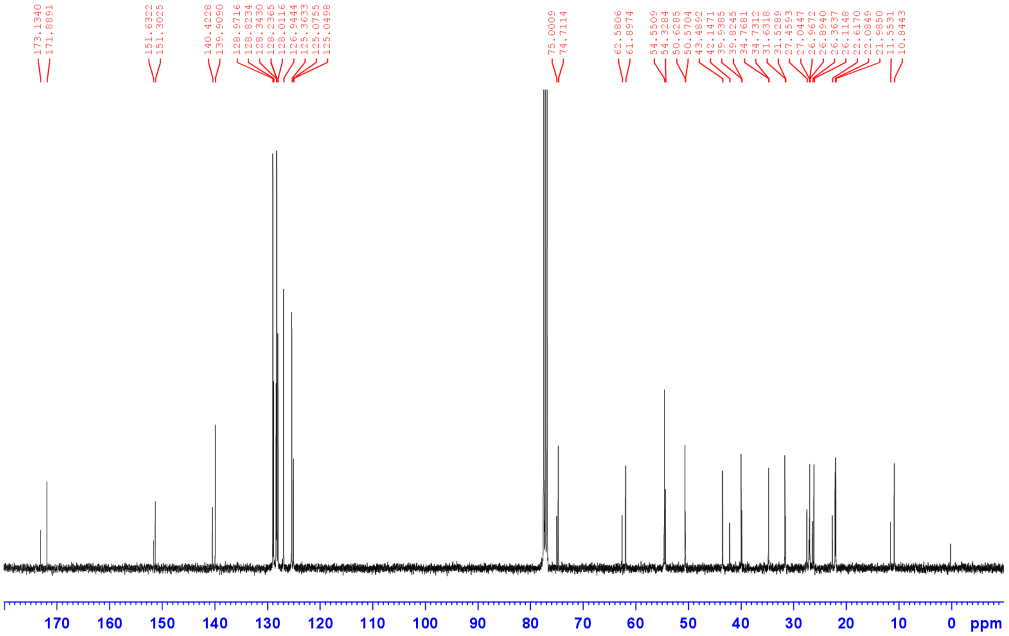

[^{1}H and ^{13}C{^{1}H} NMR Spectra of *syn*-**3aa-NH$_2$**]

^{1}H NMR
(400 MHz, $CDCl_3$)

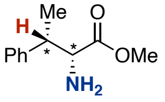

syn-**3aa-NH$_2$**

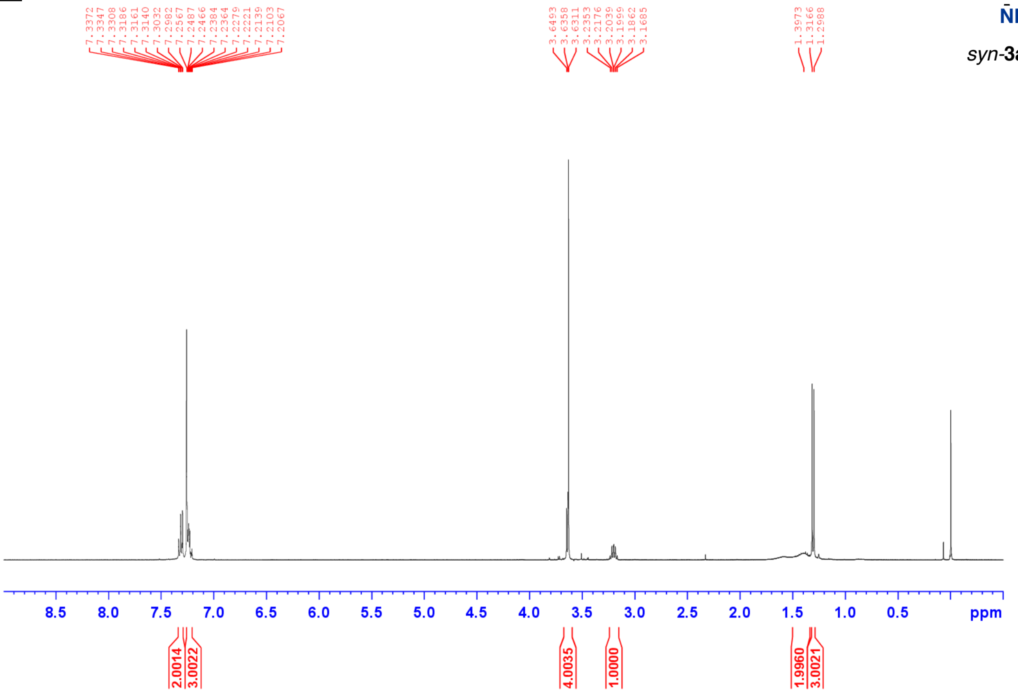

^{13}C{^{1}H} NMR
(100 MHz, $CDCl_3$)

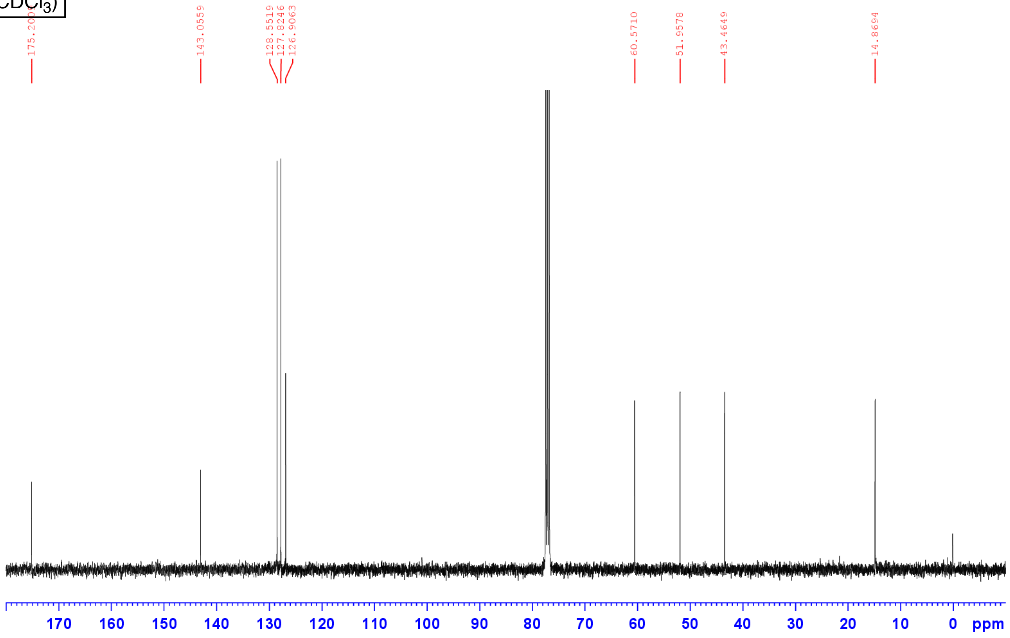

[^{1}H and ^{13}C{^{1}H} NMR Spectra of *anti*-**3aa-NH$_2$**]

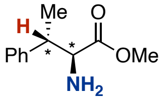

anti-**3aa-NH$_2$**

^{1}H NMR
(400 MHz, $CDCl_3$)

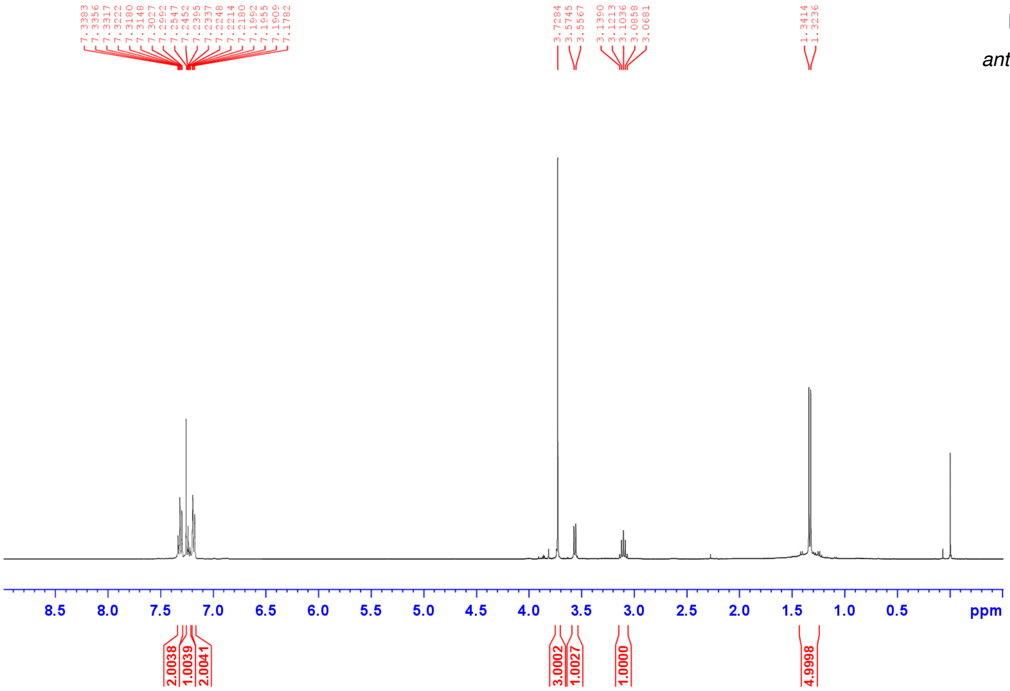

^{13}C{^{1}H} NMR
(100 MHz, $CDCl_3$)

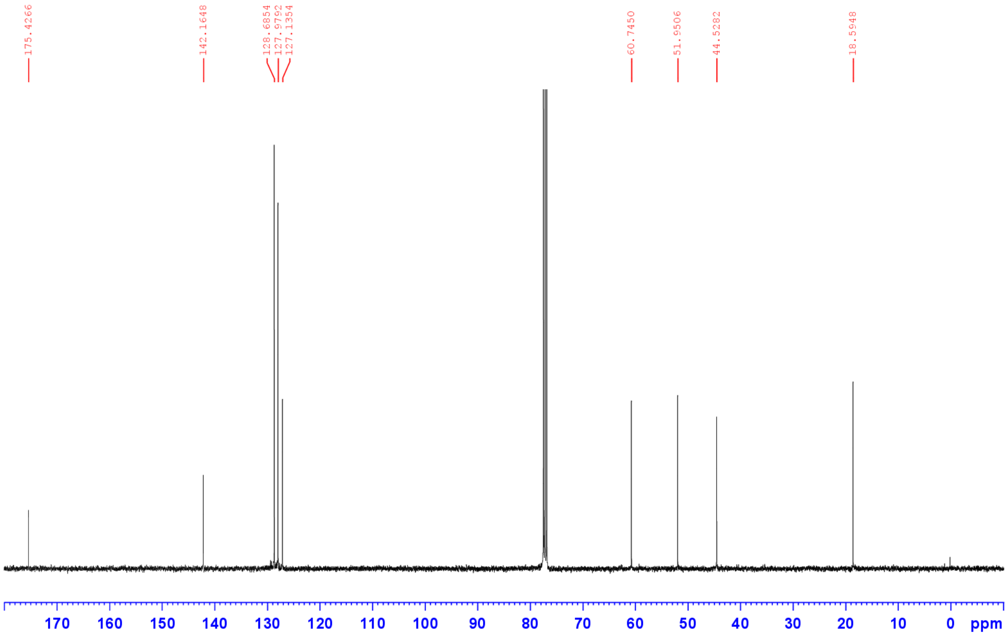

[^{1}H and ^{13}C{^{1}H} NMR Spectra of *syn*-**3wa-OH**]

^{1}H NMR
(400 MHz, $CDCl_3$)

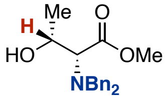

syn-**3wa-OH**

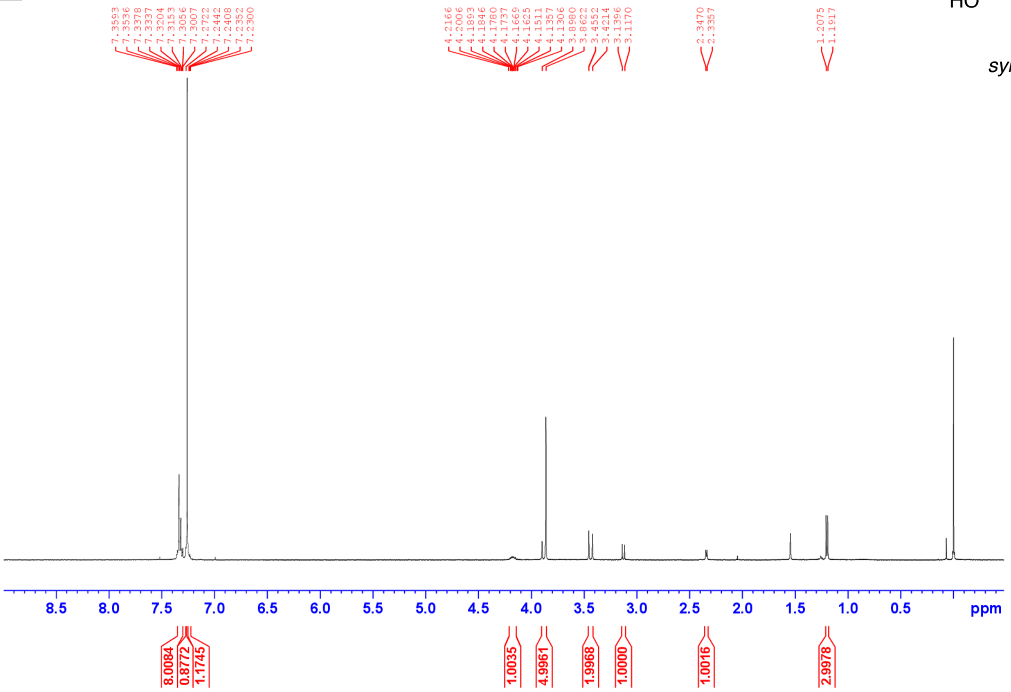

^{13}C{^{1}H} NMR
(100 MHz, $CDCl_3$)

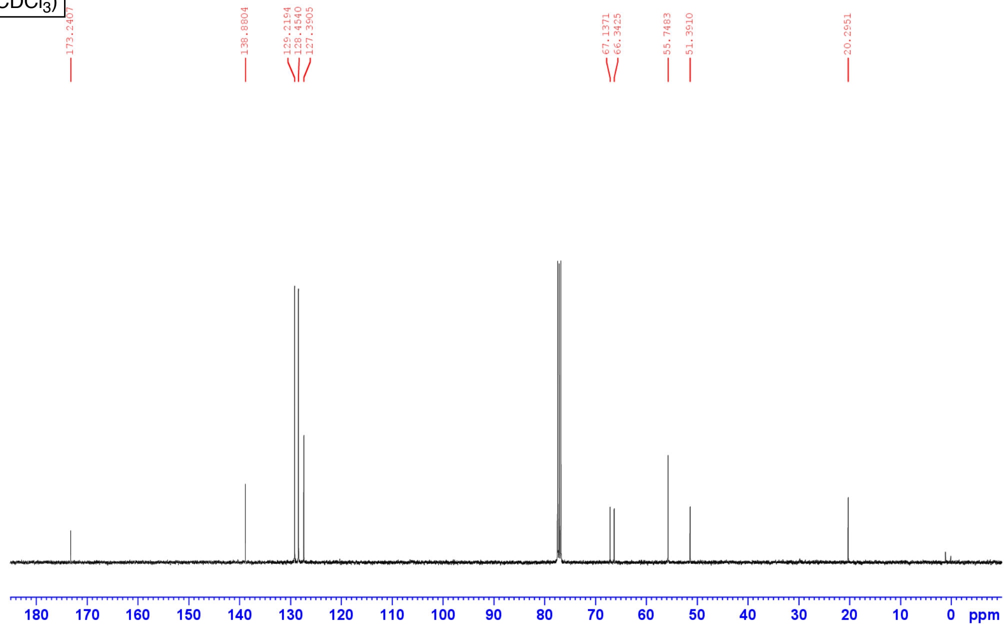

References

(S1) (*a*) S. Bovo, A. Scrivanti, M. Bertoldini, V. Beghetto and U. Matteoli, *Synthesis,* 2008, **2008**, 2547; (*b*) Z.-C. Duan, X.-P. Hu, C. Zhang and Z. Zheng, *J. Org. Chem.,* 2010, **75**, 8319.

(S2) (*a*) R. K. Saunthwal, K. M. Saini, M. Patel and A. K. Verma, *Tetrahedron,* 2017, **73**, 2415; (*b*) Y. Nakao, K. S. Kanyiva, S. Oda and T. Hiyama, *J. Am. Chem. Soc.,* 2006, **128**, 8146.

(S3) (*a*) Y. Li, K. Dong, Z. Wang and K. Ding, *Angew. Chem., Int. Ed.,* 2013, **52**, 6748; (*b*) R. K. Dieter and K. Lu, *J. Org. Chem.,* 2002, **67**, 847.

(S4) A. Kar, N. P. Argade and N. P. *J. Org. Chem.,* 2002, **67**, 7131.

(S5) A. Link, Y. Zhou and S. L. Buchwald, *Org. Lett.,* 2020, **22**, 5666.

(S6) Y.-H. Xu, L.-H. Wu, J. Wang and T.-P. Loh, *Chem. Commun.,* 2014, **50**, 7195.

(S7) (*a*) Y. Soltani, L. C. Wilkins and R. L. Melen, *Angew. Chem., Int. Ed.,* 2017, **56**, 11995; (*b*) Y. Kato, K. Niiyama, T. Nemoto, H. Jona, A. Akao, S. Okada, Z. J. Song, M. Zhao, Y. Tsuchiya, K. Tomimoto and T. Mase, *Tetrahedron,* 2002, **58**, 3409.

(S8) (*a*) A. M. Berman and J. S. Johnson, *J. Am. Chem. Soc.,* 2004, **126**, 5680; (*b*) A. M. Berman and J. S. Johnson, *J. Org. Chem*., 2006, **71**, 219.

(S9) T. Fujihara, A. Sawada, T. Yamaguchi, Y. Tani, J. Terao and Y. Tsuji, *Angew. Chem., Int. Ed.,* 2017, **56**, 15391.

(S10) G. Guanti, L. Banfi, E. Narisano and C. Scolastico, *Tetrahedron,* 1988, **44**, 3671.

(S11) S. D. Bull, S. G. Davies, S. W. Epstein, A. C. Garner, N. Mujtaba, R. M. Roberts, E. D. Savory, A. D. Smith, J. A. Tamayo and D. J. Watkin, *Tetrahedron,* 2006, **62**, 791.

(S12) The ^{1}H NMR data of *anti*-**6** was in agreement with the reported value; M. Pastó, A. Moyano, M. A. Pericàs and A. Piera, *J. Org. Chem.,* 1997, **62**, 8425.
